# Preparation of mono-substituted malonic acid half oxyesters (SMAHOs)

**DOI:** 10.3762/bjoc.17.135

**Published:** 2021-08-18

**Authors:** Tania Xavier, Sylvie Condon, Christophe Pichon, Erwan Le Gall, Marc Presset

**Affiliations:** 1 Université Paris Est Créteil, CNRS, ICMPE, 2 rue Henri Dunant, F-94320 Thiais, France

**Keywords:** alkylation, esterification, malonate, saponification, SMAHO

## Abstract

The use of mono-substituted malonic acid half oxyesters (SMAHOs) has been hampered by the sporadic references describing their preparation. An evaluation of different approaches has been achieved, allowing to define the best strategies to introduce diversity on both the malonic position and the ester function. A classical alkylation step of a malonate by an alkyl halide followed by a monosaponification gave access to reagents bearing different substituents at the malonic position, including functionalized derivatives. On the other hand, the development of a monoesterification step of a substituted malonic acid derivative proved to be the best entry for diversity at the ester function, rather than the use of an intermediate Meldrum acid. Both these transformations are characterized by their simplicity and efficiency, allowing a straightforward access to SMAHOs from cheap starting materials.

## Introduction

Malonic acid half oxyesters (MAHOs), also known as alkyl hydrogen malonates or hemimalonates, constitute an attractive class of pronucleophiles in the design of eco-compatible syntheses [1]. Indeed, they can serve as efficient precursors of ester enolates through decarboxylation [2–3], generally using a substoichiometric amount of a weak base. Another advantage inherent to this class of reagents is their easy purification by a classical acid/base work-up thanks to the presence of the carboxylic acid moiety, even if they could also be purified by standard chromatography on silica gel. They have thus been used in a variety of reactions, the most widespread applications being aldol or Mannich-type addition reactions with carbonyl compounds and imines, respectively [4], and Galat olefination reactions of aldehydes [5–7]. Nevertheless, these developments have been mostly described with unsubstituted MAHOs, and the use of mono-substituted MAHOs (SMAHOs) [8–10], or disubstituted MAHOs [11], has been only occasionally reported.

**Scheme 1:**
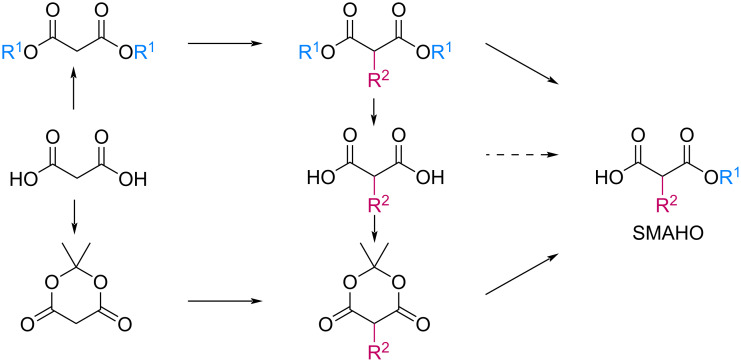
Main routes to SMAHOs.

Bibliographical data indicate that SMAHOs can be accessed by different routes (Scheme 1). The most classic one is the alkylation of a dialkyl malonate, followed by a monohydrolysis of one ester group. The alkylation is usually achieved by a nucleophilic substitution of an alkyl halide by the deprotonated malonate [12–19], but other strategies could be envisioned: Cu-catalyzed arylation reactions for aryl-substituted MAHOs [20–23]; Knoevenagel/reduction sequences for benzyl-substituted MAHOs [24–27]; and a Michael addition for 3'-oxoalkyl-substituted MAHOs [28]. As studied by Niwayama [29], the hydrolysis step is generally achieved by saponification using alcoholic KOH (or NaOH) [30–36], but other selective cleavages of one ester group are possible [37–38]. Despite its efficiency, this strategy remains limited regarding the nature of the alkoxy group of the ester function (generally Me or Et), as it arises from the parent malonate. In order to introduce diversity at this position, the privileged route is based on the opening of a Meldrum's acid derivative with an alcohol [39]. In this route, the Meldrum's acid is first alkylated, and the desired reagent is easily obtained by mixing this precursor with one equivalent of the requisite alcohol under reflux, generally in toluene [40–45]. Some reagents were also prepared by other routes [46–48], such as the α-carboxylation of an ester [49], the selective functionalization of a bromomalonate derivative [50], or the monoesterification of a malonic acid derivative [51–53].

However, even if the above-mentioned strategies appear obvious for the preparation of the simplest SMAHOs, they are not sufficiently documented to cover the preparation of more complex versions of these reagents, apart from 2-amido-MAHOs for which the parent aminomalonate is commercially available. Indeed, it is very rare to find two identical procedures or stoichiometries, and these methods are used for the synthesis of a limited range of SMAHOs, presenting little diversity. Within the framework of a project pertaining to the use of SMAHOs in Galat [54] and Mannich reactions [55], we were confronted with a lack of pertinent bibliographical information, which led us to investigate the few reported synthetic routes.

## Results and Discussion

### Substituent modification – alkylation/saponification route

Based on the literature precedents, it appeared logical to start the preparation of SMAHOs by the alkylation of a malonate derivative followed by a monosaponification of the resulting diester. This strategy affords the advantage of being based on commercially available malonates. We thus envisioned to achieve the alkylation step by deprotonation of a malonate with NaH followed by treatment with a halogenated compound. As our first attempts revealed a strong influence of the stoichiometry and the nature of the alkylating reagent on both efficiency and selectivity, we decided to perform a quick optimization study. As a model reaction, we evaluated the influence of various parameters on the reaction between diethyl malonate (**1b**) and iodobutane (**2d**) or bromobutane (**2d'**) in the presence of a stoichiometric amount of sodium hydride (Table 1). Using a 1:1:1 mixture of **1b**/**2d**/NaH in DMF (*c* = 1.0 M) at rt, the desired monoalkylated product **3bd** was isolated in 55% yield (Table 1, entry 1). By studying the stoichiometry of the reaction, we found easier to use a slight excess of the malonate to reduce the amount of the undesired dialkylated product (Table 1, entries 2 and 3). However, in all cases, the reaction mixture turned out to be very hard to stir, resulting in moderately reproducible results. We thus decided to decrease the concentration of the reaction to 0.5 M, resulting in a better 75% yield (Table 1, entry 4), whereas the use of THF led to a less efficient transformation (60%, Table 1, entry 5). The use of the brominated analog **2d'** was also possible, albeit in lower 64% yield (Table 1, entry 6).

**Table 1:** Optimization of the alkylation step.

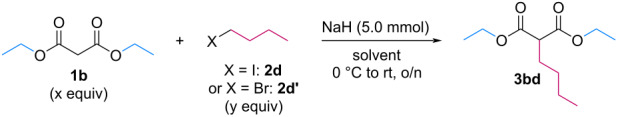					

entry	x	X	y	solvent	yield (%)^a^

1	1.0	I	1.0	DMF^b^	55
2	1.0	I	1.1	DMF^b^	68
3	1.1	I	1.0	DMF^b^	70
4	1.1	I	1.0	DMF^c^	75
5	1.1	I	1.0	THF^c^	60
6	1.1	Br	1.0	DMF^c^	64

^a^Isolated yields; ^b^*c* = 1.0 M; ^c^*c* = 0.5 M.

These optimized conditions were then applied to a variety of alkyl halides and the corresponding SMAHOs were obtained after monosaponification of the resulting intermediates (Table 2). In most cases, the desired products could be isolated in a pure form after a simple aqueous work-up based on their acidic properties. This sequence allowed the preparation of SMAHOs bearing different groups at the malonic position in moderate to good yields. Various primary aliphatic alkyl halides could be used (**3aa**, 50%; **3ab**, 40%; **3ac**, 75%; **3bd**, 93%) and the reaction was also possible using a secondary alkyl halide (**3be**, 52%) with a longer reaction time. The alkylation step was obviously more efficient with primary activated alkyl halides such as allyl (**3bf**, 81%) or benzyl (**3bh**, 76%), but lower with a base-sensitive propargyl group (**3bg**, 58%). More functionalized lateral chains like a chlorobutyl group could be introduced efficiently (**3bj**, 91%) and the use of protected aminated or hydroxylated derivatives led to lower but still useful yields (**3ak**, 41%; **3bl**, 44%; **3bm**, 51%). In all these cases, the hydrolysis step delivered the expected SMAHOs in good to excellent yields. However, this reaction should be performed in a water/alcohol mixture, this latter being the same as the ester substituent to avoid undesired transesterification reactions, which somewhat limits the usefulness of this strategy. Upon application of these precautions, yields ranging from 62% to 91% were obtained in most cases. Nevertheless, the use of some protective groups is obviously a limitation of the method as this second step was incompatible with either base-sensitive or acid-sensitive substituents, as the desired SMAHOs are isolated after acidification to pH ≤ 2. It was thus impossible to prepare reagents bearing a phthalimide, an ester or an acetal group. This limitation is overcome by the other possibilities afforded by this methodology, as illustrated by the preparation of the cyano- (**4bn**), boron- (**4ao**), or silicon- (**4ap**) substituted SMAHOs.

**Table 2:** Preparation of SMAHOs from malonates^a^.

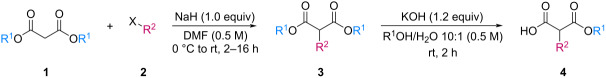							

entry	**1**	**2**	**3**	yield (%)	**4**	yield (%)	OY^b^ (%)

1	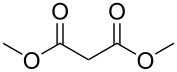 **1a**	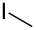 **2a**	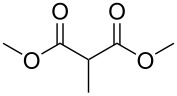 **3aa**	50	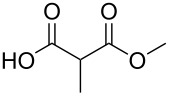 **4aa**	91	45
2	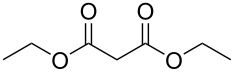 **1b**	**2a**	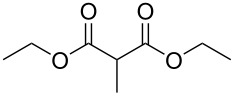 **3ba**	53 (76^c^)	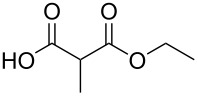 **4ba**	72	55
3	**1a**	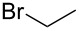 **2b**	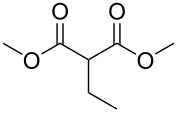 **3ab**	29 (40^c^)	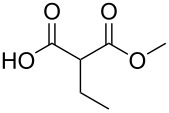 **4ab**	90	36
4	**1a**	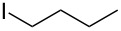 **2c**	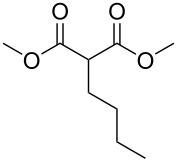 **3ac**	75	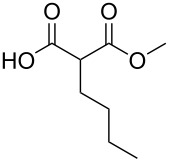 **4ac**	78	58
5	**1b**	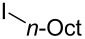 **2d**	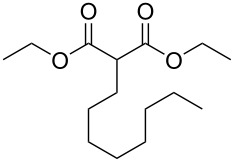 **3bd**	93	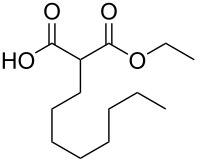 **4bd**	72	67
6	**1b**	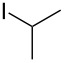 **2e**	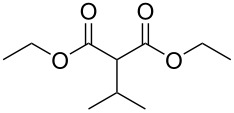 **3be**	52	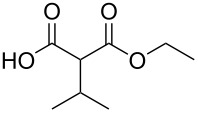 **4be**	74	38
7	**1b**	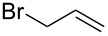 **2f**	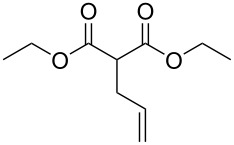 **3bf**	81	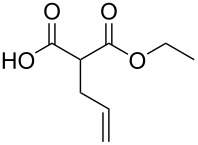 **4bf**	71	57
8	**1b**	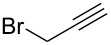 **2g**	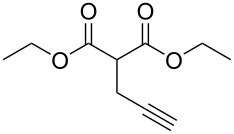 **3bg**	58	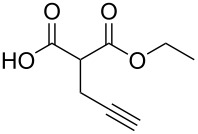 **4bg**	65	38
9	**1b**	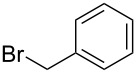 **2h**	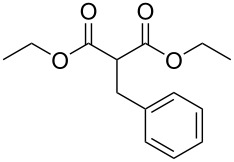 **3bh**	76	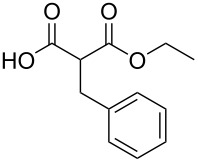 **4bh**	69	52
10	**1b** ^d^	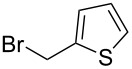 **2i**	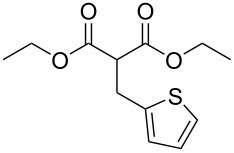 **3bi**	30^c^	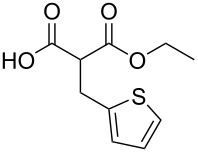 **4bi**	71	21
11	**1b**	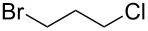 **2j**	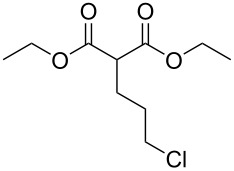 **3bj**	91^c^	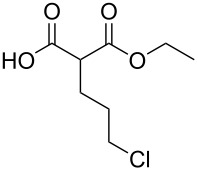 **4bj**	62	56
12	**1a**	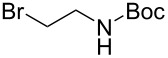 **2k**	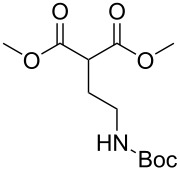 **3ak**	41^c^	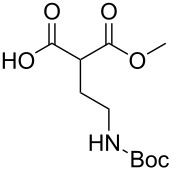 **4ak**	76	31
13	**1b** ^d^	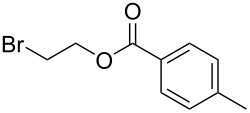 **2l**	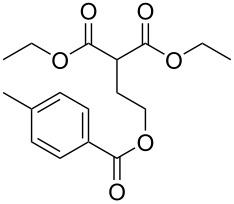 **3bl**	44^c,e^	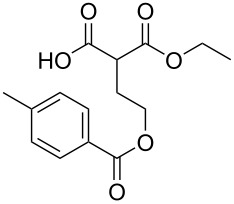 **4bl**	63	28
14	**1b**	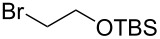 **2m**	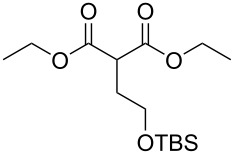 **3bm**	51^c^	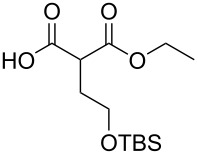 **4bm**	70	36
15	**1b**	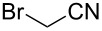 **2n**	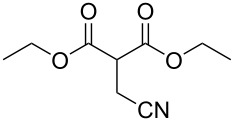 **3bn**	24^f^	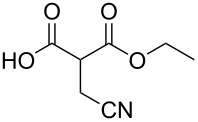 **4bn**	65	16
16	**1a**	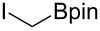 **2o**	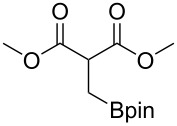 **3ao**	70	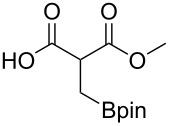 **4ao**	77	54
17	**1a**	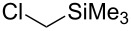 **2p**	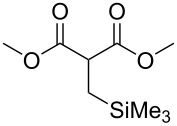 **3ap**	21^e,f,g^	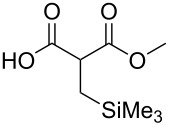 **4ap**	62	13

^a^Yields of isolated products. Step 1: **1** (1.1 equiv), **2** (5–50 mmol), NaH (1.0 equiv), DMF (*c* = 0.5–1.0 M), 0 °C to rt, 2 h. Step 2: KOH (1.0–1.2 equiv), R^1^OH/H_2_O 10:1 (*c* = 0.5 M), rt, 2 h; ^b^overall yield (2 steps); ^c^reaction time = 16 h; ^d^1.0 equiv; ^e^*T* = 80 °C; ^f^solvent = THF; reaction time = 24 h; ^g^NaI (3.0 equiv).

**Scheme 2:**
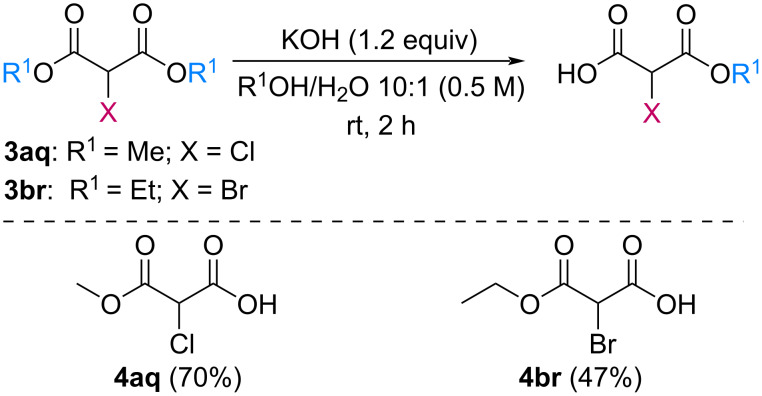
Preparation of α-halo-MAHOs.

The efficiency of the saponification step was also illustrated by the case of α-halomalonates **3aq** and **3br**. Upon application of the above-mentioned conditions, the useful reagents **4aq** and **4br** could be isolated in 70% and 47% yield, respectively (Scheme 2).

### Ester modification

#### Meldrum's acid route

**Scheme 3:**
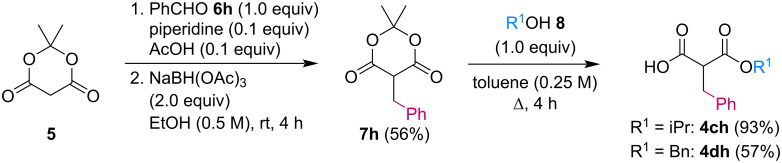
Preparation of SMAHOs from Meldrum's acid.

We next turned our attention to the diversification of the ester group of SMAHOs. Our first approach was the use of substituted malonates, protected as Meldrum's acid derivatives, as the parent compound **5** is commercially available or easily accessible on a relatively large scale (Scheme 3) [56]. Therefore, we first achieved an alkylation step of **5** using a Knoevenagel reaction with benzaldehyde, followed by an in situ reduction of the resulting alkylidene [57–58]. Even if the reaction worked well with complete conversion, the isolation of the pure product was less straightforward and required a recrystallization from a methanol/water mixture, leading to a moderate yield (56%) due to a partial solubilization of the product **7h**. This complication was yet balanced by the practicality of the second step, the opening of the Meldrum's acid moiety with an alcohol **8**. Indeed, this reaction was achieved under simple conditions and led to the desired products in useful yields, only limited by partial in situ decarboxylation. This strategy thus allowed the preparation of the isopropyl (**4ch**, 93%) and benzyl (**4dh**, 57%, corrected isolated yield) ester derivatives but remains limited to reagents bearing a benzyl substituent at the malonic position.

In an alternative strategy to circumvent the difficulties linked to the isolation of substituted Meldrum's acid derivatives, we envisioned a complementary route where the substituent was introduced prior to the protection step. Commercially available substituted malonic acids **9** were first protected with acetone under acid conditions [46], and the desired intermediates **7** were simply isolated after aqueous work-up, usually in good yields (Table 3). The opening of these latter with alcohols **8** was efficient again and allowed the preparation of new reagents in good yields, after a second aqueous work-up. Using this approach, SMAHOs bearing a benzyl (**4da**) or a (−)-menthyl group (**4ea**) were easily prepared with good overall yields (49–71%). Despite its efficiency, this second strategy required an additional step of hydrolysis of a substituted malonate to prepare the requisite substituted malonic acid.

**Table 3:** Preparation of SMAHOs from malonic acids.

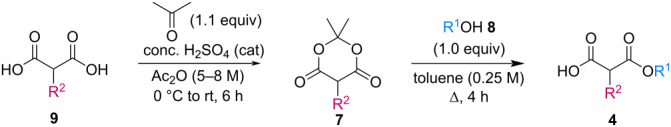					

entry	**7**	yield (%)	**4**	yield (%)	OY^a^ (%)

1	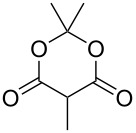 **7a**	84	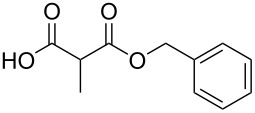 **4da**	85	71
2			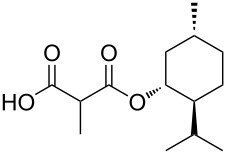 **4ea**	58	49
3	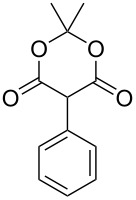 **7s**	84	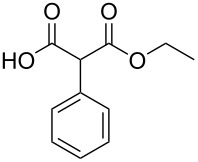 **4bs**	65^b^	55

^a^Overall yield (2 steps); ^b^in toluene/EtOH 1:1.

#### Monoesterification route

**Scheme 4:**
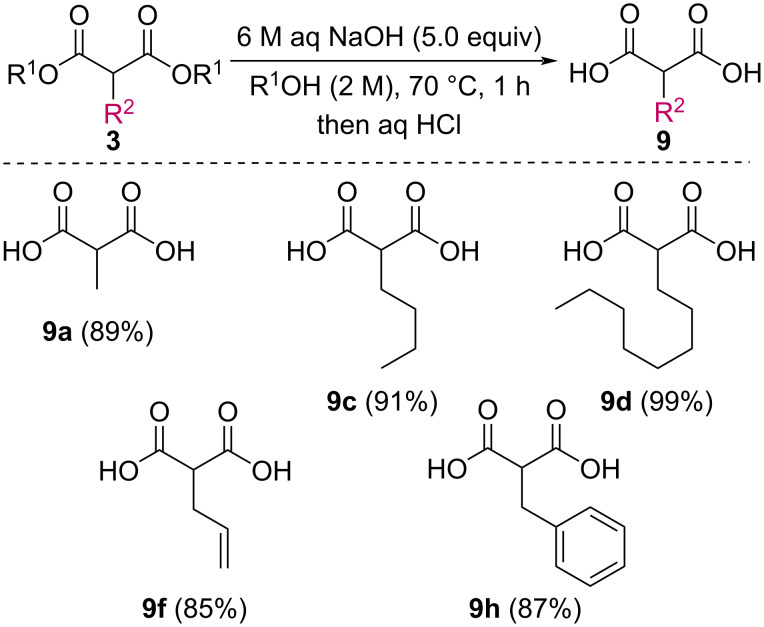
Saponification of substituted malonates.

In order to take advantage of the preparation of substituted malonates **3**, we decided to explore a more straightforward strategy, the mono-esterification of substituted malonic acids. Indeed, this approach has been only used by Fukumoto for the preparation of (−)-menthyl-derived SMAHOs [51–52]. We began this study by preparing various substituted malonic acids **9** through the saponification of the corresponding substituted malonates **3** (Scheme 4). These standard reactions were easy and delivered the expected products **9** in good to excellent yields.

We then performed a quick optimization study of the reaction between 2-methylmalonic acid (**9a**) and benzyl alcohol (**8d**) (Table 4). Starting from classical Steglich's conditions (1.0 equiv of DCC and 5 mol % of DMAP in CH_2_Cl_2_, Table 4, entry 1), the desired product was isolated in an encouraging yield of 22% after chromatography on silica gel. By adding a solution of DCC in CH_2_Cl_2_ to a solution of the other reagents in CH_3_CN (Table 4, entry 2), the yield was increased to 46%. The temperature of addition was also an important parameter and we found that an initial cooling to −15 °C was optimal (Table 4, entries 3–5), presumably by limiting the decomposition of the malonic acid derivative. We also studied the influence of the stoichiometry of the different reagents (Table 4, entries 6–9) and the use of a slight excess of alcohol **8d** was beneficial to the reaction. Finally, carrying out the reaction in only CH_3_CN allows the isolation of the desired pure compound **4da** after a simple aqueous work-up, although in a slightly lower 52% yield (Table 4, entry 10).

**Table 4:** Optimization of the mono-esterification of malonic acid **10a**.

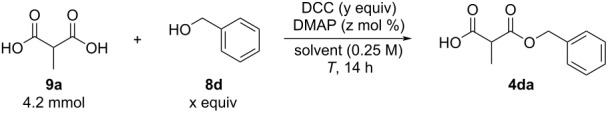						

entry	x (equiv)	y (equiv)	z (mol %)	solvent	*T*	yield (%)^a^

1	1.0	1.0	5	CH_2_Cl_2_	rt	22
2	1.0	1.0	5	CH_2_Cl_2_/CH_3_CN	rt	46
3	1.0	1.0	5	CH_2_Cl_2_/CH_3_CN	0 °C to rt	48
4	1.0	1.0	5	CH_2_Cl_2_/CH_3_CN	−15 °C to rt	59
5	1.0	1.0	5	CH_2_Cl_2_/CH_3_CN	−78 °C to rt	46
6	1.1	1.0	5	CH_2_Cl_2_/CH_3_CN	−15 °C to rt	62
7	1.5	1.0	5	CH_2_Cl_2_/CH_3_CN	−15 °C to rt	57
8	1.0	1,2	5	CH_2_Cl_2_/CH_3_CN	−15 °C to rt	53
9	1.0	1.0	20	CH_2_Cl_2_/CH_3_CN	−15 °C to rt	49
10	1.1	1.0	5	CH_3_CN	−15 °C to rt	52^b^

^a^Isolated yields after flash chromatography on silica gel; ^b^isolated yield after aqueous work-up.

**Scheme 5:**
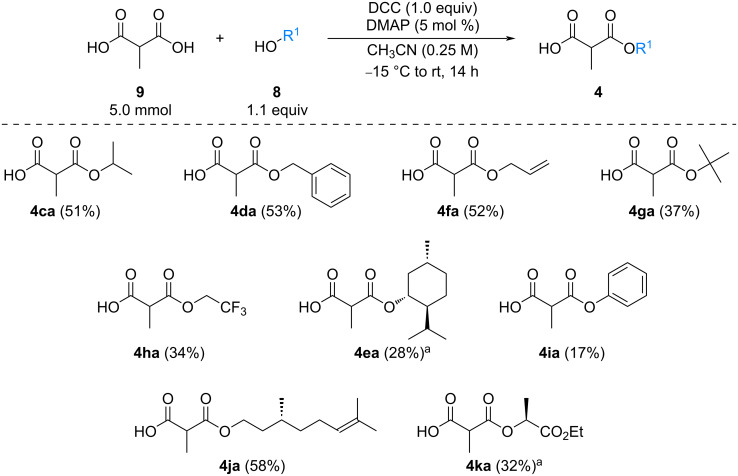
Scope of the mono-esterification of substituted malonic acids. ^a^dr = 1:1.

The scope of this reaction was next explored under such optimized conditions (Table 4, entry 10), using 2-methylmalonic acid (**9a**) as a model substrate (Scheme 5). From a general point of view, even if the yields are moderate, the reaction is easy to set up and the desired product could be isolated after a simple aqueous work-up. SMAHOs bearing classical ester groups could be obtained in moderate yields (iPr: 51%; Bn: 53%; allyl: 52%) and the use of less nucleophilic alcohols such as *t*-BuOH, 2,2,2-trifluoroethanol, (−)-menthol, and phenol led to decreased yields (37%, 34%, 28%, and 17%, respectively). More functionalized alcohols such as citronellol (**4ja**, 58%) and (−)-ethyl lactate (**4ka**, 32%) could also be employed for the direct preparation of elaborated reagents.

## Conclusion

We have prepared more than 30 SMAHOs including numerous new compounds using different strategies, thus adding a consequent input to the knowledge of these reagents. In our hands, these reagents appear to be stable to air or moisture and have been stored for several months at 4 °C. The alkylation/hydrolysis of malonates afforded a straightforward access to such derivatives but the use of an alcoholic base in the saponification step precludes the presence of sensitive functional groups and limits the degree of substitution of the ester function. This last limitation could be circumvented using the mono-esterification of malonic acid derivatives, which gives access to a broad range of derivatives. Overall, this work presents the different possibilities to access these reagents, which constitute an attractive class of pronucleophiles.

## Supporting Information

File 1 Experimental procedures, compound characterization data, and NMR spectra for all compounds.
