# Supplementary material for: Preparation of mono-substituted malonic acid half oxyesters (SMAHOs)
Source: Beilstein J Org Chem. 2021 Aug 18;17:2085–94. doi: 10.3762/bjoc.17.135 (PMC8381853; doi:10.3762/bjoc.17.135)
Supplement: File 1 — Experimental procedures, compound characterization data, and NMR spectra for all compounds. [file Beilstein_J_Org_Chem-17-2085-s001.pdf]

BEILSTEIN JOURNAL OF ORGANIC CHEMISTRY

Supporting Information

for

Preparation of mono-substituted malonic acid half oxyesters (SMAHOs)

Tania Xavier, Sylvie Condon, Christophe Pichon, Erwan Le Gall and Marc Presset

Beilstein J. Org. Chem. **2021,** *17,* 2085–2094. doi:10.3762/bjoc.17.135

Experimental procedures, compound characterization data, and NMR spectra for all compounds

License and Terms: This is a supporting information file under the terms of the Creative Commons Attribution License (https://creativecommons.org/licenses/by/4.0). Please note that the reuse, redistribution and reproduction in particular requires that the author(s) and source are credited and that individual graphics may be subject to special legal provisions.
The license is subject to the *Beilstein Journal of Organic Chemistry* terms and conditions: (https://www.beilstein-journals.org/bjoc/terms)

Table of contents

1. General information S2

2. Preparation of substituted malonates 3 .... S2

General procedure 1 (GP1) - alkylation S2

3. Preparation of substituted malonic acids 9 .... S5

General procedure 2 (GP2) - saponification S5

4. Preparation of substituted meldrum acid derivatives 7 .... S6

General procedure 3 (GP3) - preparation of meldrum acid derivatives S6

5. Preparation of SMAHOs 4 .... S6

General procedure 4 (GP4) - monosaponification S6

General procedure 5 (GP5) - monoesterification S8

General procedure 6 (GP6) - opening of meldrum acid derivatives S10

6. Copies of NMR spectra S10

1. General information

All commercially available reagents, including solvents (ethyl acetate = EA, petroleum ether = PE, cyclohexane = Cy, diethyl ether, dichloromethane, *N*,*N*-dimethylformamide = DMF, ethanol, methanol, and tetrahydrofuran = THF), were used as received. Room temperature means 18–25 °C. Melting points (mp) are uncorrected and were measured on a Büchi B-545 apparatus. Analytical thin layer chromatography (TLC) was performed on TLC silica gel plates (0.25 mm) precoated with a fluorescent indicator (Merck 60F254). Visualization was effected using ultraviolet light (λ = 254 nm) and/or a aqueous solution of $KMnO_4$. Flash chromatography (FC) was performed on 40–63 μm silica gel with mixtures of solvents. High-resolution mass spectra were obtained at the ICOA of the Université of Orléans by electrospray ionization using a Q-TOF analyzer. NMR spectra were recorded on a Bruker Avance II 400 MHz spectrometer. ^{1}H NMR chemical shifts were referenced to the residual solvent signal; ^{13}C NMR chemical shifts were referenced to the deuterated solvent signal. Multiplicity was defined by DEPT 135 analysis. Data are presented as follows: chemical shift δ (ppm), multiplicity (s = singlet, d = doublet, t = triplet, q = quartet, quint = quintet, hept = heptuplet, m = multiplet, br = broad), coupling constant *J* (Hz), integration.

2. Preparation of substituted malonates 3

General procedure 1 (GP1) - alkylation: A RBF equipped with a stirring bar was charged with NaH 60% (1.0 equiv) and purged with argon. DMF (V mL) was added and the RBF was cooled to 0 °C (ice/water bath). Malonate **1** (1.1 equiv) was added dropwise with a syringe and the mixture was stirred for 15 min. The alkyl halide **2** (1.0 equiv) was then added dropwise with a syringe. The reaction mixture was allowed to warm to room temperature and stirred for 2 h. Then, the resulting mixture was poured in sat aq NH_4Cl (3 V) and the resulting solution was extracted with Et_2O (3 × 2 V). The combined organic layers were washed with water (10 V) and brine (5 V), dried (Na_2SO_4), and evaporated. Purification by FC afforded the expected product **3**.

Compound **3aa**:[1] Following *GP1* performed with dimethyl malonate (**1a**, 1.0 mL, 8.8 mmol, 1.1 equiv), sodium hydride (0.32 g, 8.0 mmol, 1.0 equiv), and iodomethane (**2a**, 0.2 mL, 8.0 mmol, 1.0 equiv) in DMF (8 mL, *c* = 1 M) for 2 h, the desired product **3aa** was obtained after purification by FC [V(SiO_2) = 50 mL, PE/EA 95:5 (250 mL), 90:10 (250 mL)] as a colorless oil (0.591 g, 50%).
R*f* (PE/EA : 90/10, UV+KMnO₄): 0.27
^{1}H NMR (400 MHz, CDCl₃): δ 3.71 (s, 6H), 3.44 (q, *J* = 7.3 Hz, 1H), 1.40 (d, *J* = 7.3 Hz, 3H).
^{13}C{^{1}H} NMR (100 MHz, CDCl₃): δ 170.6 (2 C), 52.6 (2 CH_3), 45.9 (CH), 13.7 (CH_3).

Compound **3ba**:[2] Following *GP1* performed with diethyl malonate (**1b**, 6.7 mL, 44.0 mmol, 1.1 equiv), sodium hydride (1.92 g, 48.0 mmol, 1.2 equiv), and iodomethane (**2a**, 2.5 mL, 40.0 mmol, 1.0 equiv) in DMF (40 mL, *c* = 1 M) for 16 h, the desired product **3ba** was obtained after purification by FC [V(SiO_2) = 100 mL, PE/EA98:2 (1000 mL)] as a colorless oil (5.302 g, 76%).
R*f* (PE/EA : 90/10, UV+KMnO₄): 0.45
^{1}H NMR (400 MHz, CDCl₃): δ 4.14 (q, *J* = 7.2 Hz, 4H), 3.36 (q, *J* = 7.3 Hz, 1H), 1.35 (d, *J* = 7.3 Hz, 3H), 1.21 (t, *J* = 7.2 Hz, 6H).
^{13}C{^{1}H} NMR (100 MHz, CDCl₃): δ 170.2 (2 C), 61.3 (2 CH_2), 46.2 (CH), 14.1 (2 CH_3), 13.6 (CH_3).

Compound **3ab**:[3] Following *GP1* performed with dimethyl malonate (**1a**, 0.60 mL, 5.5 mmol, 1.1 equiv), sodium hydride (0.20 g, 5.0 mmol, 1.0 equiv), and ethyl bromide (**2b**, 0.37 mL, 5.0 mmol, 1.0 equiv) in DMF (10 mL, *c* = 0.5 M), the desired product **3ab** was obtained after purification by FC [V(SiO_2) = 50 mL, Cy/EA 95:5 (250 mL), 90:10 (250 mL)] as a colorless oil (318 mg, 40%).
R*f* (Cy/EA : 90/10, UV+KMnO₄): 0.22
^{1}H NMR (400 MHz, CDCl₃): δ 3.72 (s, 6H), 3.28 (t, *J* = 7.4 Hz, 1H), 1.92 (quint, *J* = 7.4 Hz, 2H), 0.94 (t, *J* = 7.4 Hz, 3H).
^{13}C{^{1}H} NMR (100 MHz, CDCl₃): δ 170.0 (2 C), 53.3 (CH), 52.5 (2 CH_3), 22.4 (CH_2), 12.0 (CH_3).

Compound **3ac**:[4] Following *GP1* performed with dimethyl malonate (**1a**, 0.63 mL, 5.5 mmol, 1.1 equiv), sodium hydride (0.20 g, 5.0 mmol, 1.0 equiv), and 1-iodobutane (**2c**, 0.57 mL, 5.0 mmol, 1.0 equiv) in DMF (10 mL, *c* = 0.5 M), the desired product **3ac** was obtained after purification by FC [V(SiO_2) = 50 mL, Cy/EA 95:5 (250 mL), 90:10 (250 mL)] as a colorless oil (710 mg, 75%).
R*f* (Cy/EA : 90/10, UV+KMnO₄): 0.31
^{1}H NMR (400 MHz, CDCl₃): δ 3.71 (s, 6H), 3.33 (t, *J* = 7.5 Hz, 1H), 1.87 (q, *J* = 7.5 Hz, 2H), 1.46–1.19 (m, 4H), 0.87 (t, *J* = 6.6 Hz, 3H).
^{13}C{^{1}H} NMR (100 MHz, CDCl₃): δ 170.1 (2 C), 52.5 (2 CH_3), 51.8 (CH), 29.6 (CH_2), 28.7 (CH_2), 22.4 (CH_2), 13.9 (CH_3).

---

[1] Spectral analysis matches with a commercially available sample.
[2] Xu, Q.; Cheng, B.; Ye, X.; Zhai, H. *Org. Lett.* **2009**, *11*, 4136-4138.
[3] Szostak, M.; Spain, M.; Choquette, K.A.; Flowers, R.A.; Procter, D. J. *J. Am. Chem. Soc.* **2013**, *135*, 15702-15705.
[4] Neimert-Andersson, K.; Blomberg, E.; Somfai, P. *J. Org. Chem.* **2004**, *69*, 3746-3752.

Compound **3bd**:[5] Following *GP1* performed with diethyl malonate (**1b**, 0.84 mL, 5.5 mmol, 1.1 equiv), sodium hydride (0.20 g, 5.0 mmol, 1.0 equiv), and 1-iodooctane (**2d**, 0.90 mL, 5.0 mmol, 1.0 equiv) in DMF (10 mL, c = 0.5 M), the desired product **3bd** was obtained after purification by FC [V(SiO_2) = 50 mL, Cy/EA 95:5 (250 mL), 90:10 (250 mL)] as a colorless oil (1.329 g, 93%).
R_f (PE/EA : 90/10, UV+$KMnO_4$): 0.63
^{1}H NMR (400 MHz, $CDCl_3$): δ 4.16 (q, J = 7.1 Hz, 4H), 3.28 (t, J = 7.5 Hz, 1H), 1.89–1.80 (m, 2H), 1.31–1.18 (m, 18H), 0.84 (t, J = 6.6 Hz, 3H).
^{13}C{^{1}H} NMR (100 MHz, $CDCl_3$): δ 169.7 (2 C), 61.3 (2 CH_2), 52.2 (CH), 31.9 (CH_2), 29.4 (CH_2), 29.3 (CH_2), 29.3 (CH_2), 28.8 (CH_2), 27.4 (CH_2), 22.7 (CH_2), 14.2 (3 CH_3).

Compound **3be**:[6] Following *GP1* performed with diethyl malonate (**1b**, 0.84 mL, 5.5 mmol, 1.1 equiv), sodium hydride (0.20 g, 5.0 mmol, 1.0 equiv), and 2-iodopropane (**2e**, 0.50 mL, 5.0 mmol, 1.0 equiv) in DMF (10 mL, c = 0.5 M), the desired product **3be** was obtained after purification by FC [V(SiO_2) = 50 mL, Cy/EA 95:5 (250 mL), 90:10 (250 mL)] as a colorless oil (524 mg, 52%).
R_f (Cy/EA : 90/10, UV+$KMnO_4$): 0.37
^{1}H NMR (400 MHz, $CDCl_3$): δ 4.15 (q, J = 7.1 Hz, 4H), 3.06 (d, J = 8.7 Hz, 1H), 2.41–2.28 (m, 1H), 1.22 (t, J = 7.1 Hz, 6H), 0.96 (d, J = 6.9 Hz, 6H).
^{13}C{^{1}H} NMR (100 MHz, $CDCl_3$): δ 168.9 (2 C), 61.2 (2 CH_2), 59.2 (CH), 28.8 (CH), 20.4 (2 CH_3), 14.2 (2 CH_3).

Compound **3bf**:[7] Following *GP1* performed with diethyl malonate (**1b**, 0.84 mL, 5.5 mmol, 1.1 equiv), sodium hydride (0.20 g, 5.0 mmol, 1.0 equiv), and allyl bromide (**2f**, 0.43 mL, 5.0 mmol, 1.0 equiv) in DMF (10 mL, c = 0.5 M), the desired product **3bf** was obtained after purification by FC [V(SiO_2) = 50 mL, Cy/EA 95:5 (250 mL), 90:10 (250 mL)] as a colorless oil (807 mg, 81%).
R_f (Cy/EA : 90/10, UV+$KMnO_4$): 0.29
^{1}H NMR (400 MHz, $CDCl_3$): δ 5.76 (ddt, J = 17.1, 10.3, 7.2 Hz, 1H), 5.10 (d, J = 17.1 Hz, 1H), 5.04 (d, J = 10.3 Hz, 1H), 4.18 (q, J = 7.1 Hz, 4H), 3.40 (t, J = 7.2 Hz, 1H), 2.63 (t, J = 7.2 Hz, 2H), 1.24 (t, J = 7.1 Hz, 6H).
^{13}C{^{1}H} NMR (100 MHz, $CDCl_3$): δ 169.0 (2 C), 134.2 (CH), 117.6 (CH_2), 61.5 (2 CH_2), 51.8 (CH), 32.9 (CH_2), 14.2 (2 CH_3).

Compound **3bg**:[8] Following *GP1* performed with diethyl malonate (**1b**, 0.84 mL, 5.5 mmol, 1.1 equiv), sodium hydride (0.20 g, 5.0 mmol, 1.0 equiv), and propargyl bromide (**2g**, 0.74 mL, 5.0 mmol, 1.0 equiv) in DMF (10 mL, c = 0.5 M), the desired product **3bg** was obtained after purification by FC [V(SiO_2) = 50 mL, Cy/EA 95:5 (250 mL), 90:10 (250 mL)] as a colorless oil (572 mg, 58%).
R_f (Cy/EA : 90/10, UV+$KMnO_4$): 0.34
^{1}H NMR (400 MHz, $CDCl_3$): δ 4.20 (q, J = 7.2 Hz, 4H), 3.53 (t, J = 7.5 Hz, 1H), 2.78–2.71 (m, 2H), 2.02–1.97 (m, 1H), 1.25 (t, J = 7.2 Hz, 6H).
^{13}C{^{1}H} NMR (100 MHz, $CDCl_3$): δ 167.9 (2 C), 80.0 (C), 70.5 (CH), 61.8 (2 CH_2), 51.2 (CH), 18.5 (CH_2), 14.1 (2 CH_3).

Compound **3bh**:[9] Following *GP1* performed with diethyl malonate (**1b**, 0.84 mL, 5.5 mmol, 1.1 equiv), sodium hydride (0.20 g, 5.0 mmol, 1.0 equiv), and benzyl bromide (**2h**, 0.59 mL, 5.0 mmol, 1.0 equiv) in DMF (10 mL, c = 0.5 M), the desired product **3bh** was obtained after purification by FC [V(SiO_2) = 50 mL, Cy/EA 95:5 (250 mL), 90:10 (250 mL)] as a colorless oil (959 mg, 76%).
R_f (Cy/EA : 90/10, UV+$KMnO_4$): 0.25
^{1}H NMR (400 MHz, $CDCl_3$): δ 7.33–7.27 (m, 2H), 7.26–7.21 (m, 3H), 4.19 (q, J = 7.2 Hz, 4H), 3.67 (t, J = 7.9 Hz, 1H), 3.24 (d, J = 7.9 Hz, 2H), 1.23 (t, J = 7.2 Hz, 6H).
^{13}C{^{1}H} NMR (100 MHz, $CDCl_3$): δ 169.0 (2 C), 138.0 (C), 128.9 (2 CH), 128.6 (2 CH), 126.8 (CH), 61.5 (2 CH_2), 54.0 (CH), 34.8 (CH_2), 14.1 (2 CH_3).

Compound **3bi**:[10] Following *GP1* performed with diethyl malonate (**1b**, 0.46 mL, 3.0 mmol, 1.0 equiv), sodium hydride (0.12 g, 3.0 mmol, 1.0 equiv), and 2-(bromomethyl)thiophene (**2i**, 0.53 g, 3.0 mmol, 1.0 equiv) in DMF (3 mL, c = 1 M) for 16 h, the desired product **3bi** was obtained after purification by FC [V(SiO_2) = 50 mL, Cy/EA 95:5 (500 mL)] as a yellow oil (229 mg, 30%).
R_f (Cy/EA : 80/20, UV+$KMnO_4$): 0.53
^{1}H NMR (400 MHz, $CDCl_3$): δ 7.25–7.21 (m, 1H), 7.03–7.00 (m, 1H), 6.93 (d, J = 4.9 Hz, 1H), 4.16 (q, J = 7.1 Hz, 4H), 3.63 (t, J = 7.8 Hz, 1H), 3.24 (d, J = 7.8 Hz, 2H), 1.22 (t, J = 7.1 Hz, 6H).
^{13}C{^{1}H} NMR (100 MHz, $CDCl_3$): δ 169.0 (2 C), 138.2 (C), 128.3 (CH), 125.8 (CH), 122.1 (CH), 61.6 (CH_2), 53.3 (CH_3), 29.3 (CH_2), 14.1 (CH_3).

---

[5] Ines, B.; Palomas, D.; Holle, S.; Steinberg, S.; Nicasio, J.A.; Alcarazo, M. *Angew. Chem. Int. Ed.* **2012**, *51*, 12367-12369.
[6] Cahiez, G.; Alami, M. *Tetrahedron* **1989**, *45*, 4163-4176.
[7] Yip, K.; Zhu, N.-Y.; Yang, D. *Org. Lett.* **2009**, *11*, 1911-1914.
[8] Czekelius, C.; Hafer, J.; Tonzetich, Z. J.; Schrock, R. R.; Christensen, R. L.; Mueller, P. *J. Am. Chem. Soc.* **2006**, *128*, 16664-16675.
[9] Rotthaus, O.; LeRoy, S.; Tomas, A.; Barkigia, K. M.; Artaud, I. *Eur. J. Inorg. Chem.* **2004**, 1545-1551.
[10] Che, J.; Lam, Y. *Synlett* **2010**, 2415-2420.

Compound **3bj**:[1] Following *GP1* performed with diethyl malonate (**1b**, 0.84 mL, 5.5 mmol, 1.1 equiv), sodium hydride (0.20 g, 5.0 mmol, 1.0 equiv), and 1-bromo-3-chloropropane (**2j**, 0.49 mL, 5.0 mmol, 1.0 equiv) in DMF (10 mL, *c* = 0.5 M) for 16 h, the desired product **3bj** was obtained after purification by FC [V(SiO_2) = 50 mL, Cy/EA 95:5 (250 mL), 90:10 (250 mL)] as a colorless oil (1.077 g, 91%).
R_f (PE/EA : 90/10, UV+$KMnO_4$): 0.48
^{1}H NMR (400 MHz, $CDCl_3$): δ 4.17 (q, *J* = 7.1 Hz, 4H), 3.51 (t, *J* = 6.5 Hz, 2H), 3.31 (t, *J* = 7.5 Hz, 1H), 2.01 (q, *J* = 7.5 Hz, 2H), 1.84–1.75 (m, 2H), 1.24 (t, 7.1 Hz, 6H).
^{13}C{^{1}H} NMR (100 MHz, $CDCl_3$): δ 169.1 (2 C), 61.5 (2 CH_2), 51.3 (CH), 44.2 (CH_2), 30.2 (CH_2), 26.2 (CH_2), 14.1 (2 CH_3).

Compound **3ak**:[11] Following *GP1* performed with dimethyl malonate (**1a**, 0.56 mL, 4.9 mmol, 1.1 equiv), sodium hydride (0.18 g, 4.5 mmol, 1.0 equiv), and *tert*-butyl (2-bromoethyl)carbamate (**2k**, 1.00 g, 4.5 mmol, 1.0 equiv) in DMF (10 mL, *c* = 0.5 M) for 16 h, the desired product **3ak** was obtained after purification by FC [V(SiO_2) = 50 mL, Cy/EA 90:10 (250 mL), 80:20 (250 mL)] as a colorless oil (507 mg, 41%).
R_f (Cy/EA : 80/20, UV+$KMnO_4$): 0.11
^{1}H NMR (400 MHz, $CDCl_3$): δ 4.68 (br s, 1H), 3.72 (s, 6H), 3.42 (t, *J* = 7.1 Hz, 1H), 3.19 - 3.13 (m, 2H), 2.07 (q, *J* = 6.6 Hz, 2H), 1.40 (s, 9H).
^{13}C{^{1}H} NMR (100 MHz, $CDCl_3$): δ 169.7 (2 C), 155.9 (C), 79.4 (C), 52.7 (2 CH_3), 49.2 (CH), 38.5 (CH_2), 29.1 (CH_2), 28.4 (3 CH_3).

Compound **3bl**: Following *GP1* performed with diethyl malonate (**1b**, 1.5 mL, 10.0 mmol, 1.0 equiv), sodium hydride (0.40 g, 10.0 mmol, 1.0 equiv), and 2-bromoethyl 4-methylbenzoate (**2l**, 2.431 g, 10.0 mmol, 1.0 equiv) in DMF (10 mL, *c* = 1 M) at 80 °C for 16 h, the desired product **3bl** was obtained after purification by FC [V(SiO_2) = 100 mL, PE/EA 98:2 (500 mL), 95:5 (250 mL), 90:10 (250 mL), 80/20 (250 mL)] as a colorless oil (1.425 g, 44%).
R_f (PE/EA : 90/10, UV+$KMnO_4$): 0.34
HRMS (ESI$^+$): $[M+Na]^+$ Calcd. for $C_{17}H_{22}NaO_6$: 345.1308. Found: 345.1309.
^{1}H NMR (400 MHz, $CDCl_3$): δ 7.88 (d, *J* = 7.5 Hz, 2H), 7.21 (d, *J* = 7.5 Hz, 2H), 4.35 (t, *J* = 6.1 Hz, 2H), 4.16 (t, *J* = 7.1 Hz, 4H), 3.55 (t, *J* = 7.3 Hz, 1H), 2.52–2.29 (m, 5H), 1.23 (t, *J* = 7.1 Hz, 6H).
^{13}C{^{1}H} NMR (100 MHz, $CDCl_3$): δ 169.0 (2 C), 166.4 (C), 143.8 (C), 129.7 (2 CH), 129.1 (2 CH), 127.3 (C), 62.3 (CH_2), 61.7 (2 CH_2), 49.2 (CH), 28.0 (CH_2), 21.7 (CH_3), 14.1 (2 CH_3).

Compound **3bm**: Following *GP1* performed with diethyl malonate (**1b**, 1.67 mL, 11.0 mmol, 1.1 equiv), sodium hydride (0.40 g, 10.0 mmol, 1.0 equiv), and (2-bromoethoxy)(*tert*-butyl)dimethylsilane (**2m**, 2.392 g, 10.0 mmol, 1.0 equiv) in DMF (10 mL, *c* = 1 M) for 16 h, the desired product **3bm** was obtained after purification by FC [V(SiO_2) = 50 mL, PE/EA 98:2 (250 mL), 95:5 (250 mL)] as a colorless oil (1.655 g, 51%).
R_f (PE/EA : 90/10, UV+$KMnO_4$): 0.41
HRMS (ESI$^+$): $[M+Na]^+$ Calcd. for $C_{15}H_{30}NaO_5Si$: 341.1754. Found: 341.1750.
^{1}H NMR (400 MHz, $CDCl_3$): δ 4.25–4.10 (m, 4H), 3.63 (t, *J* = 6.4 Hz, 2H), 3.56 (t, *J* = 6.4 Hz, 1H), 2.09 (q, *J* = 6.4 Hz, 2H), 1.25 (t, *J* = 7.7 Hz, 6H), 0.86 (s, 9H), 0.01 (s, 6H).
^{13}C{^{1}H} NMR (100 MHz, $CDCl_3$): δ 169.7 (2 C), 61.4 (2 CH_2), 60.4 (CH_2), 48.6 (CH), 31.8 (CH_2), 26.0 (3 CH_3), 18.4 (C), 14.2 (2 CH_3). [2 $\underline{C}H_3$ not detected]

Compound **3bn**: Following *GP1* performed with diethyl malonate (**1b**, 3.4 mL, 22.0 mmol, 1.1 equiv), sodium hydride (0.98 g, 24.0 mmol, 1.2 equiv), and bromoacetonitrile (**2n**, 1.4 mL, 20.0 mmol, 1.0 equiv) in THF (40 mL), the desired product **3bn** was obtained after purification by FC [V(SiO_2) = 100 mL, PE/EA 80:20 (500 mL), 70:30 (500 mL)] as a colorless oil (990 mg, 24%).
R_f (PE/EA : 80/20, UV+$KMnO_4$): 0.15
HRMS (ESI$^+$): $[M+H]^+$ Calcd. for $C_9H_{14}NO_4$: 200.0917. Found: 200.0916.
^{1}H NMR (400 MHz, $CDCl_3$): δ 4.29–4.20 (m, 4H), 3.68 (t, *J* = 7.3 Hz, 1H), 2.90 (d, *J* = 7.3 Hz, 2H), 1.28 (t, *J* = 7.1 Hz, 6H).
^{13}C{^{1}H} NMR (100 MHz, $CDCl_3$): δ 166.5 (2 C), 116.9 (C), 62.7 (2 CH_2), 48.1 (CH), 17.0 (CH_2), 14.0 (2 CH_3).

Compound **3ao**: Following *GP1* performed with dimethyl malonate (**1a**, 0.47 mL, 4.1 mmol, 1.1 equiv), sodium hydride (0.15 g, 3.7 mmol, 1.0 equiv), and 2-(iodomethyl)-4,4,5,5-tetramethyl-1,3,2-dioxaborolane (**2o**, 1.00 g, 3.7 mmol, 1.0 equiv) in DMF (5 mL, *c* = 1 M), the desired product **3ao** was obtained after purification by FC [V(SiO_2) = 50 mL, PE/EA 80:20 (250 mL), 60:40 (250 mL)] as a colorless oil (714 mg, 70%).
R_f (PE/EA : 80/20, UV+$KMnO_4$): 0.20
HRMS (ESI$^+$): $[M+H]^+$ Calcd. for $C_{12}H_{22}BO_6$: 273.1503. Found: 273.1506.
^{1}H NMR (400 MHz, $CDCl_3$): δ 3.67 (s, 6H), 3.55 (t, *J* = 8.0 Hz, 1H), 1.27 (d, *J* = 8.0 Hz, 2H), 1.18 (s, 12H).
^{13}C{^{1}H} NMR (100 MHz, $CDCl_3$): δ 171.0 (2 C), 83.6 (2 C), 52.5 (2 CH_2), 47.4 (CH), 24.7 (4 CH_3), 11.7 (br CH_2).

---
[11] Isaad, J.; Rolla, M.; Bianchini, R. *Eur. J. Org. Chem.* **2009**, 2748-2764.

Compound **3ap**: Following *GP1* performed with dimethyl malonate (**1a**, 0.9 mL, 7.9 mmol, 1.1 equiv), sodium hydride (0.29 g, 7.2 mmol, 1.0 equiv), (chloromethyl)trimethylsilane (**2p**, 1.0 mL, 7.2 mmol, 1.0 equiv) and sodium iodide (3.2 g, 21.5 mmol, 3.0 equiv) in THF (15 mL, *c* = 0.5 M) at reflux for 24 h, the desired product **3ap** was obtained after purification by FC [V(SiO_2) = 50 mL, Cy/EA 95:5 (250 mL), 90:10 (250 mL)] as a colorless oil (333 mg, 21%).
R*f* (PE/EA : 90/10, UV+KMnO$_4$): 0.48
HRMS (ESI$^+$): $[M+Na]^+$ Calcd. for $C_9H_{18}NaO_4Si$: 241.0866. Found: 241.0866.
^{1}H NMR (400 MHz, CDCl$_3$): δ 3.71 (s, 6H), 3.38 (t, *J* = 7.9 Hz, 1H), 1.18 (d, *J* = 7.9 Hz, 2H), 0.00 (s, 9H).
^{13}C{^{1}H} NMR (100 MHz, CDCl$_3$): δ 171.2 (2 C), 52.7 (2 CH_3), 47.6 (CH), 16.3 (CH_2), –1.54 (3 CH_3).

3. Preparation of substituted malonic acids 9

General procedure 2 (GP2) - saponification: A RBF equipped with a stirring bar was charged with malonate **3** (1.0 equiv) and MeOH (*c* = 2 M). After addition of a 6 M aqueous solution of NaOH (5.0 equiv), the reaction mixture was stirred at 70 °C for 1 h. The resulting mixture was then acidified to pH 1 with 1 M aq HCl, and extracted twice with EA (20 mL). The combined organic layers were dried (Na_2SO_4) and evaporated to afford the expected product **9**.

Compound **9a**:[12] Following *GP2* performed with **3aa** (0.40 mL, 3.0 mmol, 1.0 equiv) and NaOH (0.60 g, 15.0 mmol, 5.0 equiv) in MeOH (2.5 mL, *c* = 2 M), the desired product **9a** was obtained as an off-white solid (314 mg, 89%).
mp: 136–138 °C (lit.: 131–134°C)
^{1}H NMR (400 MHz, CD$_3$COCD$_3$): δ 10.42 (br s, 2H), 3.47 (q, *J* = 7.2 Hz, 1H), 1.35 (d, *J* = 7.2 Hz, 3H).
^{13}C{^{1}H} NMR (100 MHz, CD$_3$COCD$_3$): δ 171.8 (2 C), 46.1 (CH), 14.0 (CH_3).

Compound **9c**:[1] Following *GP2* performed with **3ac** (565 mg, 3.0 mmol, 1.0 equiv) and NaOH (0.60 g, 15.0 mmol, 5.0 equiv) in MeOH (2.5 mL, *c* = 2 M), the desired product **9c** was obtained as a white solid (439 mg, 91%).
mp: 101–103 °C (lit.: 102–103°C)
^{1}H NMR (400 MHz, CD$_3$SOCD$_3$): δ 12.62 (br s, 2H), 3.17 (t, *J* = 7.4 Hz, 1H), 1.69 (q, *J* = 7.4 Hz, 2H), 1.36–1.14 (m, 4H), 0.84 (t, *J* = 6.8 Hz, 3H).
^{13}C{^{1}H} NMR (100 MHz, CD$_3$SOCD$_3$): δ 171.1 (2 C), 51.7 (CH), 29.2 (CH_2), 28.3 (CH_2), 22.1 (CH_2), 13.9 (CH_3).

Compound **9d**:[13] Following *GP2* performed with **3bd** (817 mg, 3.0 mmol, 1.0 equiv) and NaOH (0.60 g, 15.0 mmol, 5.0 equiv) in MeOH (2.5 mL, *c* = 2 M), the desired product **9d** was obtained as a white solid (647 mg, quantitative).
mp: 110–113 °C (lit.: 113–114°C)
^{1}H NMR (400 MHz, CD$_3$SOCD$_3$): δ 12.64 (br s, 2H), 3.17 (t, *J* = 7.4 Hz, 1H), 1.68 (q, *J* = 7.4 Hz, 2H), 1.28–1.19 (m, 12H), 0.85 (t, *J* = 6.2 Hz, 3H).
^{13}C{^{1}H} NMR (100 MHz, CD$_3$SOCD$_3$): δ 171.0 (2 C), 51.7 (CH), 31.3 (CH_2), 28.9 (CH_2), 28.8 (CH_2), 28.7 (CH_2), 28.5 (CH_2), 26.9 (CH_2), 22.2 (CH_2), 14.0 (CH_3).

Compound **9f**:[14] Following *GP2* performed with **3bf** (100 mg, 0.5 mmol, 1.0 equiv) and NaOH (0.10 g, 2.5 mmol, 5.0 equiv) in MeOH (0.4 mL, *c* = 2 M), the desired product **9f** was obtained as a white solid (61 mg, 85%).
mp: 101-104 °C (lit.: 102–105°C)
^{1}H NMR (400 MHz, CD$_3$COCD$_3$): δ 9.90 (br s, 2H), 5.91–5.75 (m, 1H), 5.12 (dd, *J* = 17.1, 3.0 Hz, 1H), 5.02 (dd, *J* = 10.3, 3.0 Hz, 1H), 3.46 (t, *J* = 7.5 Hz, 1H), 2.59 (t, *J* = 7.5 Hz, 2H).
^{13}C{^{1}H} NMR (100 MHz, CD$_3$COCD$_3$): δ 170.5 (2 C), 135.7 (CH), 117.4 (CH_2), 51.8 (CH), 33.6 (CH_2).

Compound **9h**:[15] Following *GP2* performed with **3bh** (222 mg, 0.89 mmol, 1.0 equiv) and NaOH (0.18 g, 5.0 mmol, 5.0 equiv) in MeOH (0.8 mL, *c* = 2 M), the desired product **9h** was obtained as an off-white solid (151 mg, 87%).
^{1}H NMR (400 MHz, CD$_3$COCD$_3$): δ 9.78 (br s, 2H), 7.34–7.24 (m, 4H), 7.24–7.17 (m, 1H), 3.73 (t, *J* = 7.8 Hz, 1H), 3.19 (d, *J* = 7.8 Hz, 2H).
^{13}C{^{1}H} NMR (100 MHz, CD$_3$COCD$_3$): δ 170.4 (2 C), 139.5 (C), 129.7 (2 CH), 129.2 (2 CH), 127.3 (CH), 54.0 (CH), 35.4 (CH_2).

---

[12] McErlan, M. *Org. Biomol. Chem.* **2016**, *14*, 1236-1238.
[13] Jayaraman, A.; Mahanthappa, M.K. *Langmuir*, **2018**, *34*, 2290-2301.
[14] Sun, X.G.; Reeder, C. L.; Kerr, J. B. *Macromolecules*, **2004**, *37*, 2219-2227.
[15] Lai, Y.; Sun, L.; Sit, M.K.; Wang, Y.; Dai, W.M. *Tetrahedron*, **2016**, *72*, 664-673.

4. Preparation of substituted meldrum acid derivatives 7

Compound **7h**:[16] A flame-dried 100 mL RBF equipped with a stirring bar was charged with meldrum acid (**5**, 1.44 g, 10.0 mmol, 1.0 equiv), closed with a septum, and purged with argon. After the addition of benzaldehyde (**6h**, 1.00 mL, 10.0 mmol, 1.0 equiv) with a syringe, and abs. EtOH (20 mL, *c* = 0.5 M) by quick removal of the septum, the mixture was stirred (by heating with a heat gun, if necessary) until complete solubilization of the solid. Then, piperidine (99 µL, 1.0 mmol, 10 mol %) and acetic acid (57 µL, 1.0 mmol, 10 mol %) were added through the septum with syringes, and the reaction was stirred at rt for 30 min. $NaBH(OAc)_3$ (4.24 g, 20.0 mmol, 2.0 equiv) was added in 4 equal portions, one after the other on a 30 min interval by temporary removal of the septum and the reaction was stirred for 60 min at rt. Then, the resulting mixture was quenched with a sat. aq. NH_4Cl (50 mL) and extracted with DCM (3 × 20 mL). The combined organic layers were dried over Na_2SO_4 and evaporated. Purification by recrystallization ($MeOH/H_2O$) afforded the expected product **7h** as an off-white solid (1.321 g, 56%).
^{1}H NMR (400 MHz, $CDCl_3$): δ 7.39–7.22 (m, 5H), 3.79 (t, *J* = 4.7 Hz, 1H), 3.52 (d, *J* = 4.7 Hz, 2H), 1.76 (s, 3H), 1.51 (s, 3H).
^{13}C{^{1}H} NMR (100 MHz, $CDCl_3$): δ 165.4 (2 C), 137.4 (C), 129.9 (2 CH), 128.8 (2 CH), 127.3 (CH), 105.4 (C), 48.3 (CH), 32.3 (CH_2), 28.6 (CH_3), 27.4 (CH_3).

General procedure 3 (GP3) - preparation of meldrum acid derivatives: A RBF equipped with a stirring bar was charged with the malonic acid derivative **9** (1.0 equiv) and Ac_2O (5–8 M), and cooled to 0 °C (ice/water bath). After the addition of 2–3 drops of concentrated H_2SO_4, acetone (1.1 equiv) was added dropwise to the stirred mixture with a pressure-equalizing dropping funnel. The reaction was allowed to warm to room temperature and stirred for 6 h. Then, the resulting mixture was placed in the refrigerator for 2 h, and the resulting solid was collected by filtration on a fritted funnel and rinsed with cold water (2 times) and Et_2O (3 times). Traces of water were removed by addition of toluene (10 mL) and evaporation (3 times) to afford the desired product **7**.

Compound **7a**:[17] Following *GP3* performed with methylmalonic acid **9a** (2.95 g, 25.0 mmol, 1.5 equiv) and acetone (2.0 mL, 27.5 mmol, 1.1 equiv) in Ac_2O (3.2 mL, *c* = 8.0 M), the desired product **7a** was obtained as a white solid (3.354 g, 84%).
mp: 114–116 °C (lit.: 113–114 °C)
^{1}H NMR (400 MHz, $CDCl_3$): δ 3.61 (q, *J* = 7.0 Hz, 1H), 1.80 (s, 3H), 1.74 (s, 3H), 1.54 (d, *J* = 7.0 Hz, 3H).
^{13}C{^{1}H} NMR (100 MHz, $CDCl_3$): δ 166.2 (2 C), 105.0 (C), 41.4 (CH), 28.6 (CH_3), 26.5 (CH_3), 10.8 (CH_3).

Compound **7s**:[18] Following *GP3* performed with phenylmalonic acid **9s** (4.50 g, 25.0 mmol, 1.5 equiv) and acetone (2.0 mL, 27.5 mmol, 1.1 equiv) in Ac_2O (5.0 mL, *c* = 5.0 M), the desired product **7s** was obtained as a pale yellow solid (4.690 g, 84%).
mp: 135–138 °C (lit.: 135–137°C)
^{1}H NMR (400 MHz, $CDCl_3$): δ 7.50–7.39 (m, 3H), 7.37–7.24 (m, 2H), 4.82 (s, 1H), 1.88 (s, 3H), 1.78 (s, 3H).
^{13}C{^{1}H} NMR (100 MHz, $CDCl_3$): δ 164.9 (2 C), 130.7 (C), 129.3 (2 CH), 129.2 (2 CH), 128.9 (CH), 105.9 (C), 52.9 (CH), 28.6 (CH_3), 27.5 (CH_3).

5. Preparation of SMAHOs 4

General procedure 4 (GP4) - monosaponification: In a manner similar to [19], a RBF equipped with a stirring bar was charged with the malonate derivative **3** (1.0 equiv) and the solvent (*c* = 0.5 M, V mL). A 5 M aqueous solution of KOH (1.0 equiv) was added with a syringe and the reaction mixture was stirred at rt for 2 h. Then, the mixture was concentrated, diluted with water (V mL), and the resulting aqueous layer was washed with Et_2O (3*V/4 mL), acidified to pH 1 by the addition of 1 M aq HCl, saturated with NaCl, and extracted with EA (3*V/4 mL). The combined organic layers were dried (Na_2SO_4) and evaporated to afford the expected product **4**.

Compound **4aa**:[20] Following *GP4* performed with **3aa** (6.70 mL, 50.0 mmol, 1.0 equiv) and potassium hydroxide (3.37 g, 60.0 mmol, 1.2 equiv) in MeOH (100 mL), the desired product **4aa** was obtained as a colorless oil (6.011 g, 91%).
Characterization date are in agreement with those reported in [19].

Compound **4ba**:[20] Following *GP4* performed with **3ba** (3.00 g, 17.2 mmol, 1.0 equiv) and potassium hydroxide (0.96 g, 17.2 mmol, 1.0 equiv) in EtOH (34 mL), the desired product **4ba** was obtained as a colorless oil (1.818 g, 72%).
^{1}H NMR (400 MHz, $CDCl_3$): δ 10.15 (br s, 1H), 4.18 (q, *J* = 7.1 Hz, 2H), 3.45 (q, *J* = 7.4 Hz, 1H), 1.41 (d, *J* = 7.4 Hz, 3H), 1.25 (t, *J* = 7.1 Hz, 3H).
^{13}C{^{1}H} NMR (100 MHz, $CDCl_3$): δ 175.9 (C), 170.1 (C), 61.9 (CH_2), 46.1 (CH), 14.0 (CH_3), 13.6 (CH_3).

[16] Fillion, E.; Wilsily, A.; Fishlock, D. *J. Org. Chem.* **2009**, *74*, 1259-1267.
[17] Nikolaev, V. A.; Shevchenko, V. V.; Platz, M. S.; Khimich, N. N. *Russ. J. Org. Chem*. **2006**, *42*, 815-827.
[18] Szostak, M.; Lyons, S. E.; Spain, M.; Procter, D. J. *Chem. Commun.* **2014**, *50*, 8391-8394.
[19] Xavier, T. Condon, S.; Pichon, C.; Le Gall, E.; Presset, M. *J. Org. Chem.*, **2021**, *86*, 5452–5462.
[20] Niwayama, S.; Cho, H.; Lin, C. *Tetrahedron Lett.* **2008**, *49*, 4434-4436.

Compound **4ab**:[21] Following *GP4* performed with **3ab** (1.28 g, 8.0 mmol, 1.0 equiv) and potassium hydroxide (0.54 g, 10.0 mmol, 1.2 equiv) in MeOH (15 mL), the desired product **4ab** was obtained as a colorless oil (1.052 g, 90%).
^{1}H NMR (400 MHz, CDCl$_3$): δ 10.62 (br s, 1H), 3.75 (s, 3H), 3.32 (t, *J* = 7.4 Hz, 1H), 1.95 (quint, *J* = 7.4 Hz, 2H), 0.98 (t, *J* = 7.4 Hz, 3H).
^{13}C{^{1}H} NMR (100 MHz, CDCl$_3$): δ 175.4 (C), 169.8 (C), 53.2 (CH), 52.8 (CH_3), 22.4 (CH_2), 11.9 (CH_3).

Compound **4ac**:[22] Following *GP4* performed with **3ac** (1.02 g, 6.4 mmol, 1.0 equiv) and potassium hydroxide (0.43 g, 7.7 mmol, 1.2 equiv) in MeOH (11 mL), the desired product **4ac** was obtained as a colorless oil (0.871 g, 78%).
Characterization date are in agreement with those reported in 19.

Compound **4bd**:[23] Following *GP4* performed with **3bd** (5.50 g, 20.2 mmol, 1.0 equiv) and potassium hydroxide (1.13 g, 20.2 mmol, 1.0 equiv) in EtOH (40 mL), the desired product **4bd** was obtained as a colorless oil (3.560 g, 72%).
Characterization date are in agreement with those reported in 19.

Compound **4be**: Following *GP4* performed with **3be** (2.70 g, 13.3 mmol, 1.0 equiv) and potassium hydroxide (0.75 g, 13.3 mmol, 1.0 equiv) in EtOH (27 mL), the desired product **4be** was obtained as a colorless oil (1.716 g, 74%).
HRMS (ESI$^+$): m/z $[M+Na]^+$ Calcd. for $C_8H_{14}NaO_4$: 197.0784. Found: 197.0783.
^{1}H NMR (400 MHz, CDCl$_3$): δ 10.76 (br s, 1H), 4.19 (q, *J* = 7.1 Hz, 2H), 3.15 (d, *J* = 8.3 Hz, 1H), 2.37 (m, 1H), 1.26 (t, *J* = 7.1 Hz, 3H), 1.01 (t, *J* = 7.4 Hz, 6H).
^{13}C{^{1}H} NMR (100 MHz, CDCl$_3$): δ 174.6 (C), 169.1 (C), 61.7 (CH_2), 58.7 (CH), 29.1 (CH), 20.4 (CH_3), 20.3 (CH_3), 14.1 (CH_3).

Compound **4bf**: Following *GP4* performed with **3bf** (0.98 g, 4.9 mmol, 1.0 equiv) and potassium hydroxide (0.27 g, 4.9 mmol, 1.0 equiv) in EtOH (10 mL), the desired product **4bf** was obtained as a colorless oil (0.603 g, 71%).
Characterization date are in agreement with those reported in 19.

Compound **4bg**:[24] Following *GP4* performed with **3bg** (1.70 g, 8.6 mmol, 1.0 equiv) and potassium hydroxide (0.48 g, 8.6 mmol, 1.0 equiv) in EtOH (18 mL), the desired product **4bg** was obtained as a colorless oil (0.963 g, 65%).
Characterization date are in agreement with those reported in 19.

Compound **4bh**: Following *GP4* performed with **3bh** (1.07 g, 4.3 mmol, 1.0 equiv) and potassium hydroxide (0.24 g, 4.3 mmol, 1.0 equiv) in EtOH (10 mL), the desired product **4bh** was obtained as a colorless oil (0.657 g, 69%).
Characterization date are in agreement with those reported in 19.

Compound **4bi**:[25] Following *GP4* performed with **3bi** (0.19 g, 0.73 mmol, 1.0 equiv) and potassium hydroxide (49 mg, 0.88 mmol, 1.2 equiv) in EtOH (2 mL), the desired product **4bi** was obtained as a colorless oil (0.118 g, 71%).
^{1}H NMR (400 MHz, CDCl$_3$): δ 9.03 (br s, 1H), 7.40–7.19 (m, 1H), 7.06 (m, 1H), 6.97 (d, *J* = 4.9 Hz, 1H), 4.21 (q, *J* = 7.2 Hz, 2H), 3.73 (t, *J* = 7.6 Hz, 1H), 3.29 (d, *J* = 7.6 Hz, 2H), 1.24 (t, *J* = 7.2 Hz, 3H).
^{13}C{^{1}H} NMR (100 MHz, CDCl$_3$): δ 174.1 (C), 168.8 (C), 137.7 (C), 128.1 (CH), 125.9 (CH), 122.2 (CH), 62.0 (CH_2), 53.0 (CH), 29.2 (CH_2), 14.0 (CH_3).

Compound **4bj**: Following *GP4* performed with **3bj** (1.62 g, 6.8 mmol, 1.0 equiv) and potassium hydroxide (0.38 g, 6.8 mmol, 1.0 equiv) in EtOH (14 mL), the desired product **4bj** was obtained as a colorless oil (0.890 g, 62%).
HRMS (ESI$^+$): m/z $[M+Na]^+$ Calcd. for $C_8H_{13}NaO_4$: 231.0394. Found: 231.0391.
^{1}H NMR (400 MHz, CDCl$_3$): δ 10.05 (br s, 1H), 4.22 (q, *J* = 7.1 Hz, 2H), 3.54 (t, *J* = 6.4 Hz, 2H), 3.40 (t, *J* = 7.4 Hz, 1H), 2.11–2.02 (m, 2H), 1.89–1.79 (m, 2H), 1.27 (t, *J* = 7.1 Hz, 3H).
^{13}C{^{1}H} NMR (100 MHz, CDCl$_3$): δ 174.8 (C), 169.0 (C), 62.0 (CH_2), 51.1 (CH), 44.2 (CH_2), 30.1 (CH_2), 26.2 (CH_2), 14.1 (CH_3).

Compound **4ak**: Following *GP4* performed with **3ak** (0.50 g, 1.8 mmol, 1.0 equiv) and potassium hydroxide (0.10 g, 1.8 mmol, 1.0 equiv) in MeOH (5 mL), the desired product **4ak** was obtained as a colorless oil (0.366 g, 76%).

---

[21] Krumme, D.; Tschesche, H. *Tetrahedron* **1999**, *55*, 3007-3018.
[22] Nakagawa, A.; Ohno, H.; Miyano, K.; Omura, S. *J. Org. Chem.* **1980**, *45*, 3268-3274.
[23] Volonterio, A.; Zanda, M. *J. Org. Chem.* **2008**, *73*, 7486-7497.
[24] Aleman, J.; del Solar, V.; Martin-Santos, C.; Cubo, L.; Ranninger, C. N. *J. Org. Chem.* **2011**, *76*, 7287-7293.
[25] Keenan, R. M.; Weinstock, J.; Finkelstein, J. A.; Franz, R. G.; Gaitanopoulos, D. E.; Girard, G. R.; Hill, D. T.; Morgan, T. M.; Samanen, J. M.; Peishoff, C. E.; Tucker, L. M.; Aiyar, N.; Griffin, E.; Ohlstein, E. H.; Stack, E. J.; Weidley, E. F.; Edwards, R. M. *J. Med. Chem.* **1993**, *36*, 1880-1892.

HRMS (ESI$^+$): $[M+K]^+$ Calcd. for $C_{11}H_{19}KNO_6$: 300.0843. Found: 300.0843.
^{1}H NMR (400 MHz, CD_3COCD_3,): δ 6.09 (br s, 1H), 3.71 (s, 3H), 3.48 (t, *J* = 7.2 Hz, 1H), 3.16 (q, *J* = 6.4 Hz, 2H), 2.10–2.06 (m, 2H), 1.40 (s, 9H). [$CO_2\underline{H}$ not detected]
^{13}C{^{1}H} NMR (100 MHz, CD_3COCD_3,): δ 170.5 (C), 170.4 (C), 156.7 (C), 78.6 (C), 52.4 (CH_3), 49.5 (CH), 38.9 (CH_2), 30.5 (CH_2), 28.5 (3 CH_3).

Compound **4bl**: Following *GP4* performed with **3bl** (1.40 g, 4.4 mmol, 1.0 equiv) and potassium hydroxide (0.30 g, 5.3 mmol, 1.2 equiv) in EtOH (9 mL), the desired product **4bl** was obtained as a colorless oil (0.825 g, 63%).
Characterization date are in agreement with those reported in 19.

Compound **4bm**: Following *GP4* performed with **3bm** (0.98 g, 3.1 mmol, 1.0 equiv) and potassium hydroxide (0.21 g, 3.7 mmol, 1.2 equiv) in EtOH (6 mL), the desired product **4bm** was obtained as a colorless oil (0.633 g, 70%).
HRMS (ESI$^+$): $[M+Na]^+$ Calcd. for $C_{13}H_{26}NaO_5Si$: 313.1442. Found: 313.1444.
^{1}H NMR (400 MHz, $CDCl_3$): δ 9.30 (br s, 1H), 4.20 (q, *J* = 7.0 Hz, 2H), 3.75–3.61 (m, 2H), 3.61 (t, *J* = 6.6 Hz, 1H), 2.13 (q, *J* = 6.0 Hz, 2H), 1.27 (t, *J* = 7.0 Hz, 3H), 0.87 (s, 9H), 0.02 (s, 6H).
^{13}C{^{1}H} NMR (100 MHz, $CDCl_3$): δ 175.1 (C), 169.5 (C), 61.8 (CH_2), 60.4 (CH_2), 48.6 (CH), 31.7 (CH_2), 26.0 (3 CH_3), 18.4 (C), 14.1 (CH_3). [2 $\underline{C}H_3$ not detected]

Compound **4bn**: Following *GP4* performed with **3bn** (0.94 g, 4.7 mmol, 1.0 equiv) and potassium hydroxide (0.26 g, 4.7 mmol, 1.0 equiv) in EtOH (20 mL), the desired product **4bn** was obtained as a colorless oil (0.528 g, 65%).
HRMS (ESI$^+$): $[M+Na]^+$ Calcd. for $C_7H_9NNaO_4$: 194.0424. Found: 194.0428.
^{1}H NMR (400 MHz, $CDCl_3$): δ 9.31 (br s, 1H), 4.30 (q, *J* = 7.1 Hz, 2H), 3.78 (t, *J* = 7.3 Hz, 1H), 2.94 (d, *J* = 7.3 Hz, 2H), 1.32 (t, *J* = 7.1 Hz, 3H).
^{13}C{^{1}H} NMR (100 MHz, $CDCl_3$): δ 170.8 (C), 166.5 (C), 116.8 (C), 63.2 (CH_2), 47.9 (CH), 17.0 (CH_2), 14.0 (CH_3).

Compound **4ao**: Following *GP4* performed with **3ao** (0.41 g, 1.5 mmol, 1.0 equiv) and potassium hydroxide (0.10 g, 1.8 mmol, 1.2 equiv) in MeOH (3 mL), the desired product **4ao** was obtained as a colorless oil (0.300 g, 77%).
HRMS (ESI$^+$): $[M+Na]^+$ Calcd. for $C_{11}H_{19}BNaO_6$: 281.1167. Found: 281.1165.
^{1}H NMR (400 MHz, $CDCl_3$): δ 7.62 (br s, 1H), 3.72 (s, 3H), 3.61 (t, *J* = 7.9 Hz, 1H), 1.32 (d, *J* = 7.9 Hz, 2H), 1.20 (s, 12H).
^{13}C{^{1}H} NMR (100 MHz, $CDCl_3$): δ 175.5 (C), 170.9 (C), 83.9 (2 C), 52.7 (CH_3), 47.4 (CH), 24.7 (4 CH_3), 11.5 (CH_2).

Compound **4ap**: Following *GP4* performed with **3ap** (92 mg, 0.57 mmol, 1.0 equiv) and potassium hydroxide (39 mg, 0.69 mmol, 1.2 equiv) in MeOH (2 mL), the desired product **4ap** was obtained as a colorless oil (73 mg, 62%).
HRMS (ESI$^+$): $[M+Na]^+$ Calcd. for $C_8H_{16}NaO_4Si$: 227.0710. Found: 227.0713.
^{1}H NMR (400 MHz, $CDCl_3$): δ 10.16 (br s, 1H), 3.73 (s, 3H), 3.40 (t, *J* = 7.8 Hz, 1H), 1.18 (t, *J* = 7.8 Hz, 2H), 0.01 (s, 9H).
^{13}C{^{1}H} NMR (100 MHz, $CDCl_3$): δ 176.7 (C), 170.8 (C), 52.8 (CH_3), 47.6 (CH), 16.2 (CH_2), –1.54 (3 CH_3).

Compound **4aq**: Following *GP4* performed with dimethyl chloromalonate **3aq** (2.5 mL, 20.0 mmol, 1.0 equiv) and potassium hydroxide (1.12 g, 20.0 mmol, 1.0 equiv) in MeOH (40 mL), the desired product **4aq** was obtained as a colorless oil (2.160 g, 70%).
HRMS (ESI$^+$): m/z $[M+Na]^+$ Calcd. for $C_4H_5NaO_4$: 174.9768. Found: 174.9768.
^{1}H NMR (400 MHz, $CDCl_3$): δ 11.17 (br s, 1H), 4.95 (s, 1H), 3.86 (s, 3H).
^{13}C{^{1}H} NMR (100 MHz, $CDCl_3$): δ 169.6 (C), 164.9 (C), 54.9 (CH), 54.3 (CH_3).

Compound **4br**: Following *GP4* performed with diethyl bromomalonate **3br** (1.7 mL, 10.0 mmol, 1.0 equiv) and potassium hydroxide (0.56 g, 10.0 mmol, 1.0 equiv) in EtOH (20 mL), the desired product **4br** was obtained as a colorless oil (1.001 g, 47%).
HRMS (ESI$^+$): m/z $[M+Na]^+$ Calcd. for $C_5H_7BrNaO_4$: 232.9419. Found: 232.9418.
^{1}H NMR (400 MHz, $CDCl_3$): δ 10.66 (br s, 1H), 4.91 (s, 1H), 4.34 (q, *J* = 7.1 Hz, 2H), 1.35 (t, *J* = 7.1 Hz, 3H).
^{13}C{^{1}H} NMR (100 MHz, $CDCl_3$): δ 169.8 (C), 164.7 (C), 63.9 (CH_2), 41.4 (CH), 13.9 (CH_3).

General procedure 5 (GP5) - monoesterification: A 50 mL RBF equipped with a stirring bar was charged with the methyl malonic acid (**9a**, 0.50 g, 4.2 mmol, 1.0 equiv), DMAP (26 mg, 0.21 mmol, 5 mol %), CH_3CN (10 mL), and the requisite alcohol **8** (1.1 equiv) and cooled at −15 °C (ice/salt bath). A solution of DCC (0.87 g, 4.2 mmol, 1.0 equiv) in CH_3CN (10 mL) was added dropwise to the stirred mixture with a pressure-equalizing dropping funnel. The reaction was allowed to warm to rt and stirred for 14 h. Then, the reaction mixture was filtered through a fritted funnel (rinsed with CH_2Cl_2), and the filtrate was evaporated. To the resulting crude oil were added 30 mL of sat aq $NaHCO_3$. The resulting solution was washed with Et_2O (2 × 10 mL), acidified to pH 1 with 1 M aq HCl, and extracted with CH_2Cl_2 (2 × 20 mL). The combined organic layers were dried (Na_2SO_4) and evaporated to afford the expected product **4**.

Compound **4ca**: Following *GP5* performed with isopropanol (**8c**, 0.35 mL, 4.6 mmol, 1.1 equiv), the desired product **4ca** was obtained as a colorless oil (0.341 g, 51%).
HRMS (ESI$^+$): $[M+Na]^+$ Calcd. for $C_7H_{12}NaO_4$: 183.0627. Found: 183.0633.
^{1}H NMR (400 MHz, CDCl$_3$): δ 9.80 (br s, 1H), 5.03 (hept, *J* = 6.5 Hz, 1H), 3.42 (q, *J* = 7.3 Hz, 1H), 1.40 (d, *J* = 7.3 Hz, 3H), 1.22 (d, *J* = 6.5 Hz, 6H).
^{13}C{^{1}H} NMR (100 MHz, CDCl$_3$): δ 175.9 (C), 169.7 (C), 69.5 (CH), 46.2 (CH), 21.6 (CH_3), 21.5 (CH_3), 13.5 (CH_3).

Compound **4da**: Following *GP5* performed with benzyl alcohol (**8d**, 0.48 mL, 4.6 mmol, 1.1 equiv), the desired product **4da** was obtained as a colorless oil (0.462 g, 53%).
Characterization date are in agreement with those reported in 19.

Compound **4fa**: Following *GP5* performed with allyl alcohol (**8f**, 0.31 mL, 4.6 mmol, 1.1 equiv), the desired product **4fa** was obtained as a colorless oil (0.345 g, 52%).
HRMS (ESI$^+$): $[M+Na]^+$ Calcd. for $C_7H_{10}NaO_4$: 181.0471. Found: 181.0477.
^{1}H NMR (400 MHz, CDCl$_3$): δ 11.01 (br s, 1H), 5.89 (ddt, *J* = 16.2, 10.6, 5.7 Hz, 1H), 5.31 (dt, *J* = 16.2, 1.6 Hz, 1H), 5.27–5.18 (m, 1H), 4.63 (d, *J* = 5.7 Hz, 2H), 3.51 (q, *J* = 7.3 Hz, 1H), 1.44 (d, *J* = 7.3 Hz, 3H).
^{13}C{^{1}H} NMR (100 MHz, CDCl$_3$): δ 176.1 (C), 169.6 (C), 131.5 (CH), 118.8 (CH_2), 66.3 (CH_2), 46.04 (CH), 13.6 (CH_3).

Compound **4ga**:[26] Following *GP5* performed with *tert*-butanol **8g** (0.44 mL, 4.6 mmol, 1.1 equiv), the desired product **4ga** was obtained as a colorless oil (0.271 g, 37%).
^{1}H NMR (400 MHz, CDCl$_3$): δ 10.90 (br s, 1H), 3.37 (q, *J* = 7.3 Hz, 1H), 1.44 (s, 9H), 1.38 (d, *J* = 7.3 Hz, 3H).
^{13}C{^{1}H} NMR (100 MHz, CDCl$_3$): δ 176.5 (C), 169.3 (C), 82.4 (C), 47.0 (CH), 27.9 (3 CH_3), 13.7 (CH_3).

Compound **4ha**: Following *GP5* performed with 2,2,2-trifluoroethanol (**8h**, 0.33 mL, 4.6 mmol, 1.1 equiv), the desired product **4ha** was obtained as a colorless oil (0.285 g, 34%).
HRMS (ESI$^+$): $[M+Na]^+$ Calcd. for $C_6H_7F_3NaO_4$: 223.0188. Found: 223.0189.
^{1}H NMR (400 MHz, CDCl$_3$): δ 10.51 (br s, 1H), 4.59–4.49 (m, 2H), 3.62 (q, *J* = 7.2 Hz, 1H), 1.51 (d, *J* = 7.2 Hz, 3H).
^{13}C{^{1}H} NMR (100 MHz, CDCl$_3$): δ 175.4 (C), 168.3 (C), 122.7 (q, $^1J_{C-F}$ = 277.3 Hz, CF_3), 61.2 (q, $^2J_{C-F}$ = 37.0 Hz, CH_2), 45.7 (CH), 13.5 (CH_3).
^{19}F{^{1}H} NMR (377 MHz, CDCl$_3$): δ –73.8 (s, 3 F).

Compound **4ea**:[27] Following *GP5* performed with (−)-menthol (**8e**, 722 mg, 4.6 mmol, 1.1 equiv), the desired product **4ea** was obtained as a colorless oil (0.297 g, 28%).
^{1}H NMR (400 MHz, CDCl$_3$): *(2 diastereomers)* δ 9.94 (br s, 2H), 4.76–4.66 (m, 2H), 3.51–3.40 (m, 2H), 2.06–1.95 (m, 2H), 1.92–1.79 (m, 2H), 1.72–1.62 (m, 4H), 1.53–1.35 (m, 10H), 1.12–0.92 (m, 4H), 0.93–0.82 (m, 14H), 0.78–0.68 (m, 6H).
^{13}C{^{1}H} NMR (100 MHz, CDCl$_3$): *(2 diastereomers)* δ 176.2 (C), 176.1 (C), 169.7 (C), 169.6 (C), 76.0 (CH), 76.0 (CH), 47.0 (CH), 47.0 (CH), 46.3 (CH), 46.3 (CH), 40.6 (CH_2), 40.4 (CH_2), 34.3 (2 CH_2), 31.5 (2 CH), 26.3 (CH), 26.1 (CH), 23.4 (CH_2), 23.4 (CH_2), 22.1 (2 CH_3), 20.8 (2 CH_3), 16.3 (CH_3), 16.1 (CH_3), 13.8 (CH_3), 13.7 (CH_3).

Compound **4ia**: Following *GP5* performed with phenol (**8i**, 440 mg, 4.6 mmol, 1.1 equiv), the desired product **4ia** was obtained as a colorless oil (0.136 g, 17%).
HRMS (ESI$^+$): $[M+Na]^+$ Calcd. for $C_{10}H_{10}NaO_4$: 217.0471. Found: 217.0471.
^{1}H NMR (400 MHz, CDCl$_3$): δ 10.92 (br s, 1H), 7.40 (t, *J* = 7.3 Hz, 2H), 7.26 (t, *J* = 7.3 Hz, 1H), 7.13 (d, *J* = 8.0 Hz, 2H), 3.77 (q, *J* = 7.2 Hz, 1H), 1.61 (d, *J* = 7.2 Hz, 3H).
^{13}C{^{1}H} NMR (100 MHz, CDCl$_3$): δ 176.1 (C), 168.4 (C), 150.5 (C), 129.6 (2 CH), 126.3 (CH), 121.4 (2 CH), 46.2 (CH), 13.6 (CH_3).

Compound **4ja**: Following *GP5* performed with (+)-citronellol (**8j**, 0.85 mL, 4.6 mmol, 1.1 equiv), the desired product **4ja** was obtained as a pale yellow oil (0.625 g, 58%).
HRMS (ESI$^+$): $[M+H]^+$ Calcd. for $C_{14}H_{25}O_4$: 257.1747. Found: 257.1750.
^{1}H NMR (400 MHz, CDCl$_3$): δ 9.20 (br s, 1H), 5.07 (t, *J* = 6.4 Hz, 1H), 4.19 (br s, 2H), 3.47 (q, *J* = 7.3 Hz, 1H), 2.00–1.92 (m, 2H), 1.71–1.66 (m, 1H), 1.67 (s, 3H), 1.59 (s, 3H), 1.56–1.40 (m, 2H), 1.44 (d, *J* = 7.3 Hz, 3H), 1.37–1.14 (m, 2H), 0.90 (d, *J* = 6.4 Hz, 3H).
^{13}C{^{1}H} NMR (100 MHz, CDCl$_3$): δ 175.9 (C), 170.1 (C), 131.5 (C), 124.6 (CH), 66.4 (CH_2), 46.1 (CH), 37.0 (CH_2), 35.3 (CH_2), 29.5 (CH), 25.8 (CH_3), 25.5 (CH_2), 19.4 (CH_3), 17.8 (CH_3), 13.7 (CH_3).

---

[26] Park, C.; Ha, M.W.; Kim, B.; Hong, S.; Kim, D.; Park, Y.; Kim, M.H.; Lee, J. K.; Lee, J.; Park, H.G. *Adv. Synth. Catal.* **2015**, *357*, 2841-2848.
[27] Ihara, M.; Takahashi, M.; Taniguchi, N.; Yasui, K.; Fukumoto, K.; Kametani, T *J. Chem. Soc., Perkin Trans. 1*, **1989**, 897-903.

Compound **4ka**: Following *GP5* performed with (*S*)-ethyl lactate (**8k**, 0.58 mL, 4.6 mmol, 1.1 equiv), the desired product **4ka** was obtained as a colorless oil (0.298 g, 32%).
HRMS (ESI$^+$): $[M+H]^+$ Calcd. for $C_9H_{15}O_6$: 219.0863. Found: 219.0864.
^{1}H NMR (400 MHz, $CDCl_3$): *(2 diastereomers)* δ 7.51 (br s, 2H), 5.12 (q, *J* = 7.0 Hz, 2H), 4.19 (q, *J* = 7.1 Hz, 4H), 3.62–3.51 (m, 2H), 1.50 (d, *J* = 7.2 Hz, 6H), 1.47 (d, *J* = 7.4 Hz, 6H), 1.26 (t, *J* = 7.1 Hz, 6H).
^{13}C{^{1}H} NMR (100 MHz, $CDCl_3$): *(2 diastereomers)* δ 174.9 (C), 174.6 (C), 170.6 (C), 170.5 (C), 169.6 (C), 169.4 (C), 69.7 (CH), 69.7 (CH), 61.7 (CH_2), 61.7 (CH_2), 46.0 (CH), 45.7 (CH), 16.8 (2 CH_3), 14.1 (CH_3), 14.1 (CH_3), 13.7 (CH_3), 13.6 (CH_3).

General procedure 6 (GP6) - opening of meldrum acid derivatives: In a manner similar to [19], a RBF equipped with a stirring bar was charged with the substituted meldrum acid **7** (1.0 equiv), the alcohol **8** (1.0 equiv), and toluene (*c* = 0.25 M). The reaction mixture was stirred at reflux for 4 h (or at 80 °C for 24 h). Then, the mixture was diluted with Et_2O (20 mL), and the resulting organic layer was extracted with saturated aqueous $NaHCO_3$ (2 times). The combined aqueous layers were cooled to 0 °C (ice/water bath) and acidified to pH 1 with 1 M aq HCl, saturated with NaCl, and extracted with Et_2O (3 × 20 mL). The combined organic layers were dried (Na_2SO_4) and evaporated to afford the expected product **4**.

Compound **4ch**: Following *GP6* performed with **7h** (0.50 g, 2.1 mmol, 1.0 equiv) and propan-2-ol (**8c**, 0.19 mL, 2.5 mmol, 1.0 equiv) in toluene at 80 °C for 24 h, the desired product **4ch** was obtained as a pale yellow oil (0.462 g, 93%).
HRMS (ESI$^+$): m/z $[M+Na]^+$ Calcd. for $C_{13}H_{16}NaO_4$: 259.0940. Found: 259.0938.
^{1}H NMR (400 MHz, $CDCl_3$): δ 10.61 (br s, 1H), 7.35–7.29 (m, 2H), 7.28–7.23 (m, 3H), 5.05 (hept, *J* = 6.3 Hz, 1H), 3.72 (t, *J* = 7.8 Hz, 1H), 3.27 (d, *J* = 7.8 Hz, 2H), 1.25 (d, *J* = 6.3 Hz, 3H), 1.16 (d, *J* = 6.3 Hz, 3H).
^{13}C{^{1}H} NMR (100 MHz, $CDCl_3$): δ 174.8 (C), 168.3 (C), 137.5 (C), 128.9 (2 CH), 128.7 (2 CH), 127.0 (CH), 69.7 (CH), 53.8 (CH), 34.7 (CH_2), 21.6 (2 CH_3).

Compound **4dh**: Following *GP6* performed with **7h** (0.50 g, 2.1 mmol, 1.0 equiv) and benzyl alcohol (**8d**, 0.22 mL, 2.1 mmol, 1.0 equiv) in refluxing toluene for 4 h, the desired product **4dh** was obtained as a pale yellow oil contaminated by ca. 13% of the decarboxylated product (0.392 g, 57%).
HRMS (ESI$^+$): m/z $[M+Na]^+$ Calcd. for $C_{17}H_{16}NaO_4$: 307.0940. Found: 307.0941.
^{1}H NMR (400 MHz, $CDCl_3$): δ 10.71 (br s, 1H), 7.41–7.21 (m, 10H), 5.19 (s, 2H), 3.84 (t, *J* = 7.7 Hz, 1H), 3.31 (d, *J* = 7.7 Hz, 2H).
^{13}C{^{1}H} NMR (100 MHz, $CDCl_3$): δ 174.6 (C), 168.6 (C), 137.3 (C), 135.1 (C), 128.9 (2 CH), 128.7 (2 CH), 128.7 (2 CH), 128.5 (CH), 128.3 (2 CH), 127.0 (CH), 67.5 (CH_2), 53.7 (CH), 34. 8 (CH_2).

Compound **4da**: Following *GP6* performed with **7a** (0.32 g, 2.0 mmol, 1.0 equiv) and benzyl alcohol (**8d**, 0.21 mL, 2.0 mmol, 1.0 equiv) in refluxing toluene for 8 h, the desired product **4da** was obtained as a pale yellow oil (0.357 g, 85%).

Compound **4ea**: Following *GP6* performed with **7a** (0.32 g, 2.0 mmol, 1.0 equiv) and (−)-menthol (**8e**, 0.312 g, 2.0 mmol, 1.0 equiv) in refluxing toluene for 5 h, the desired product **4ea** was obtained as a colorless oil (0.375 g, 58%).

Compound **4bs**:[28] Following *GP6* performed with **7s** (0.440 g, 2.0 mmol, 1.0 equiv) in a refluxing mixture of toluene and EtOH 1:1 for 8 h, the desired product **4bs** was obtained as white solid (0.272 g, 65%).
^{1}H NMR (400 MHz, $CDCl_3$): δ 9.52 (br s, 1H), 7.46–7.29 (m, 5H), 4.65 (s, 1H), 4.30–4.15 (m, 2H), 1.26 (t, *J* = 7.1 Hz, 3H).
^{13}C{^{1}H} NMR (100 MHz, $CDCl_3$): δ 173.5 (C), 168.3 (C), 132.3 (C), 129.3 (2 CH), 128.9 (2 CH), 128.6 (CH), 62.3 (CH_2), 57.7 (CH), 14.1 (CH_3).

6. Copies of NMR spectra

[28] Bagum, H.; Christensen, K.E.; Genov, M.; Pretsch, A.; Pretsch, D.; Moloney, M. G. *Tetrahedron* **2019**, *75*, 130561.

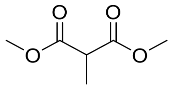

3aa

^1H, $CDCl_3$, 400 MHz

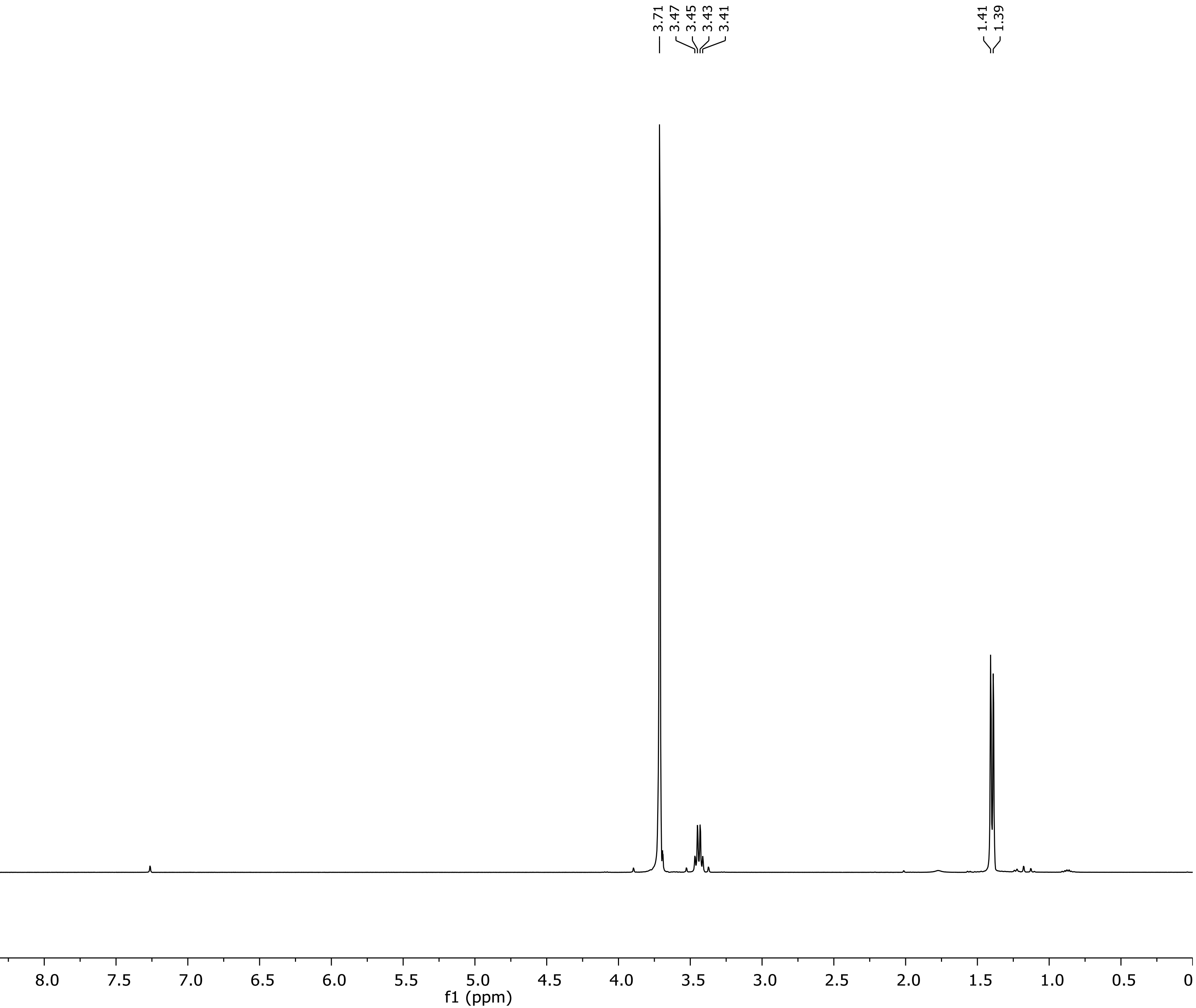

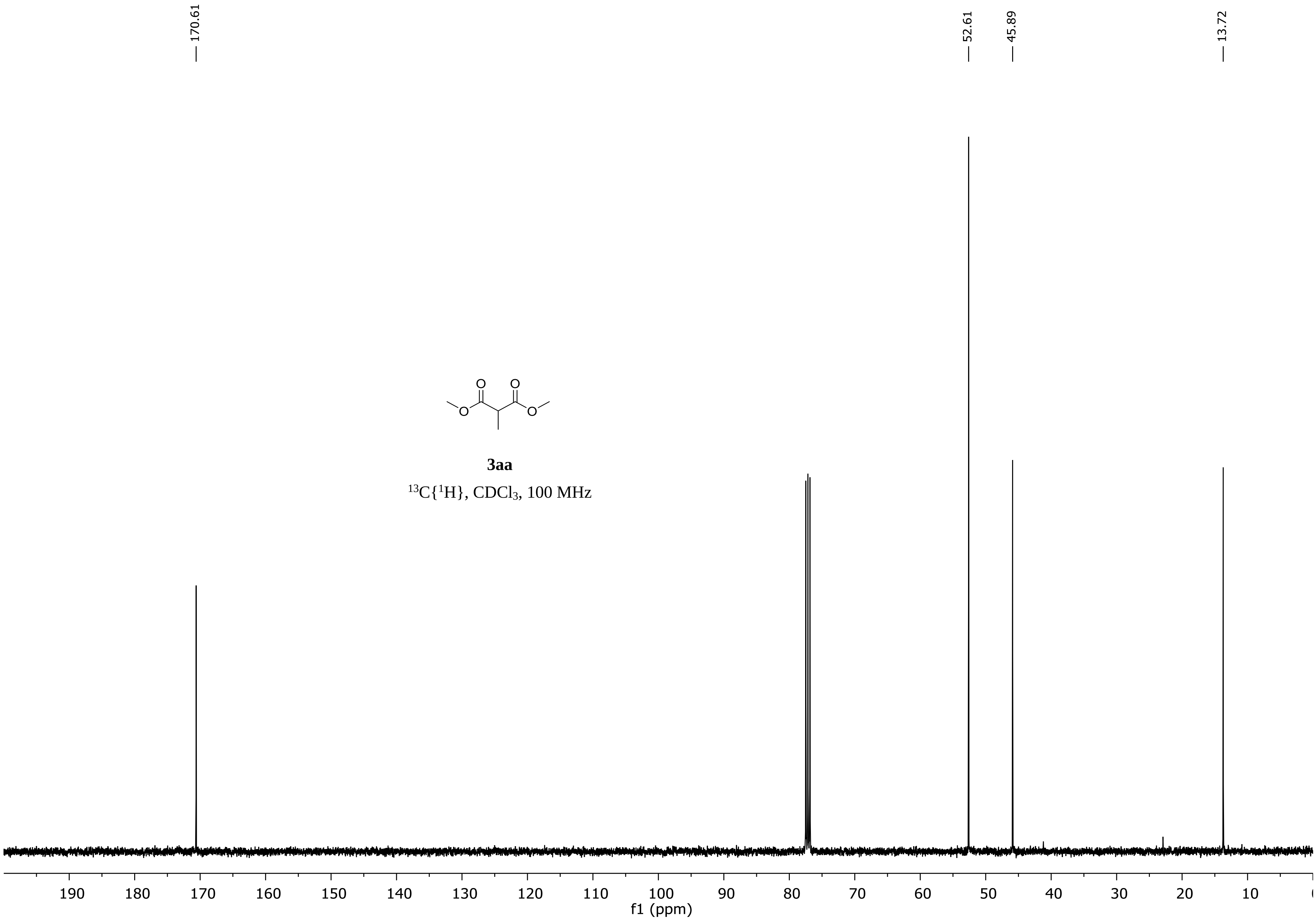
170.61
52.61
45.89
13.72
3aa
$^{13}C\{^{1}H\}$, $CDCl_3$, 100 MHz
190
180
170
160
150
140
130
120
110
100
90
80
70
60
50
40
30
20
10
f1 (ppm)

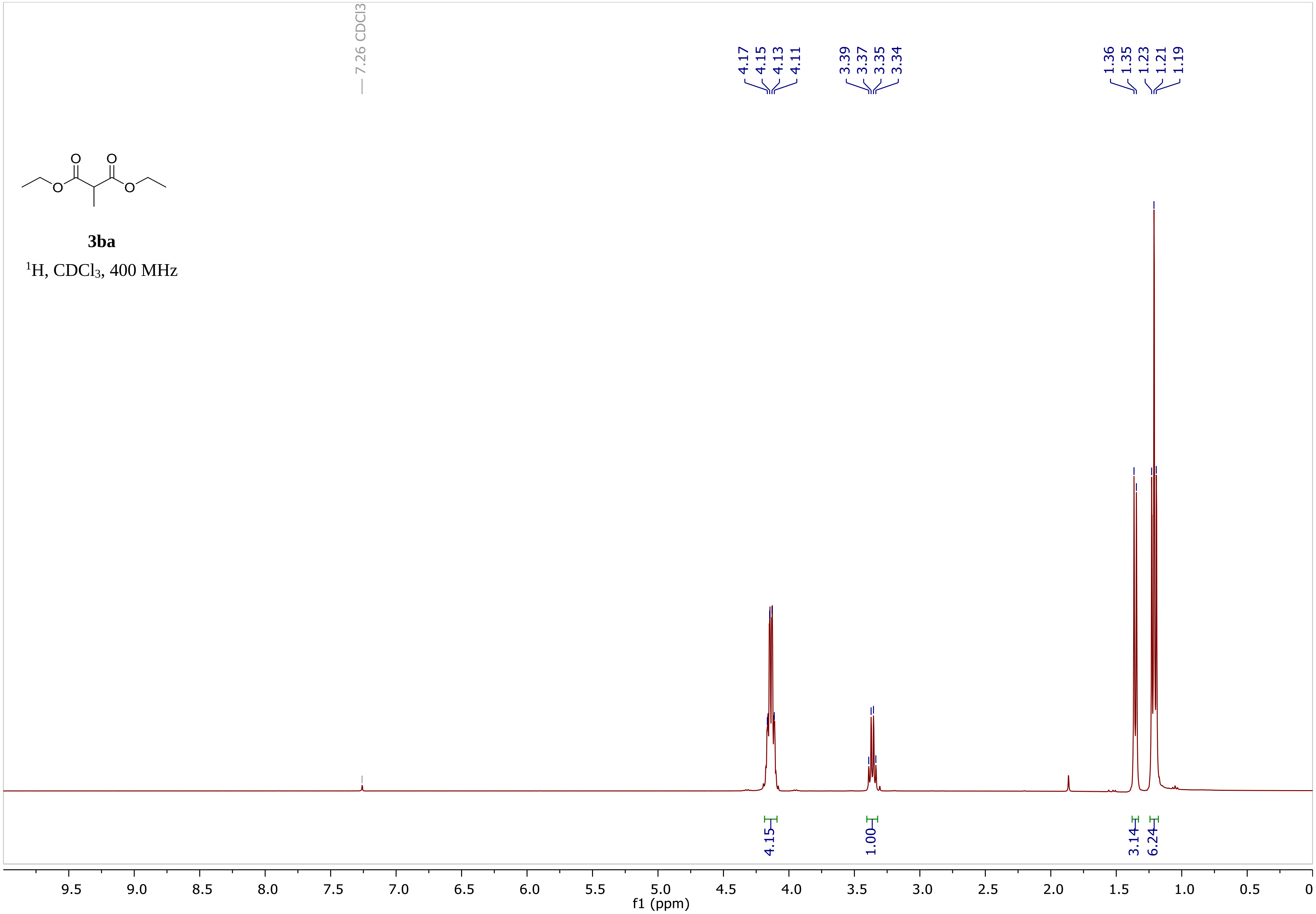

3ba

^{1}H, $CDCl_3$, 400 MHz

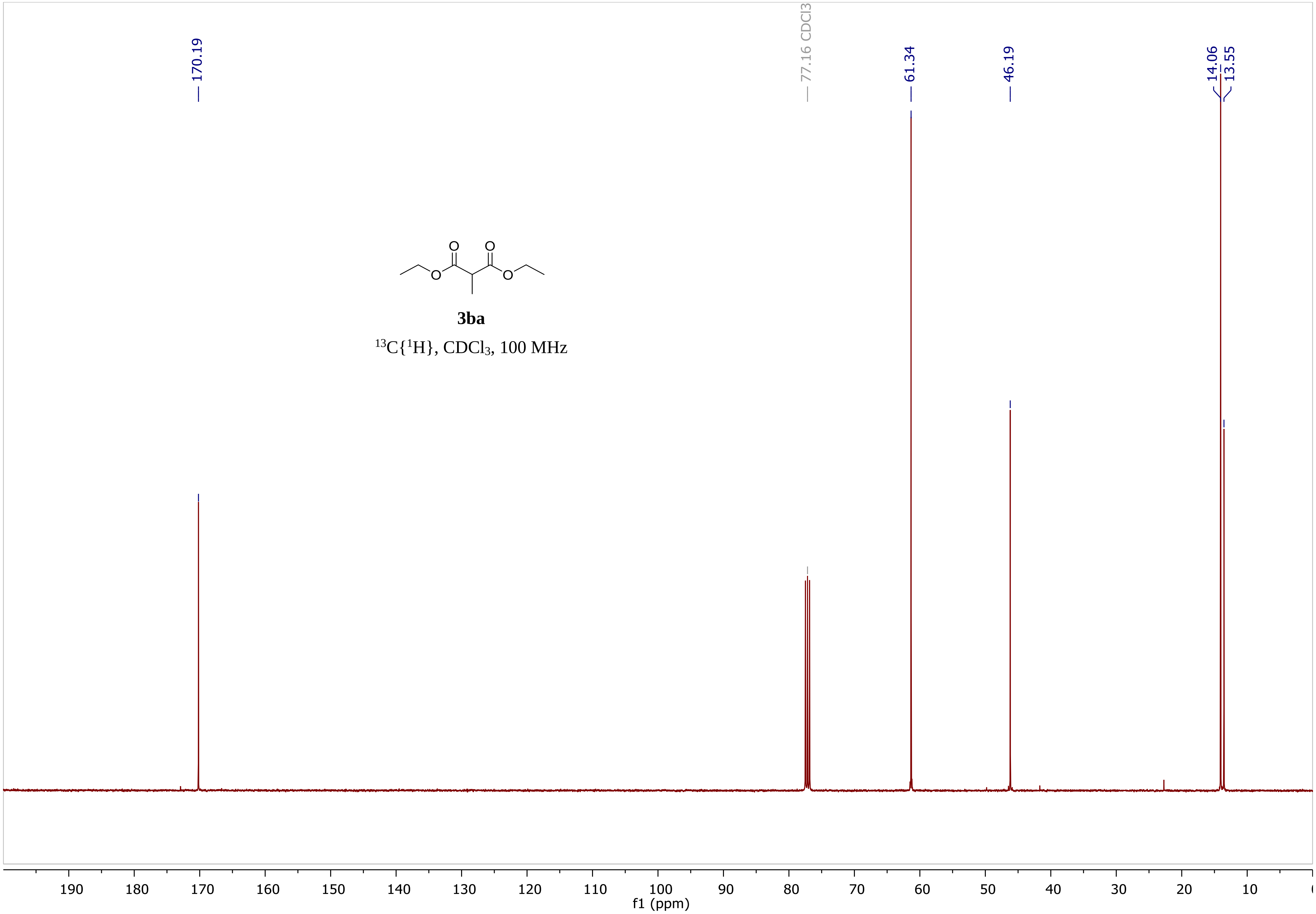
170.19
77.16 CDCl3
61.34
46.19
14.06
13.55
3ba
$^{13}C\{^1H\}$, $CDCl_3$, 100 MHz
190 180 170 160 150 140 130 120 110 100 90 80 70 60 50 40 30 20 10
f1 (ppm)

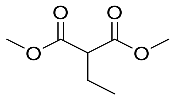

3ab

^{1}H, $CDCl_3$, 400 MHz

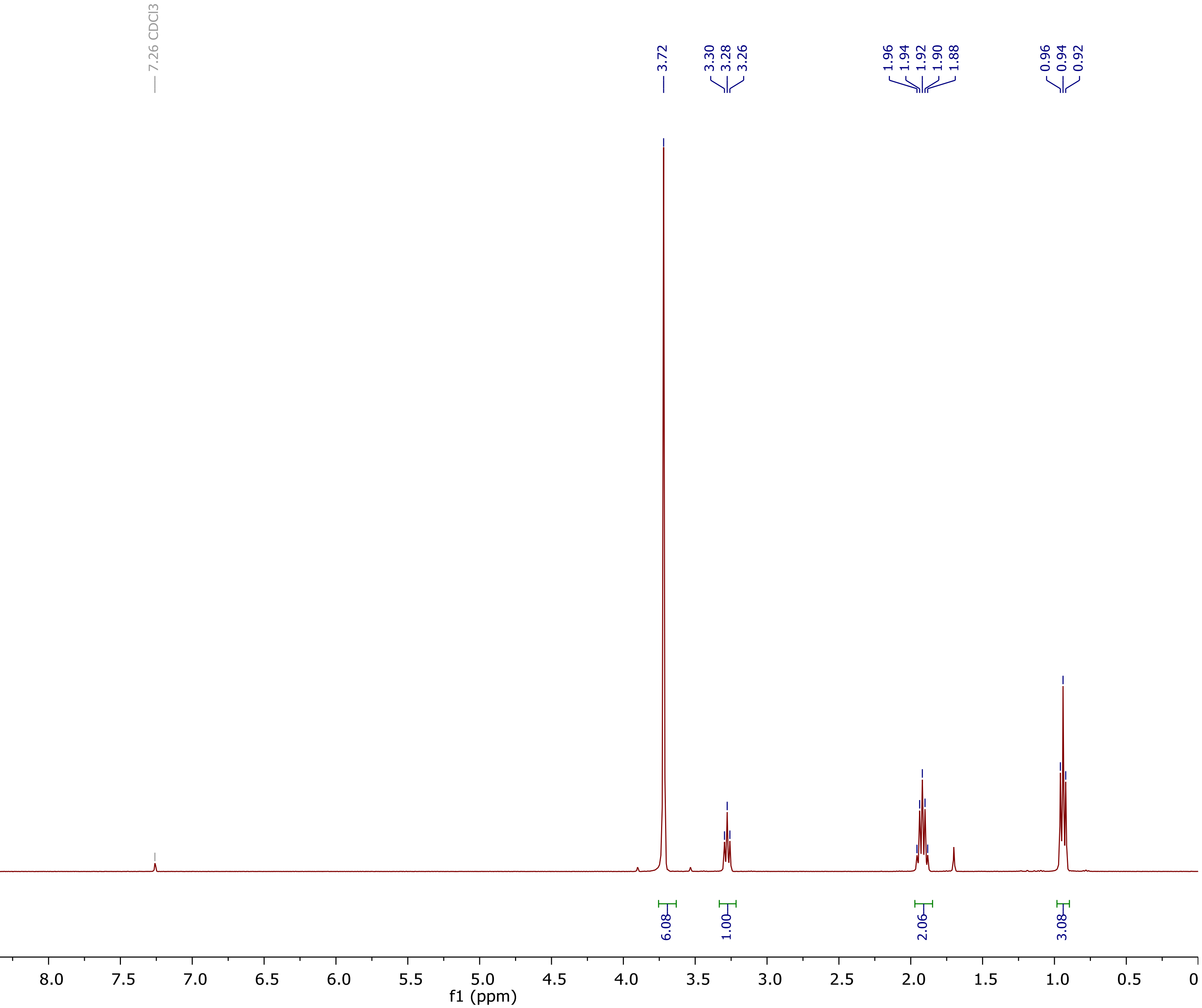

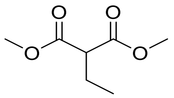

3ab

$^{13}C\{^{1}H\}$, $CDCl_3$, 100 MHz

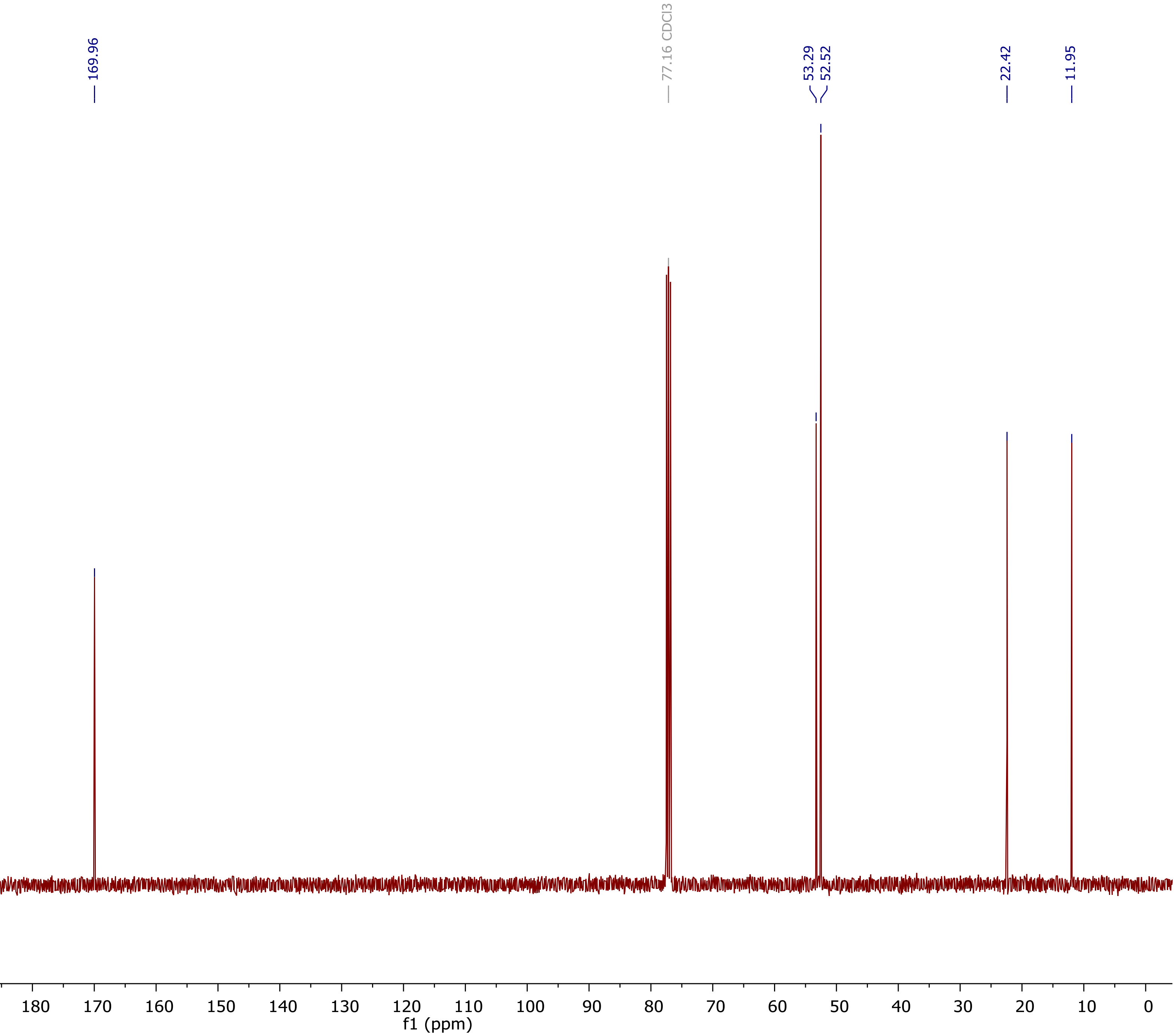

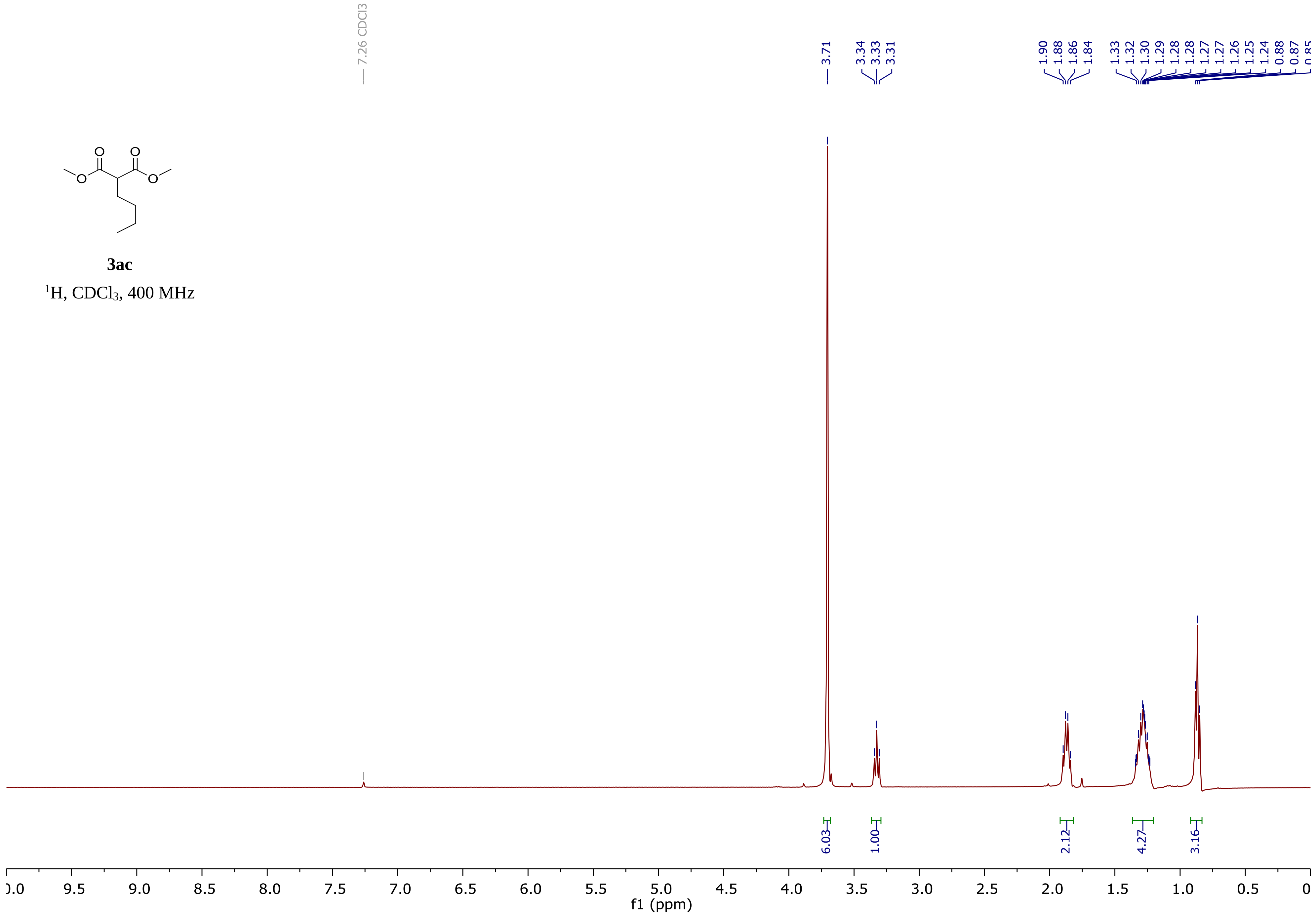

3ac

^{1}H, $CDCl_3$, 400 MHz

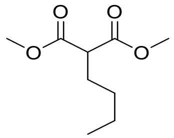

3ac

$^{13}C\{^{1}H\}$, $CDCl_3$, 100 MHz

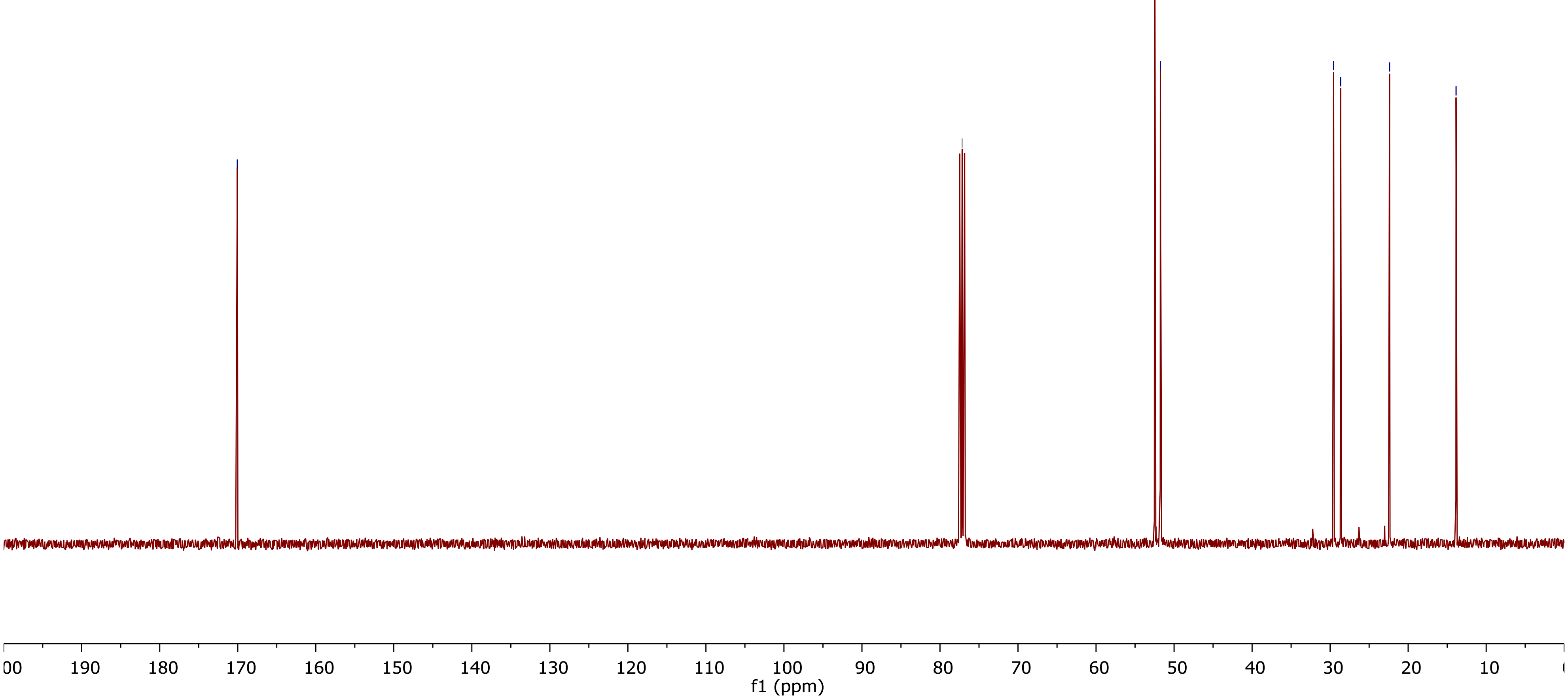

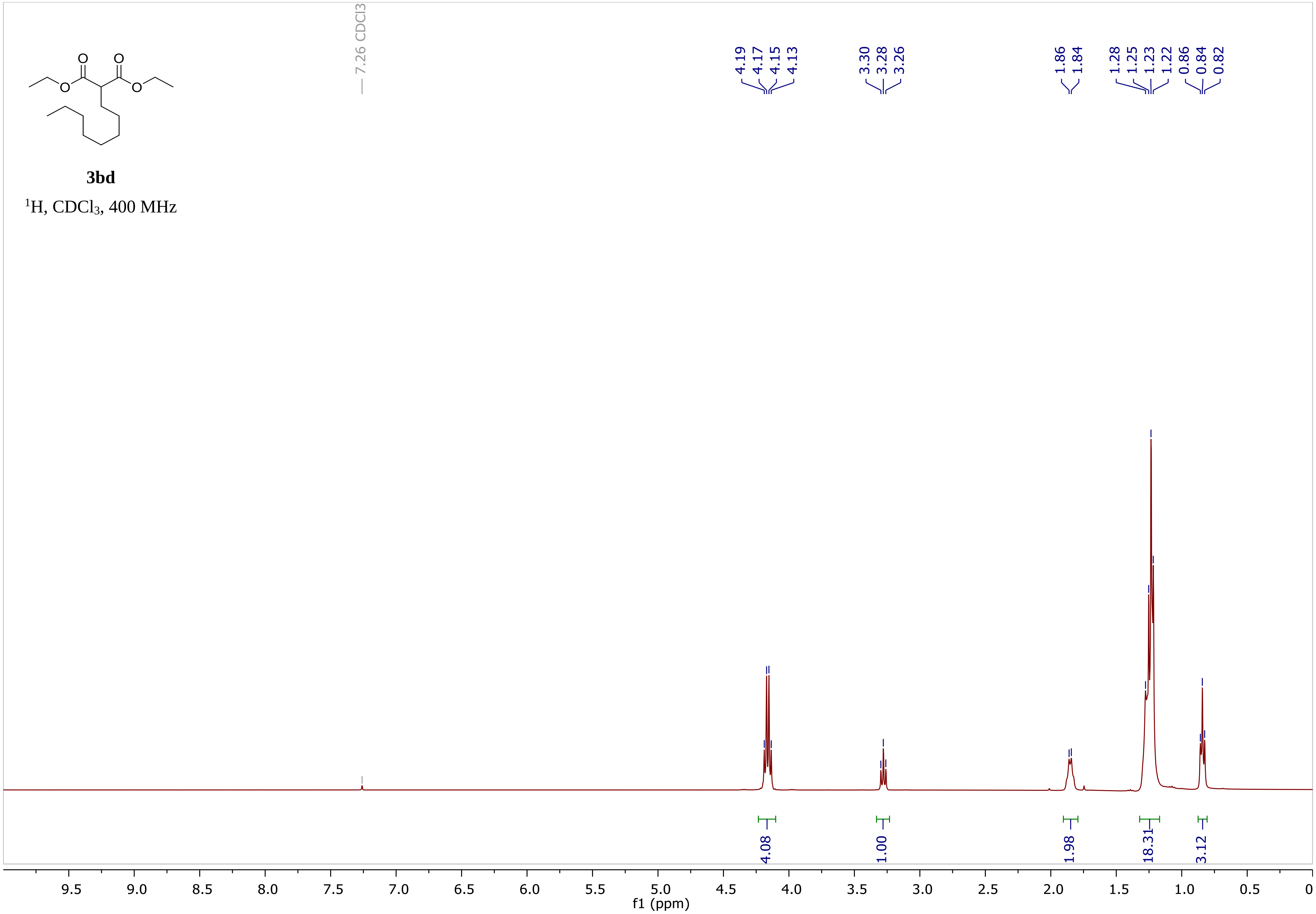

3bd
^{1}H, CDCl$_3$, 400 MHz
7.26 CDCl3
4.19
4.17
4.15
4.13
3.30
3.28
3.26
1.86
1.84
1.28
1.25
1.23
1.22
0.86
0.84
0.82
4.08
1.00
1.98
18.31
3.12
9.5 9.0 8.5 8.0 7.5 7.0 6.5 6.0 5.5 5.0 4.5 4.0 3.5 3.0 2.5 2.0 1.5 1.0 0.5 0
f1 (ppm)

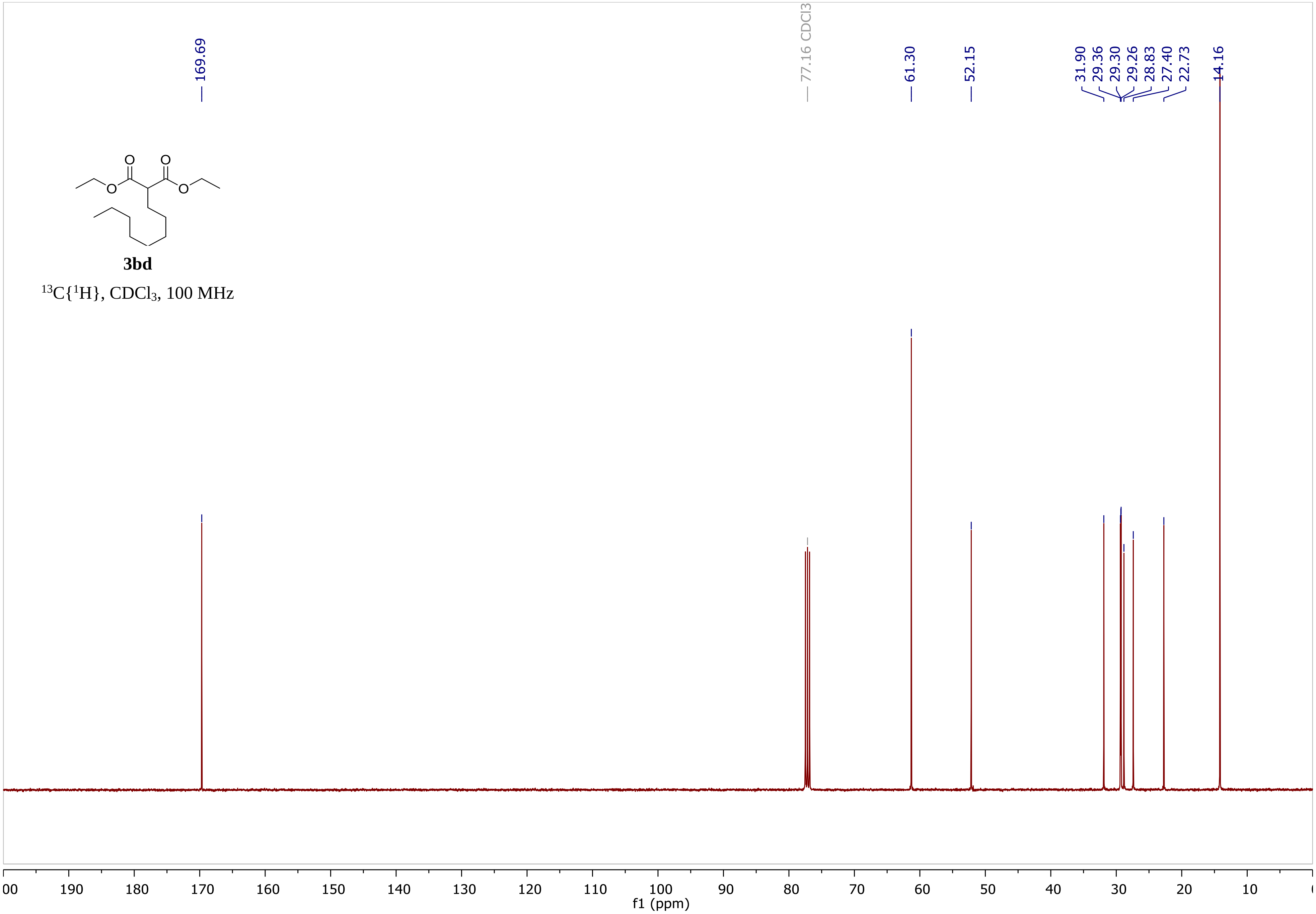
169.69
77.16 CDCl3
61.30
52.15
31.90
29.36
29.30
29.26
28.83
27.40
22.73
14.16
3bd
$^{13}C\{^{1}H\}$, $CDCl_3$, 100 MHz
00 190 180 170 160 150 140 130 120 110 100 90 80 70 60 50 40 30 20 10
f1 (ppm)

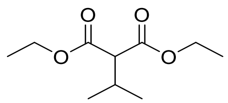

3be

^{1}H, $CDCl_3$, 400 MHz

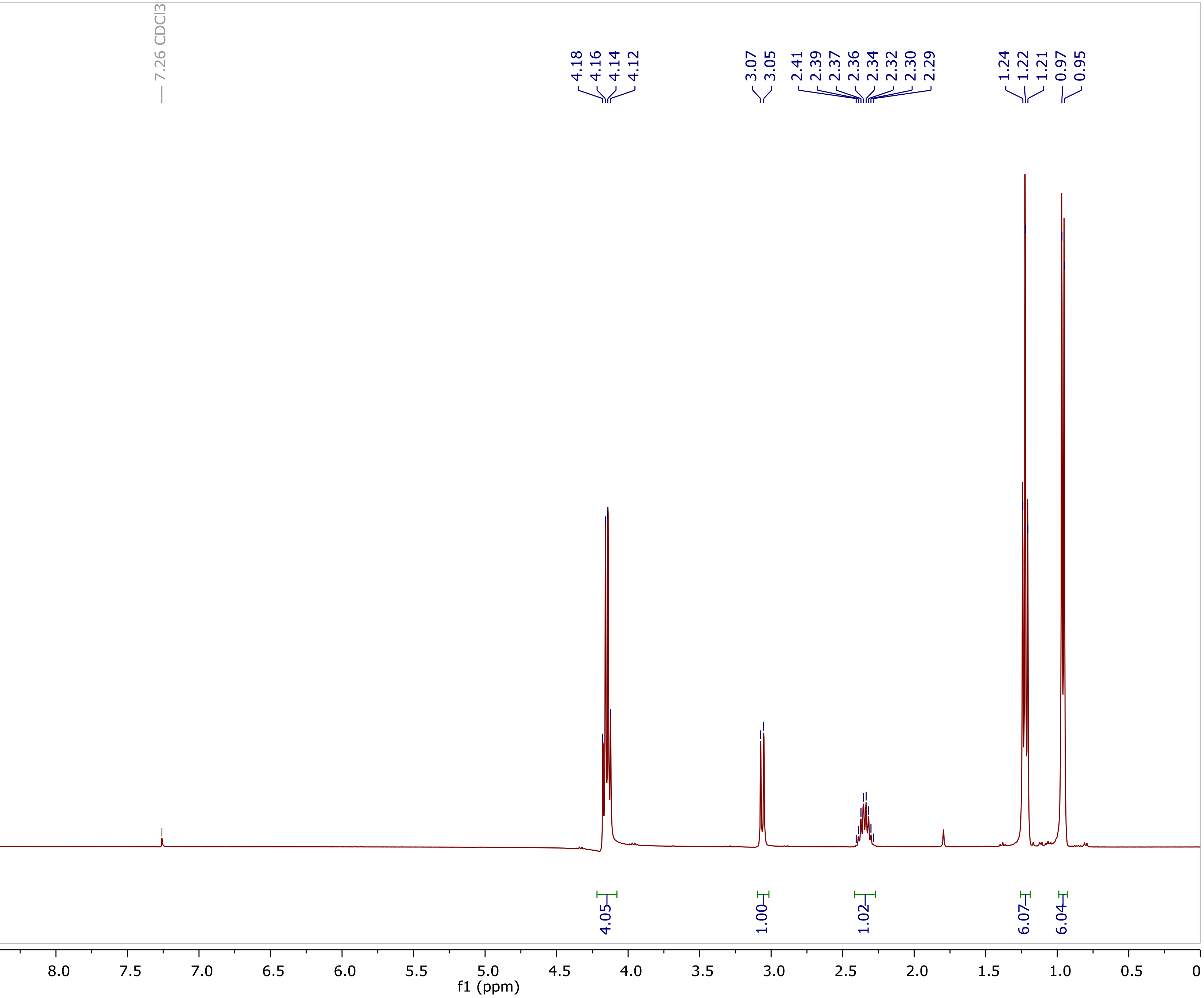

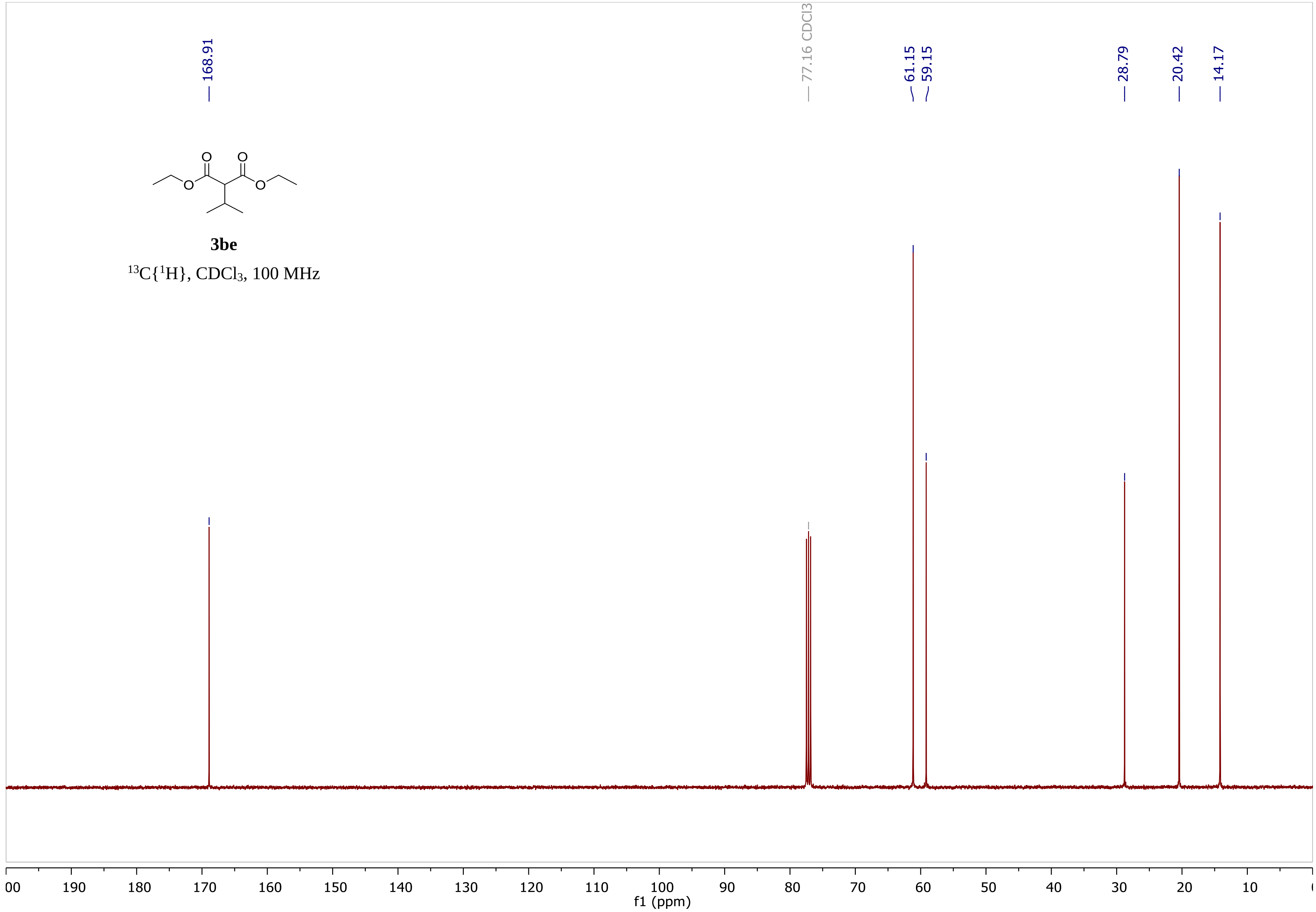
168.91
77.16 CDCl3
61.15
59.15
28.79
20.42
14.17
3be
$^{13}C\{^{1}H\}$, $CDCl_3$, 100 MHz
00 190 180 170 160 150 140 130 120 110 100 90 80 70 60 50 40 30 20 10
f1 (ppm)

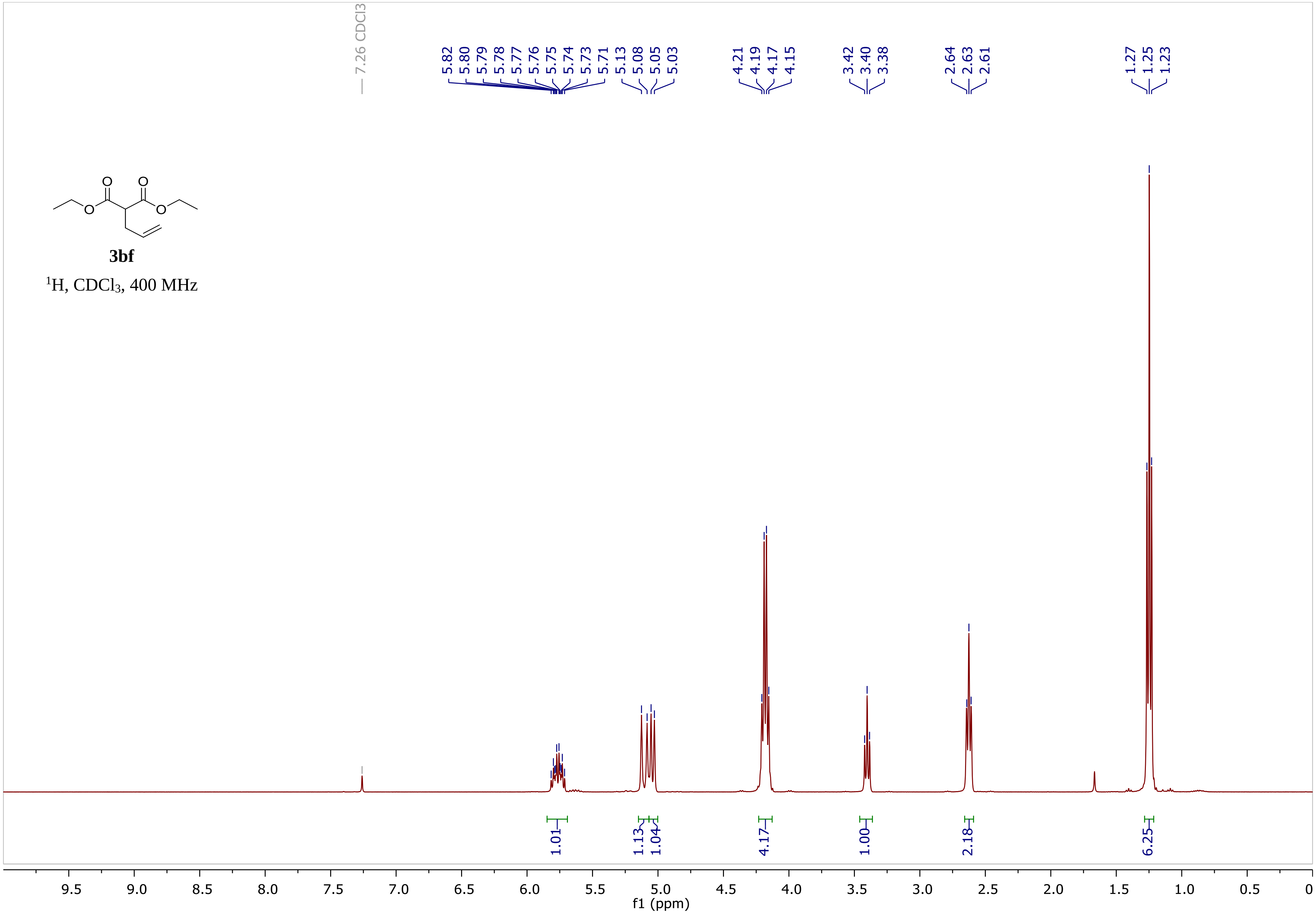
3bf
^{1}H, CDCl$_3$, 400 MHz
7.26 CDCl3
5.82
5.80
5.79
5.78
5.77
5.76
5.75
5.74
5.73
5.71
5.13
5.08
5.05
5.03
4.21
4.19
4.17
4.15
3.42
3.40
3.38
2.64
2.63
2.61
1.27
1.25
1.23
1.01
1.13
1.04
4.17
1.00
2.18
6.25
9.5 9.0 8.5 8.0 7.5 7.0 6.5 6.0 5.5 5.0 4.5 4.0 3.5 3.0 2.5 2.0 1.5 1.0 0.5 0
f1 (ppm)

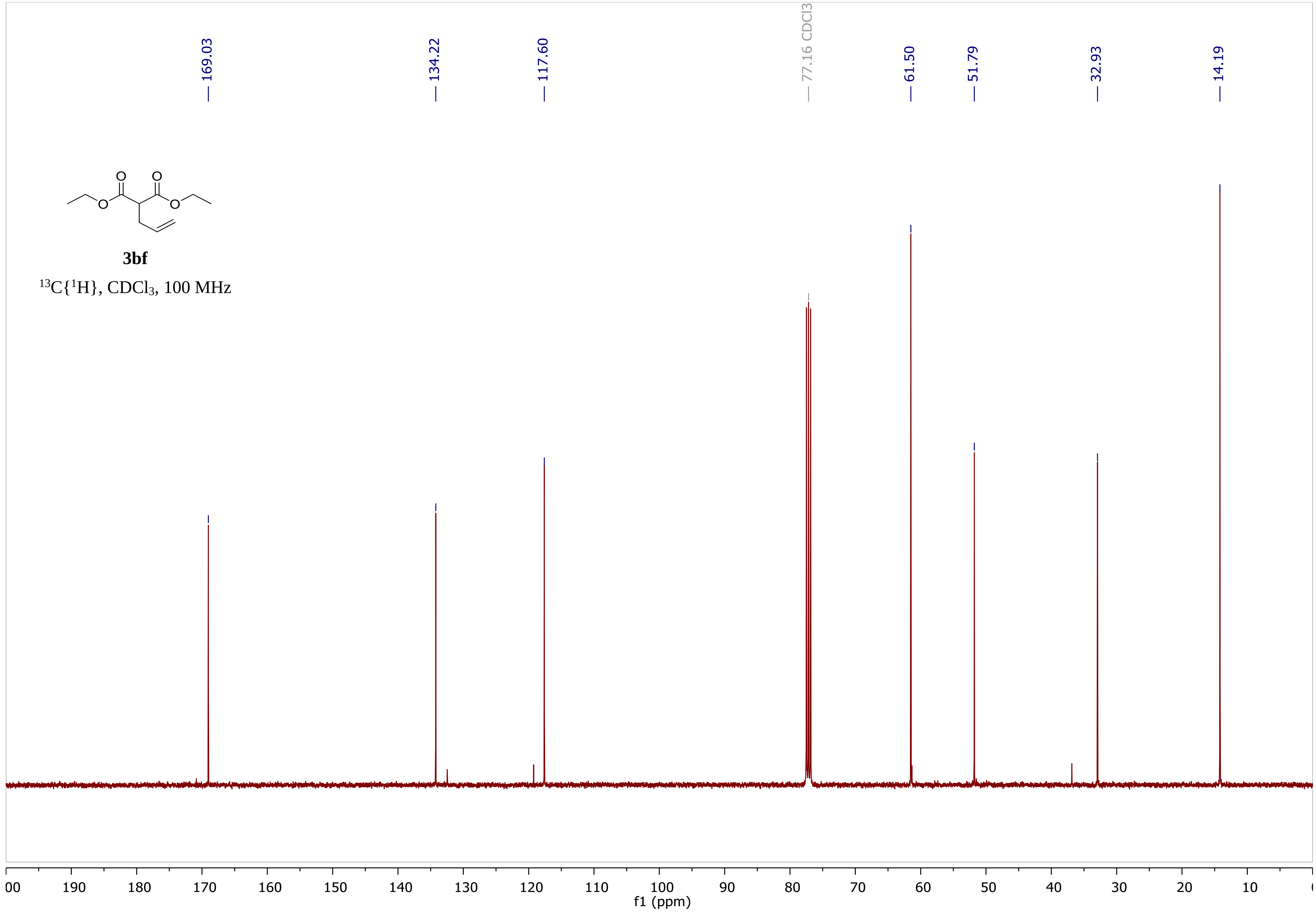
169.03
134.22
117.60
77.16 CDCl3
61.50
51.79
32.93
14.19
3bf
$^{13}C\{^{1}H\}$, $CDCl_3$, 100 MHz
f1 (ppm)

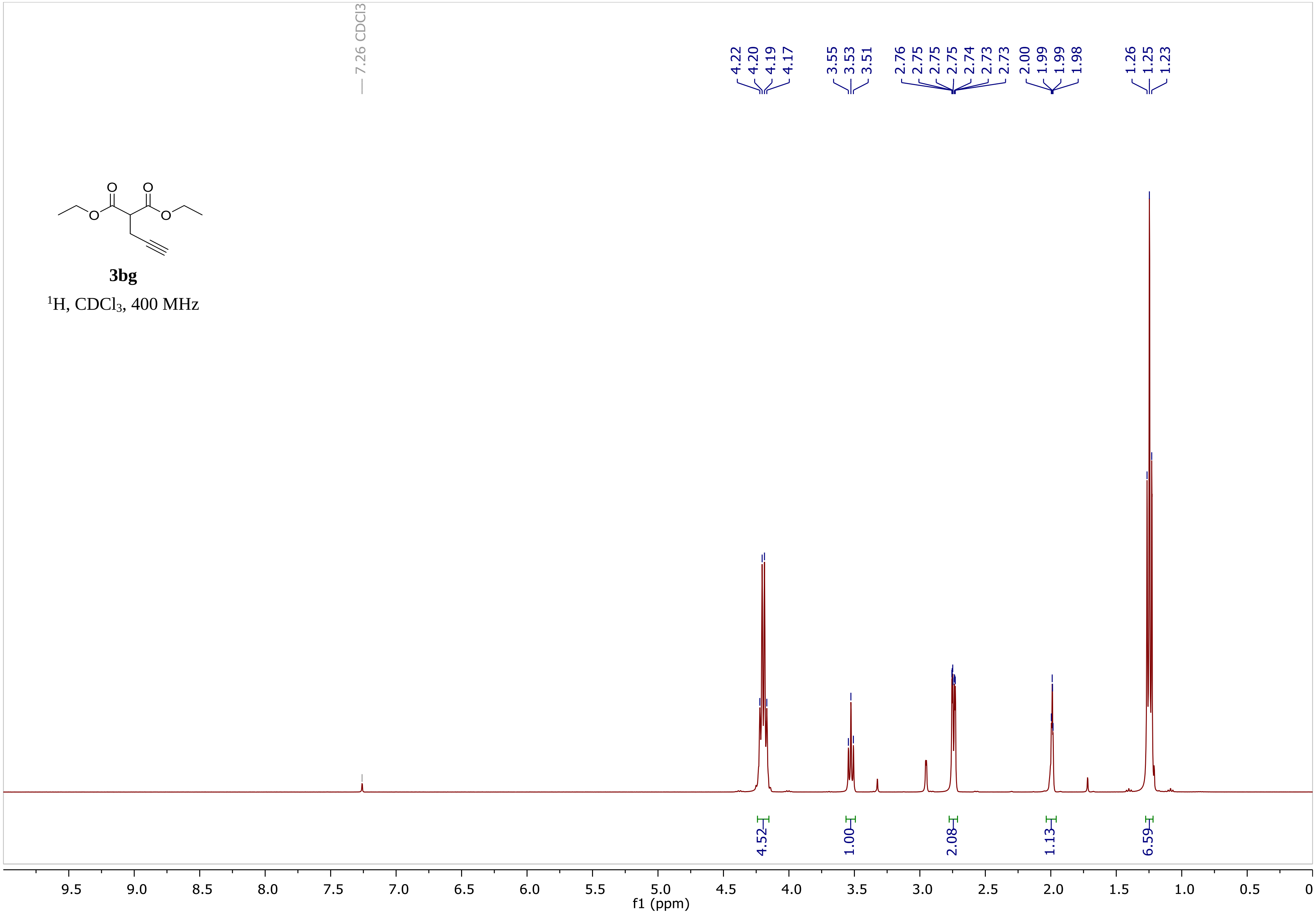

7.26 CDCl3
4.22
4.20
4.19
4.17
3.55
3.53
3.51
2.76
2.75
2.75
2.75
2.74
2.73
2.73
2.00
1.99
1.99
1.98
1.26
1.25
1.23
3bg
^{1}H, $CDCl_3$, 400 MHz
4.52
1.00
2.08
1.13
6.59
9.5
9.0
8.5
8.0
7.5
7.0
6.5
6.0
5.5
5.0
4.5
4.0
3.5
3.0
2.5
2.0
1.5
1.0
0.5
0
f1 (ppm)

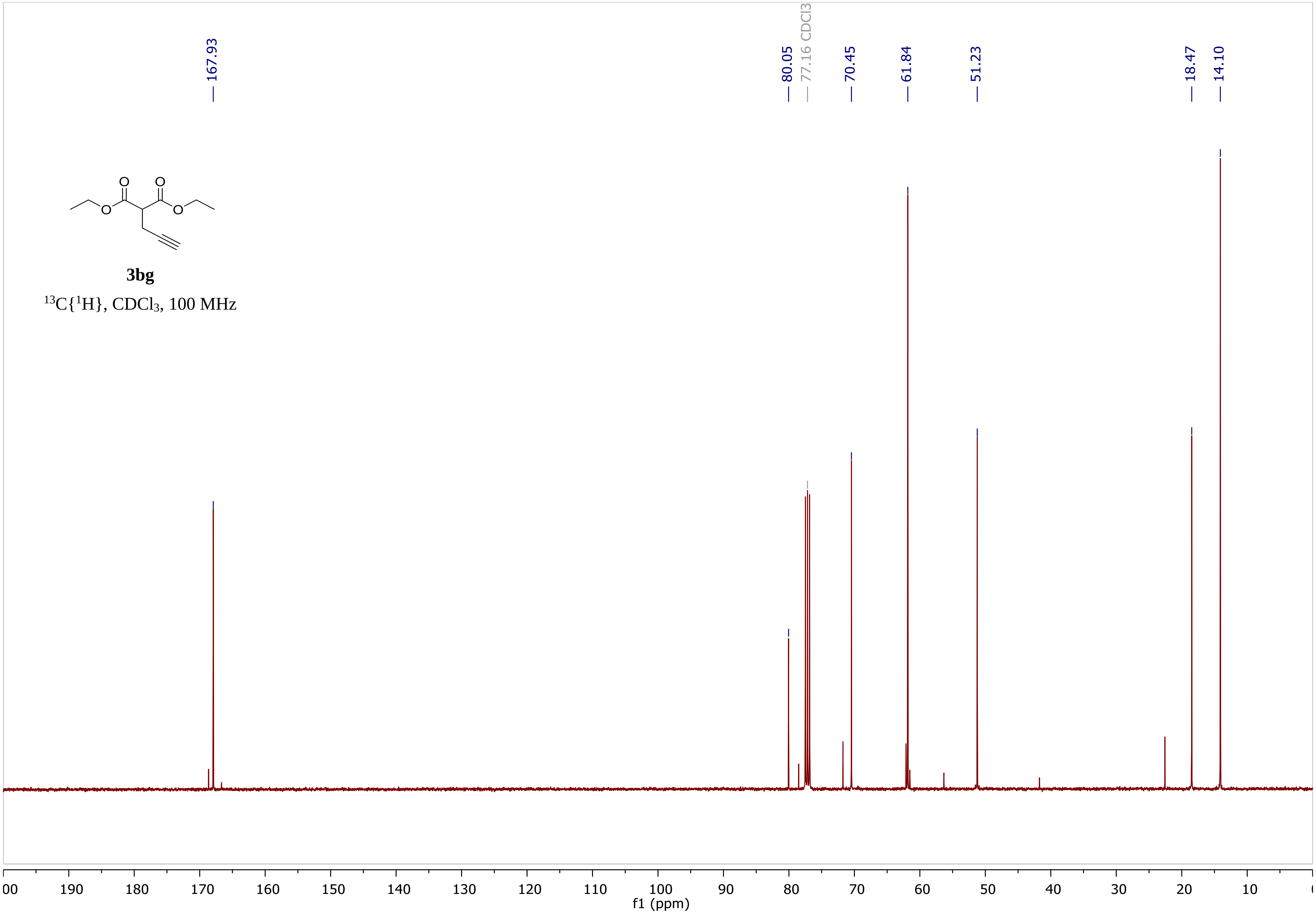
3bg
$^{13}C\{^{1}H\}$, $CDCl_3$, 100 MHz
167.93
80.05
77.16 CDCl3
70.45
61.84
51.23
18.47
14.10
00
190
180
170
160
150
140
130
120
110
100
90
80
70
60
50
40
30
20
10
f1 (ppm)

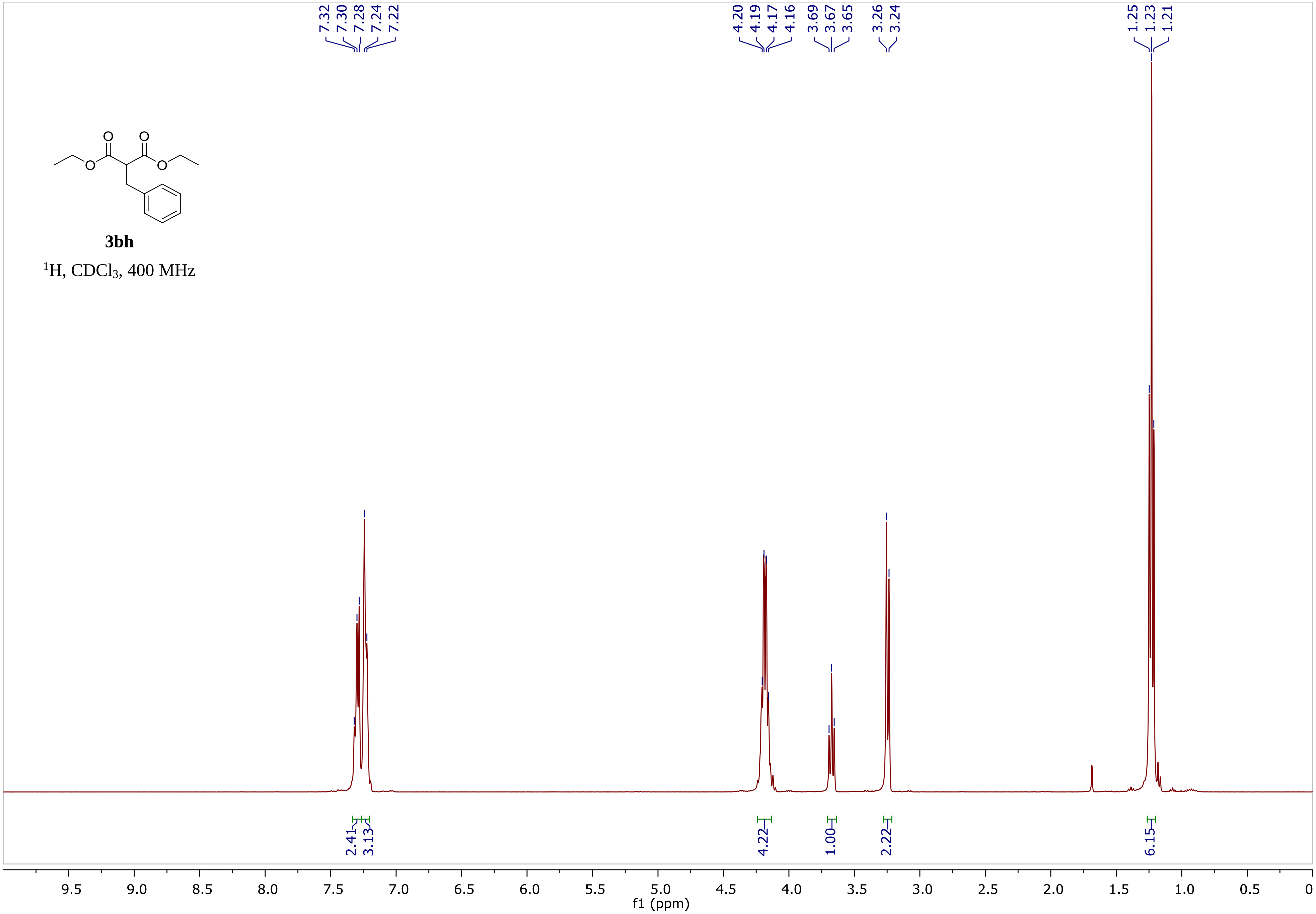
3bh
^{1}H, $CDCl_3$, 400 MHz
7.32
7.30
7.28
7.24
7.22
4.20
4.19
4.17
4.16
3.69
3.67
3.65
3.26
3.24
1.25
1.23
1.21
2.41
3.13
4.22
1.00
2.22
6.15
9.5
9.0
8.5
8.0
7.5
7.0
6.5
6.0
5.5
5.0
4.5
4.0
3.5
3.0
2.5
2.0
1.5
1.0
0.5
0
f1 (ppm)

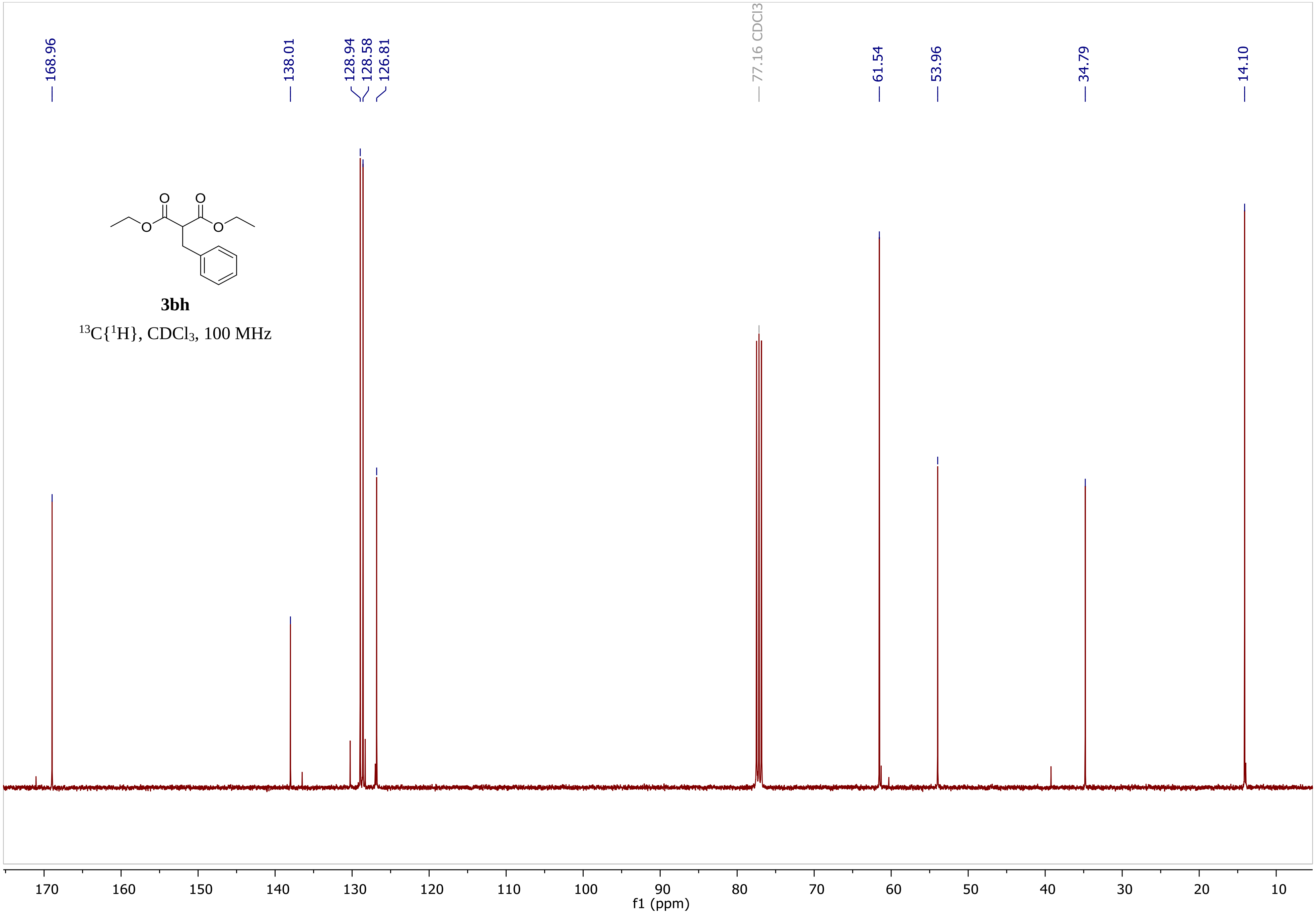

168.96
138.01
128.94
128.58
126.81
77.16 CDCl3
61.54
53.96
34.79
14.10
3bh
13C{1H}, CDCl3, 100 MHz
170
160
150
140
130
120
110
100
90
80
70
60
50
40
30
20
10
f1 (ppm)

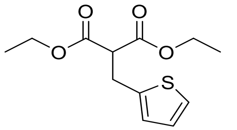

3bi

^{1}H, $CDCl_3$, 400 MHz

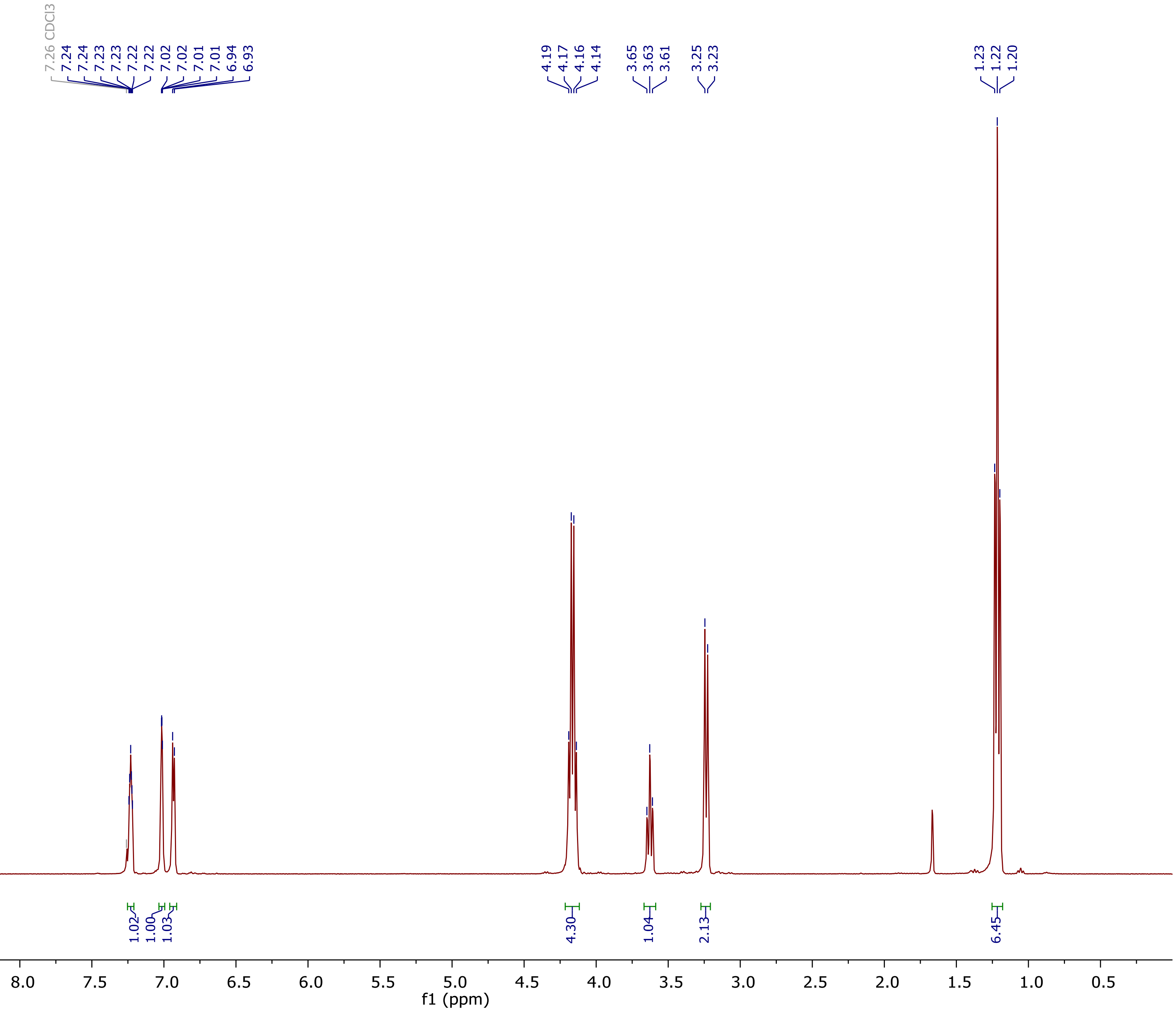

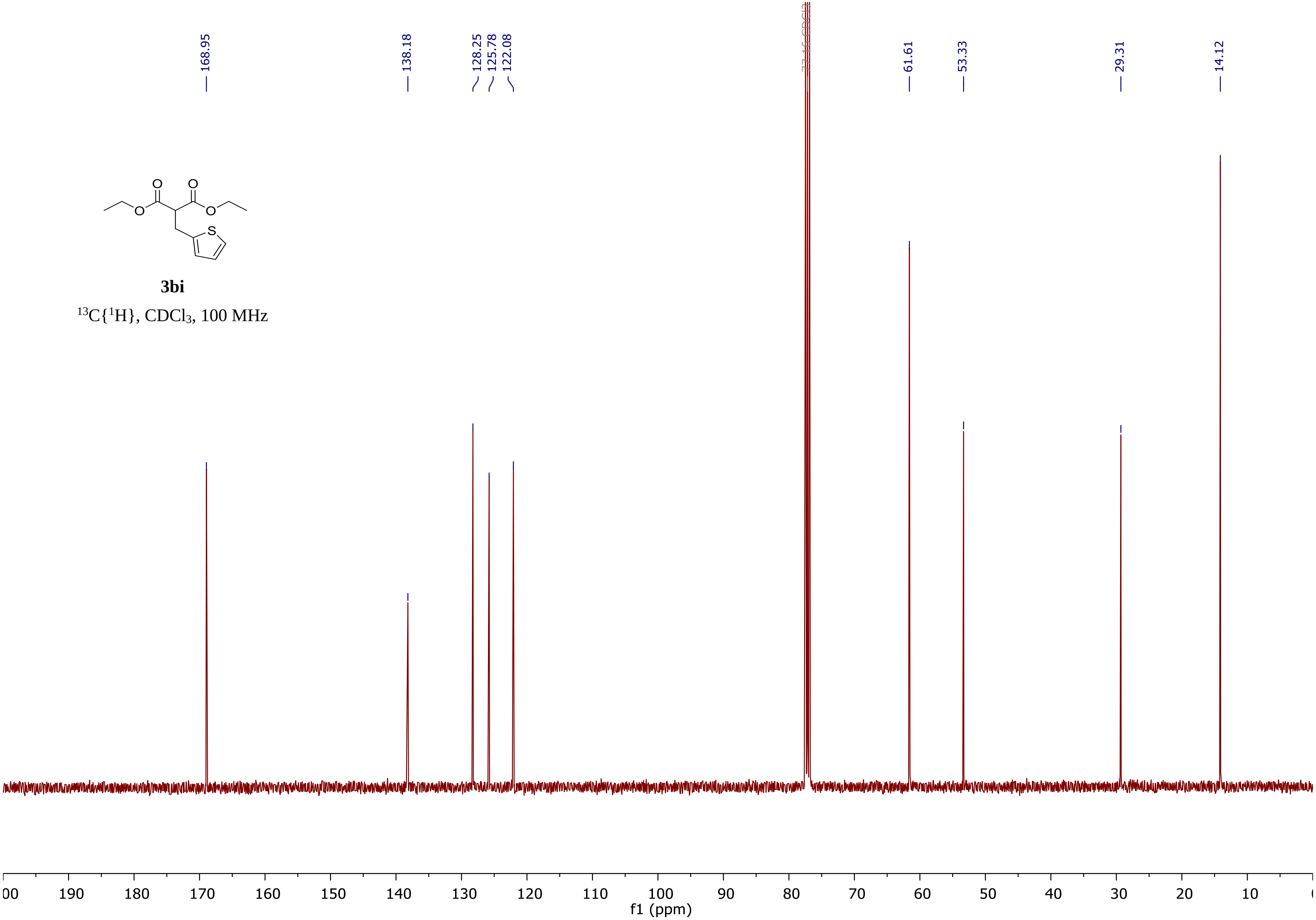

3bi
$^{13}C\{^{1}H\}$, $CDCl_3$, 100 MHz
168.95
138.18
128.25
125.78
122.08
77.16 CDCl3
61.61
53.33
29.31
14.12
00
190
180
170
160
150
140
130
120
110
100
90
80
70
60
50
40
30
20
10
f1 (ppm)

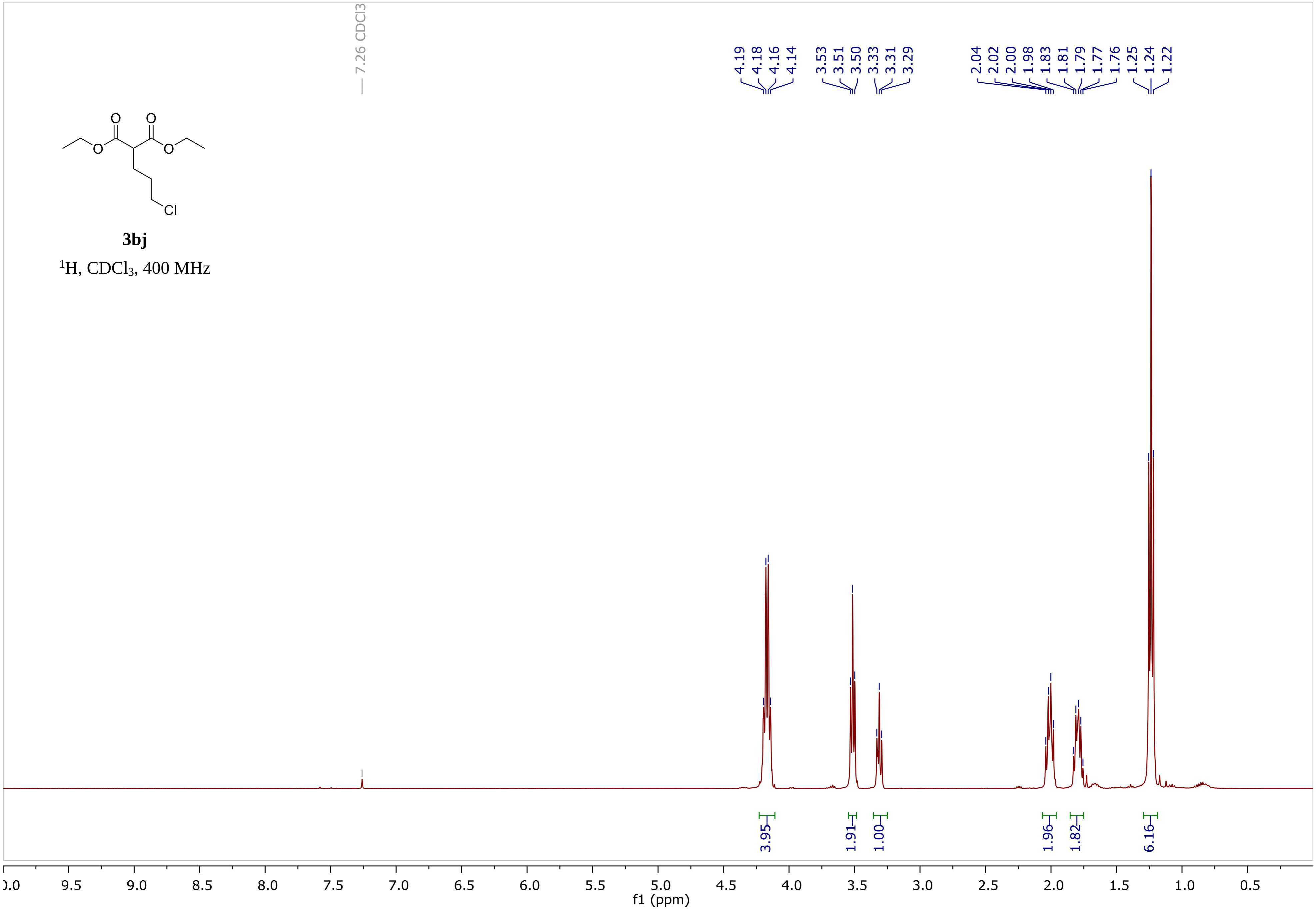

3bj

^{1}H, $CDCl_3$, 400 MHz

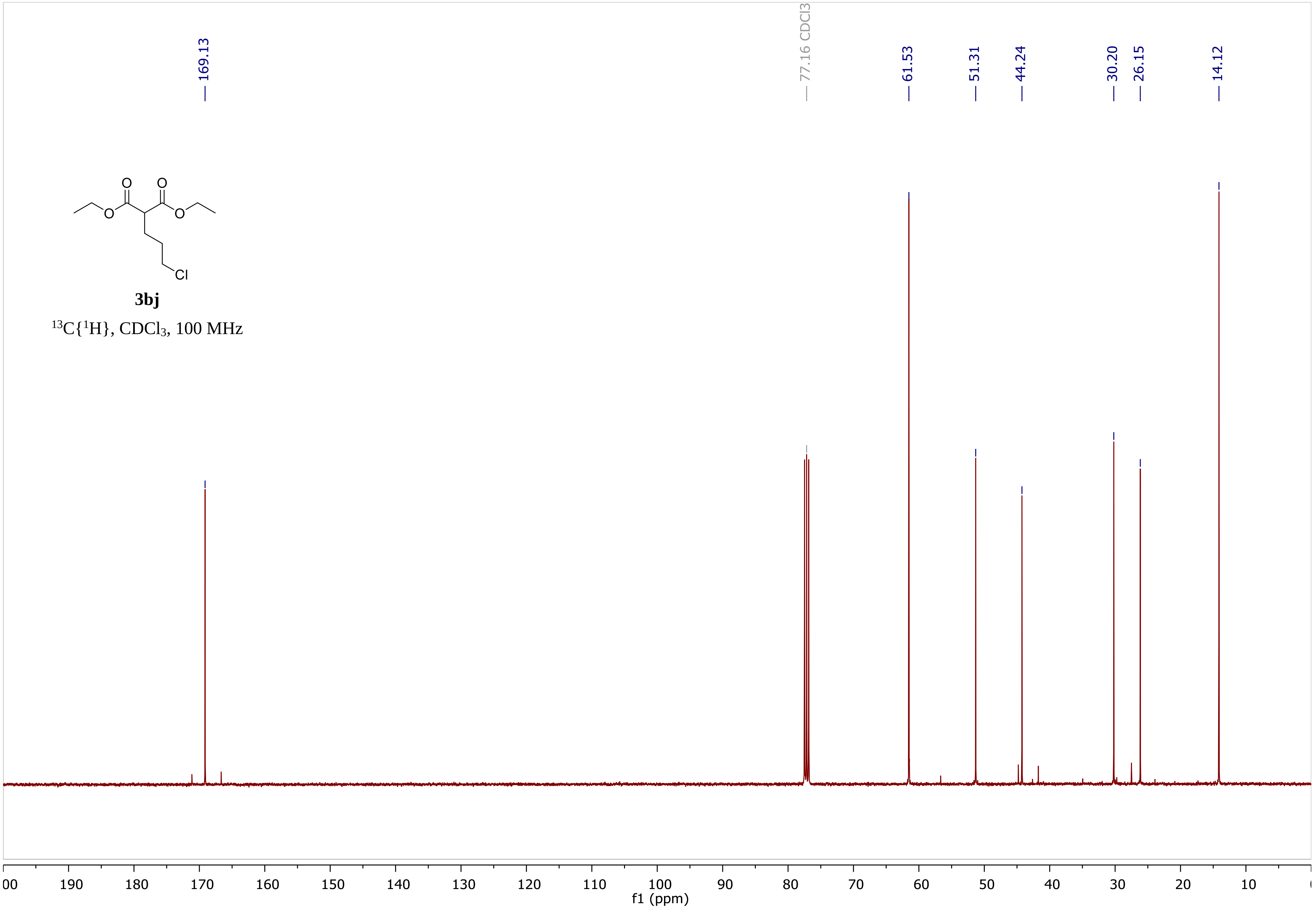
169.13
77.16 CDCl3
61.53
51.31
44.24
30.20
26.15
14.12
O
O
O
O
Cl
3bj
$^{13}C\{^{1}H\}$, $CDCl_3$, 100 MHz
00
190
180
170
160
150
140
130
120
110
100
90
80
70
60
50
40
30
20
10
f1 (ppm)

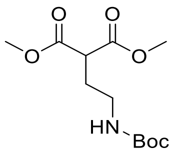

3ak

^{1}H, $CDCl_3$, 400 MHz

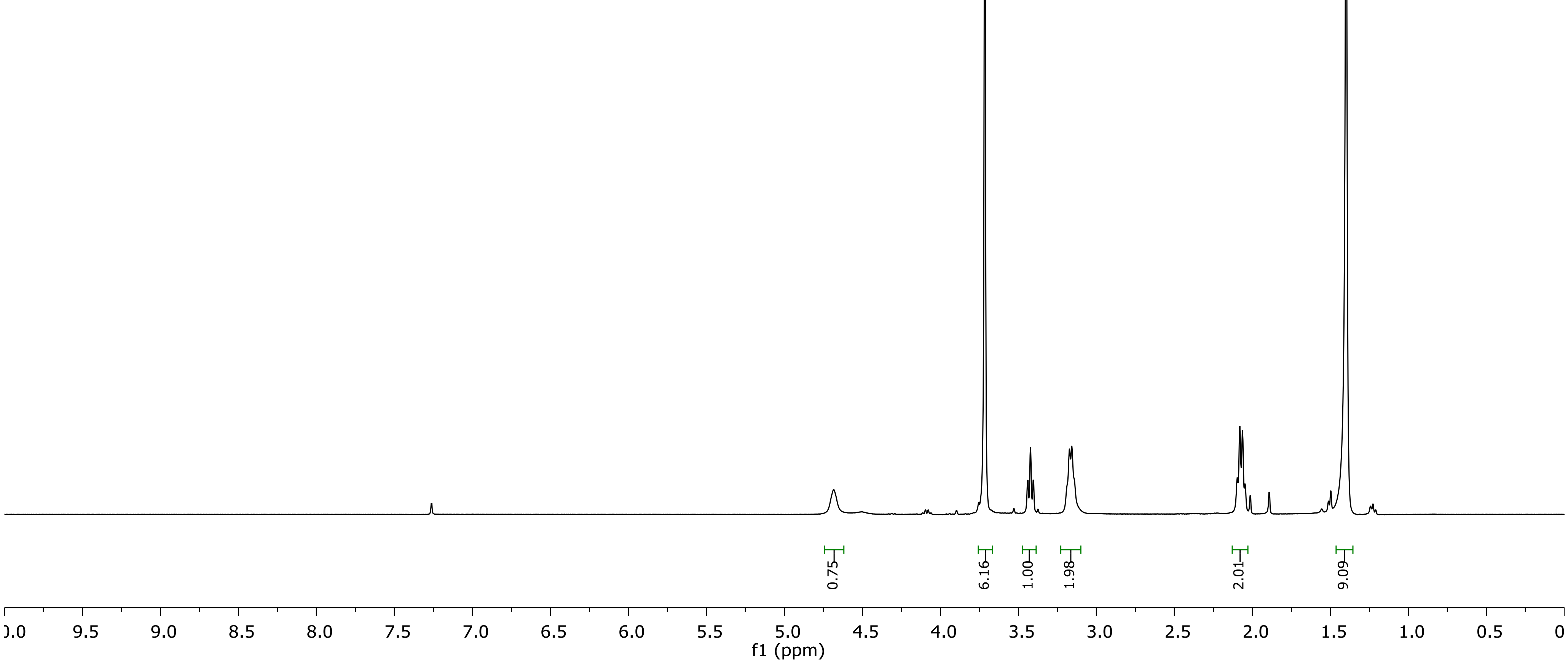

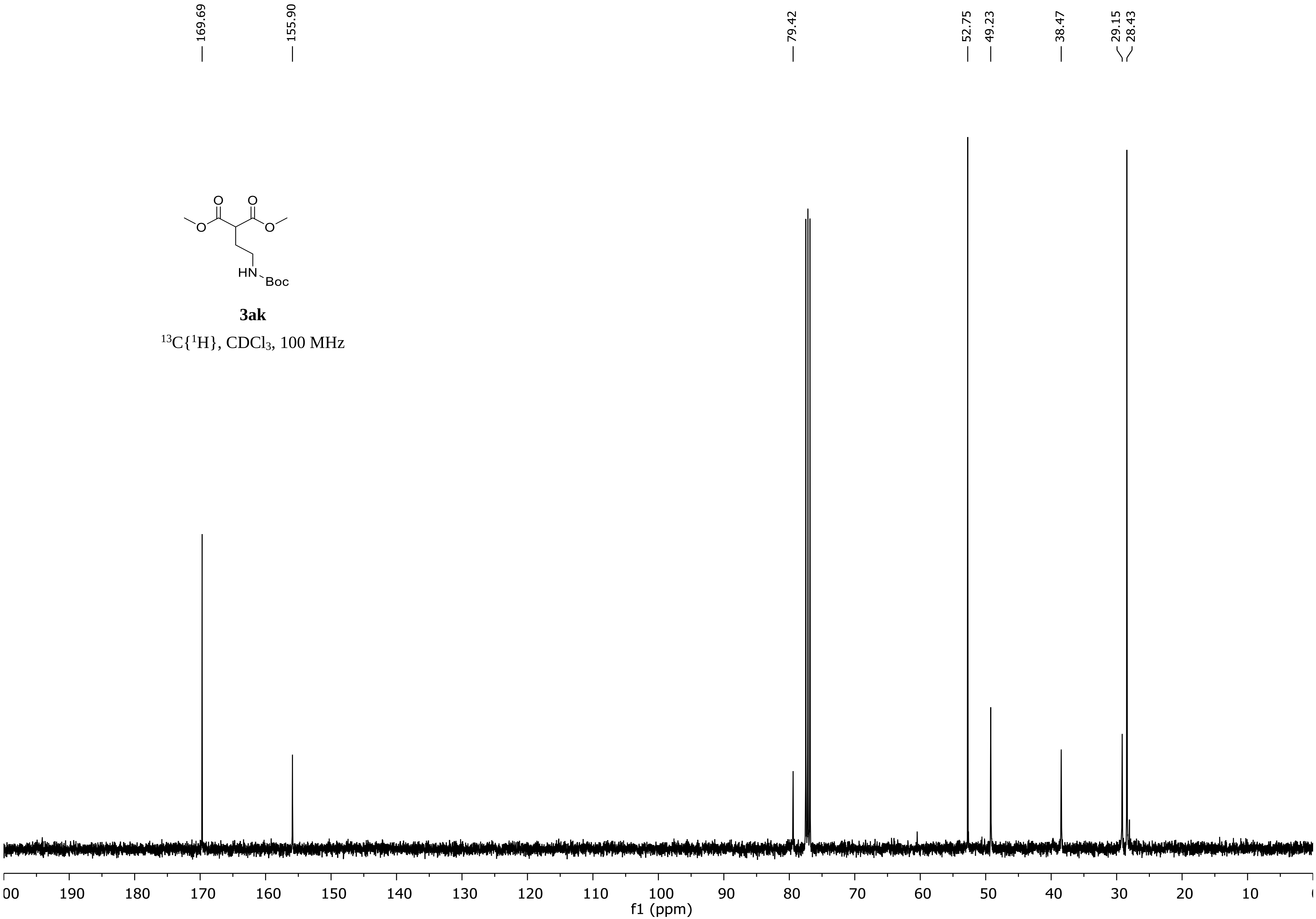

3ak

$^{13}C\{^1H\}$, $CDCl_3$, 100 MHz

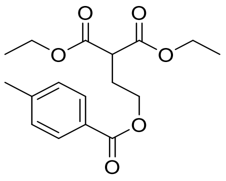

3bl

^{1}H, $CDCl_3$, 400 MHz

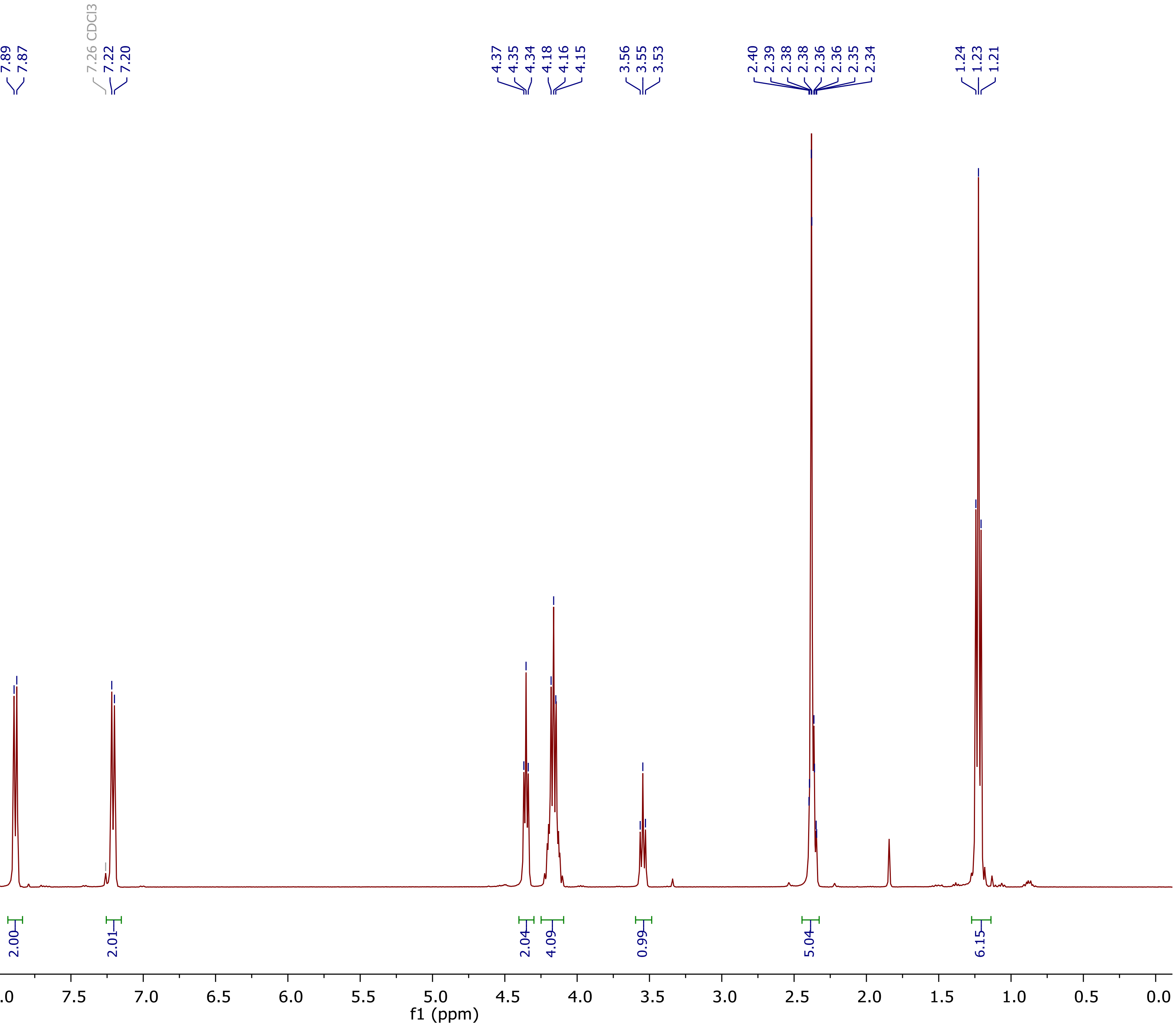

3bl

$^{13}C\{^1H\}$, $CDCl_3$, 100 MHz

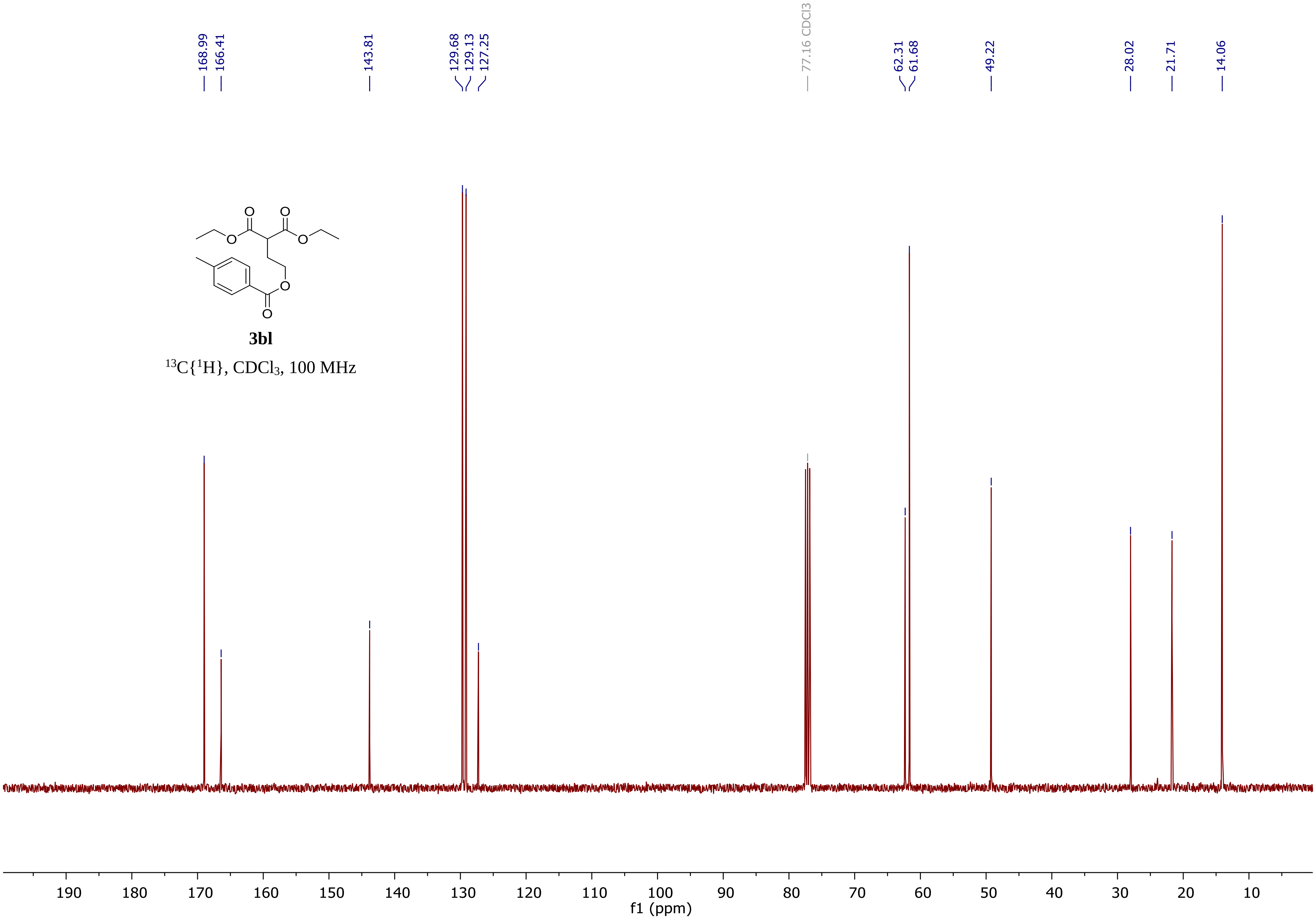

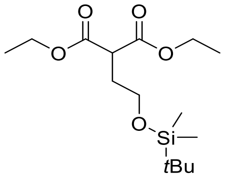

3bm

^{1}H, $CDCl_3$, 400 MHz

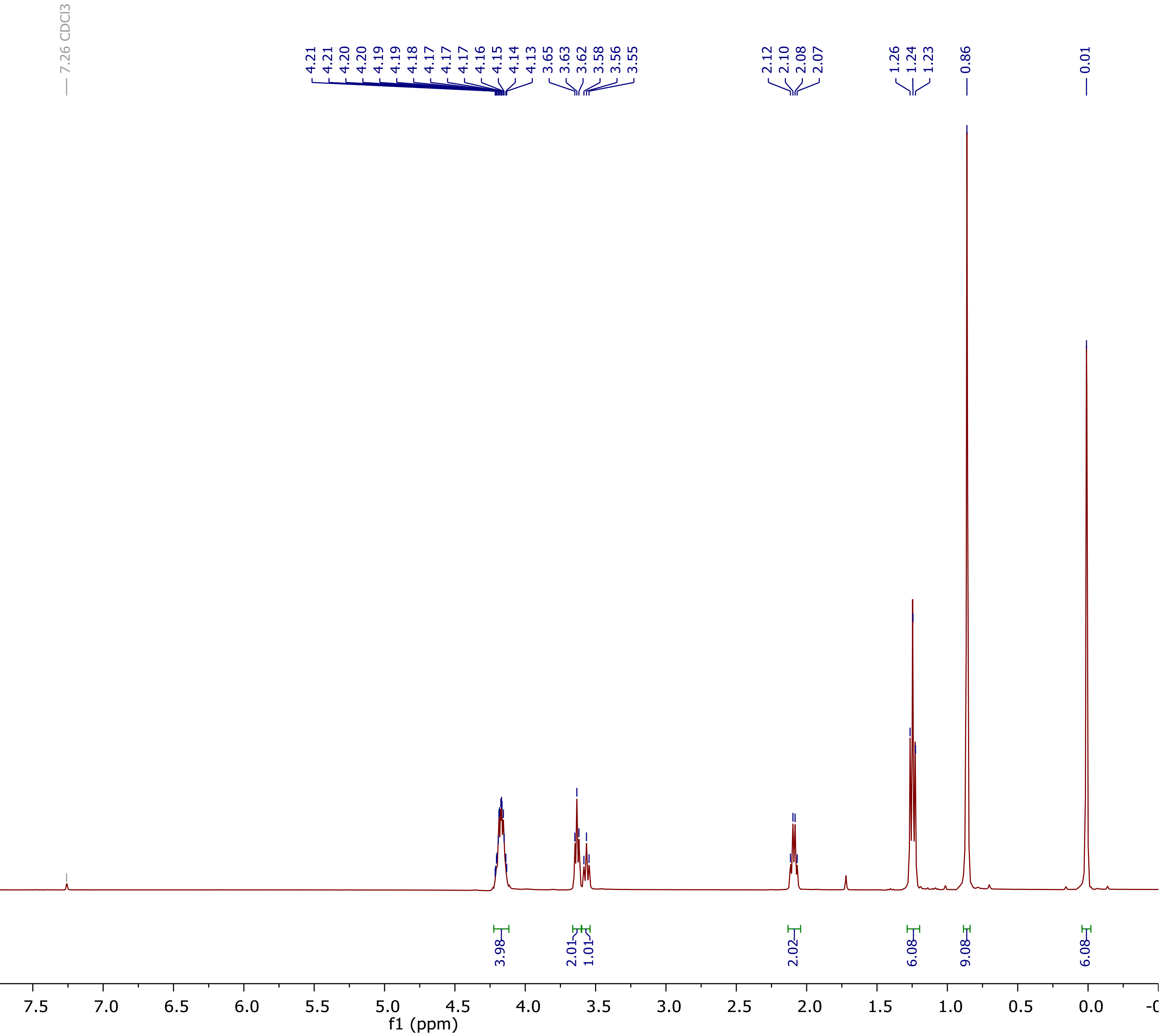

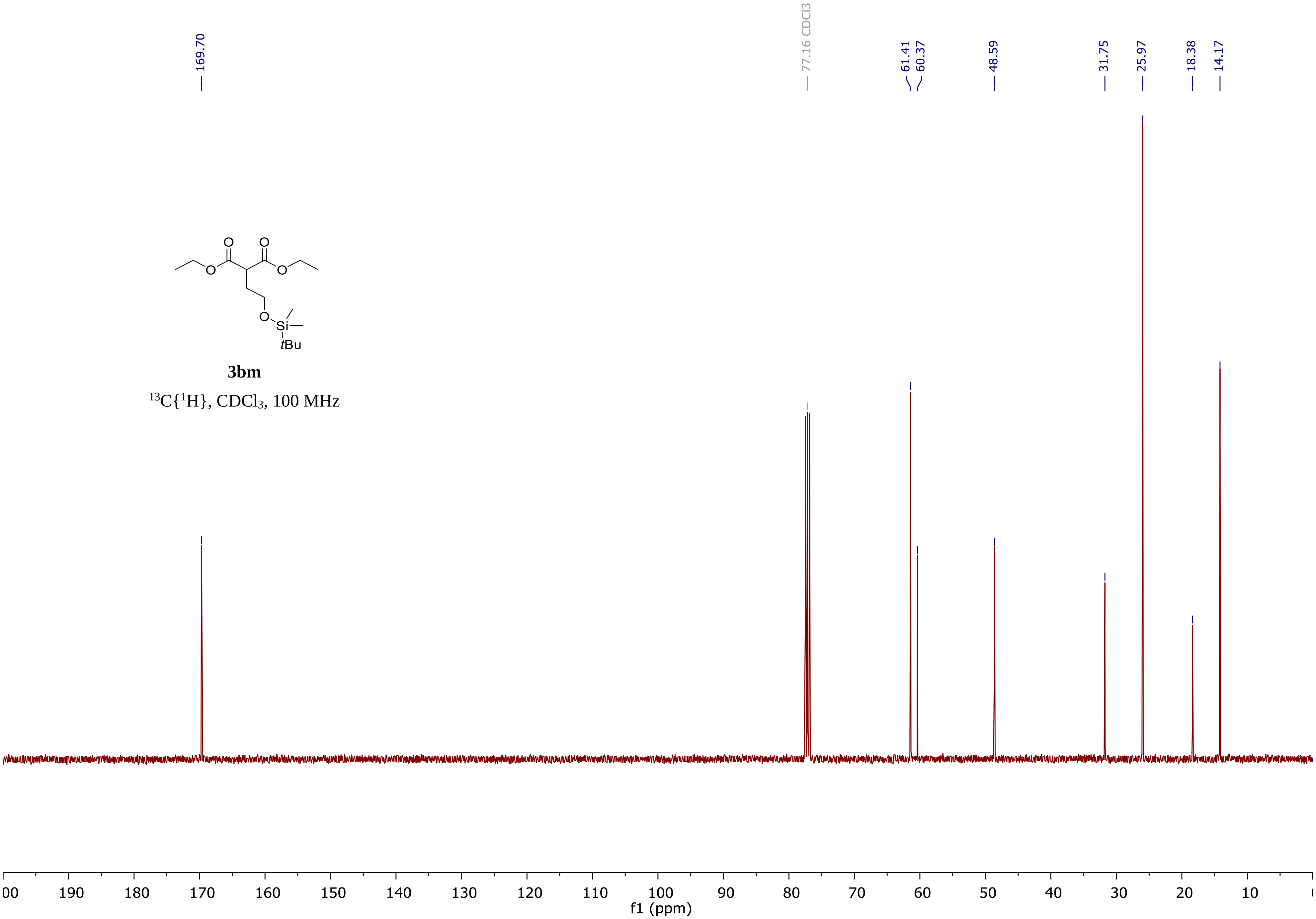

3bm

$^{13}C\{^1H\}$, $CDCl_3$, 100 MHz

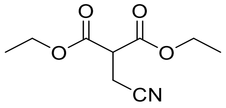

3bn

^{1}H, $CDCl_3$, 400 MHz

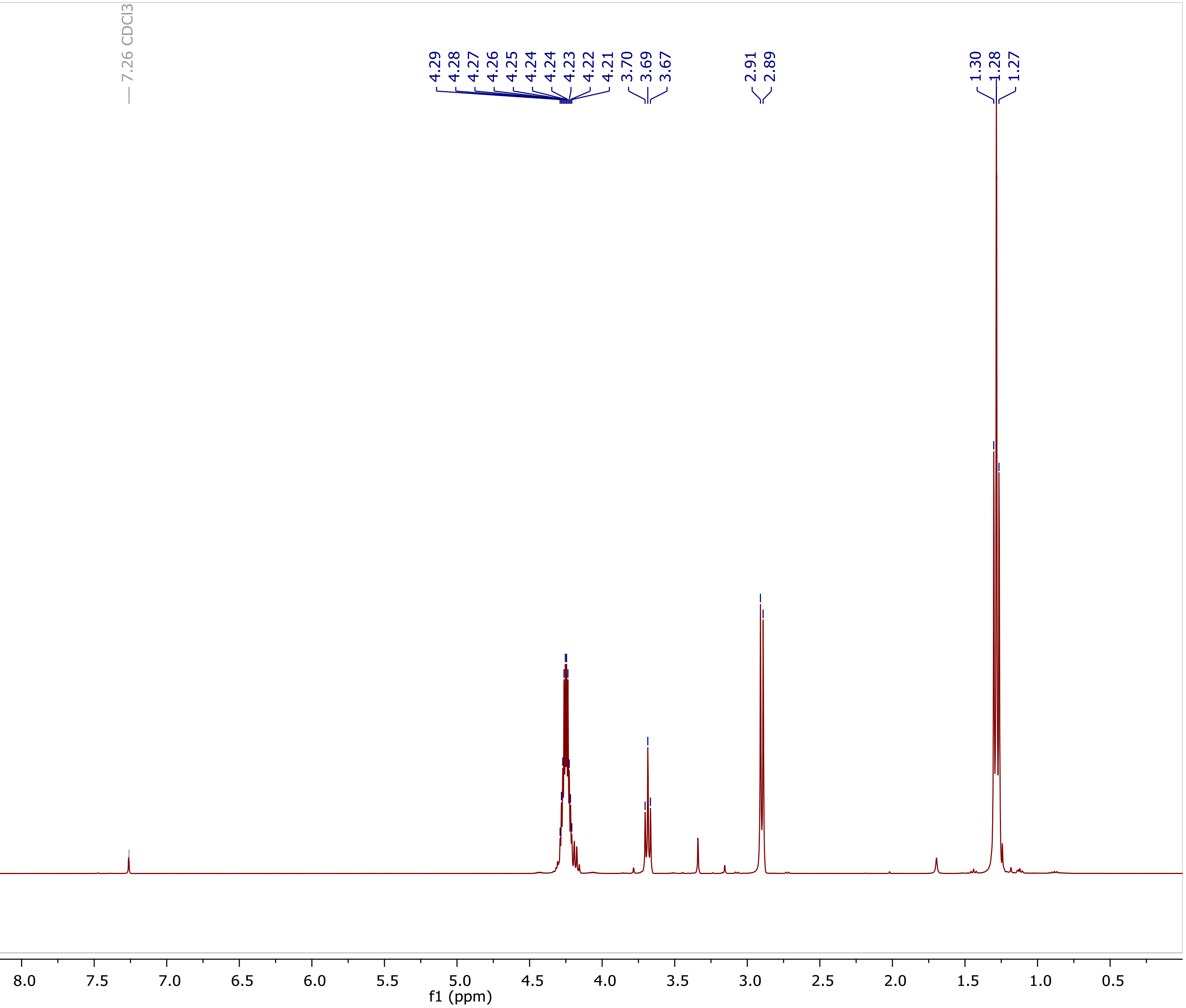

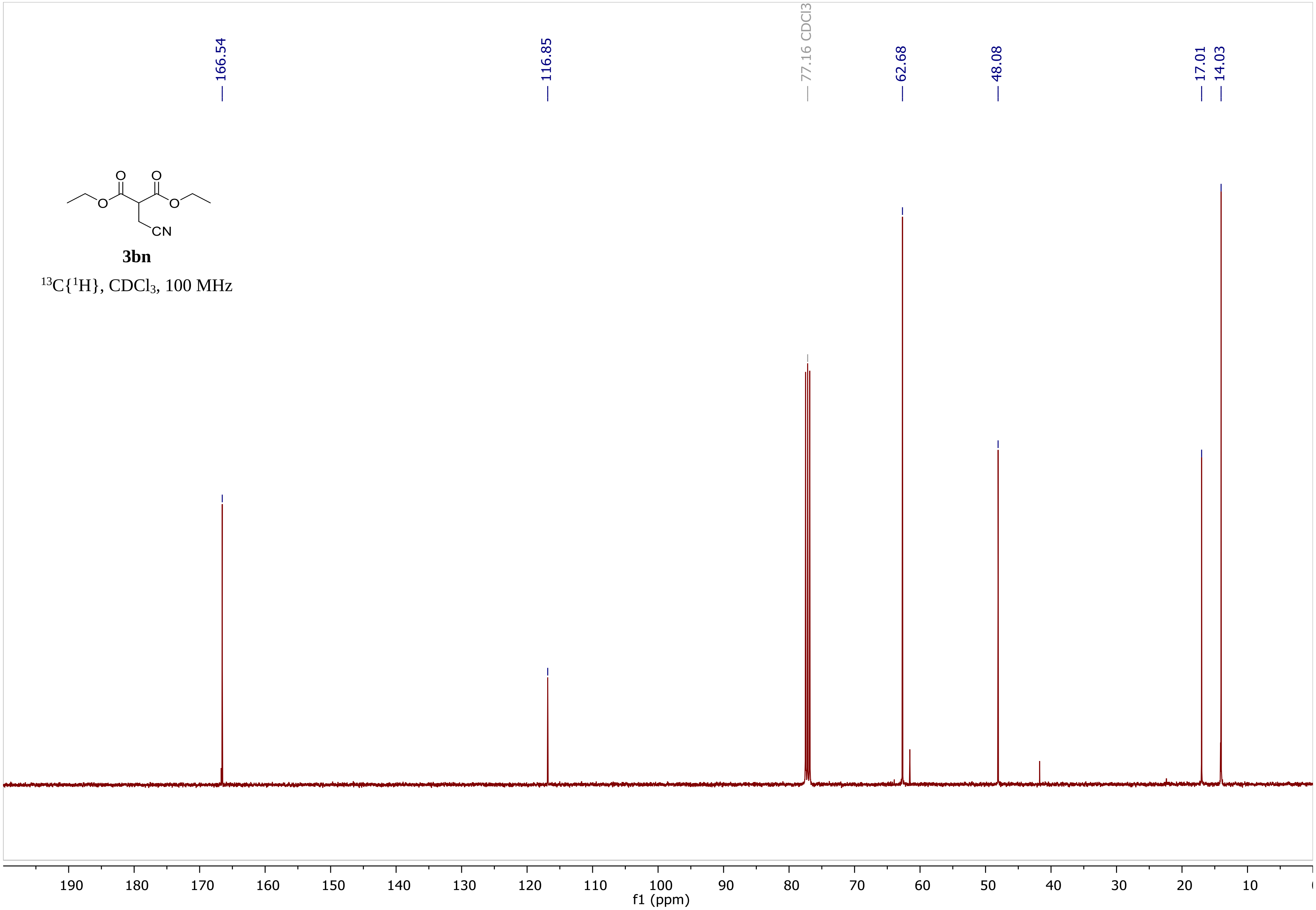

166.54
116.85
77.16 CDCl3
62.68
48.08
17.01
14.03
3bn
13C{1H}, CDCl3, 100 MHz
190
180
170
160
150
140
130
120
110
100
90
80
70
60
50
40
30
20
10
f1 (ppm)

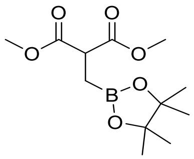

3ao

^{1}H, $CDCl_3$, 400 MHz

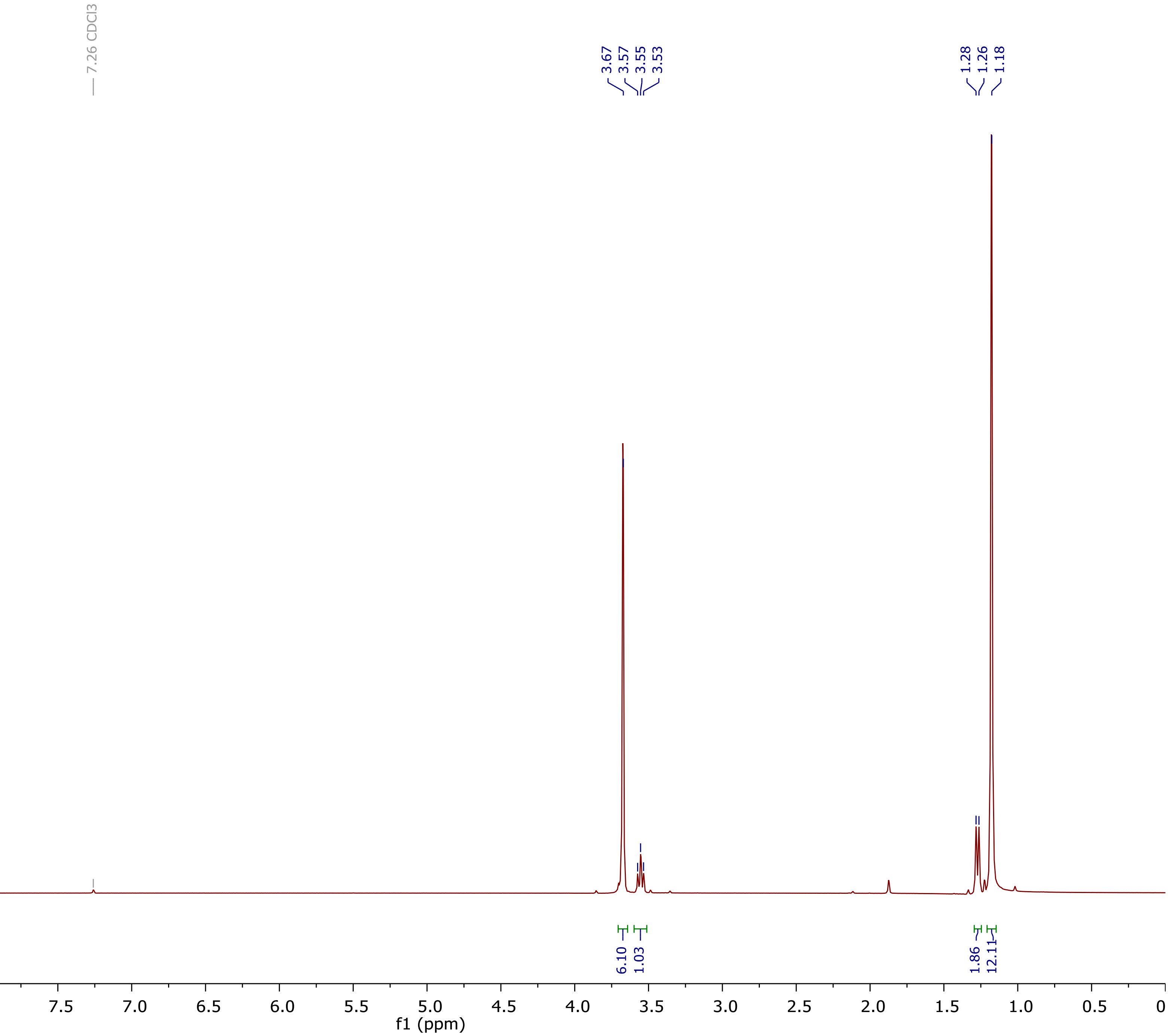

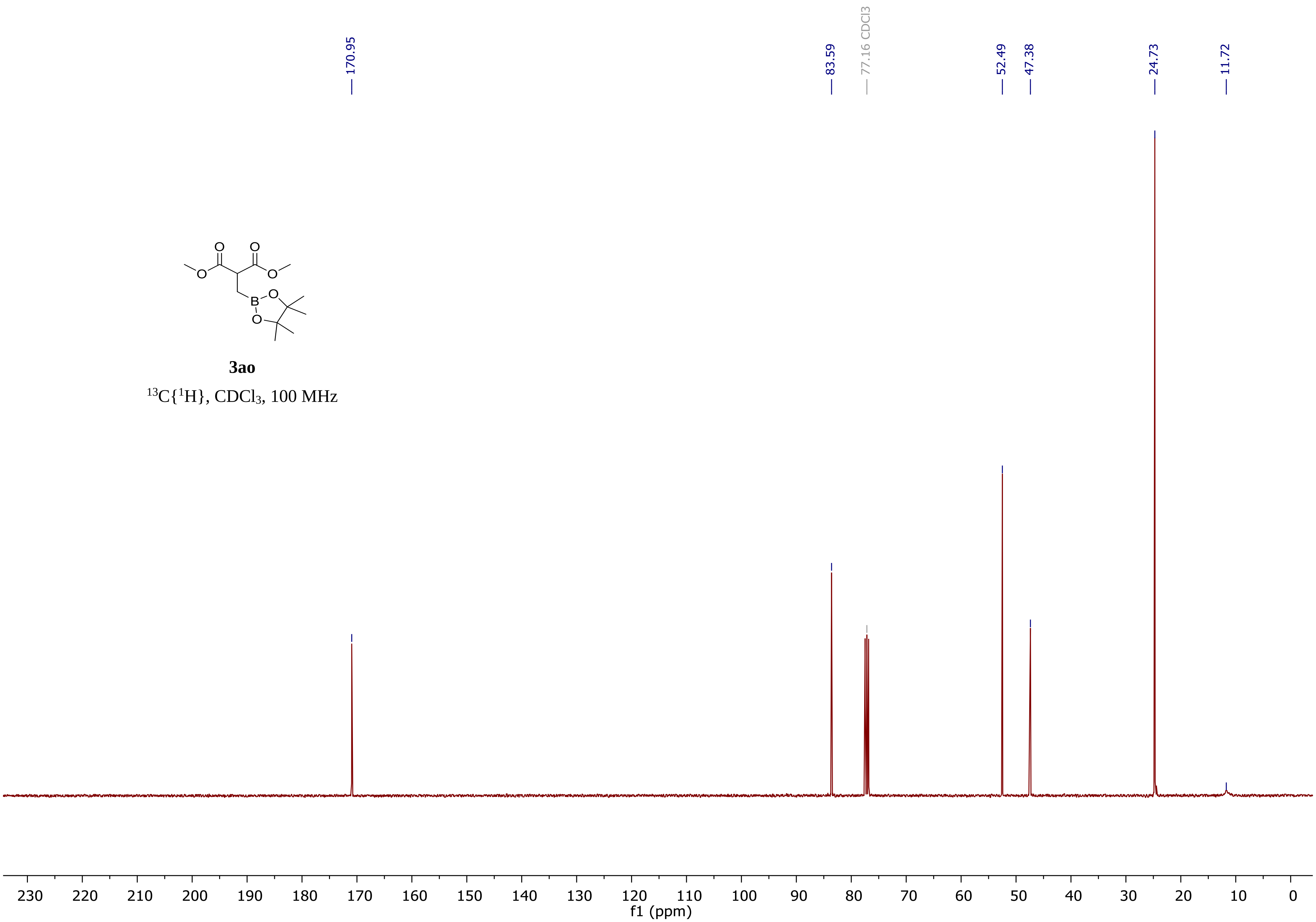

3ao

$^{13}C\{^1H\}$, $CDCl_3$, 100 MHz

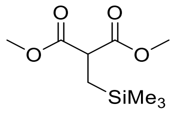

3ap

^{1}H, $CDCl_3$, 400 MHz

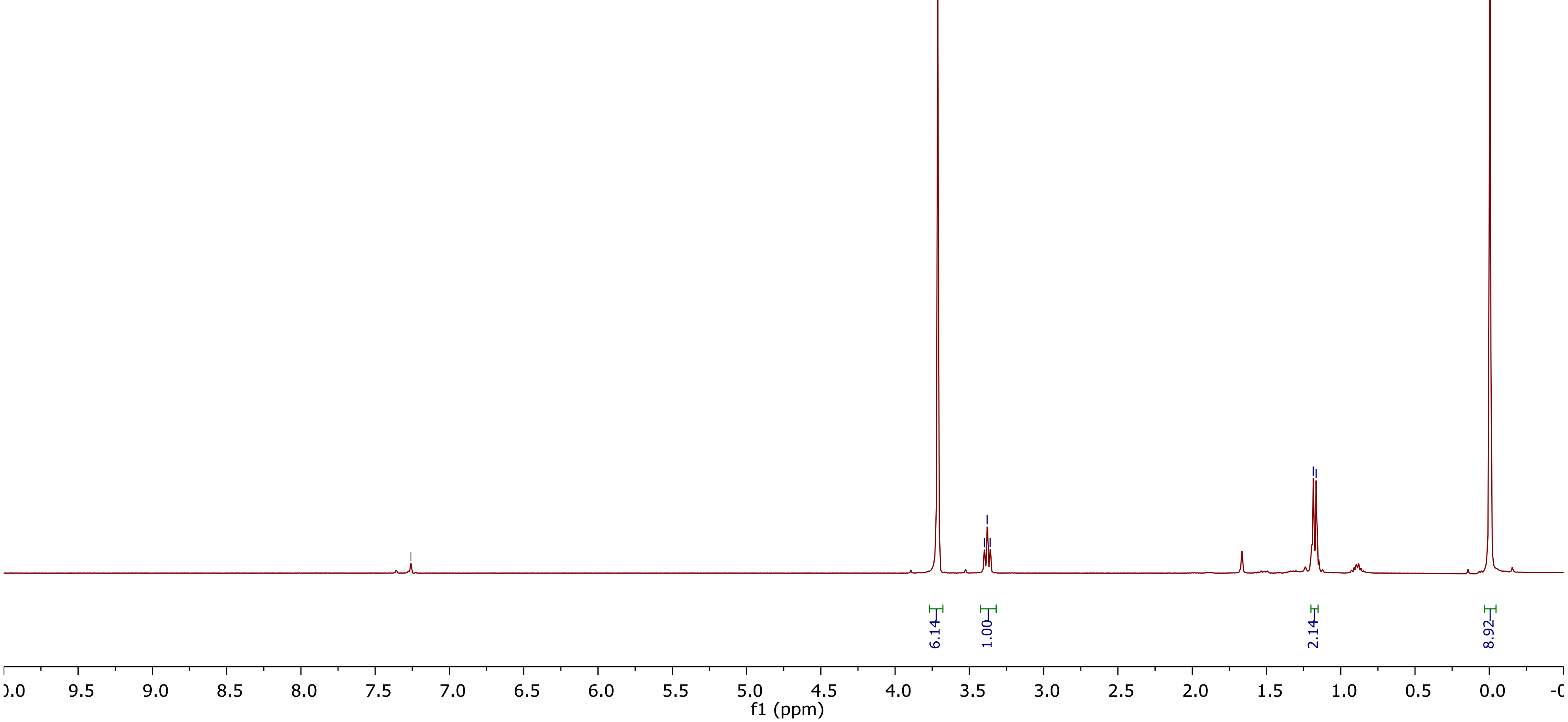

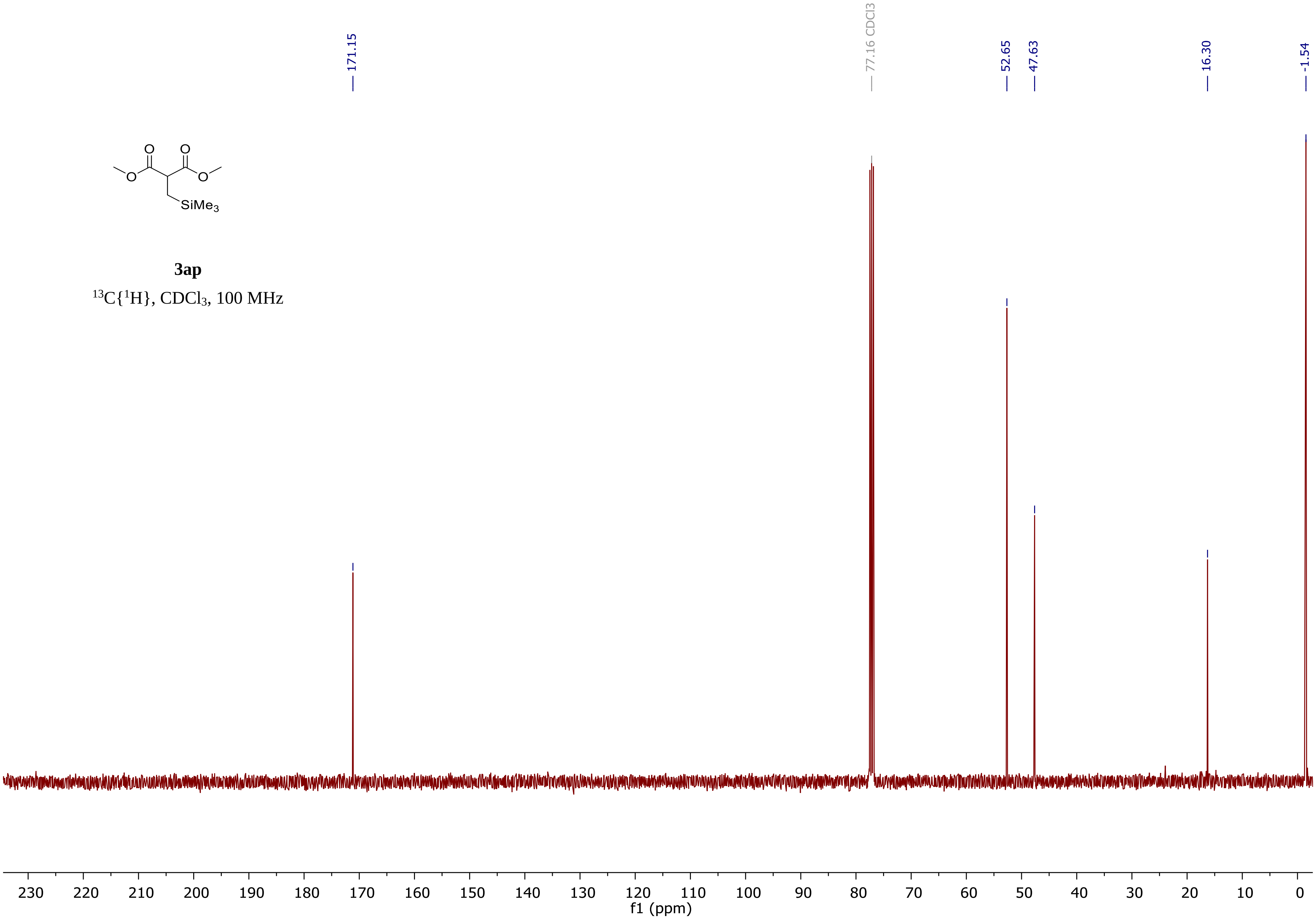

3ap

$^{13}C\{^1H\}$, $CDCl_3$, 100 MHz

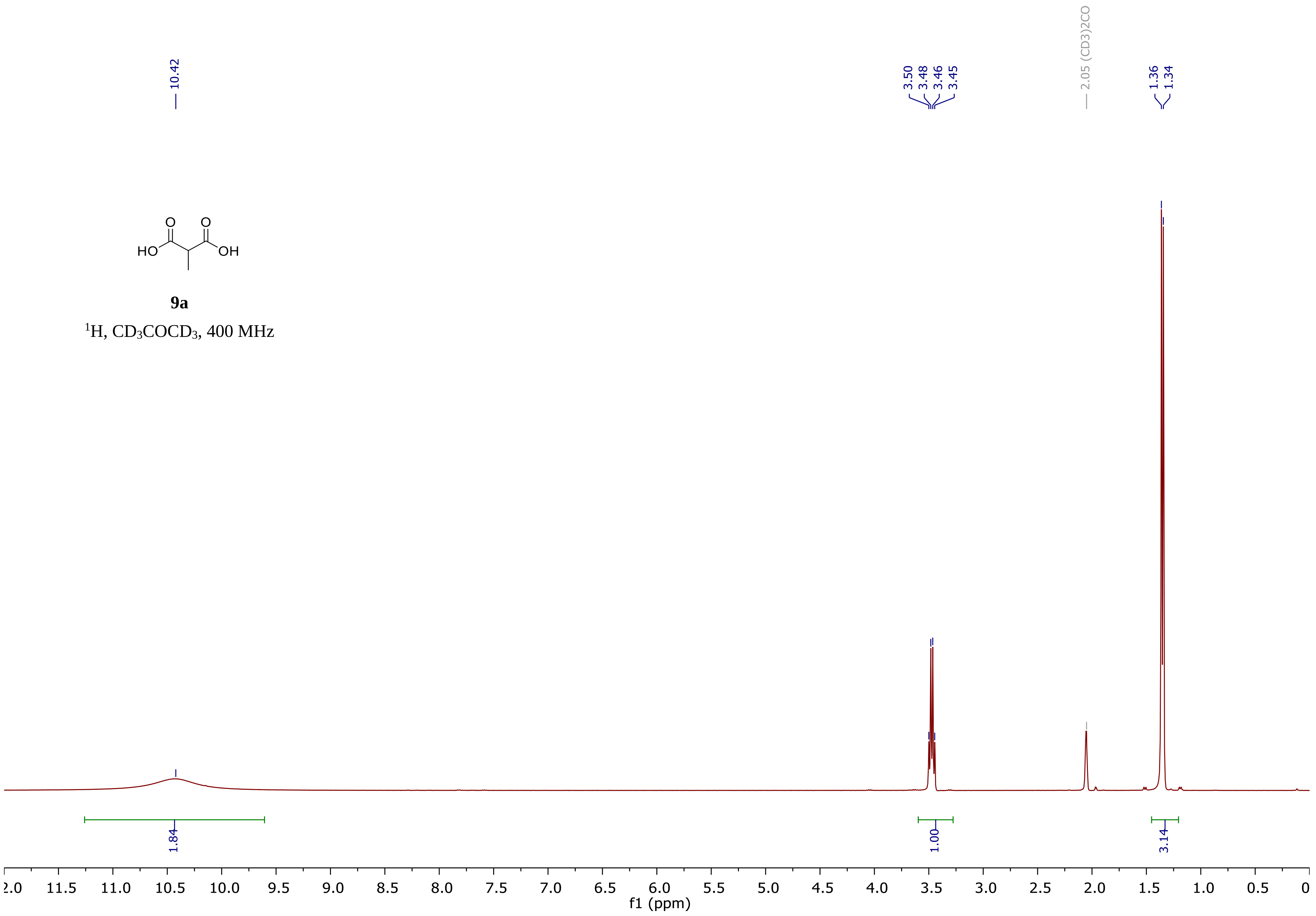

9a

^{1}H, CD_3COCD_3, 400 MHz

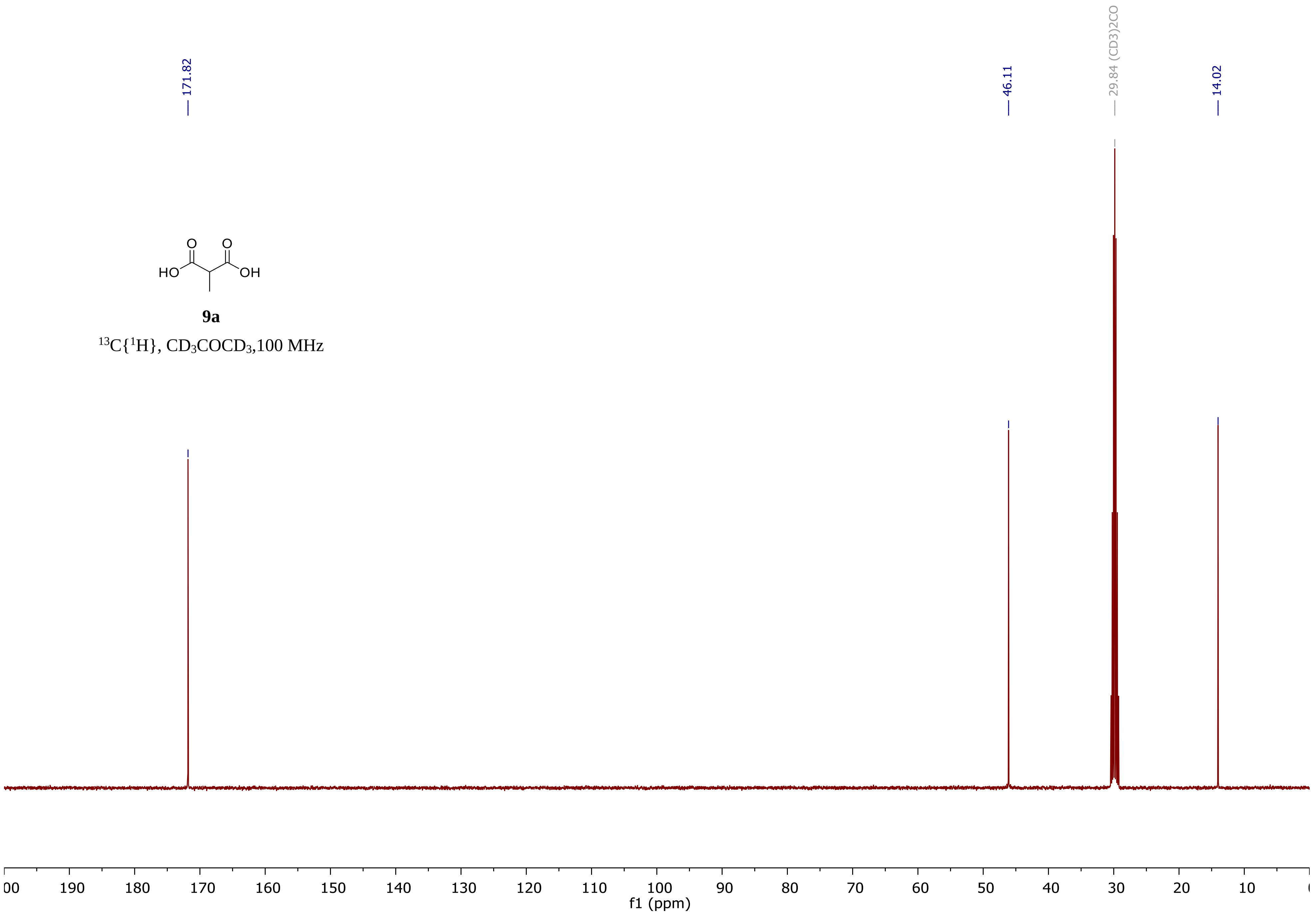

171.82
46.11
29.84 (CD3)2CO
14.02
HO
OH
O
O
9a
13C{1H}, CD3COCD3,100 MHz
00 190 180 170 160 150 140 130 120 110 100 90 80 70 60 50 40 30 20 10
f1 (ppm)

9c

^{1}H, CD_3SOCD_3, 400 MHz

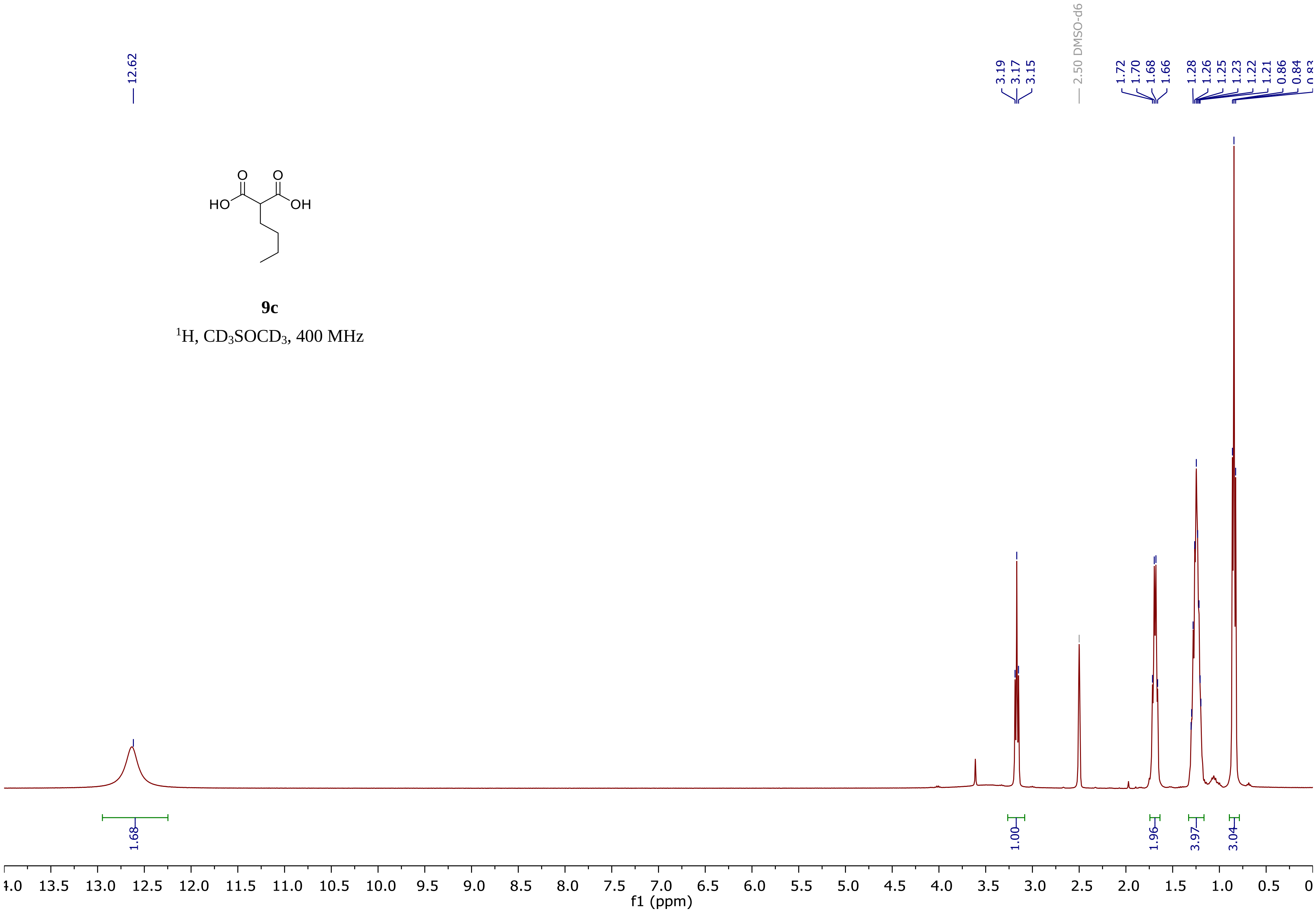

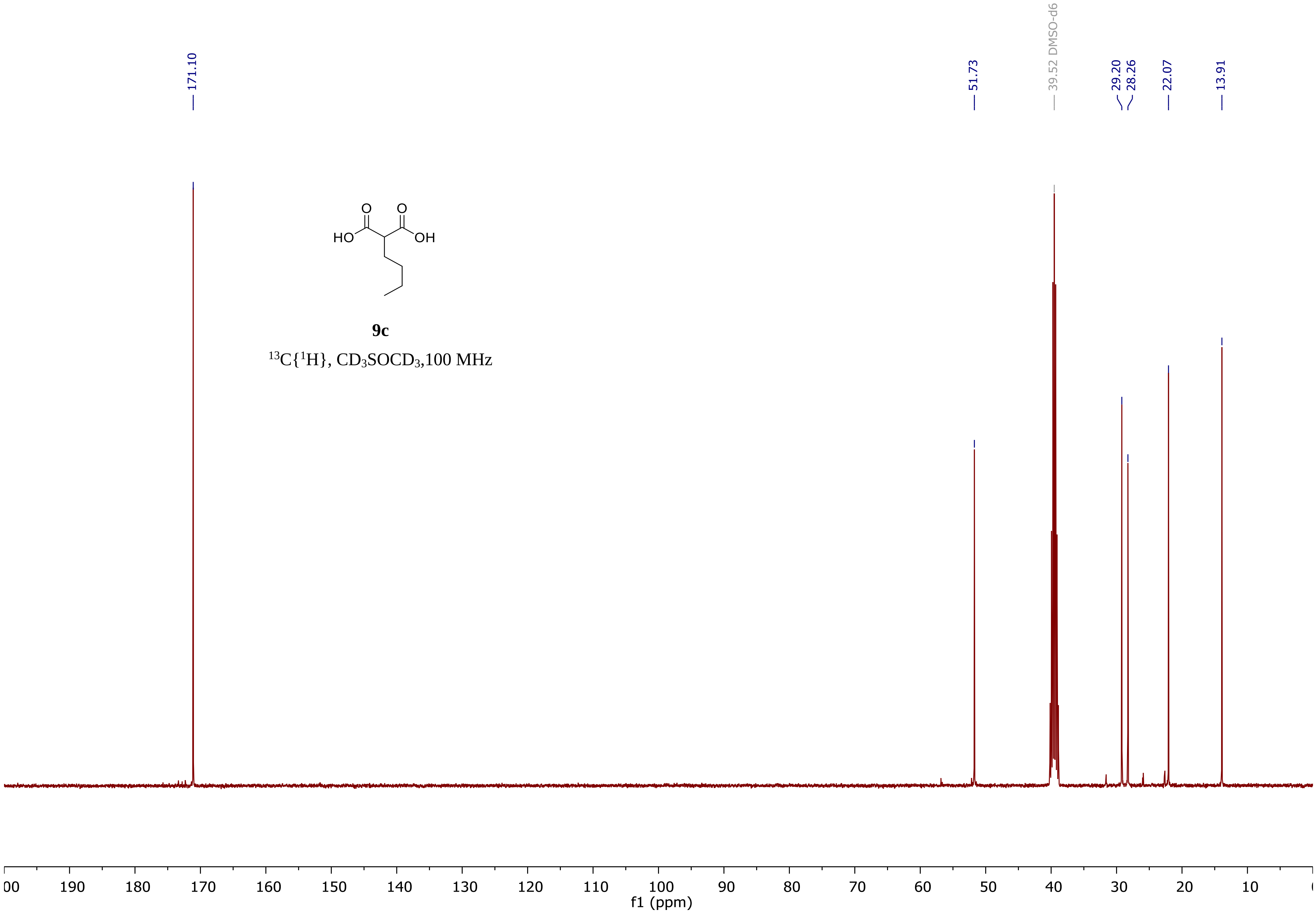
171.10
51.73
39.52 DMSO-d6
29.20
28.26
22.07
13.91
HO
O
O
OH
9c
13C{1H}, CD3SOCD3,100 MHz
00
190
180
170
160
150
140
130
120
110
100
90
80
70
60
50
40
30
20
10
f1 (ppm)

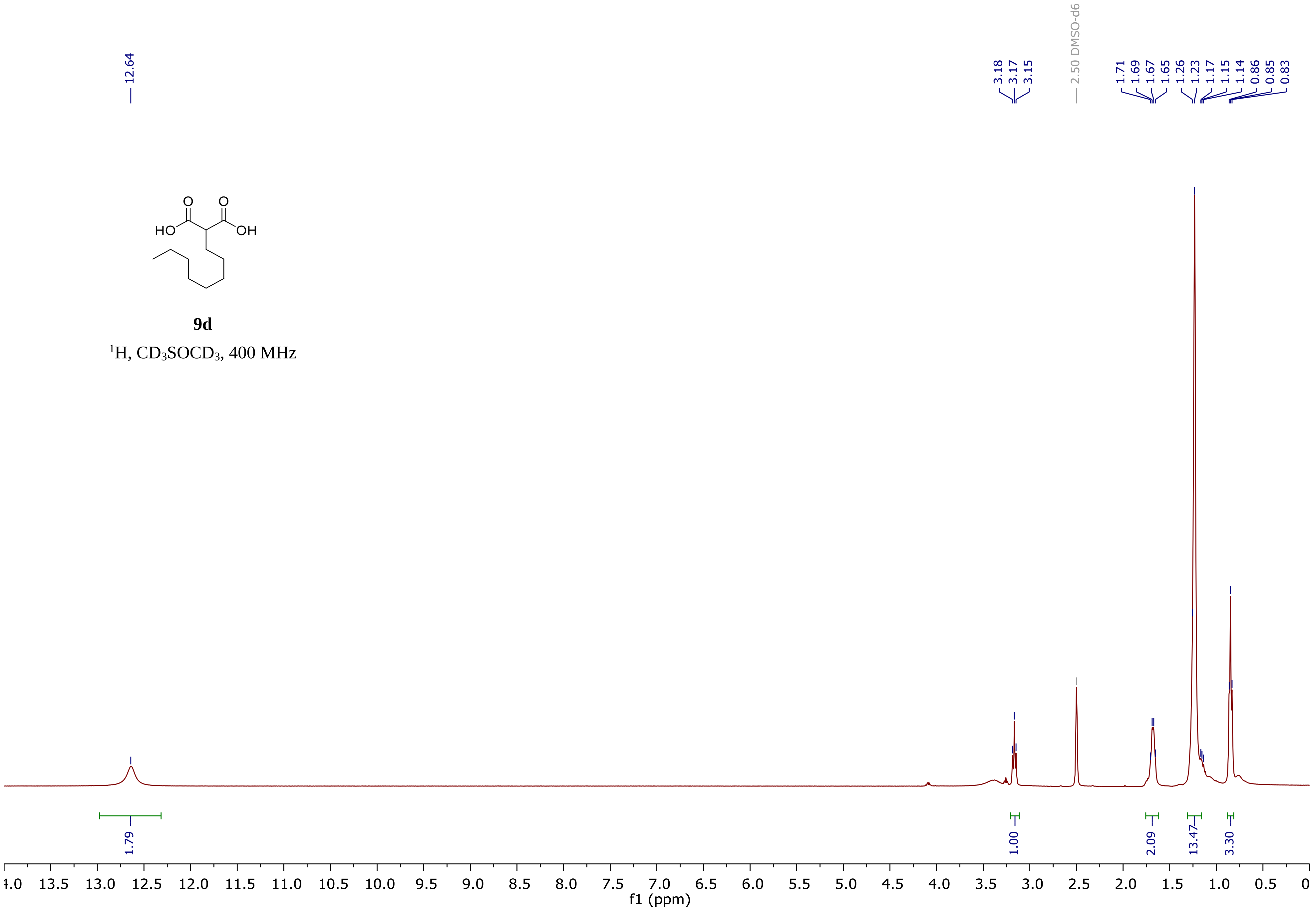

9d

^{1}H, CD_3SOCD_3, 400 MHz

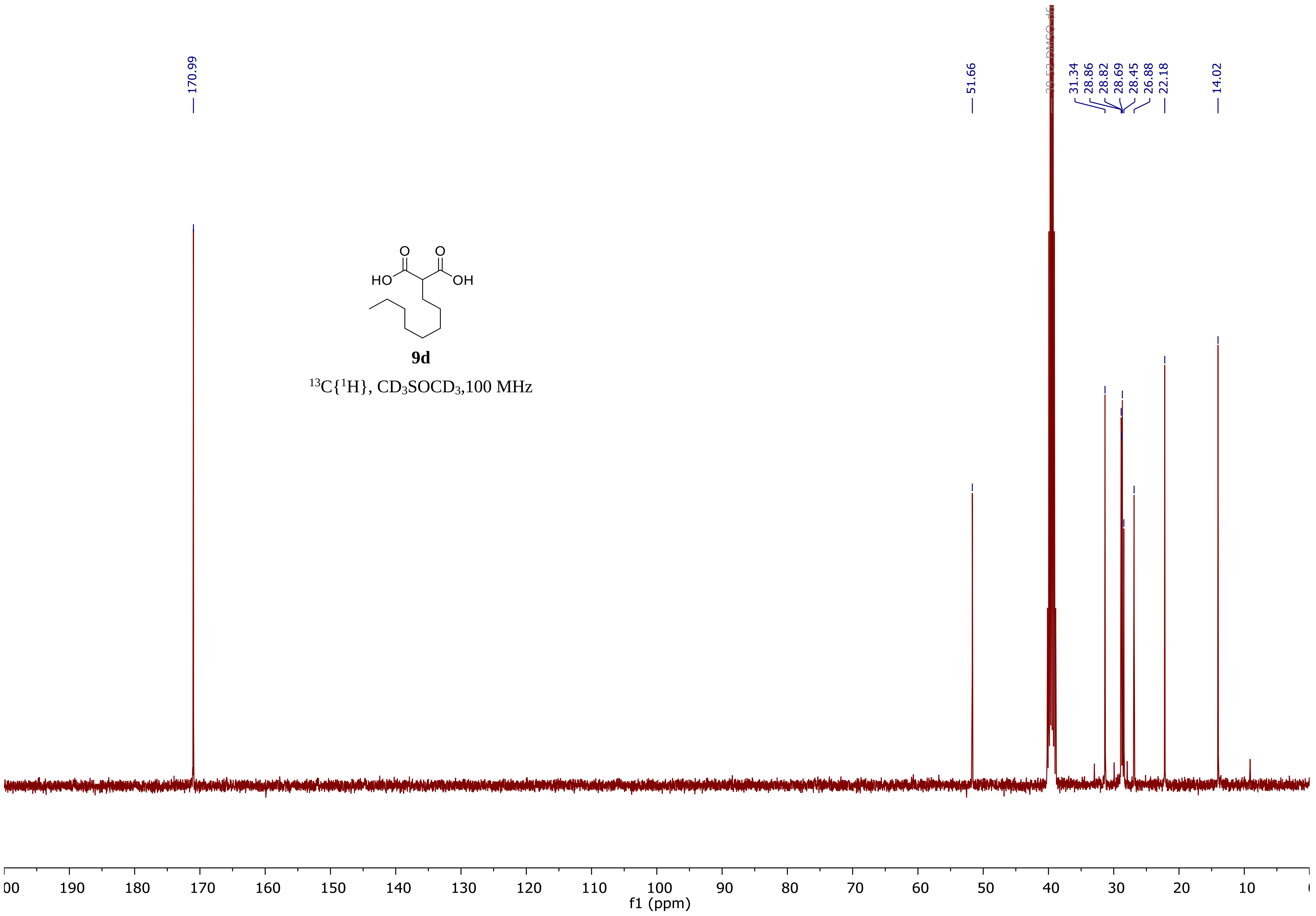
170.99
51.66
39.52 DMSO-d6
31.34
28.86
28.82
28.69
28.45
26.88
22.18
14.02
HO
OH
O
O
9d
$^{13}C\{^{1}H\}$, CD_3SOCD_3, 100 MHz
00
190
180
170
160
150
140
130
120
110
100
90
80
70
60
50
40
30
20
10
f1 (ppm)

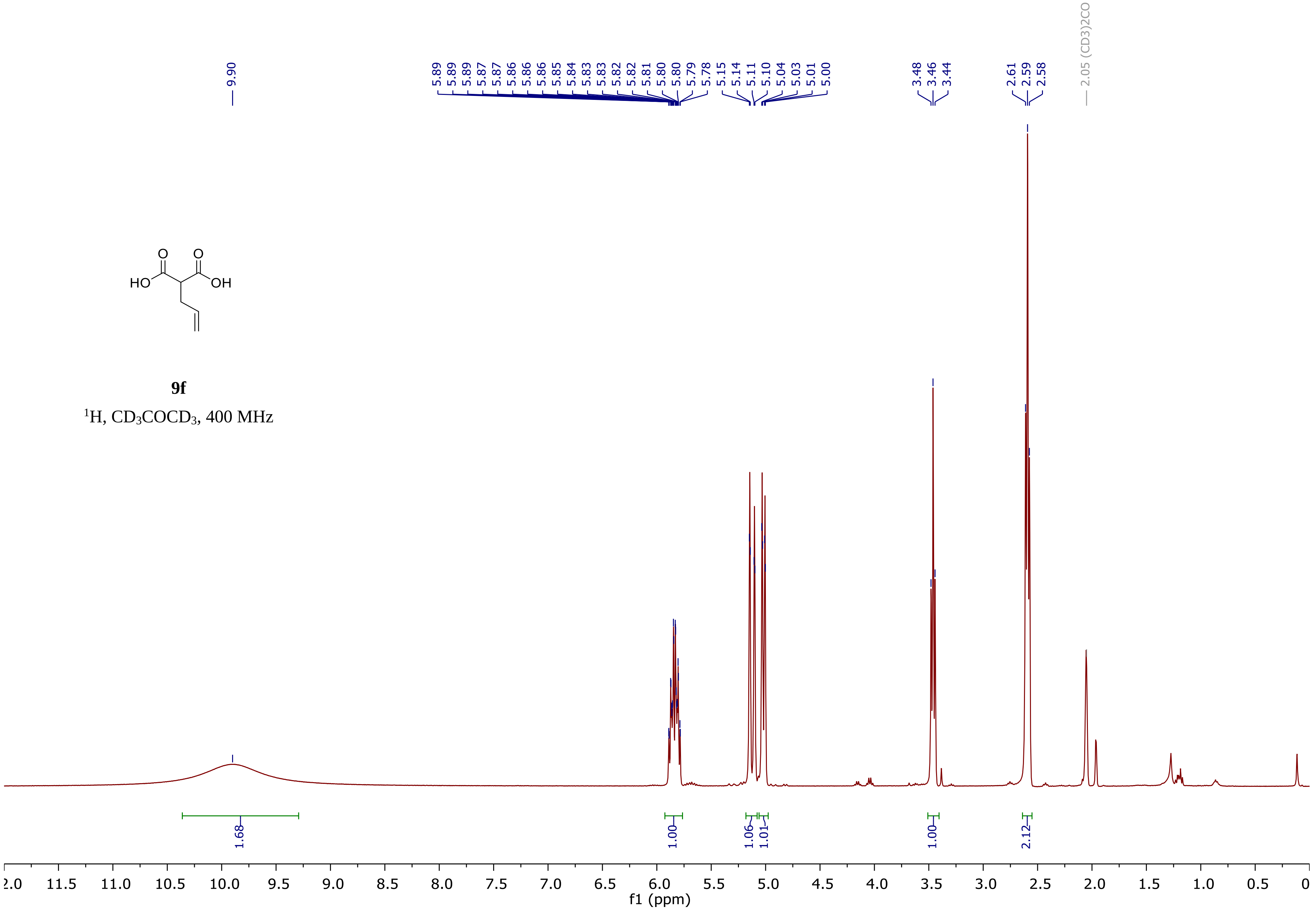

9f

^{1}H, CD_3COCD_3, 400 MHz

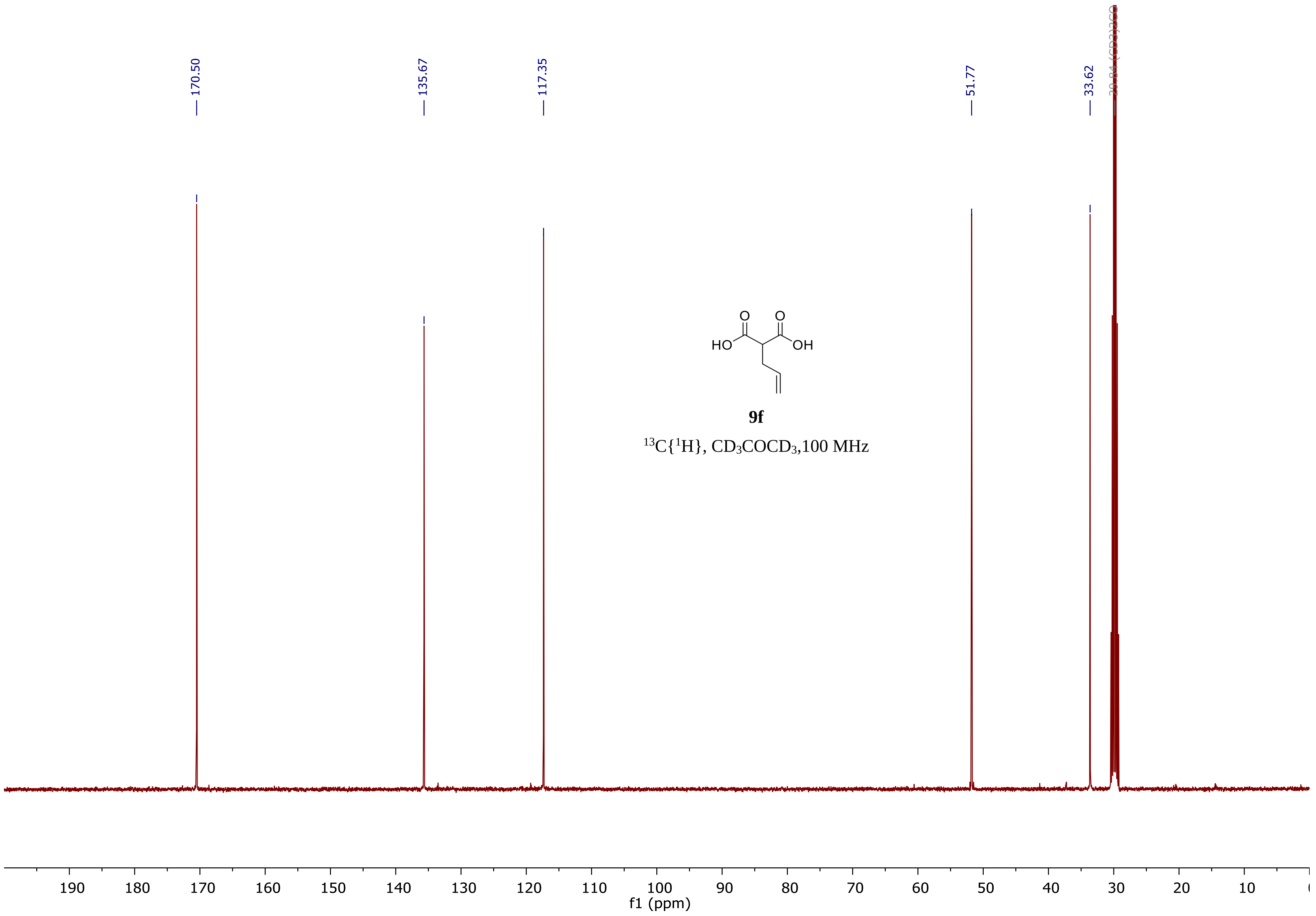
170.50
135.67
117.35
51.77
33.62
HO
OH
O
O
9f
13C{1H}, CD3COCD3,100 MHz
190
180
170
160
150
140
130
120
110
100
90
80
70
60
50
40
30
20
10
f1 (ppm)

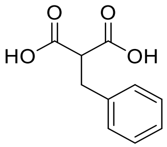

9h

^1H, CD_3COCD_3, 400 MHz

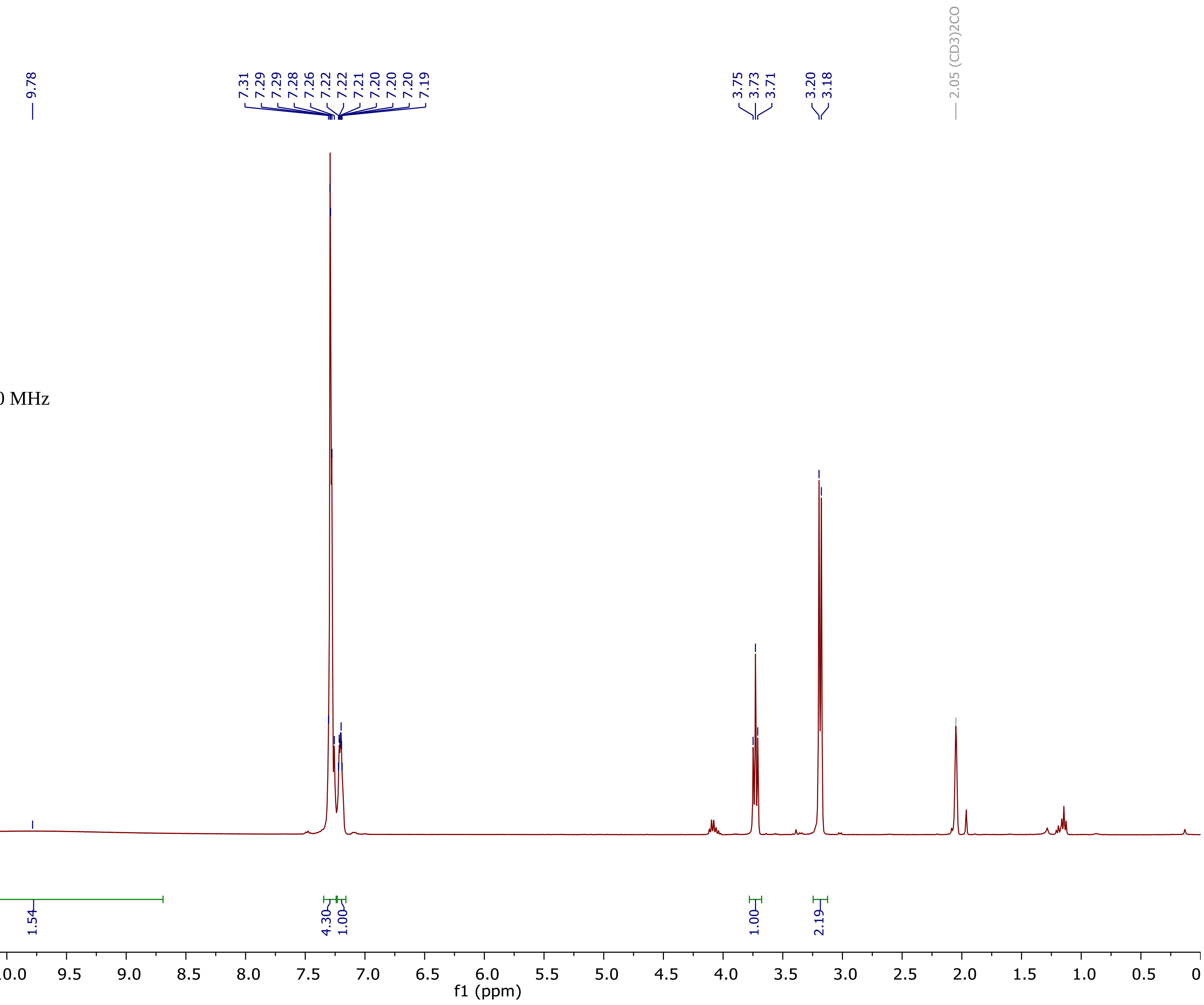

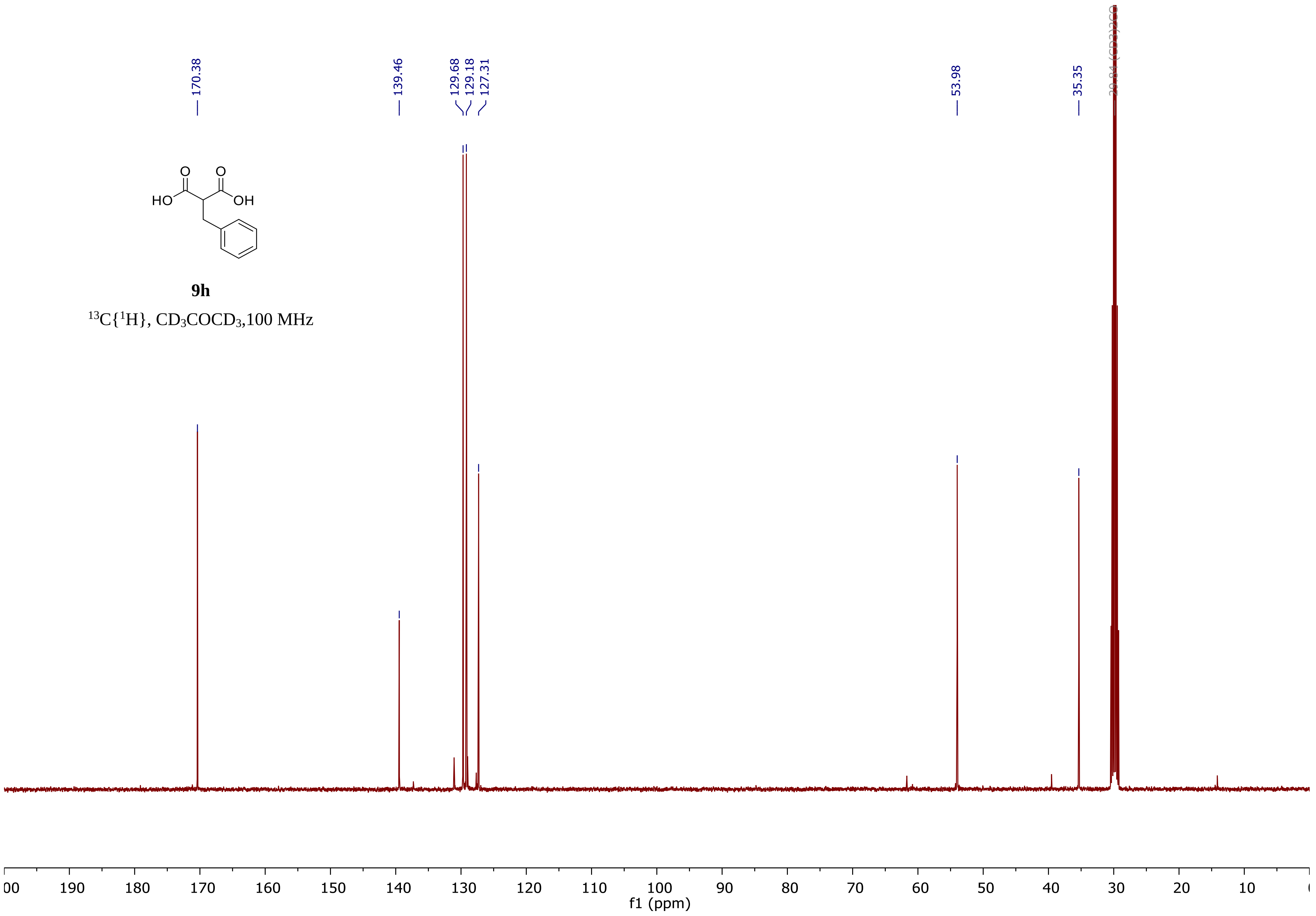
170.38
139.46
129.68
129.18
127.31
53.98
35.35
29.84 (CD3)2CO
HO
OH
O
O
9h
$^{13}C\{^{1}H\}$, CD_3COCD_3,100 MHz
f1 (ppm)

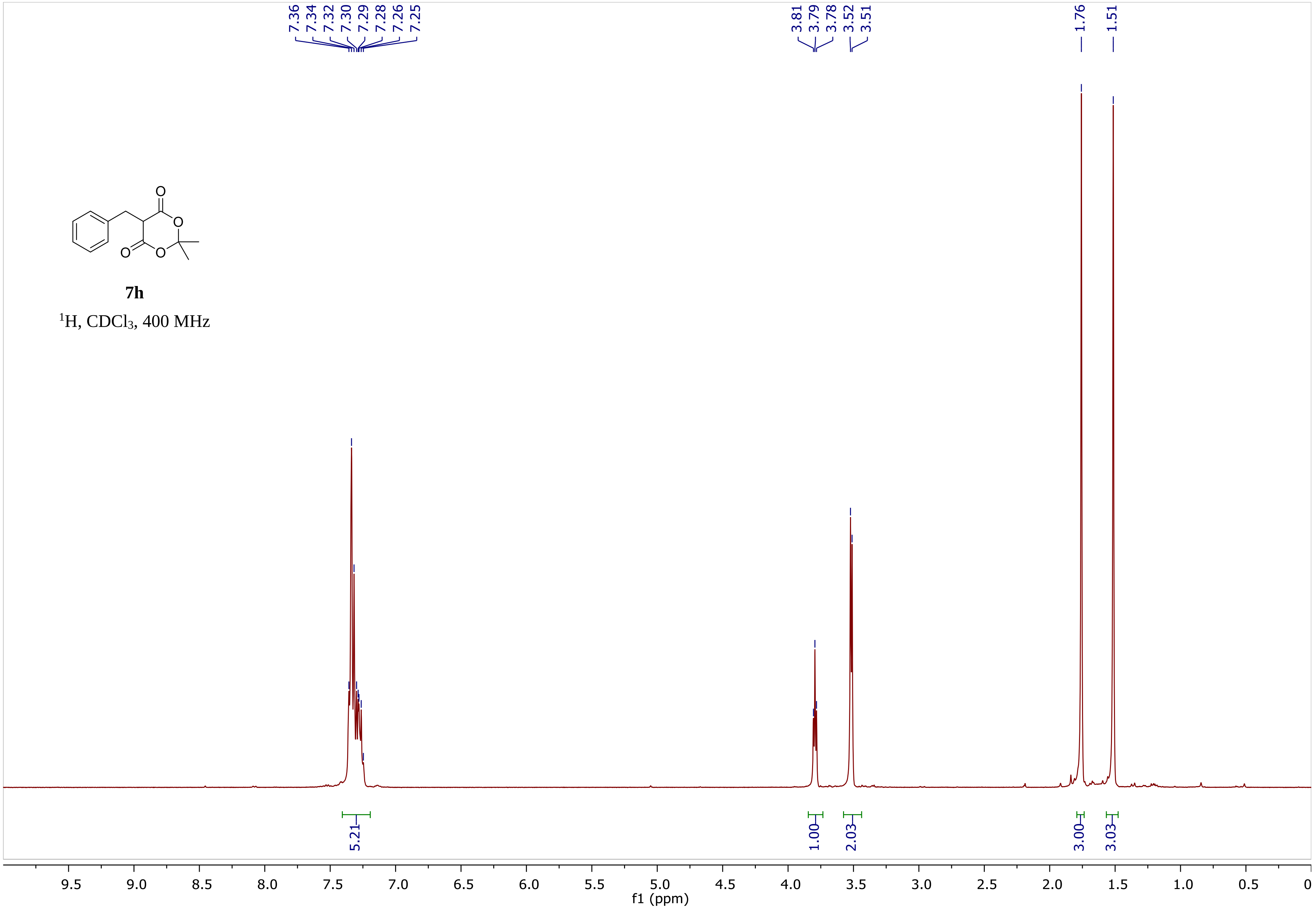
7.36
7.34
7.32
7.30
7.29
7.28
7.26
7.25
3.81
3.79
3.78
3.52
3.51
1.76
1.51
7h
^1H, $CDCl_3$, 400 MHz
5.21
1.00
2.03
3.00
3.03
9.5
9.0
8.5
8.0
7.5
7.0
6.5
6.0
5.5
5.0
4.5
4.0
3.5
3.0
2.5
2.0
1.5
1.0
0.5
0
f1 (ppm)

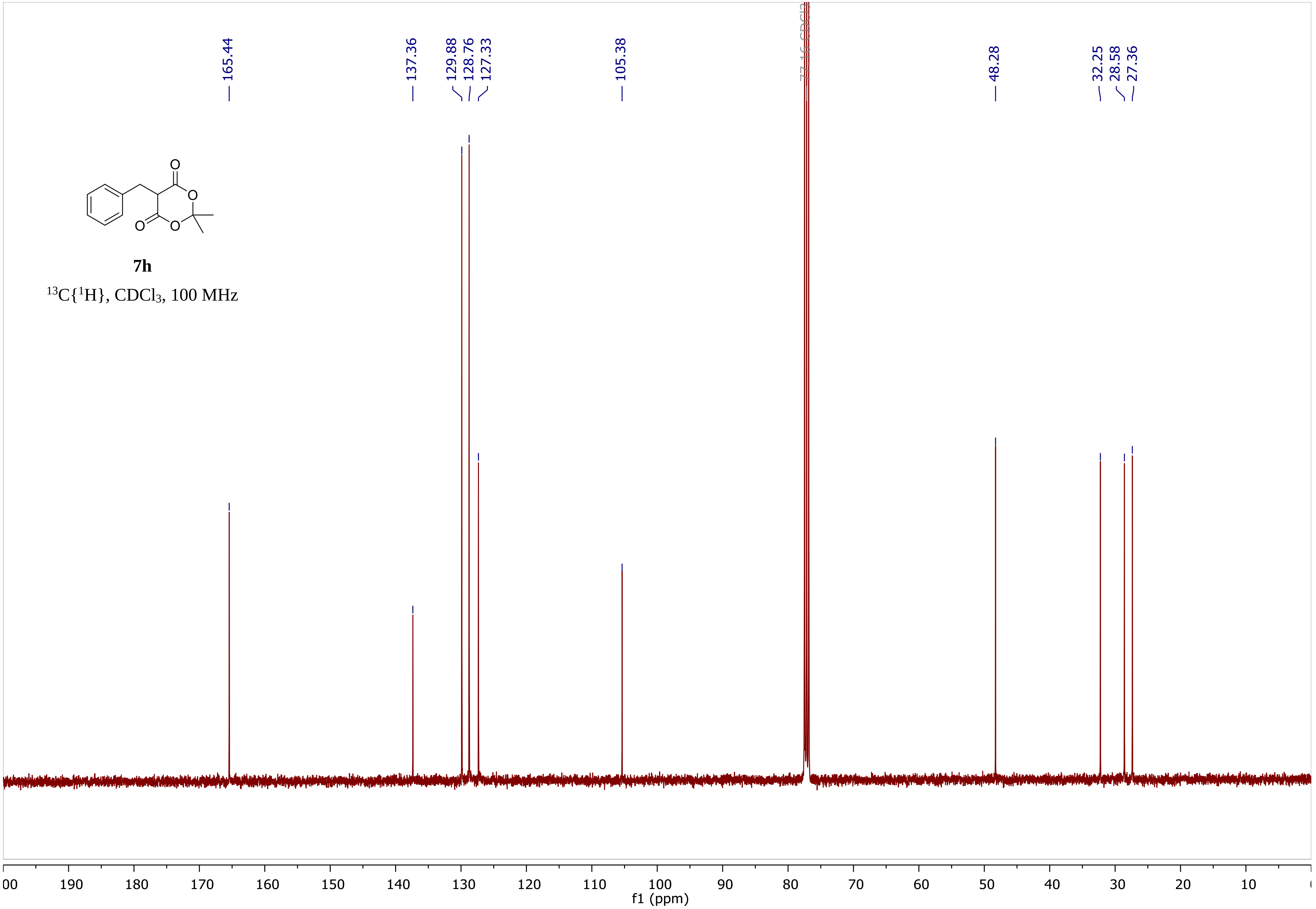

165.44
137.36
129.88
128.76
127.33
105.38
77.16 CDCl3
48.28
32.25
28.58
27.36
7h
$^{13}C\{^{1}H\}$, $CDCl_3$, 100 MHz
f1 (ppm)

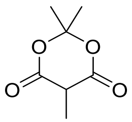

7a

^{1}H, $CDCl_3$, 400 MHz

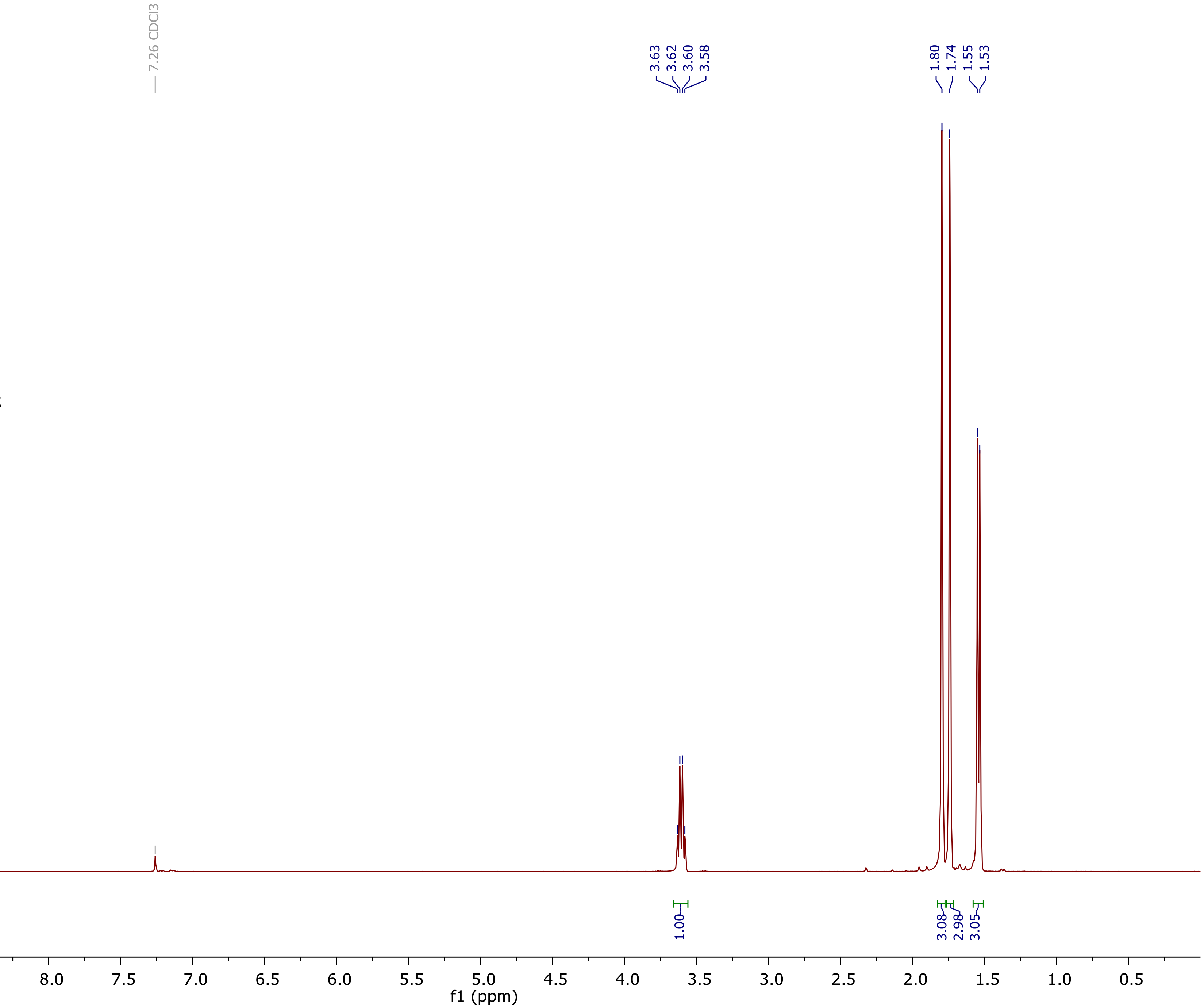

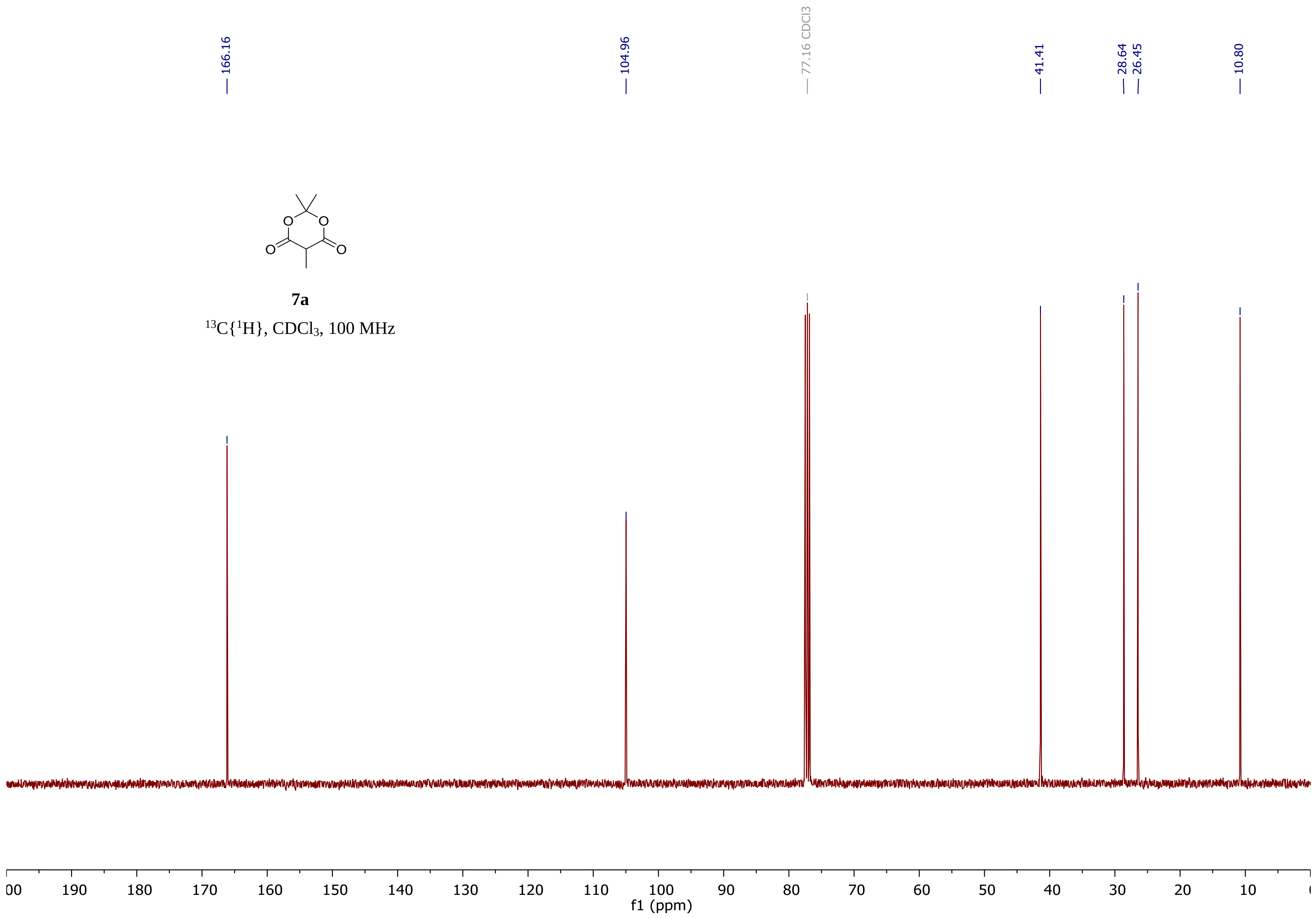

7a

$^{13}C\{^{1}H\}$, $CDCl_3$, 100 MHz

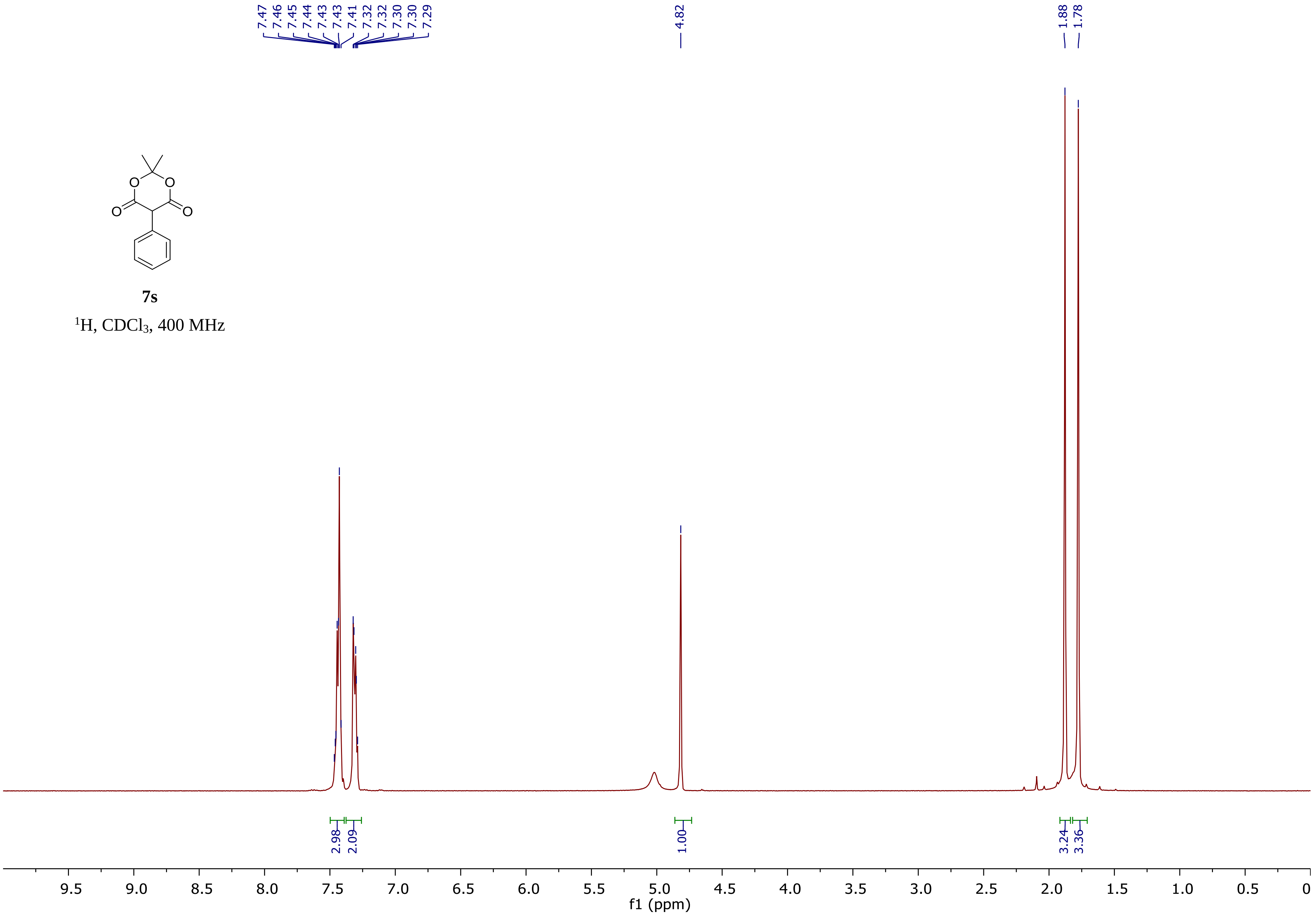

7s

^{1}H, $CDCl_3$, 400 MHz

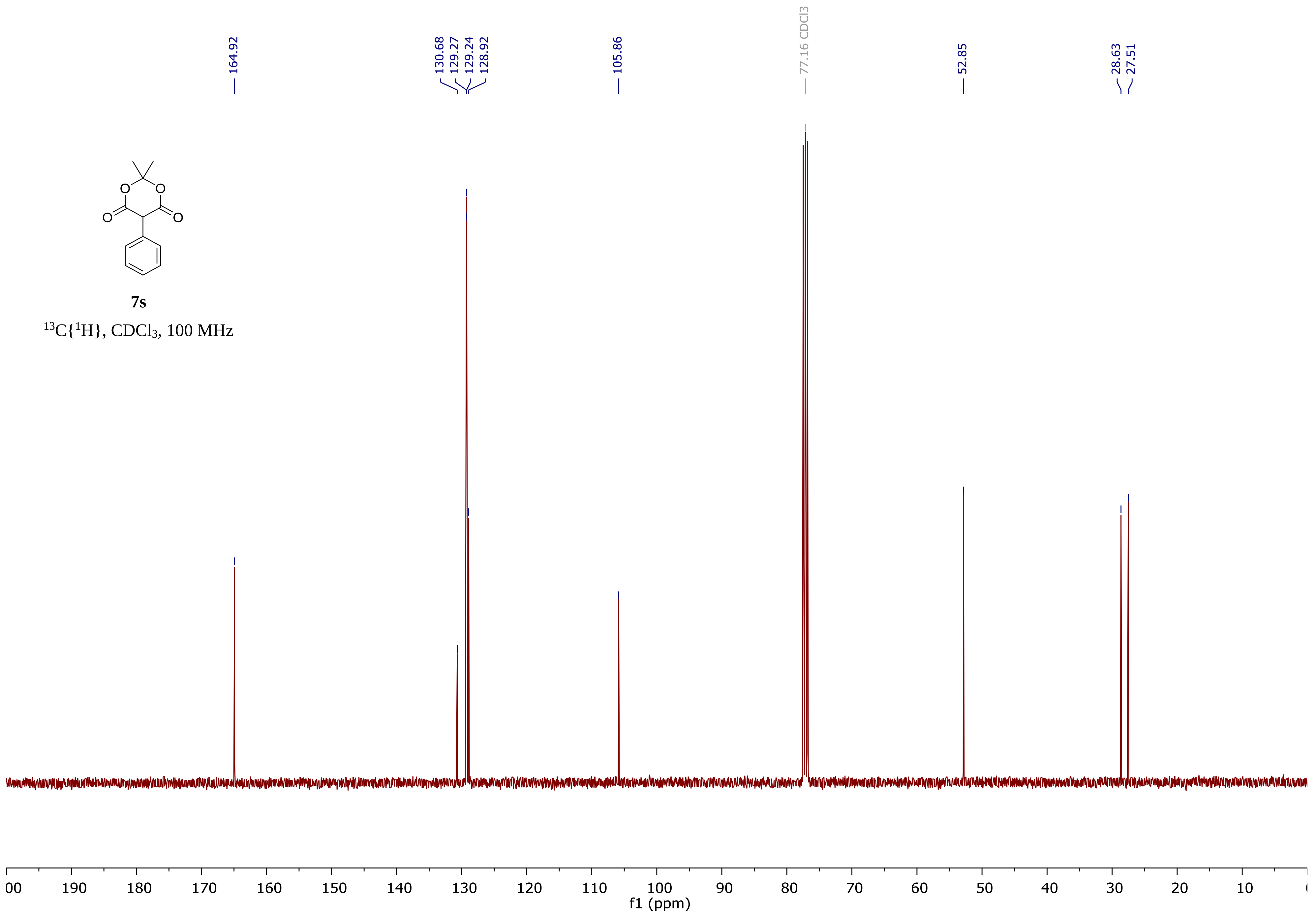

164.92
130.68
129.27
129.24
128.92
105.86
77.16 CDCl3
52.85
28.63
27.51
7s
$^{13}C\{^1H\}$, $CDCl_3$, 100 MHz
00
190
180
170
160
150
140
130
120
110
100
90
80
70
60
50
40
30
20
10
f1 (ppm)

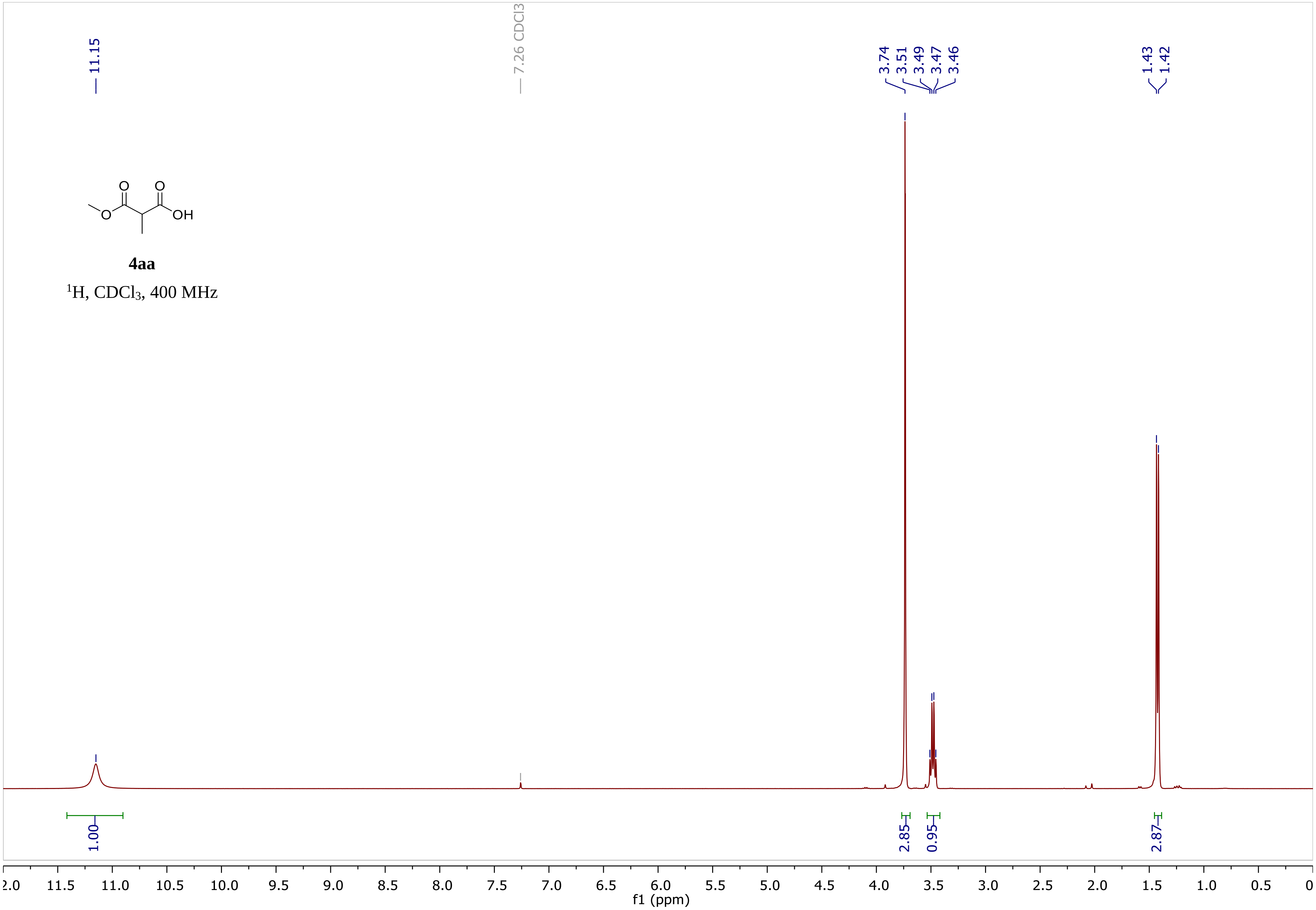
11.15
7.26 CDCl3
3.74
3.51
3.49
3.47
3.46
1.43
1.42
4aa
1H, CDCl3, 400 MHz
OH
1.00
2.85
0.95
2.87
f1 (ppm)

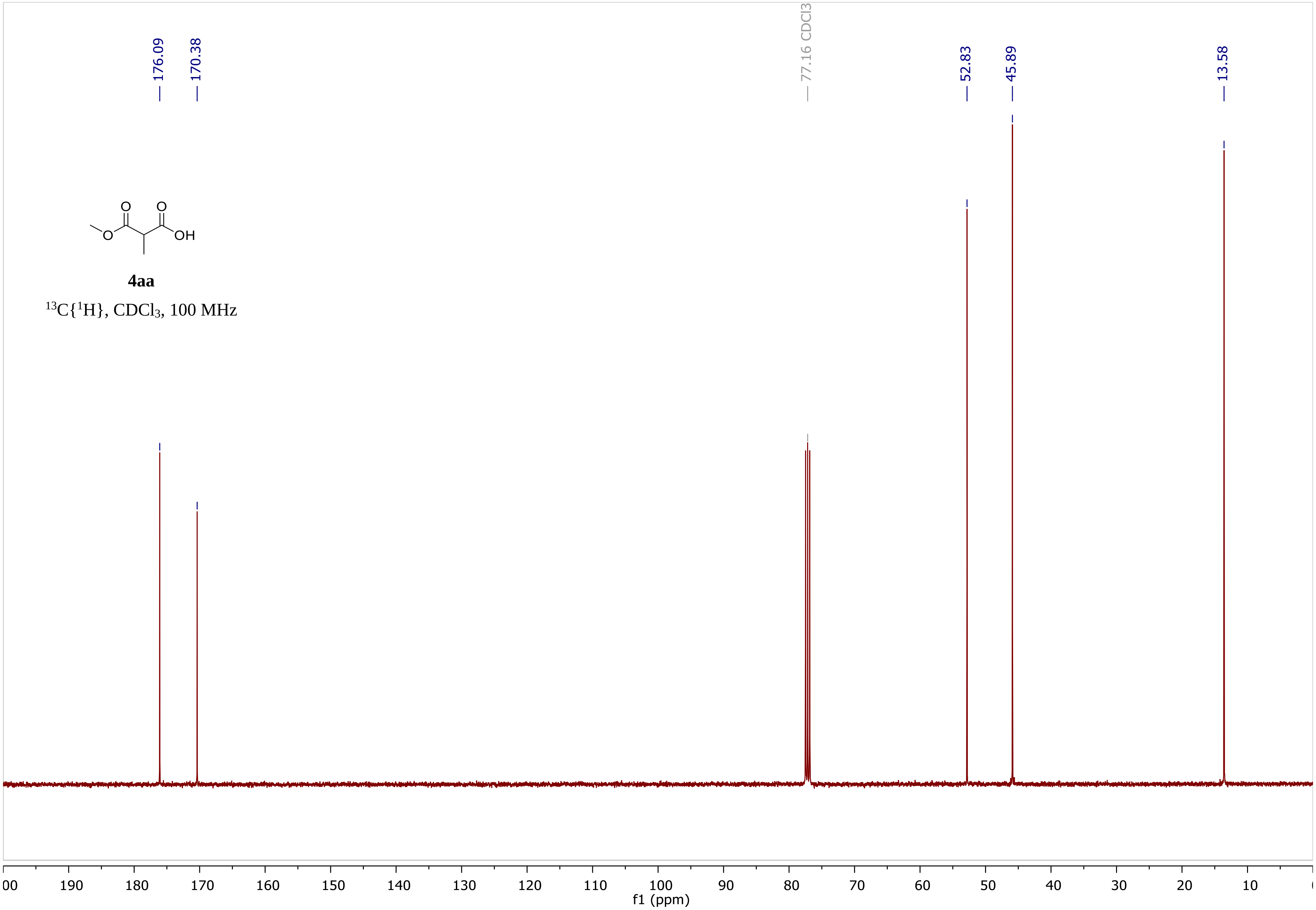
176.09
170.38
77.16 CDCl3
52.83
45.89
13.58
OH
4aa
13C{1H}, CDCl3, 100 MHz
00
190
180
170
160
150
140
130
120
110
100
90
80
70
60
50
40
30
20
10
f1 (ppm)

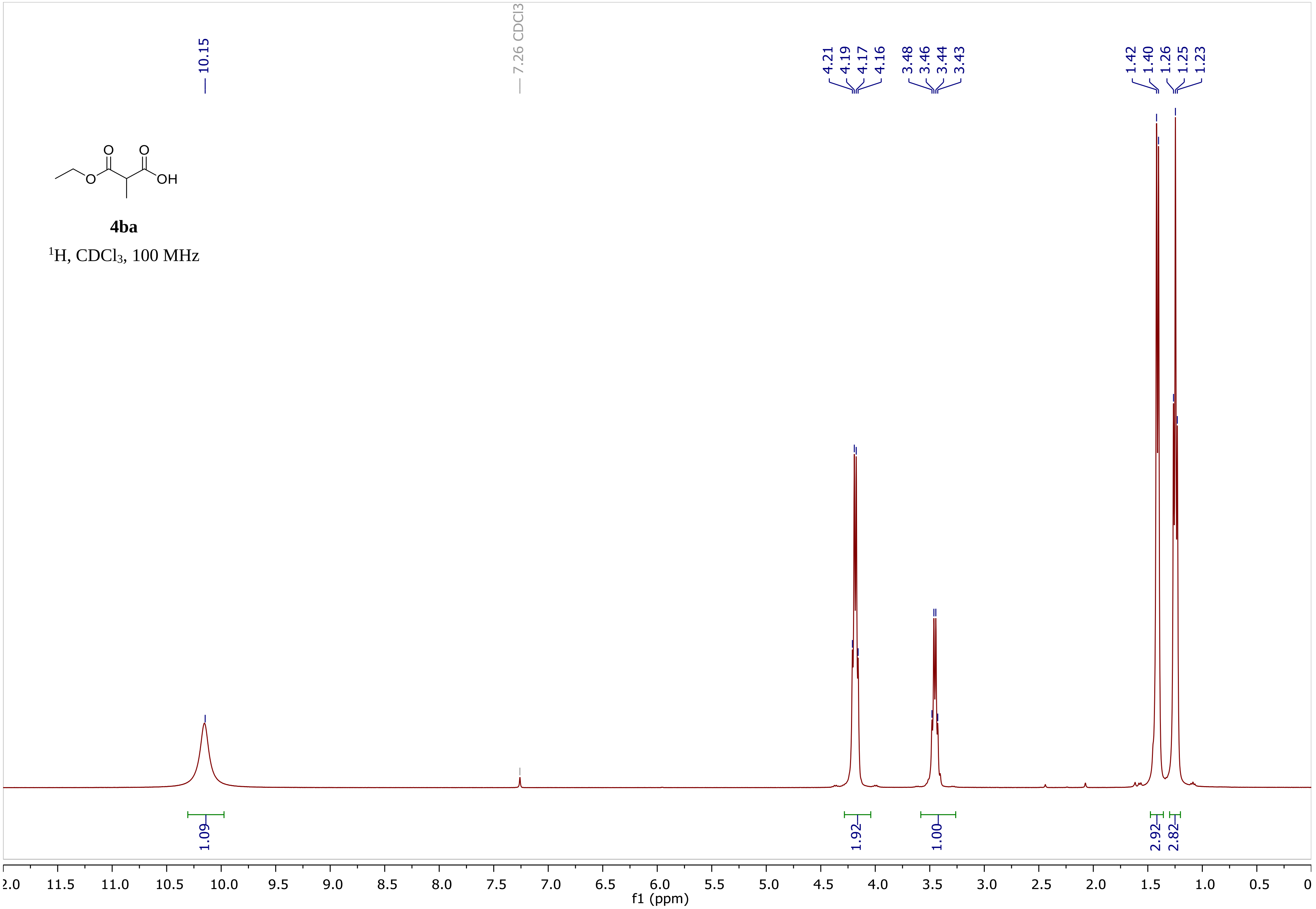
10.15
7.26 CDCl3
4.21 4.19 4.17 4.16
3.48 3.46 3.44 3.43
1.42 1.40 1.26 1.25 1.23
O O
O OH
4ba
1H, CDCl3, 100 MHz
1.09
1.92
1.00
2.92
2.82
12.0 11.5 11.0 10.5 10.0 9.5 9.0 8.5 8.0 7.5 7.0 6.5 6.0 5.5 5.0 4.5 4.0 3.5 3.0 2.5 2.0 1.5 1.0 0.5 0
f1 (ppm)

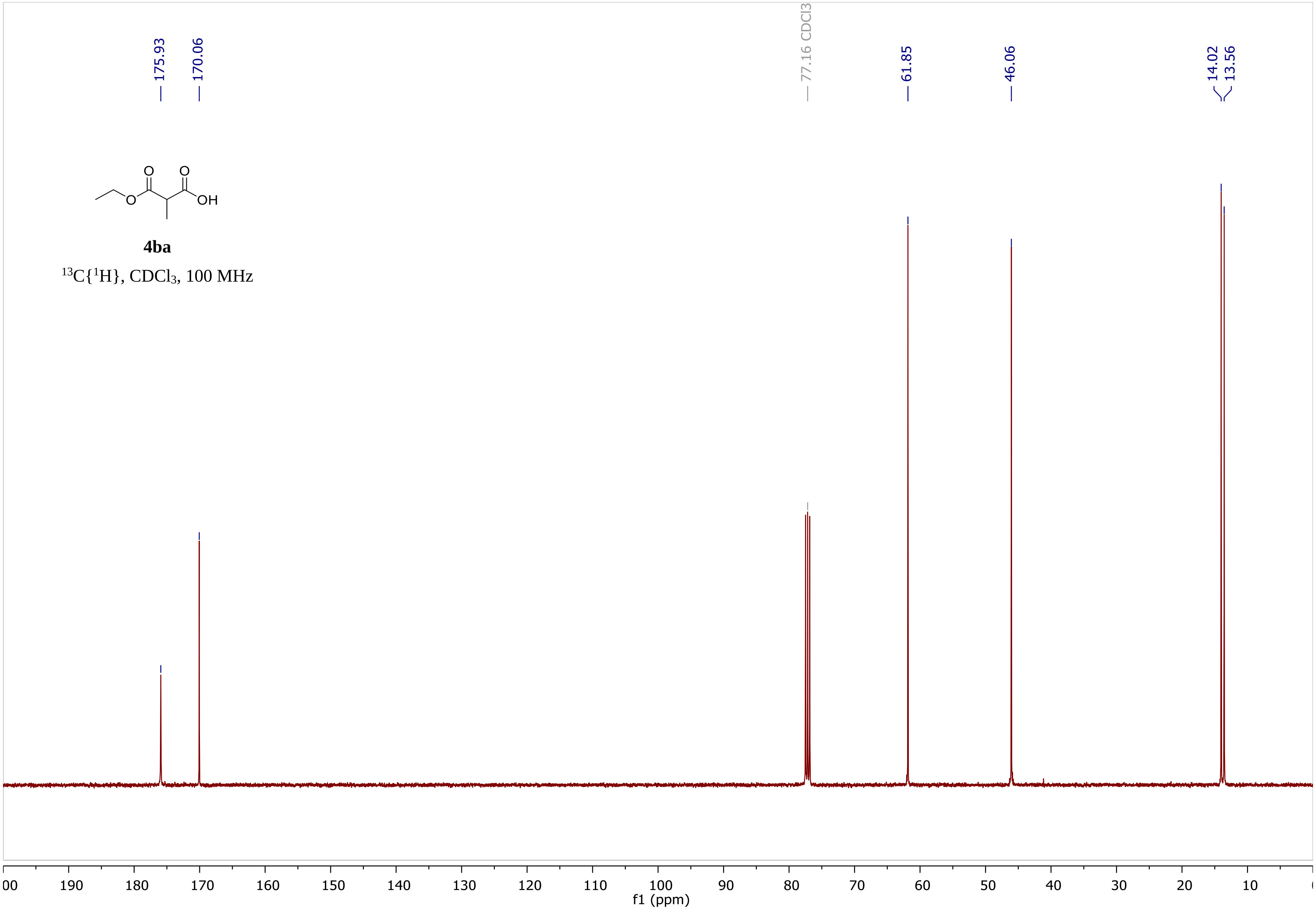

175.93
170.06
77.16 CDCl3
61.85
46.06
14.02
13.56
O
O
O
OH
4ba
13C{1H}, CDCl3, 100 MHz
00
190
180
170
160
150
140
130
120
110
100
90
80
70
60
50
40
30
20
10
f1 (ppm)

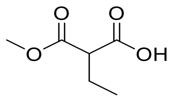

4ab

^1H, $CDCl_3$, 100 MHz

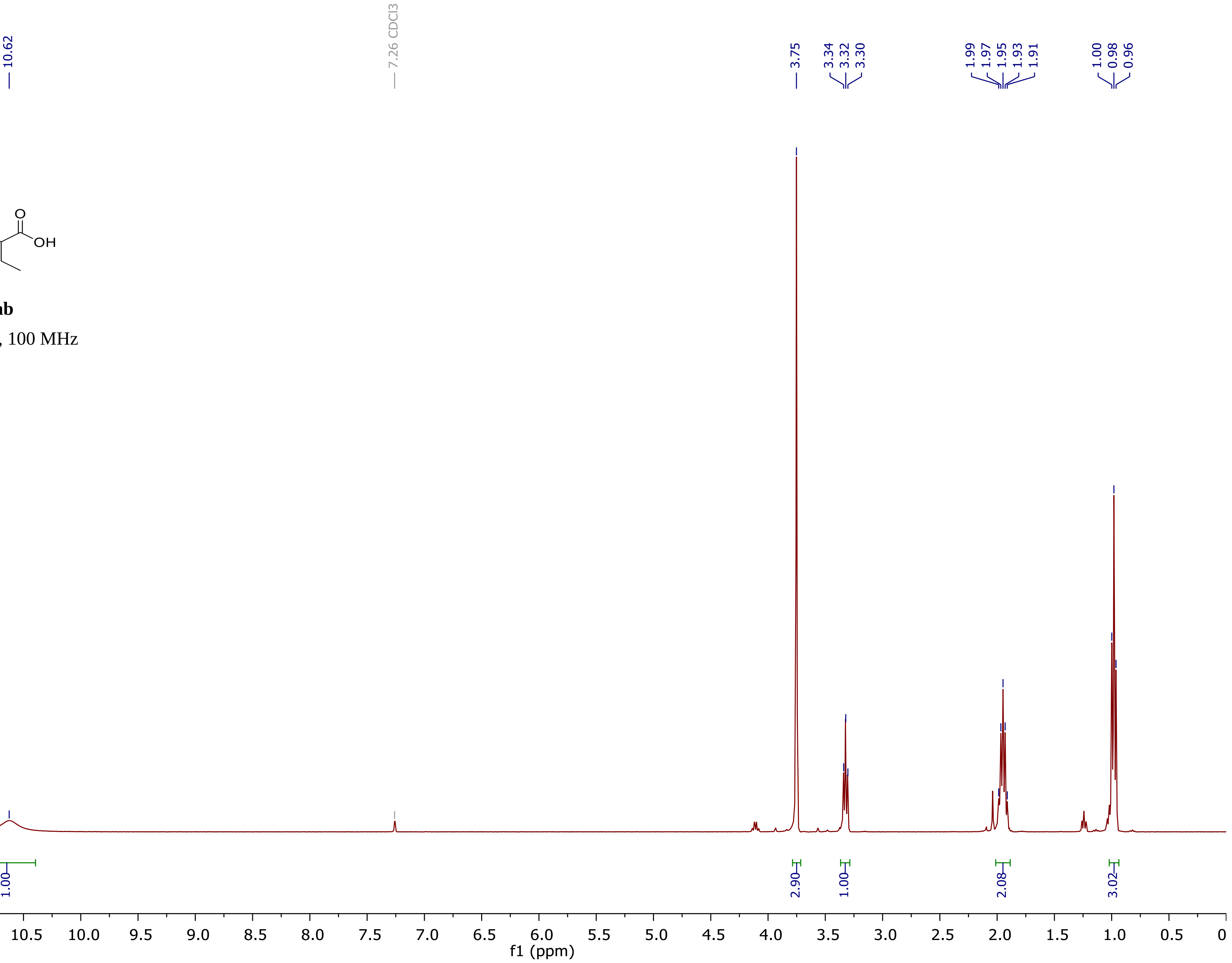

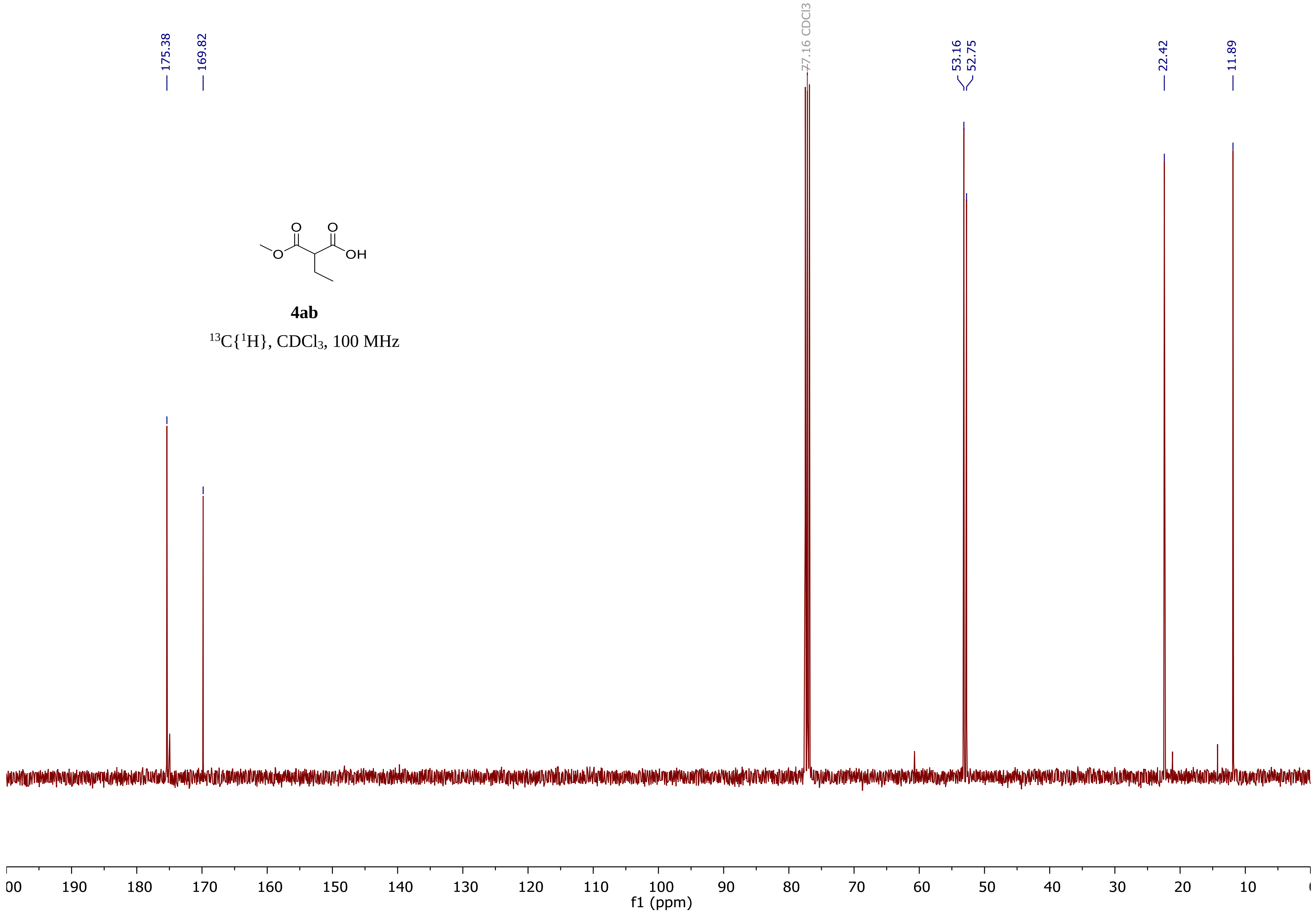
175.38
169.82
77.16 CDCl3
53.16
52.75
22.42
11.89
O
O
O
OH
4ab
$^{13}C\{^{1}H\}$, $CDCl_3$, 100 MHz
00
190
180
170
160
150
140
130
120
110
100
90
80
70
60
50
40
30
20
10
f1 (ppm)

4ac

^1H, $CDCl_3$, 400 MHz

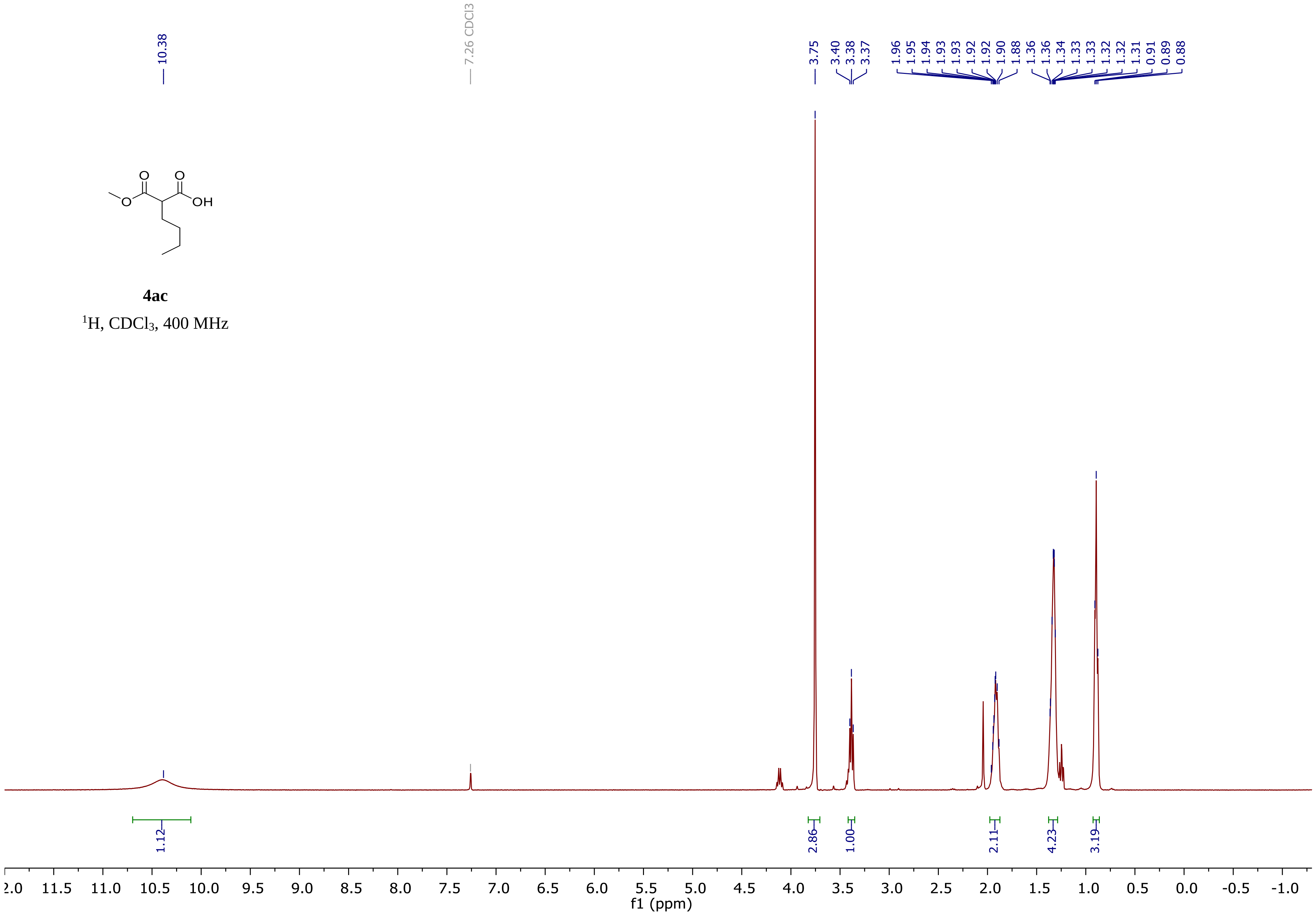

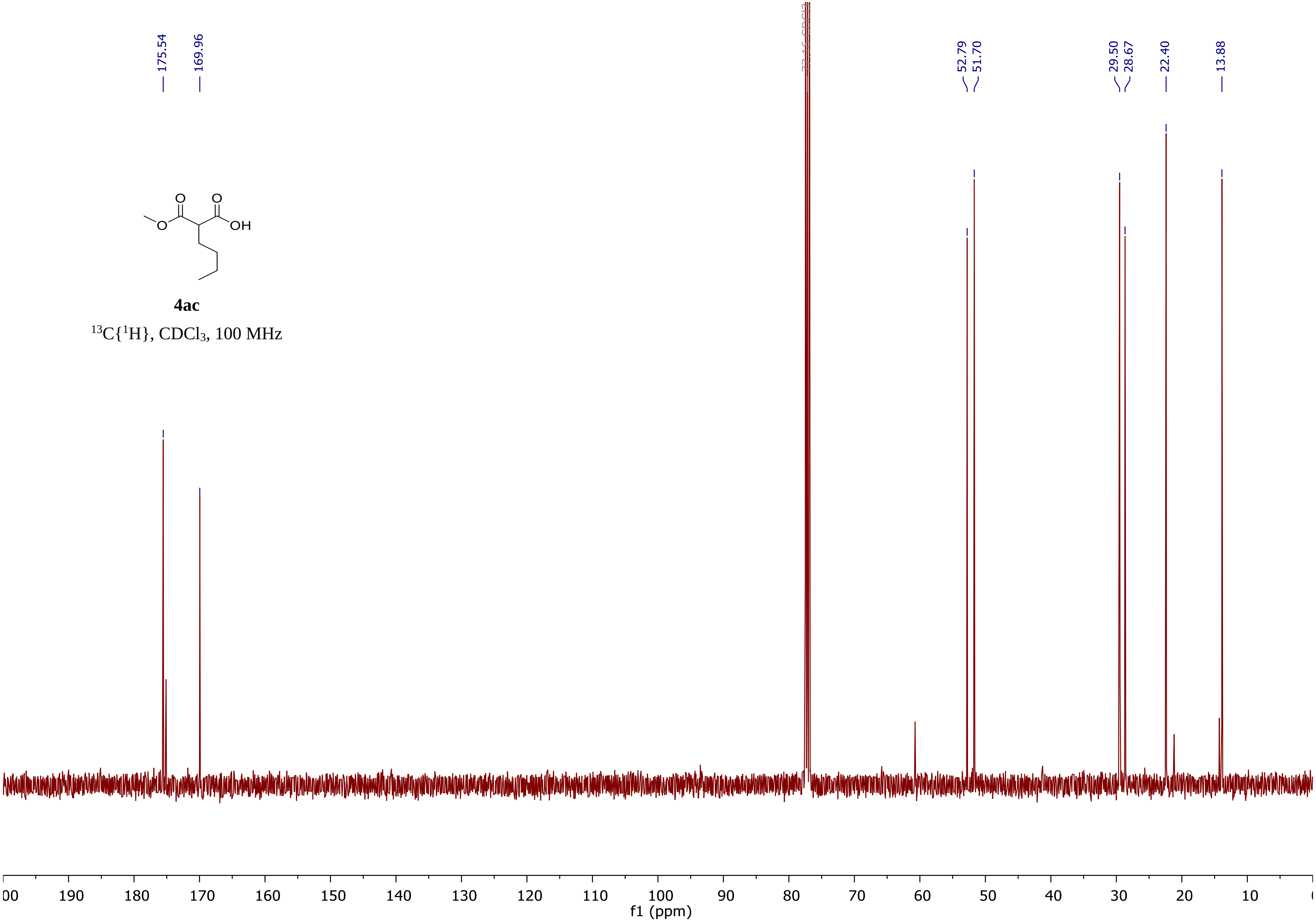

4ac

$^{13}C\{^{1}H\}$, $CDCl_3$, 100 MHz

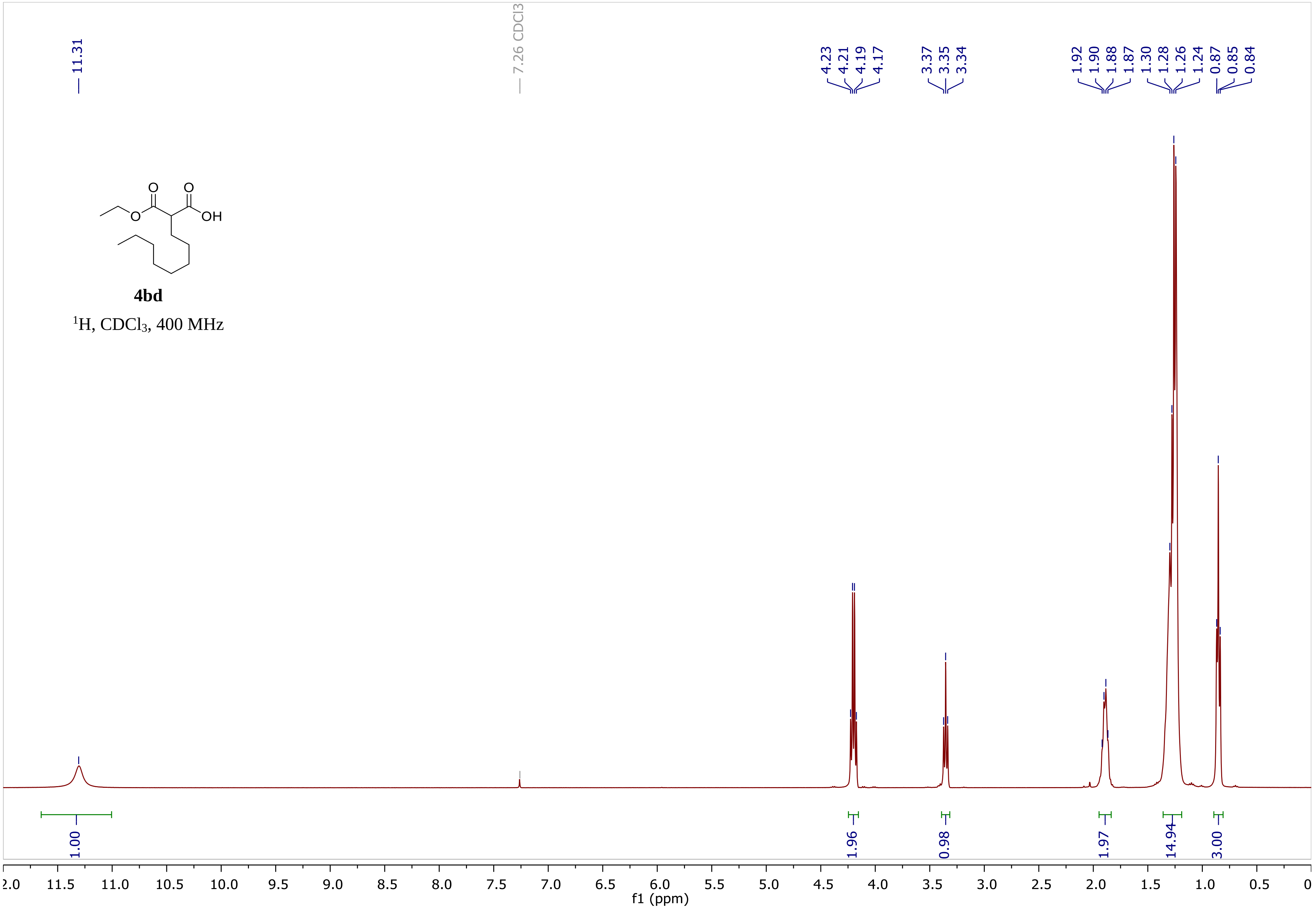

4bd
^{1}H, $CDCl_3$, 400 MHz
11.31
7.26 CDCl3
4.23 4.21 4.19 4.17
3.37 3.35 3.34
1.92 1.90 1.88 1.87 1.30 1.28 1.26 1.24 0.87 0.85 0.84
1.00
1.96
0.98
1.97
14.94
3.00
f1 (ppm)

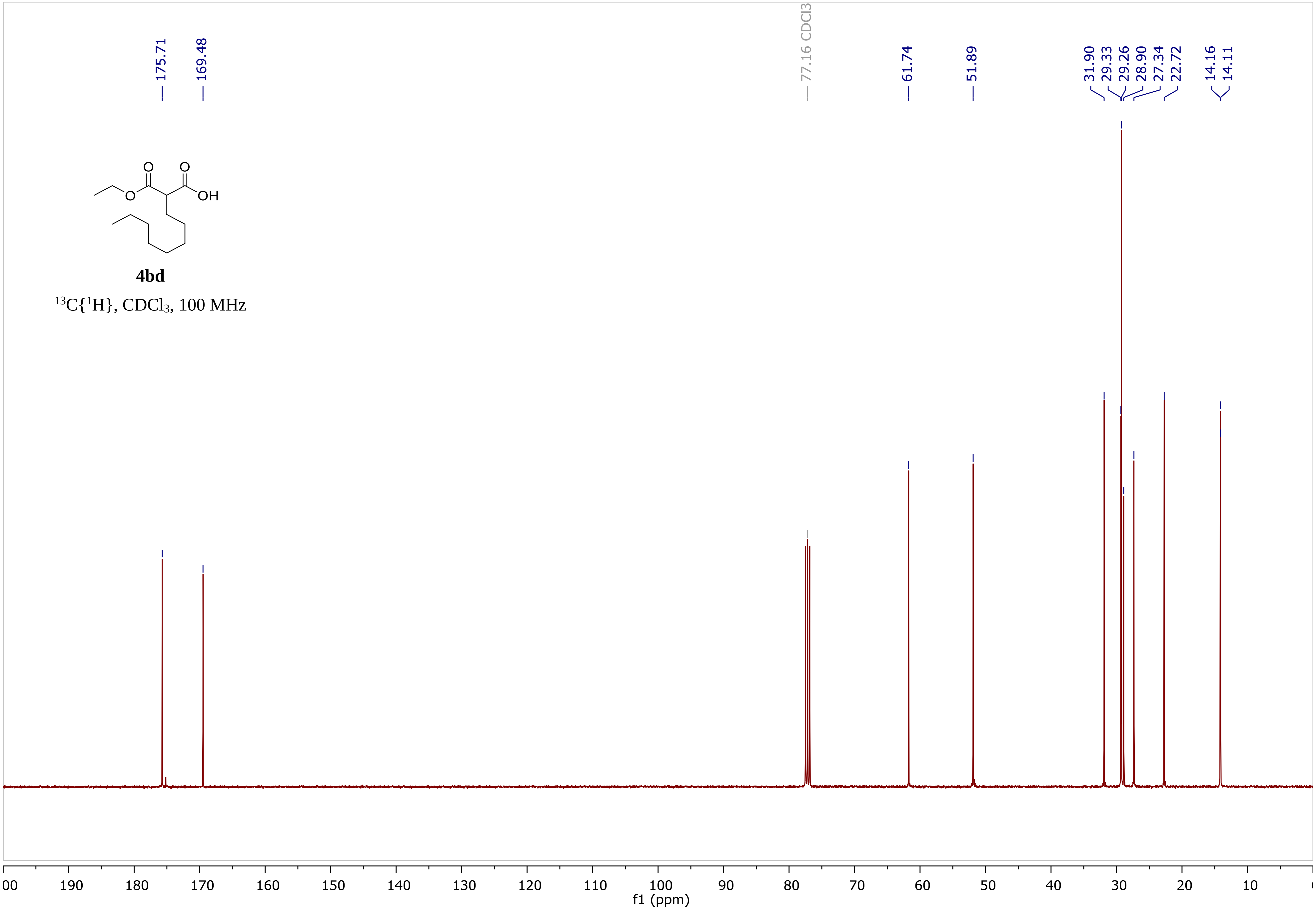

175.71
169.48
77.16 CDCl3
61.74
51.89
31.90
29.33
29.26
28.90
27.34
22.72
14.16
14.11
O
O
O
OH
4bd
13C{1H}, CDCl3, 100 MHz
190
180
170
160
150
140
130
120
110
100
90
80
70
60
50
40
30
20
10
f1 (ppm)

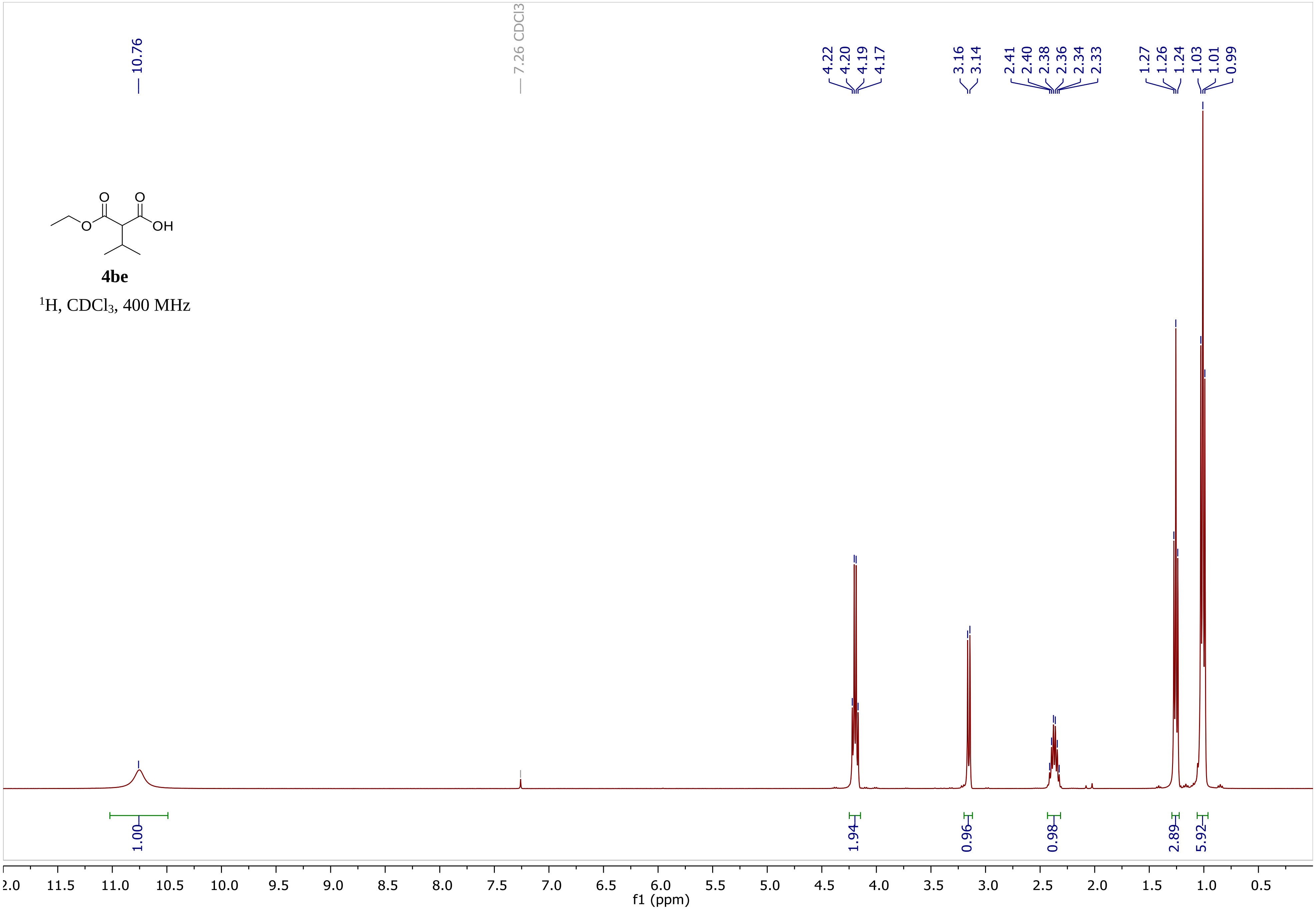
10.76
7.26 CDCl3
4.22
4.20
4.19
4.17
3.16
3.14
2.41
2.40
2.38
2.36
2.34
2.33
1.27
1.26
1.24
1.03
1.01
0.99
O
O
O
OH
4be
^{1}H, $CDCl_3$, 400 MHz
1.00
1.94
0.96
0.98
2.89
5.92
f1 (ppm)

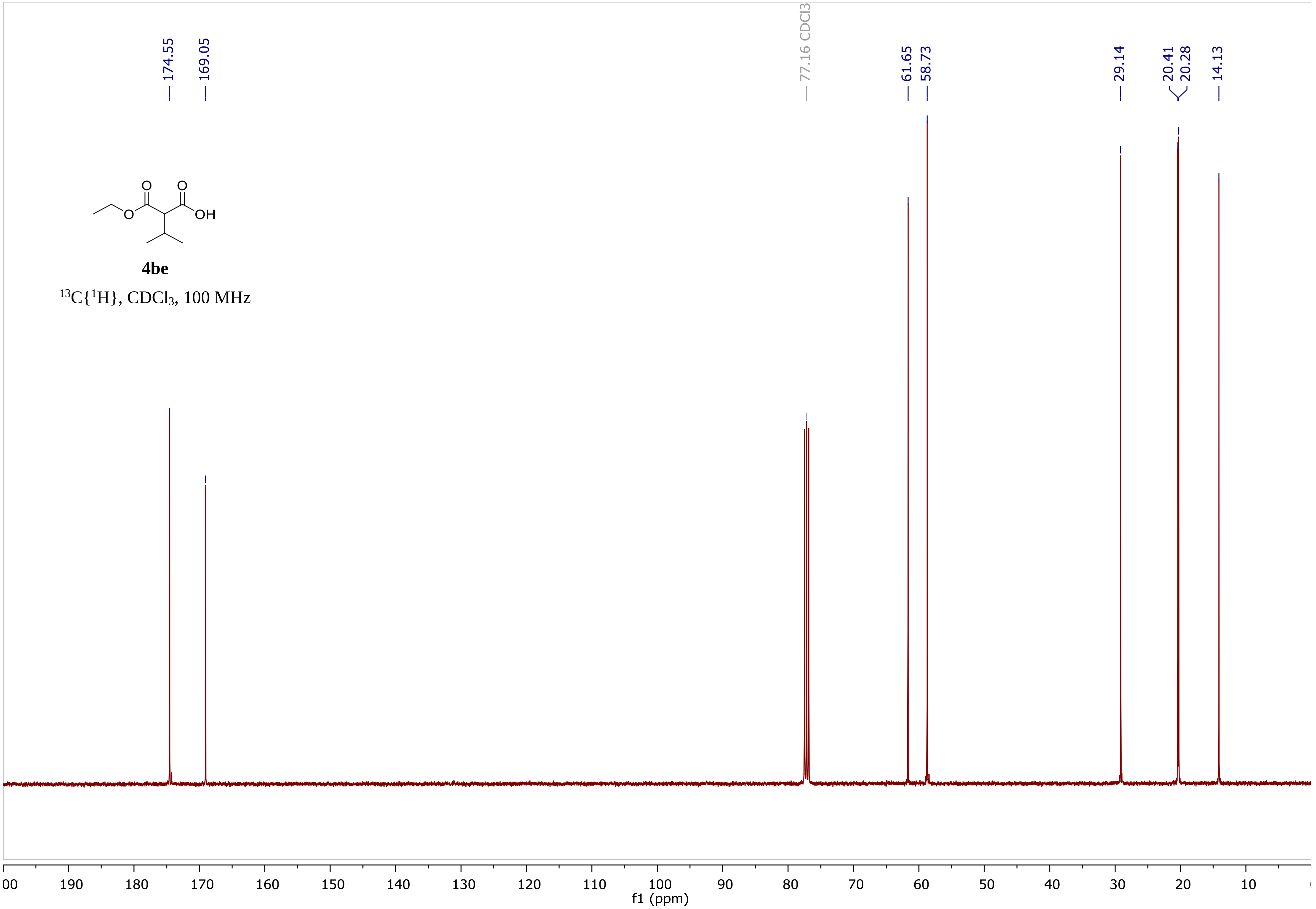

174.55
169.05
77.16 CDCl3
61.65
58.73
29.14
20.41
20.28
14.13
OH
4be
$^{13}C\{^{1}H\}$, $CDCl_3$, 100 MHz
190
180
170
160
150
140
130
120
110
100
90
80
70
60
50
40
30
20
10
f1 (ppm)

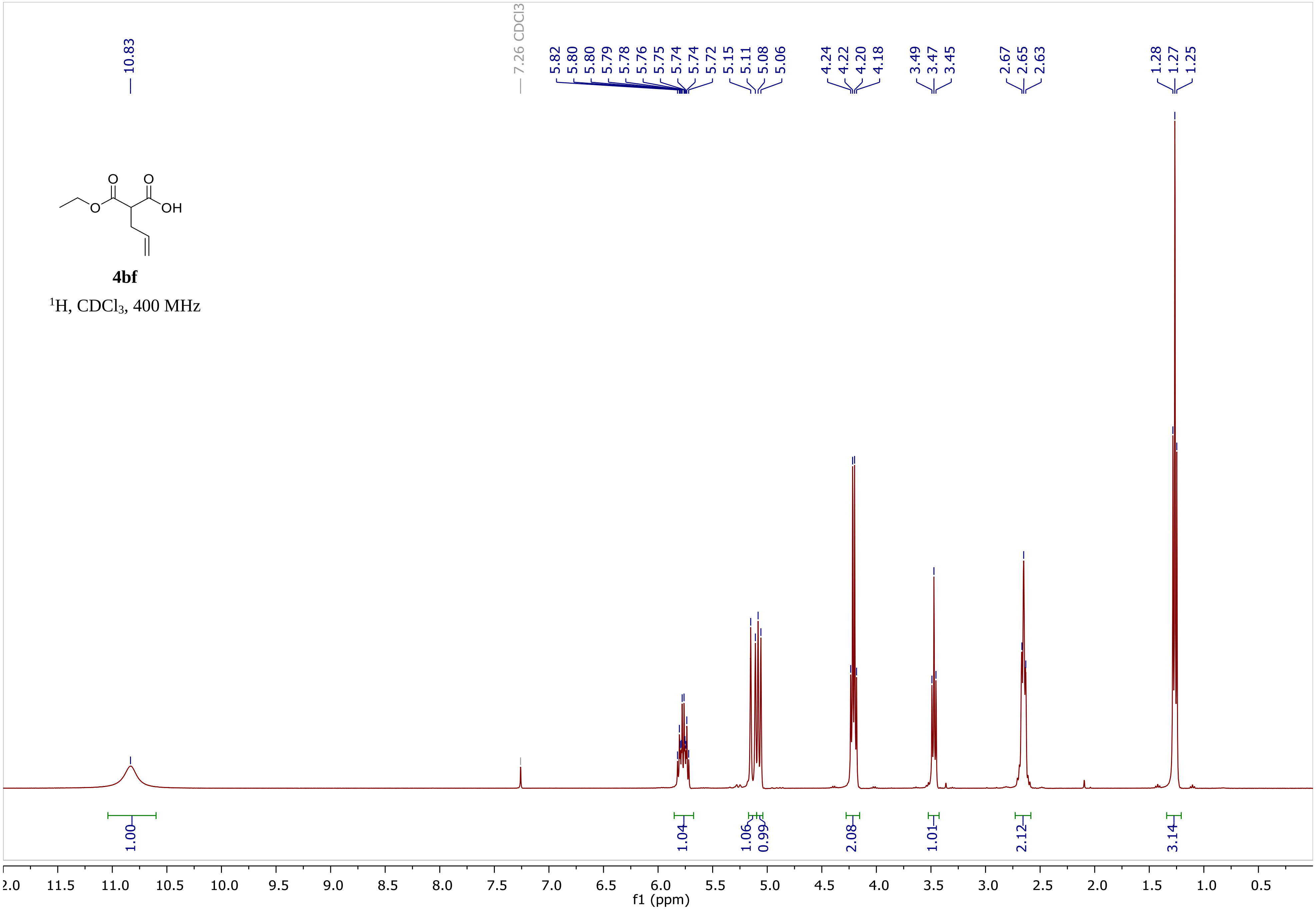
4bf
1H, CDCl3, 400 MHz
10.83
7.26 CDCl3
5.82 5.80 5.80 5.79 5.78 5.76 5.75 5.74 5.74 5.72 5.15 5.11 5.08 5.06
4.24 4.22 4.20 4.18
3.49 3.47 3.45
2.67 2.65 2.63
1.28 1.27 1.25
1.00 1.04 1.06 0.99 2.08 1.01 2.12 3.14
f1 (ppm)

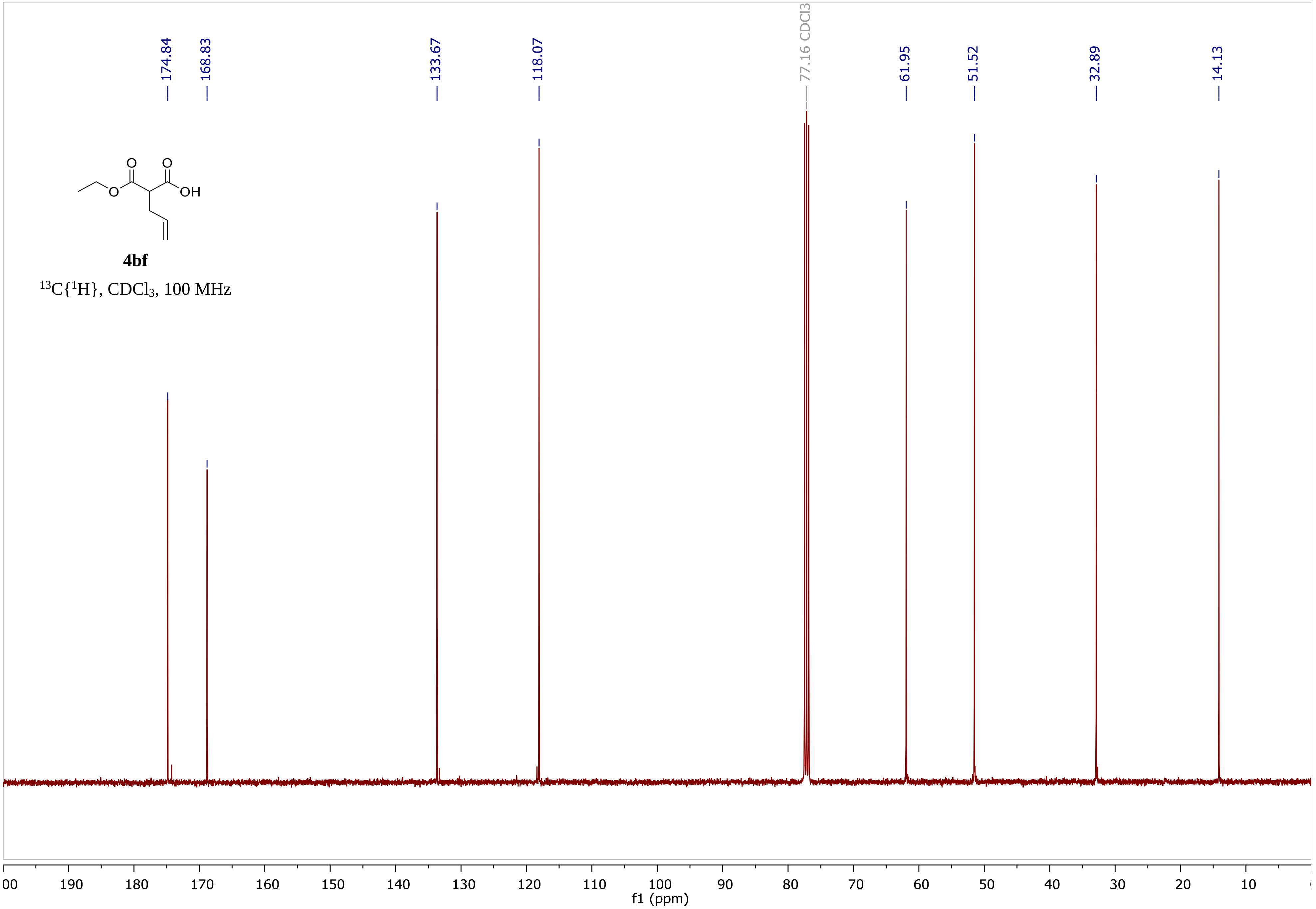

4bf

$^{13}C\{^{1}H\}$, $CDCl_3$, 100 MHz

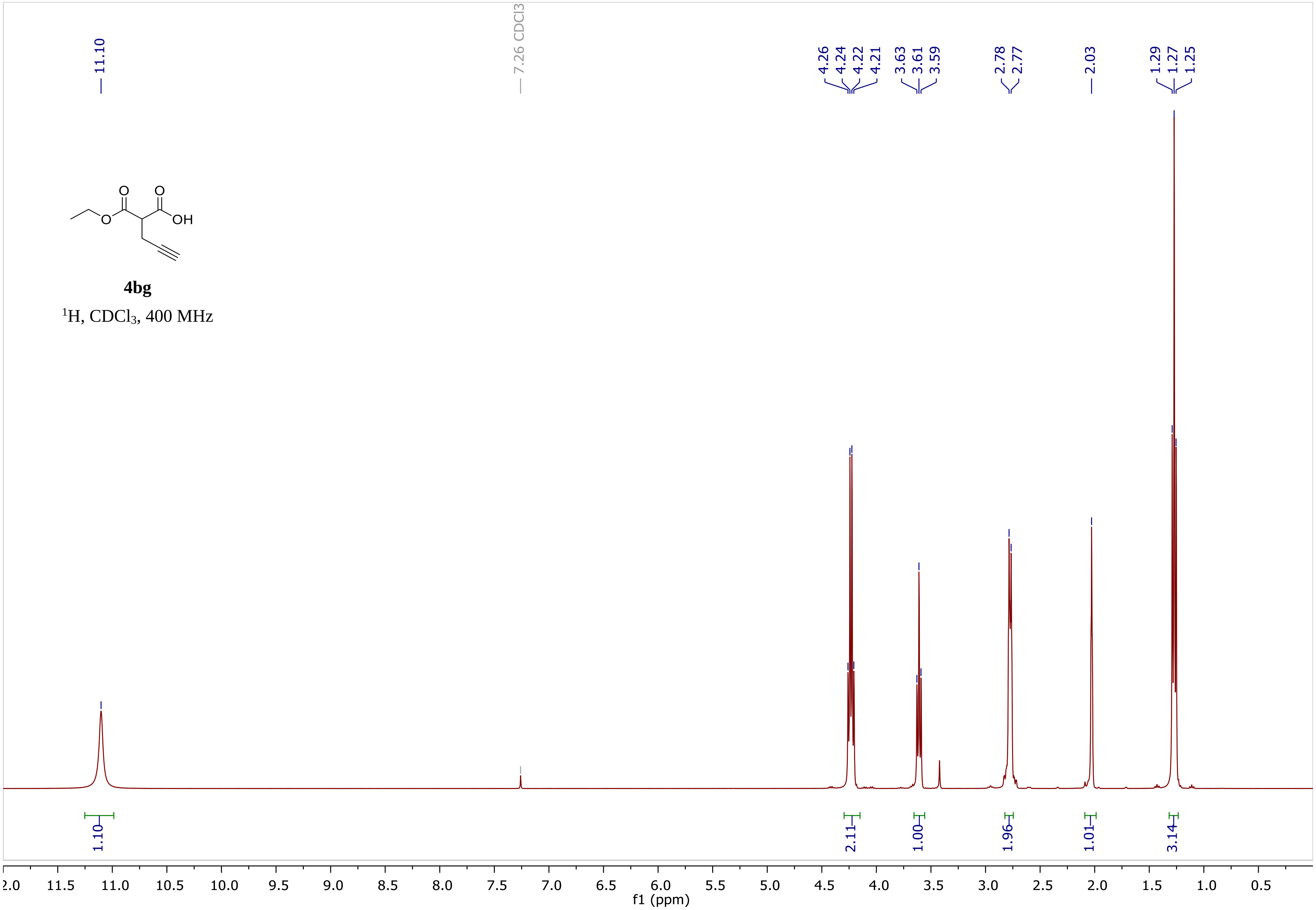
11.10
7.26 CDCl3
4.26
4.24
4.22
4.21
3.63
3.61
3.59
2.78
2.77
2.03
1.29
1.27
1.25
O
O
O
OH
4bg
^{1}H, $CDCl_3$, 400 MHz
1.10
2.11
1.00
1.96
1.01
3.14
12.0 11.5 11.0 10.5 10.0 9.5 9.0 8.5 8.0 7.5 7.0 6.5 6.0 5.5 5.0 4.5 4.0 3.5 3.0 2.5 2.0 1.5 1.0 0.5
f1 (ppm)

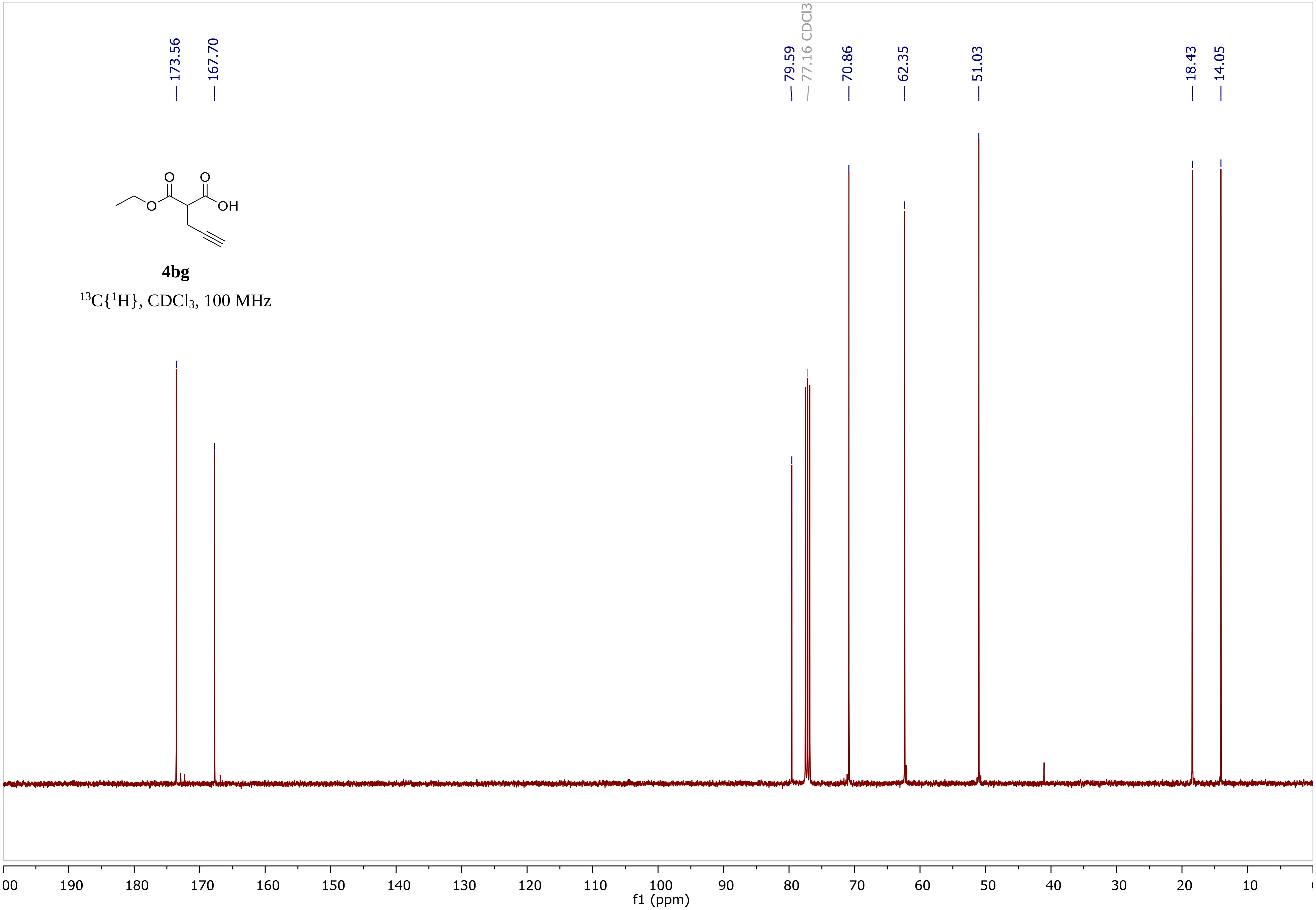

4bg

$^{13}C\{^{1}H\}$, $CDCl_3$, 100 MHz

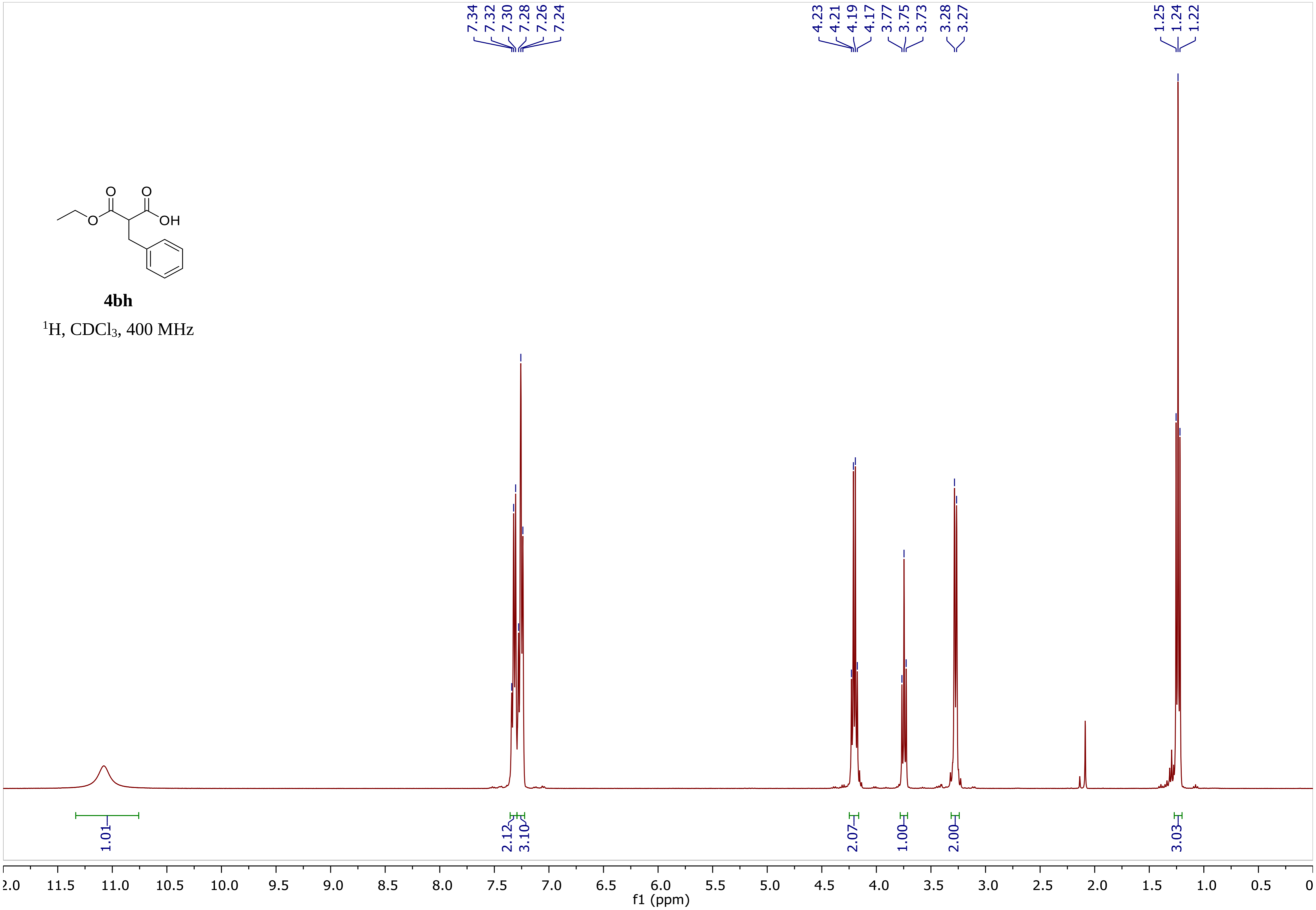
7.34
7.32
7.30
7.28
7.26
7.24
4.23
4.21
4.19
4.17
3.77
3.75
3.73
3.28
3.27
1.25
1.24
1.22
O
O
O
OH
4bh
^{1}H, $CDCl_3$, 400 MHz
1.01
2.12
3.10
2.07
1.00
2.00
3.03
f1 (ppm)

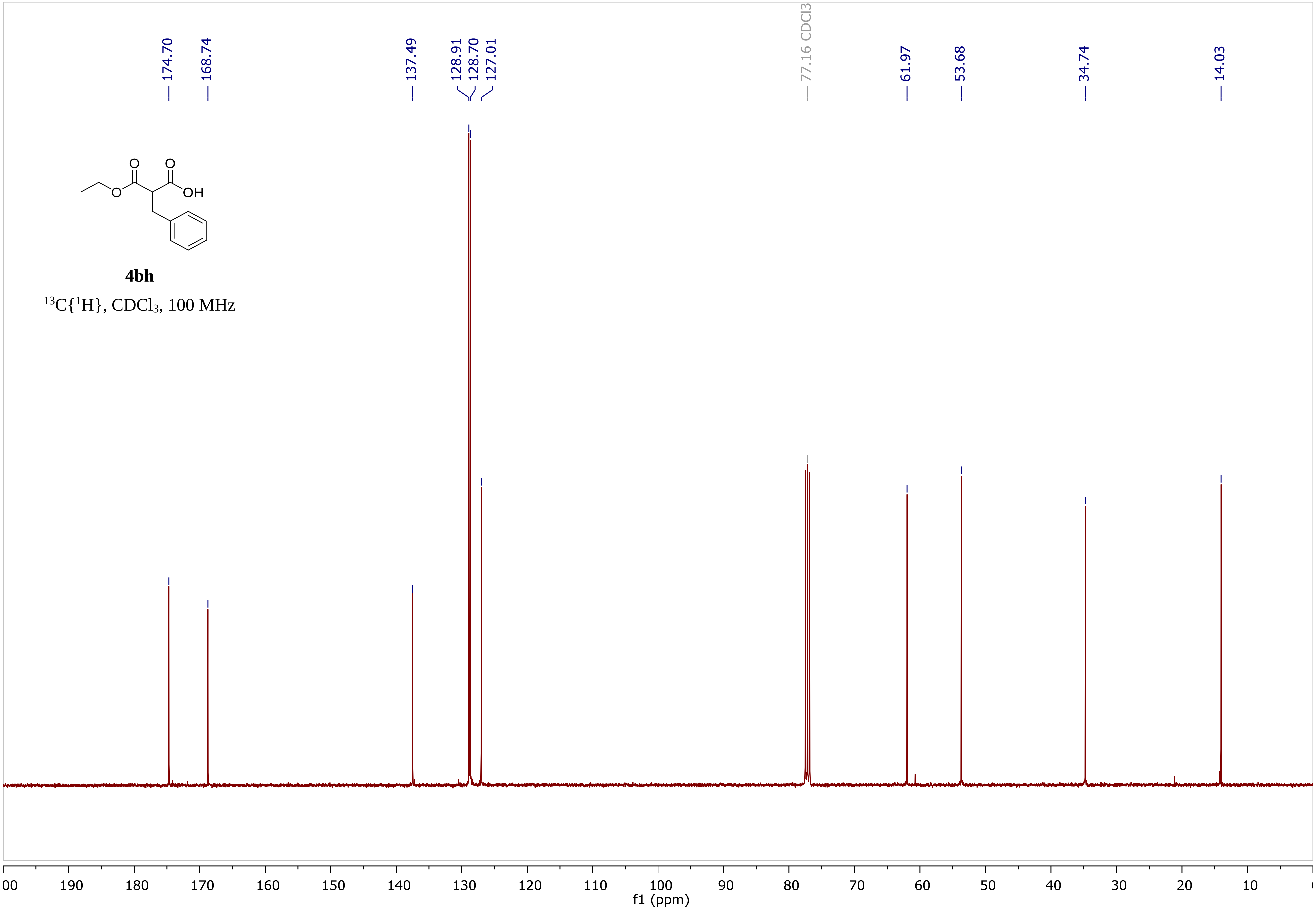
174.70
168.74
137.49
128.91
128.70
127.01
77.16 CDCl3
61.97
53.68
34.74
14.03
OH
O
4bh
13C{1H}, CDCl3, 100 MHz
190
180
170
160
150
140
130
120
110
100
90
80
70
60
50
40
30
20
10
f1 (ppm)

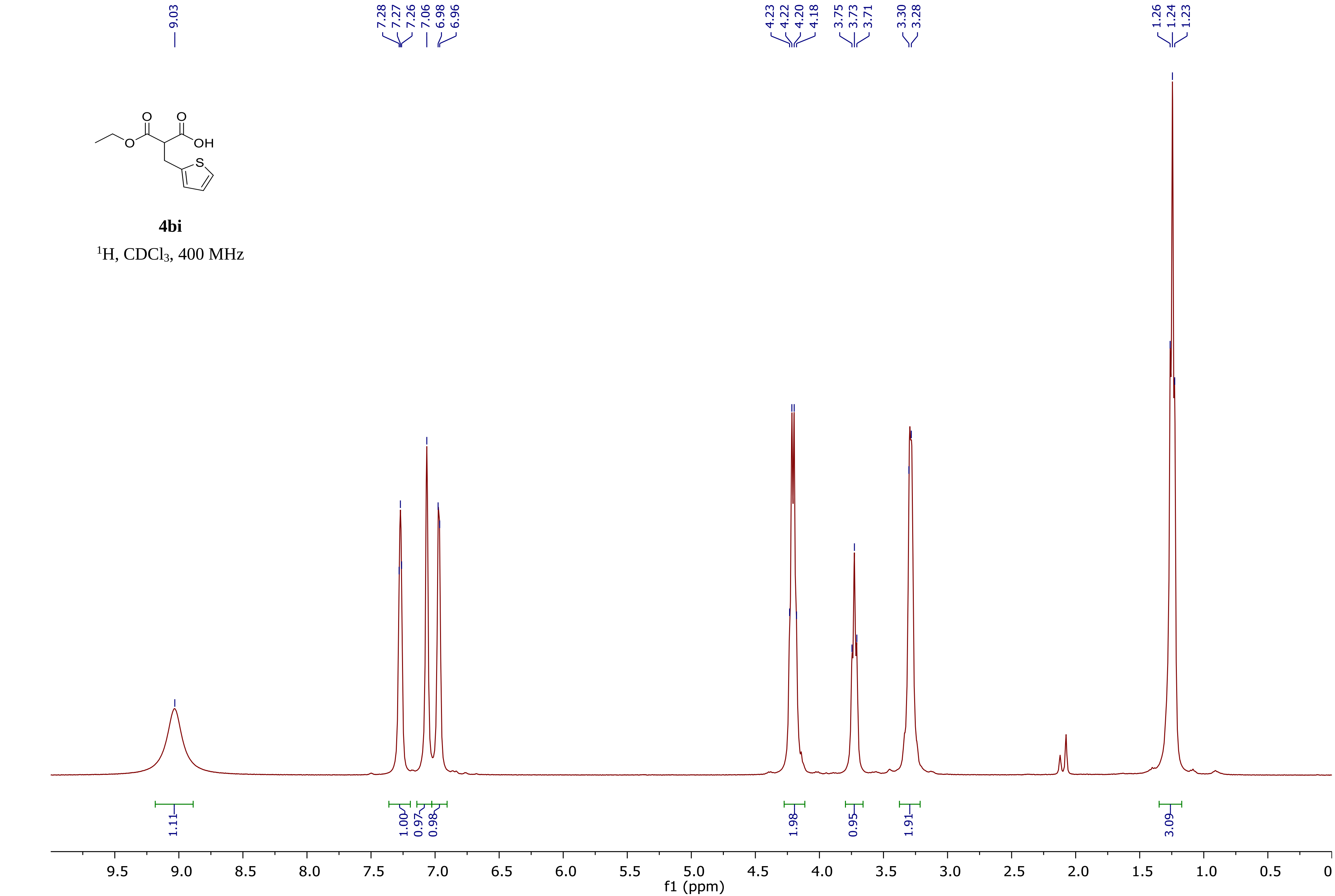

4bi

^{1}H, $CDCl_3$, 400 MHz

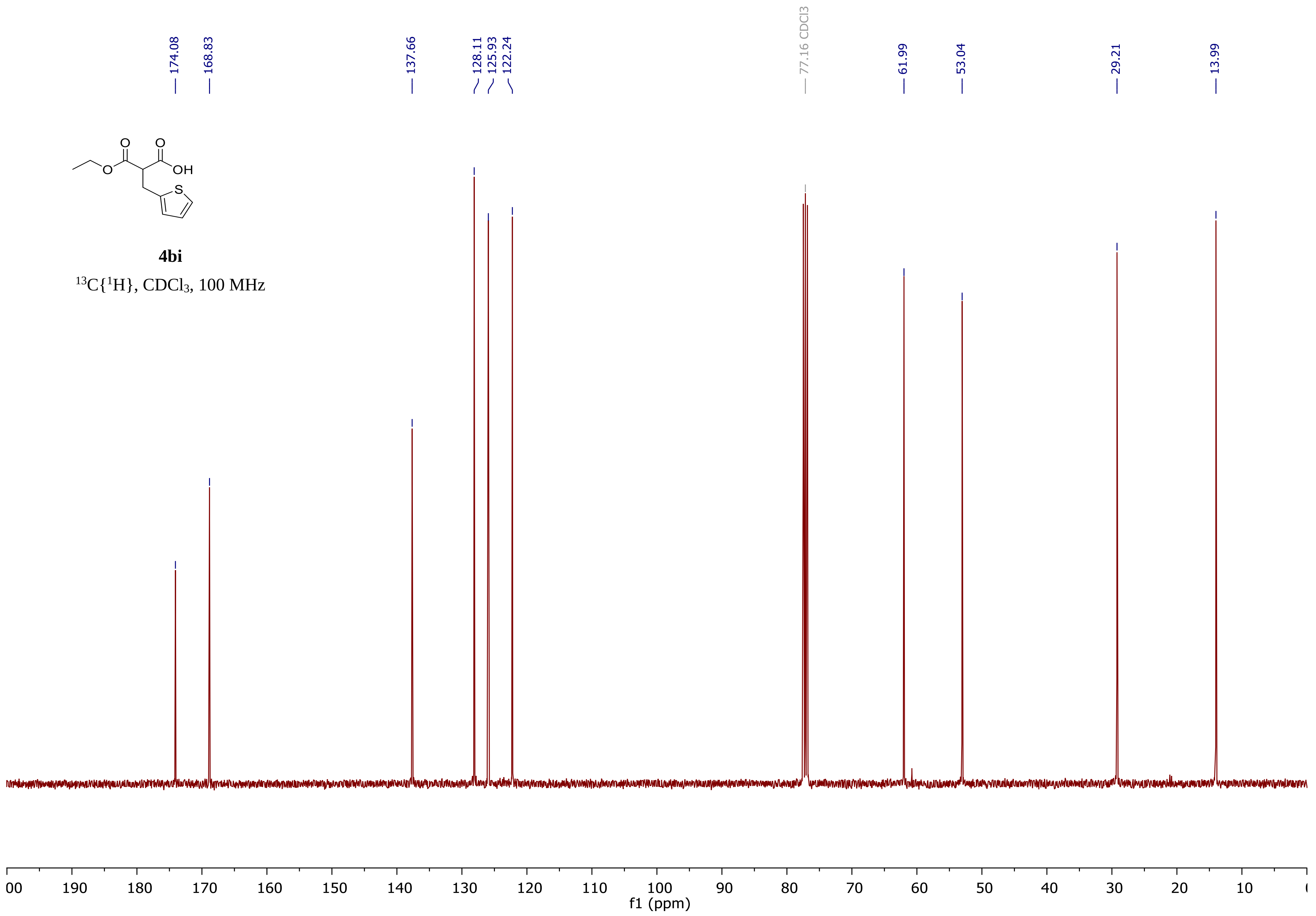

174.08
168.83
137.66
128.11
125.93
122.24
77.16 CDCl3
61.99
53.04
29.21
13.99
O
O
O
OH
S
4bi
$^{13}C\{^{1}H\}$, $CDCl_3$, 100 MHz
00
190
180
170
160
150
140
130
120
110
100
90
80
70
60
50
40
30
20
10
f1 (ppm)

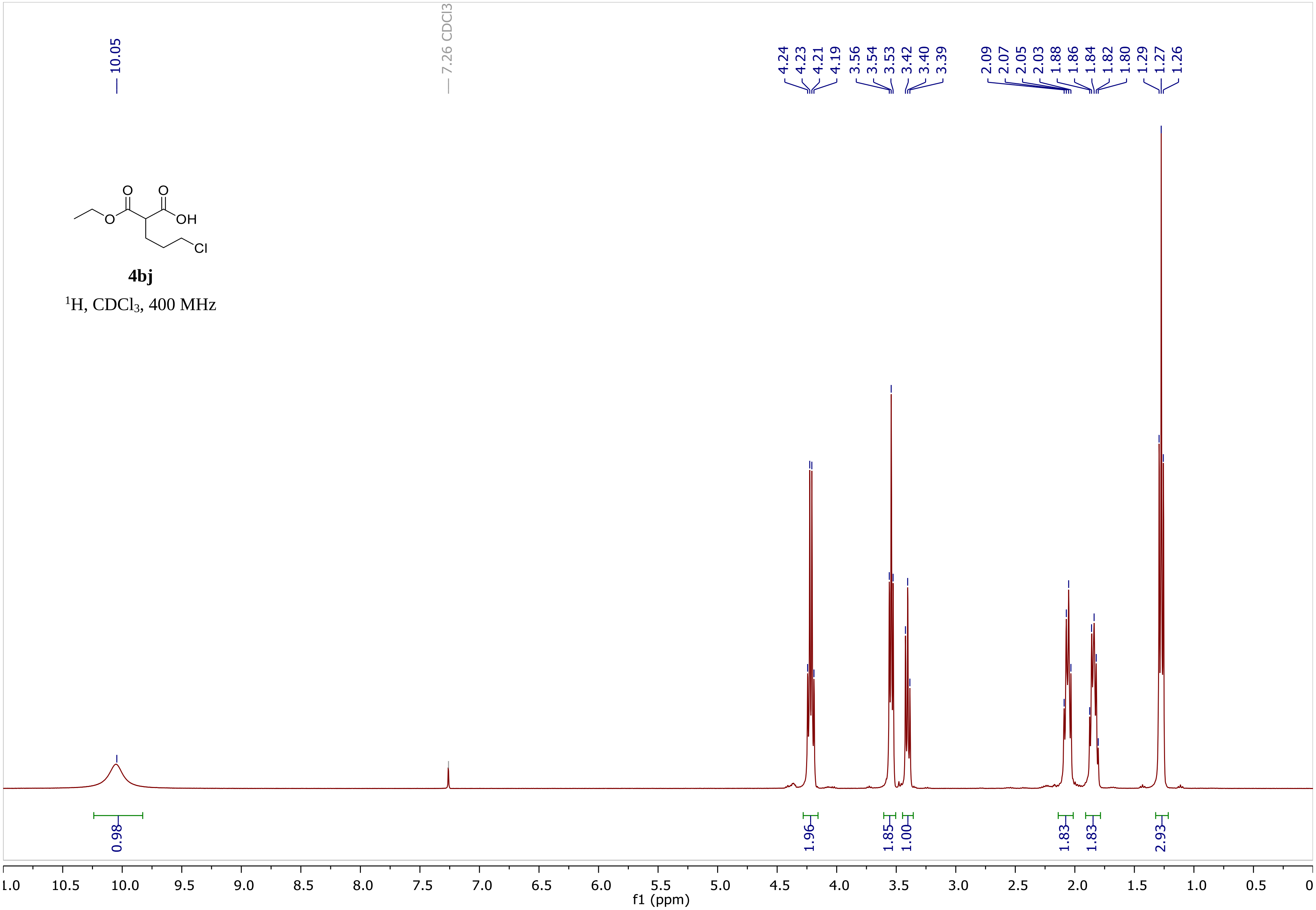

4bj

^{1}H, $CDCl_3$, 400 MHz

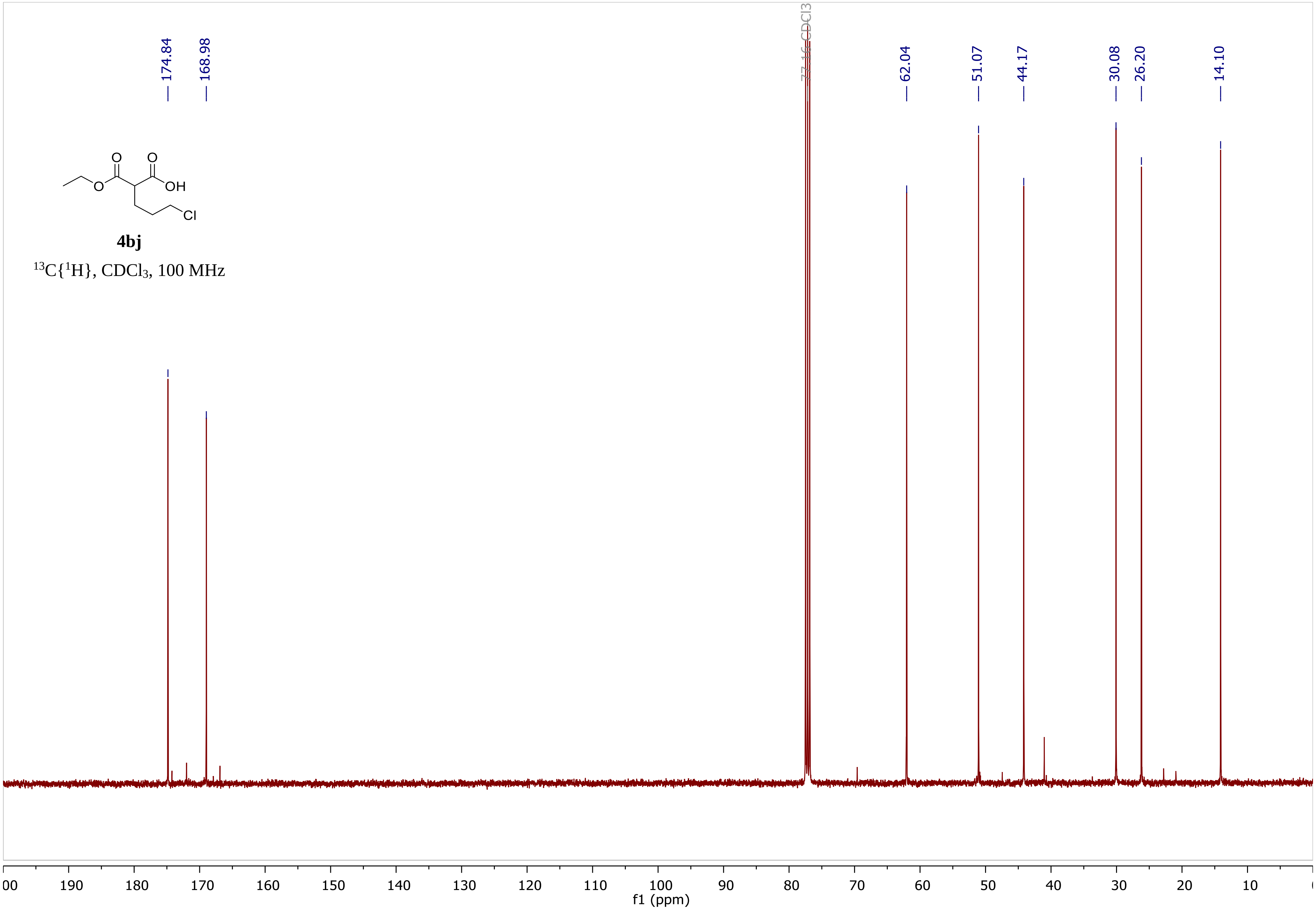

174.84
168.98
77.16 CDCl3
62.04
51.07
44.17
30.08
26.20
14.10
O
O
O
OH
Cl
4bj
$^{13}C\{^{1}H\}$, $CDCl_3$, 100 MHz
00
190
180
170
160
150
140
130
120
110
100
90
80
70
60
50
40
30
20
10
f1 (ppm)

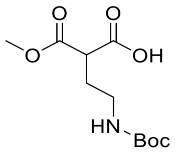

4ak

^{1}H, CD_3COCD_3, 400 MHz

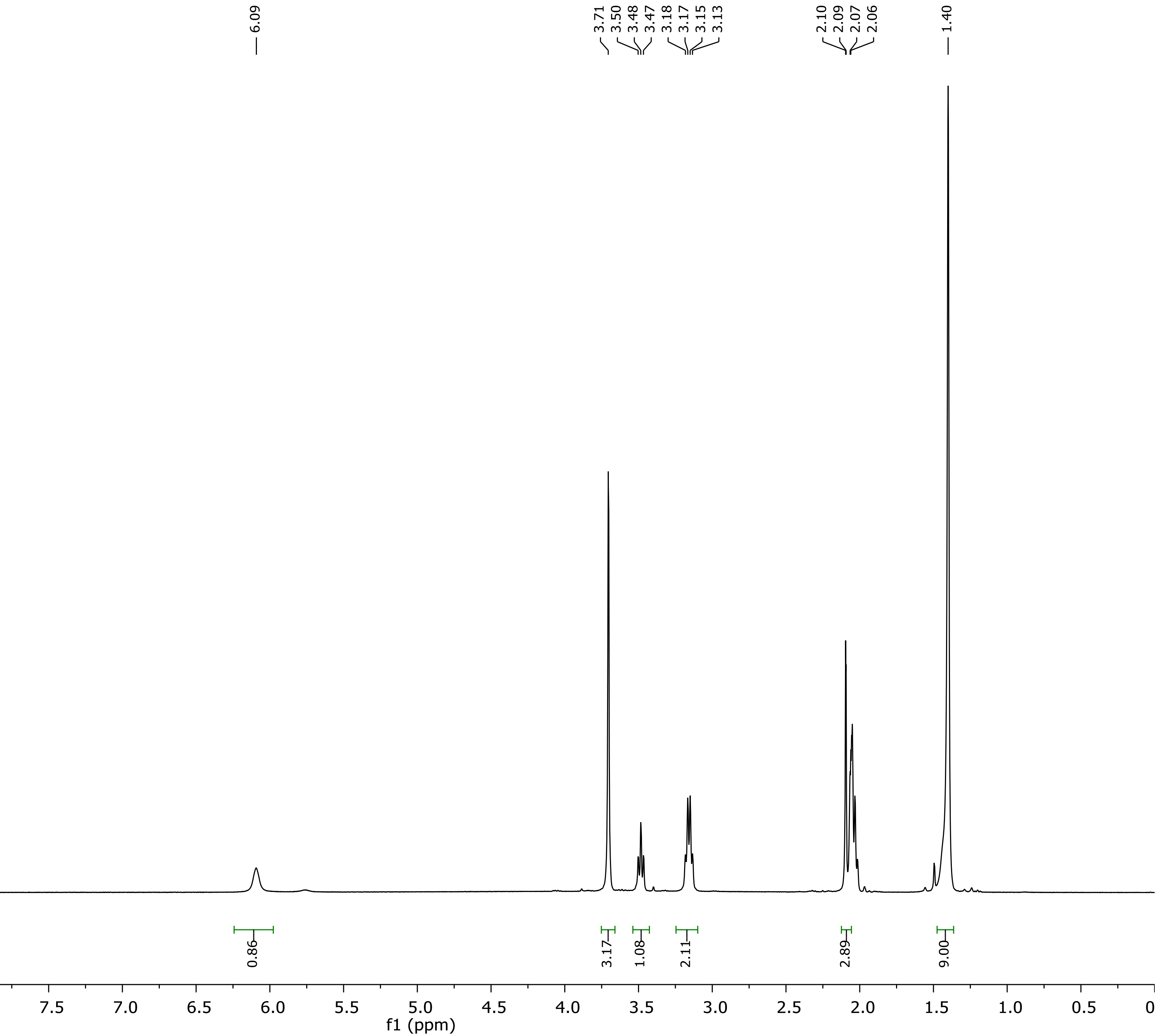

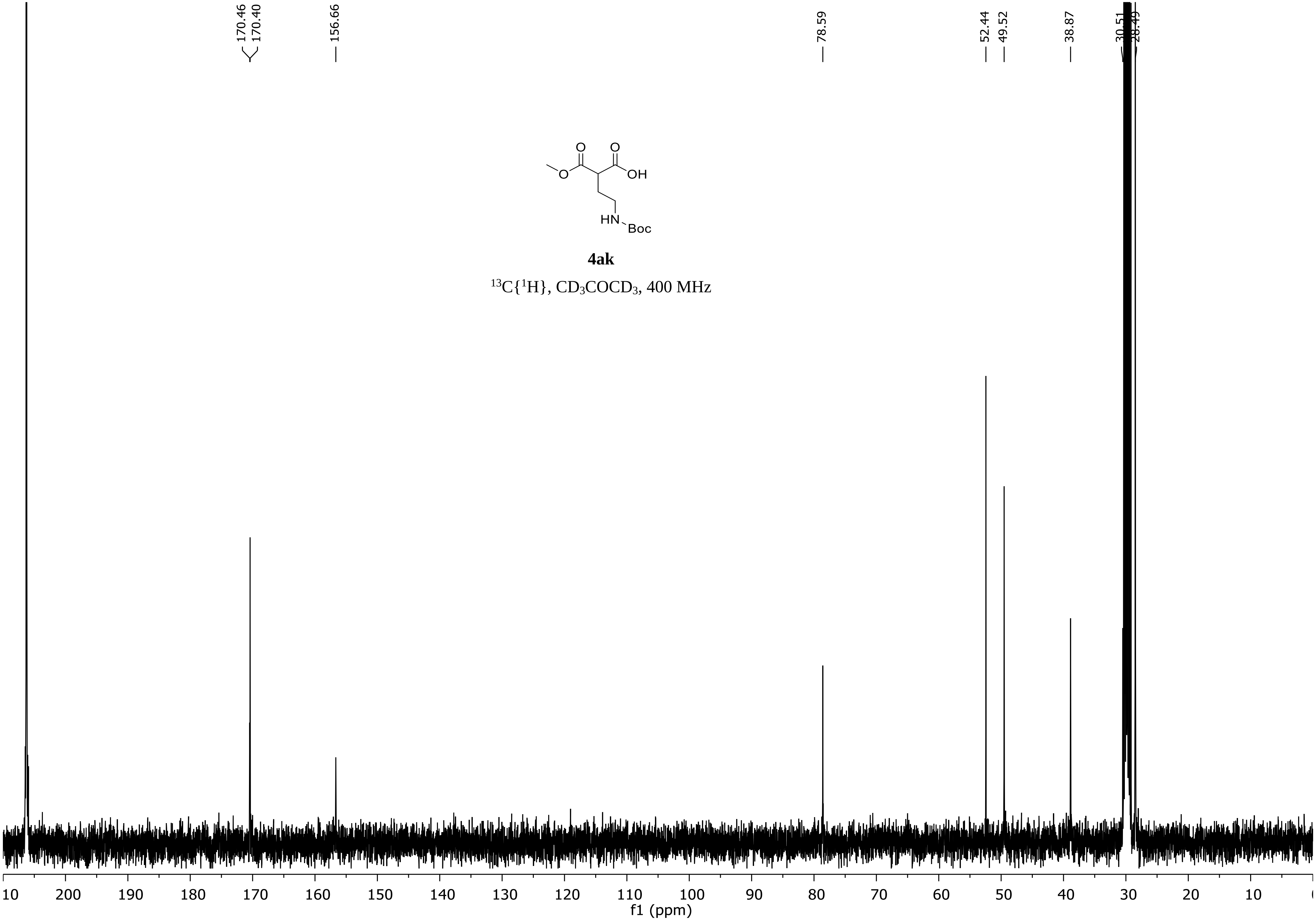

170.46
170.40
156.66
78.59
52.44
49.52
38.87
30.51
28.49
O
O
O
OH
HN
Boc
4ak
13C{1H}, CD3COCD3, 400 MHz
10 200 190 180 170 160 150 140 130 120 110 100 90 80 70 60 50 40 30 20 10
f1 (ppm)

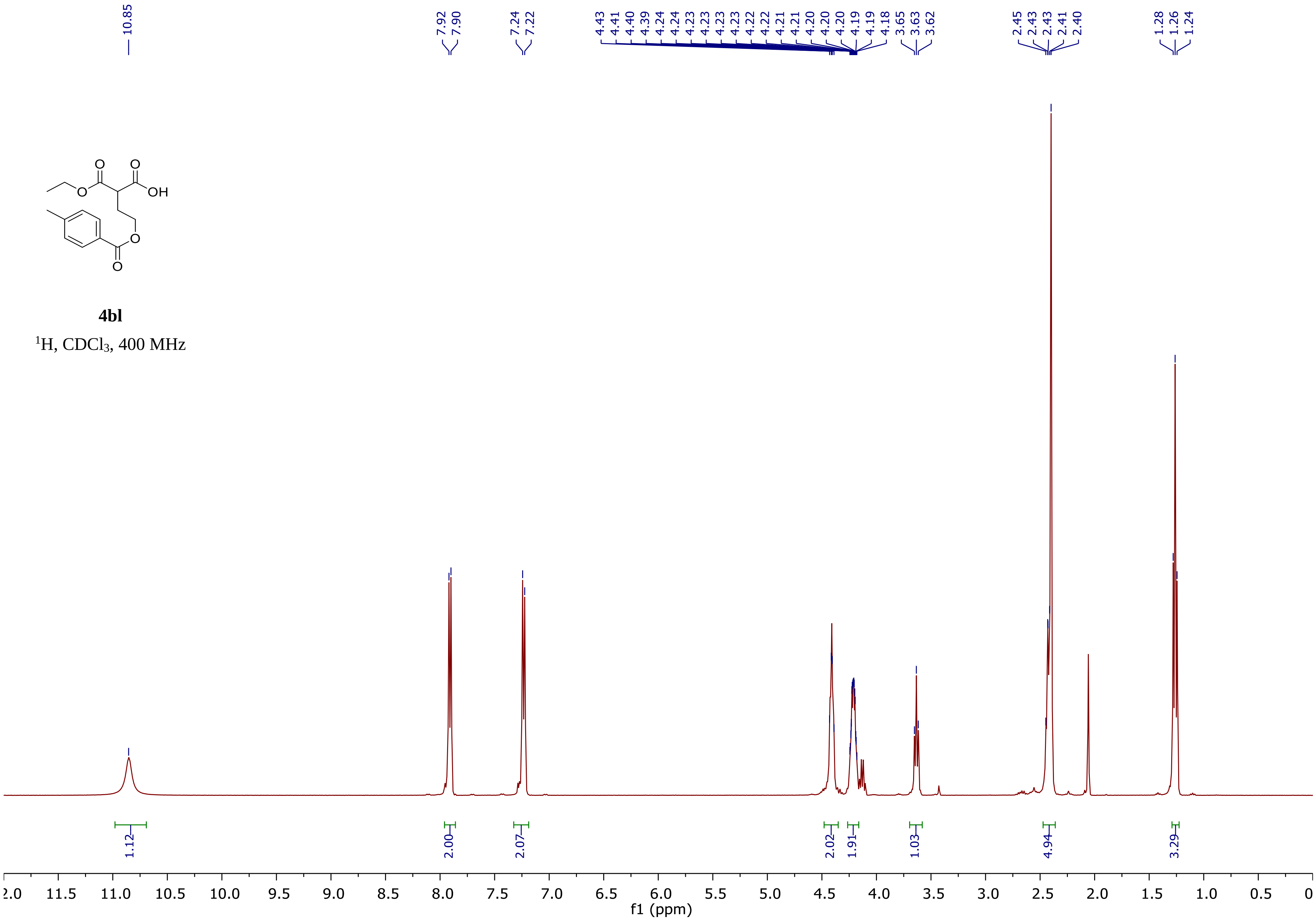

4bl

^{1}H, $CDCl_3$, 400 MHz

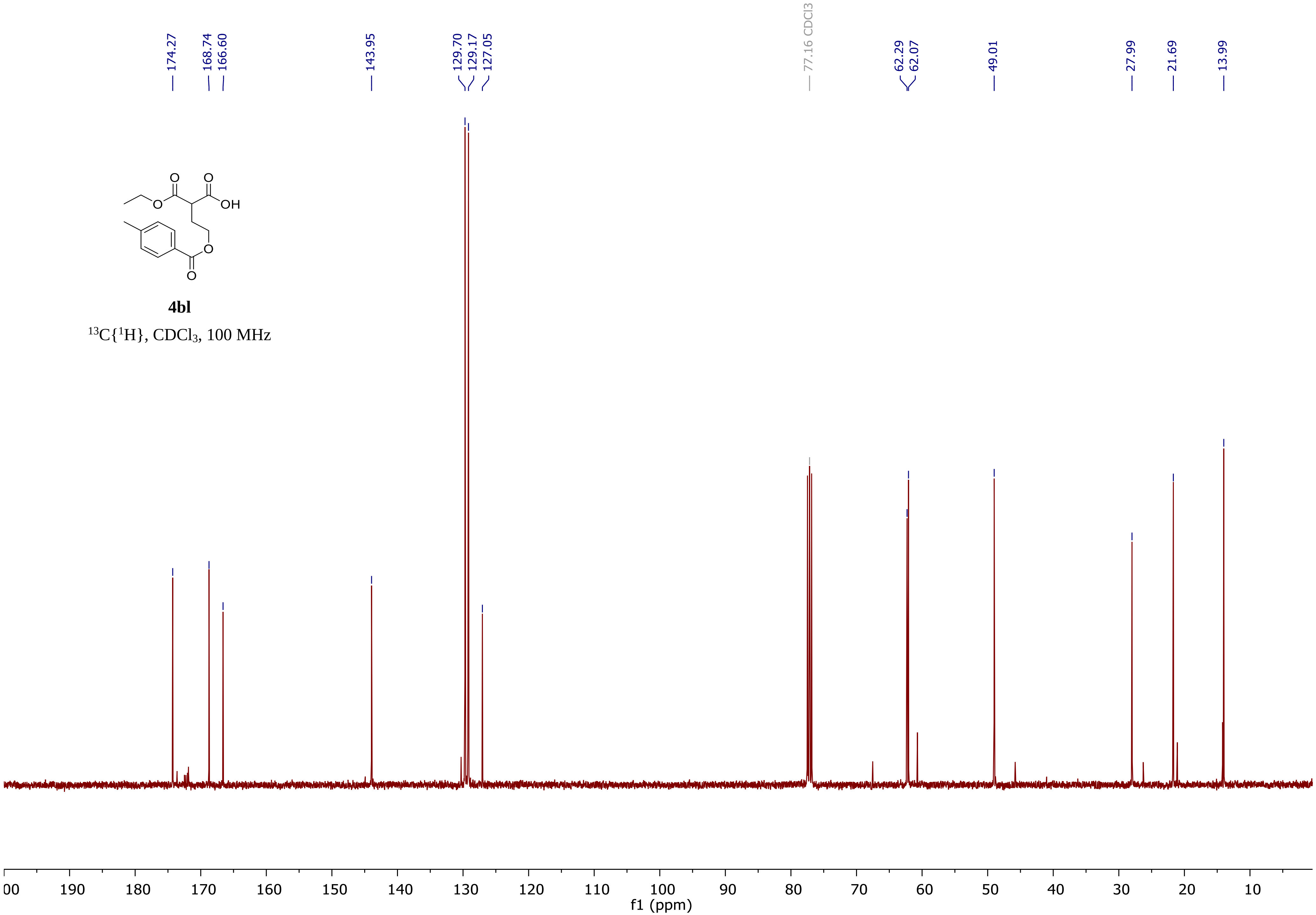

4bl

$^{13}C\{^1H\}$, $CDCl_3$, 100 MHz

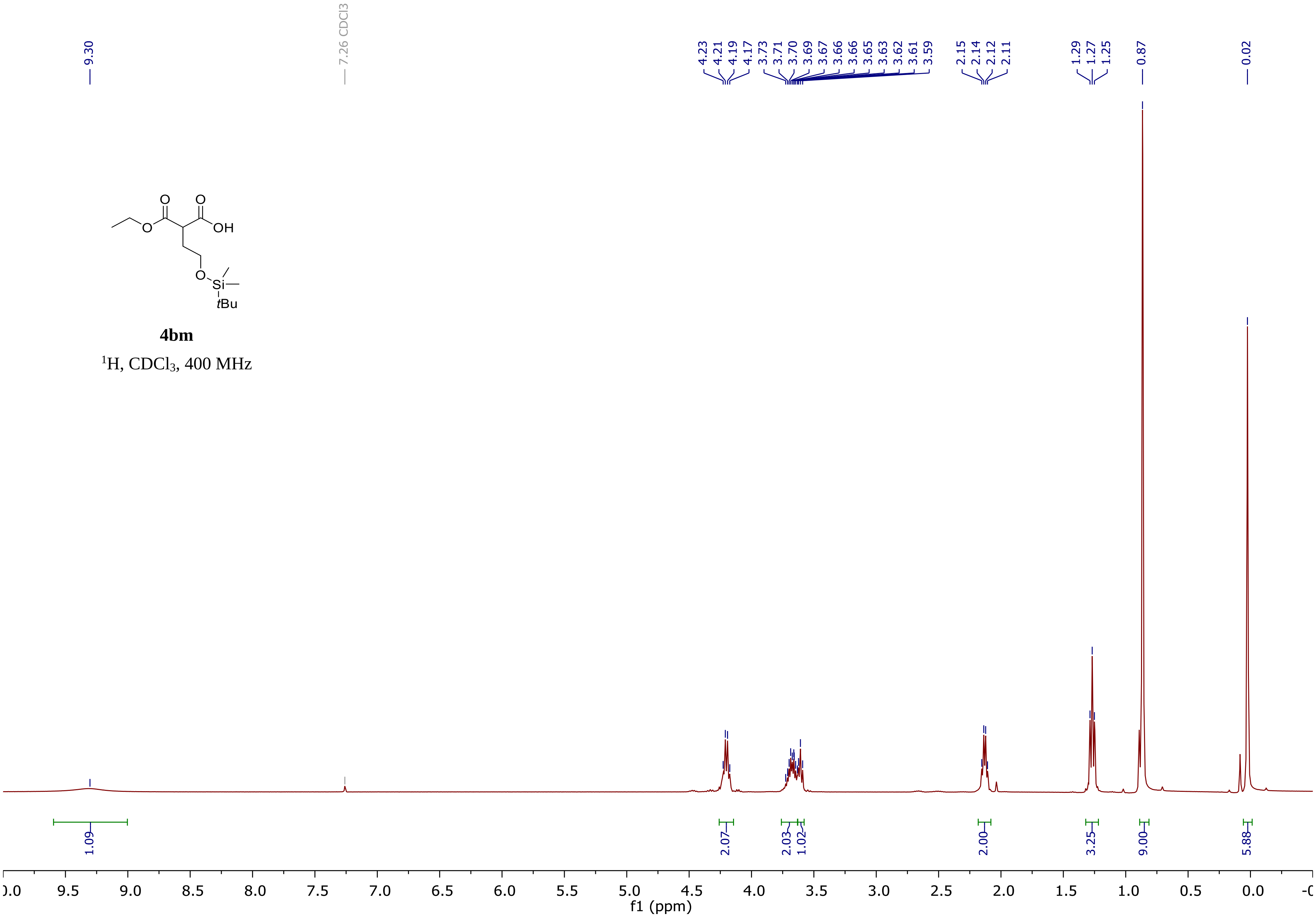

4bm

^{1}H, $CDCl_3$, 400 MHz

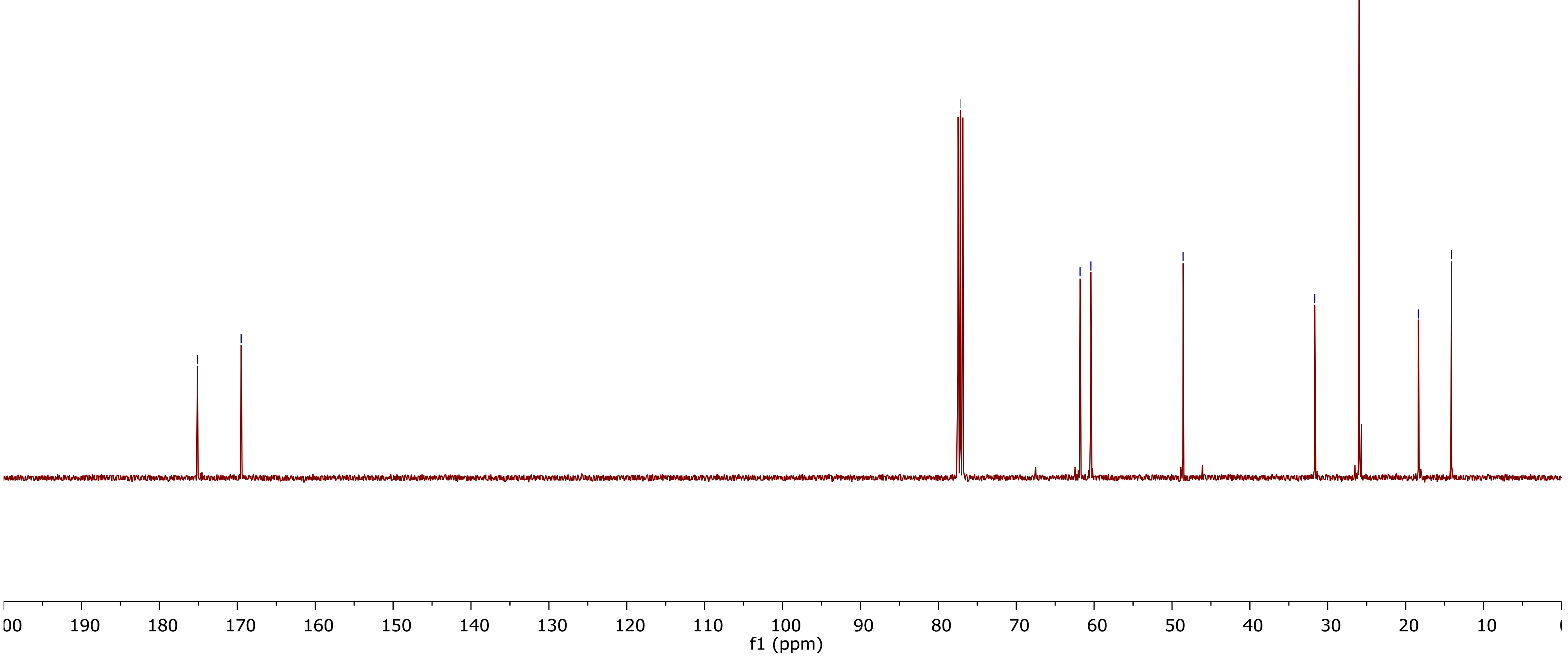

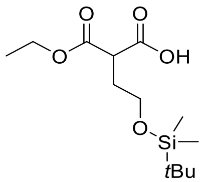

4bm

$^{13}C\{^{1}H\}$, $CDCl_3$, 100 MHz

4bn

^1H, $CDCl_3$, 400 MHz

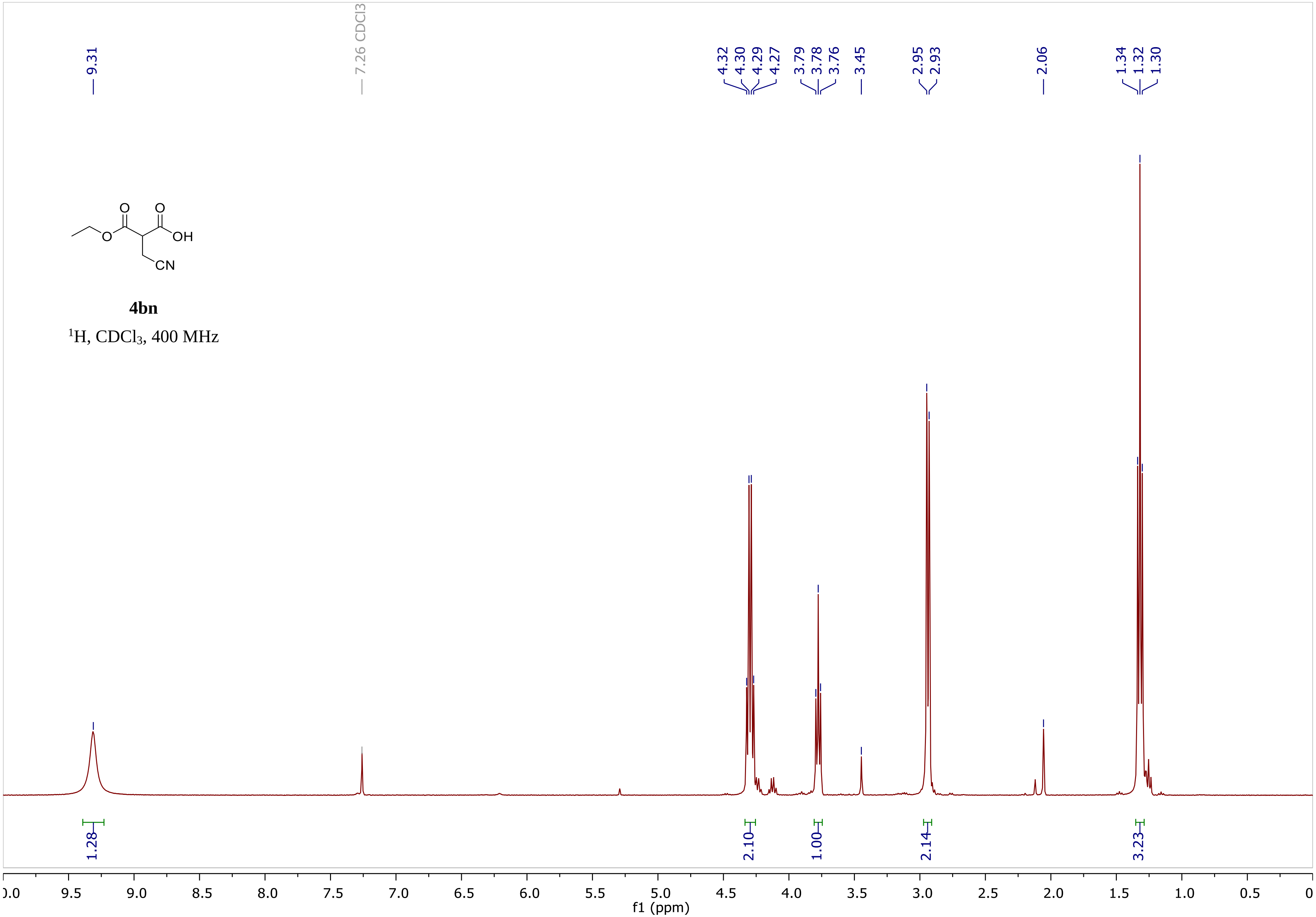

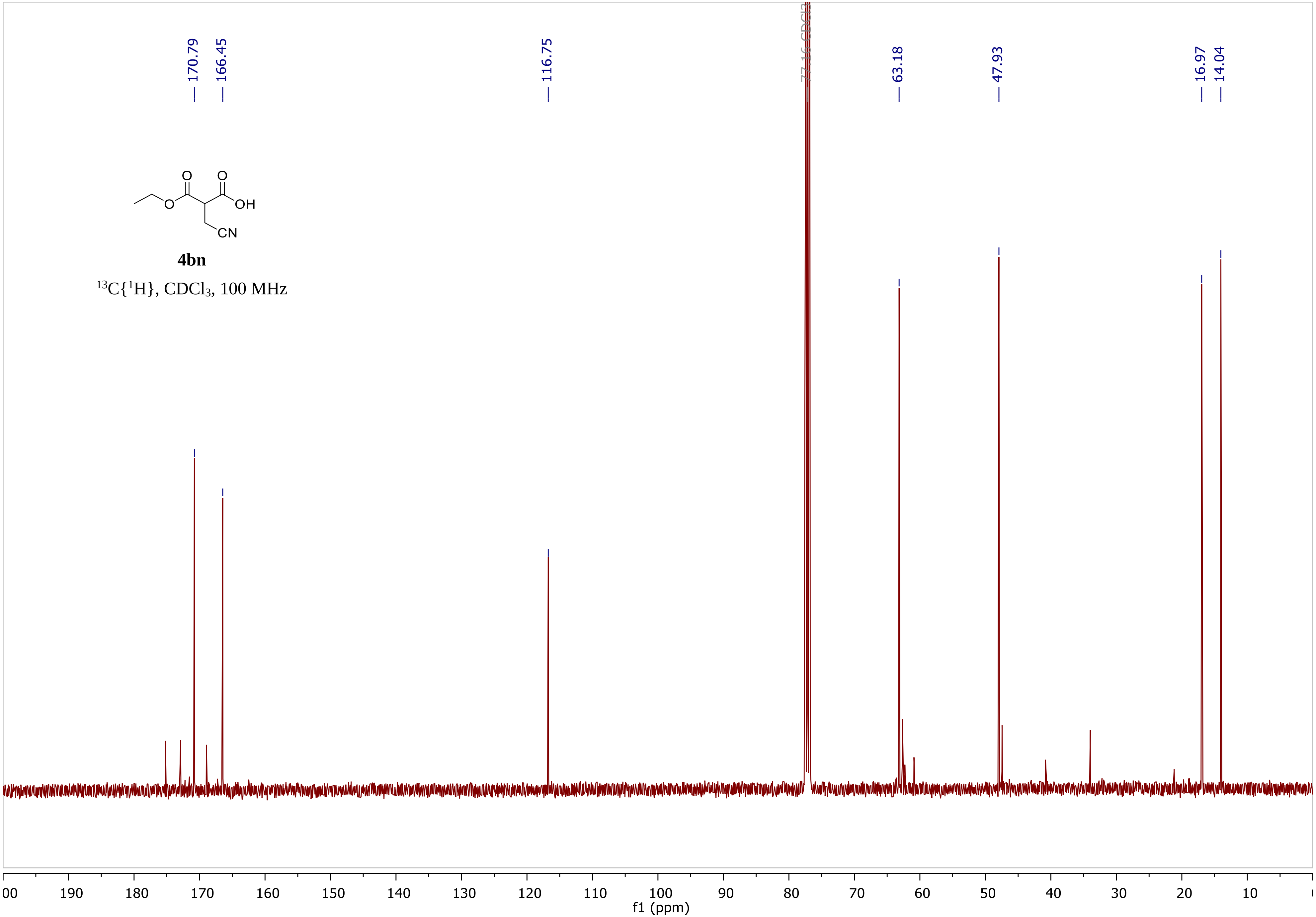
170.79
166.45
116.75
77.16 CDCl3
63.18
47.93
16.97
14.04
O
O
O
OH
CN
4bn
13C{1H}, CDCl3, 100 MHz
00
190
180
170
160
150
140
130
120
110
100
90
80
70
60
50
40
30
20
10
f1 (ppm)

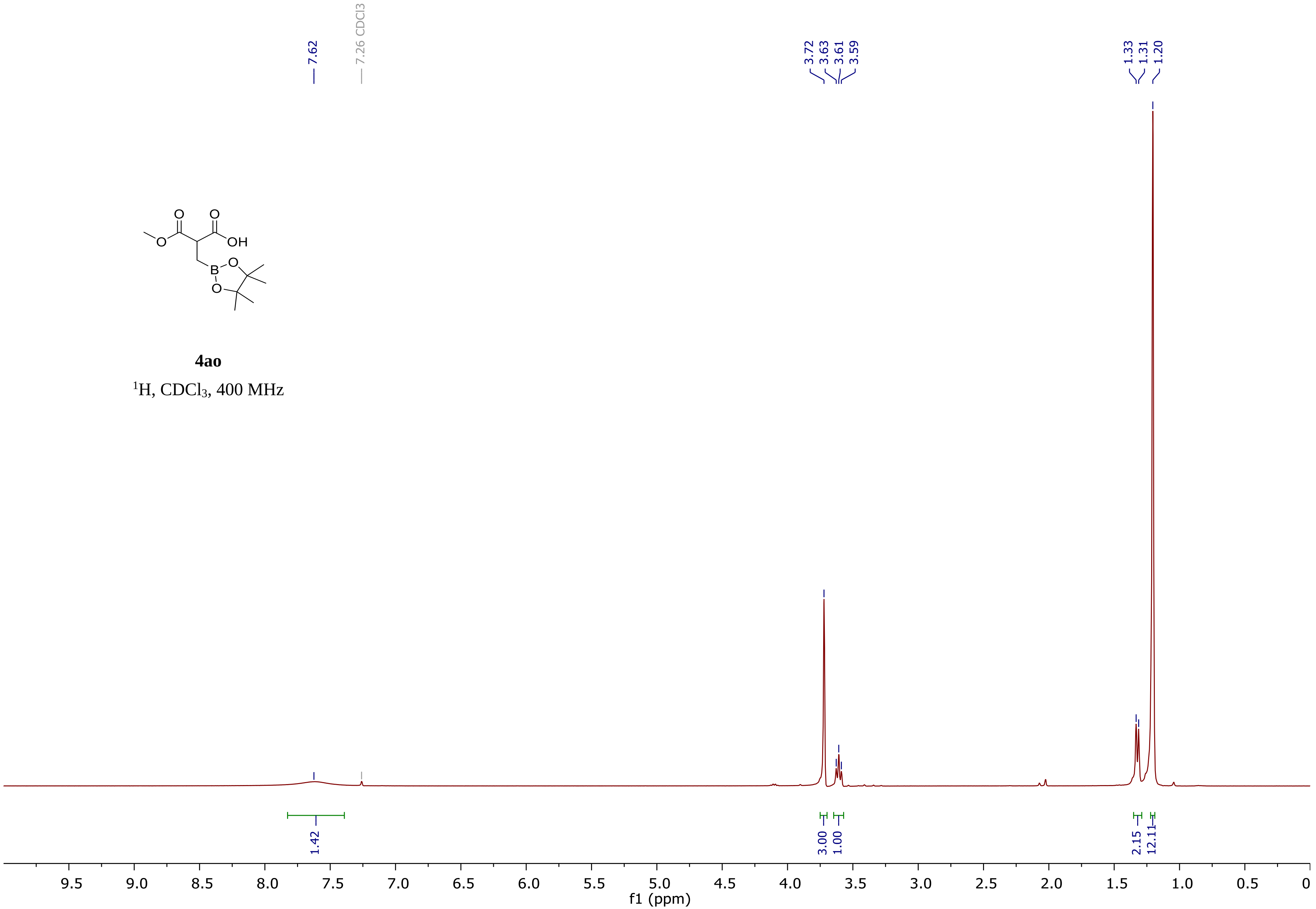

4ao

^{1}H, $CDCl_3$, 400 MHz

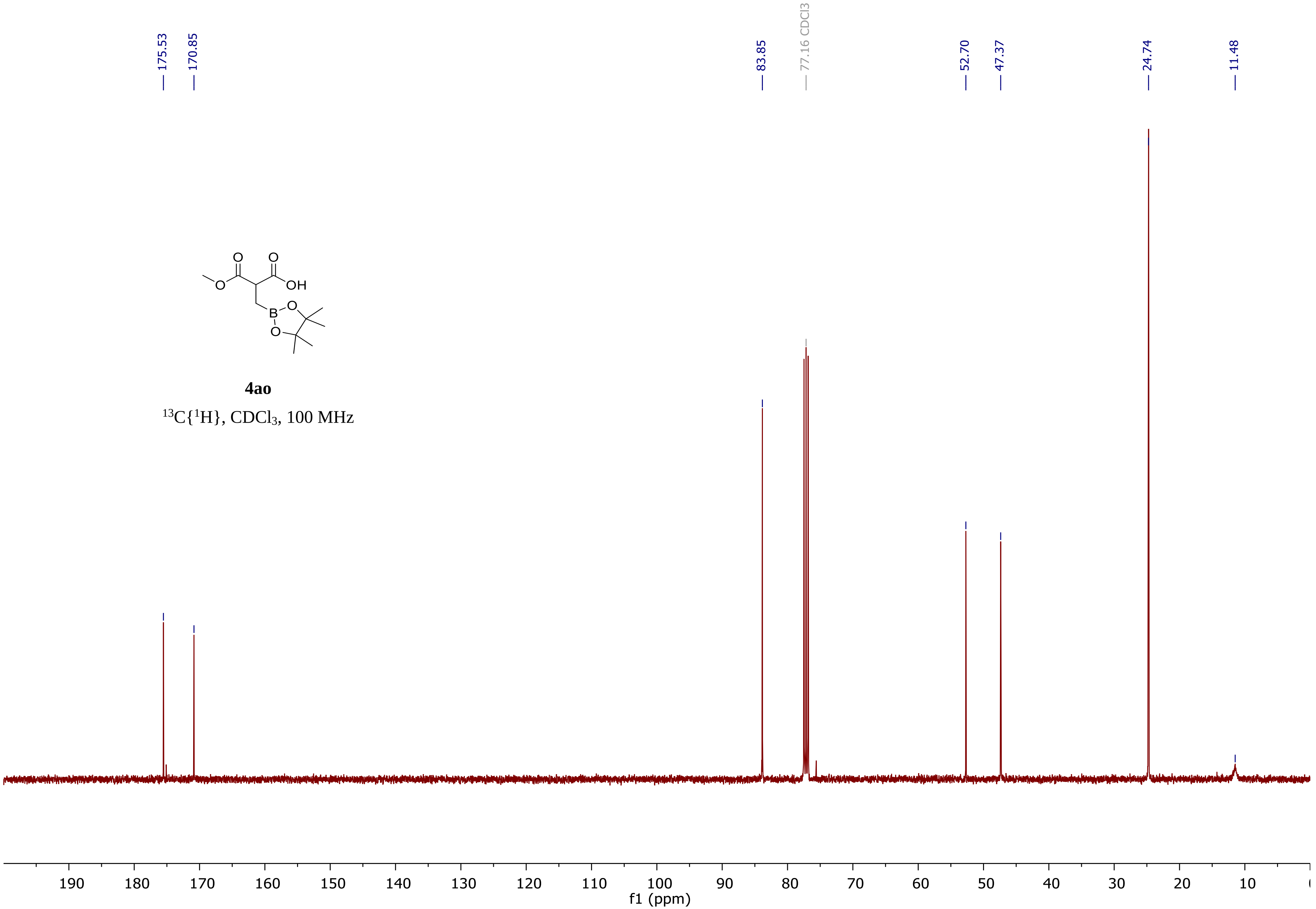

4ao

$^{13}C\{^{1}H\}$, $CDCl_3$, 100 MHz

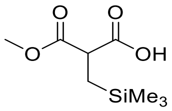

4ap

^{1}H, $CDCl_3$, 400 MHz

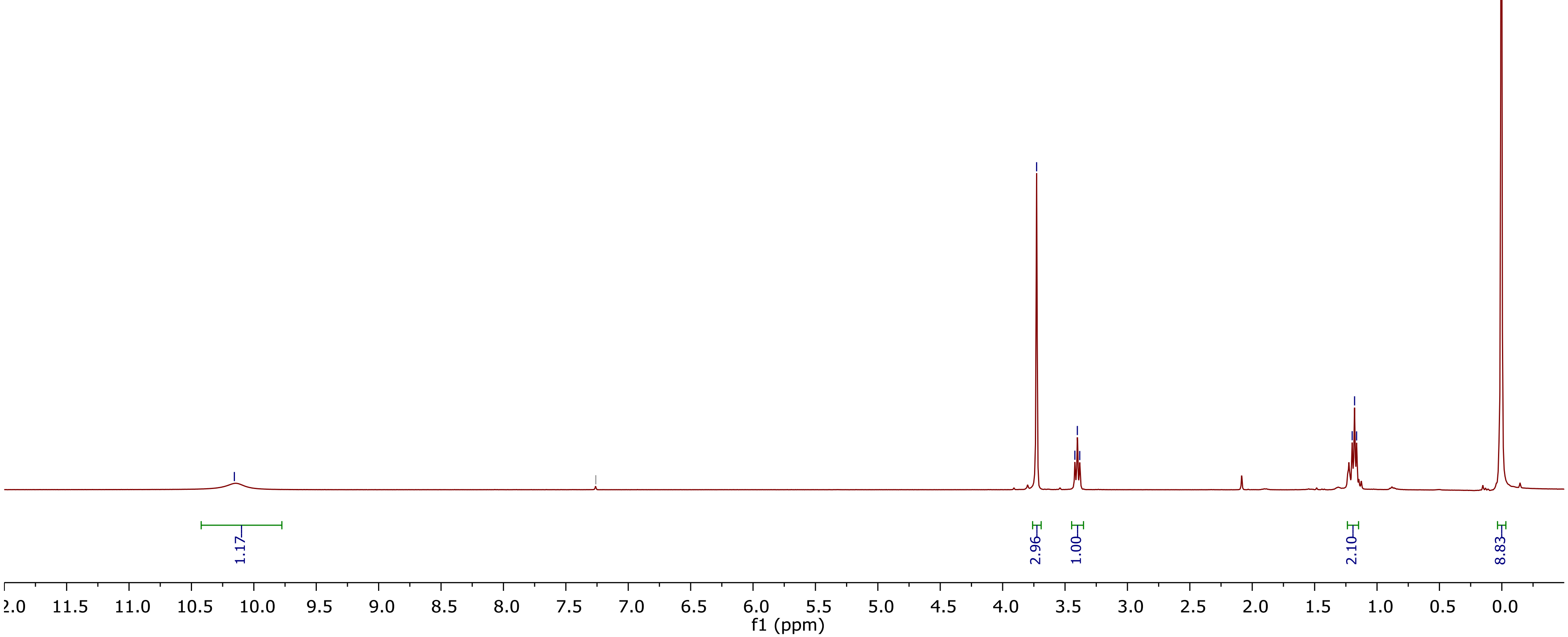

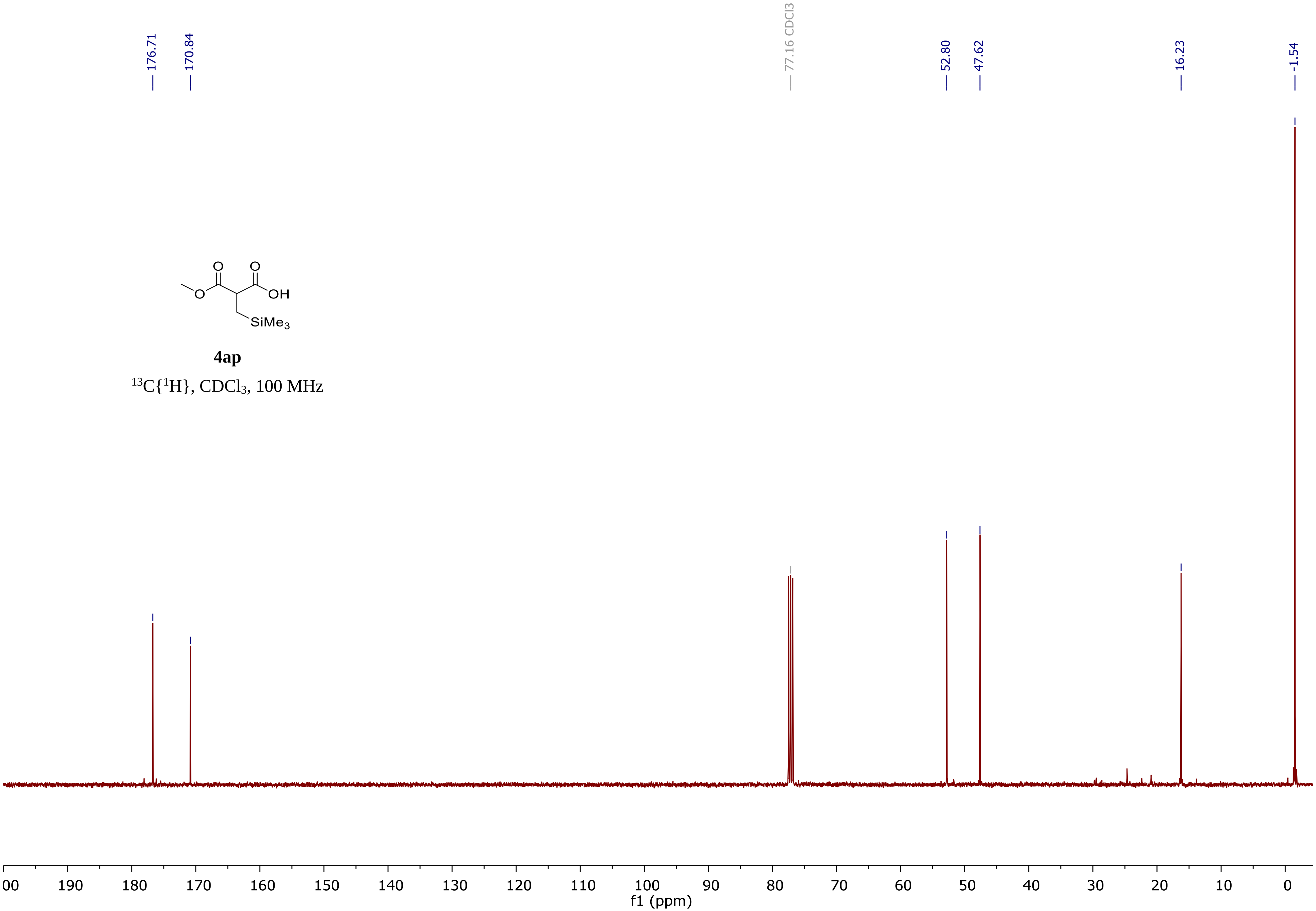

4ap

$^{13}C\{^1H\}$, $CDCl_3$, 100 MHz

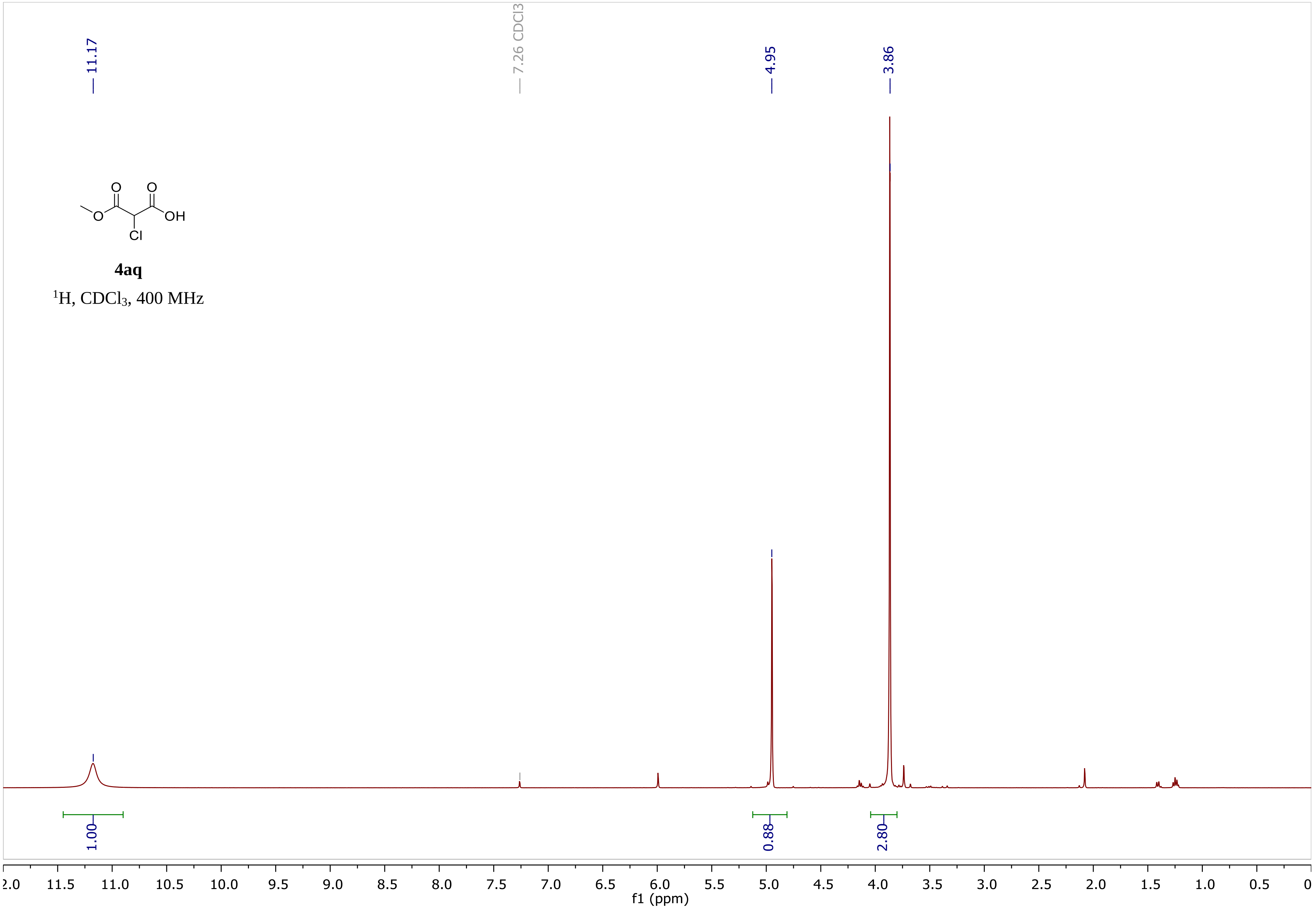

4aq

^1H, $CDCl_3$, 400 MHz

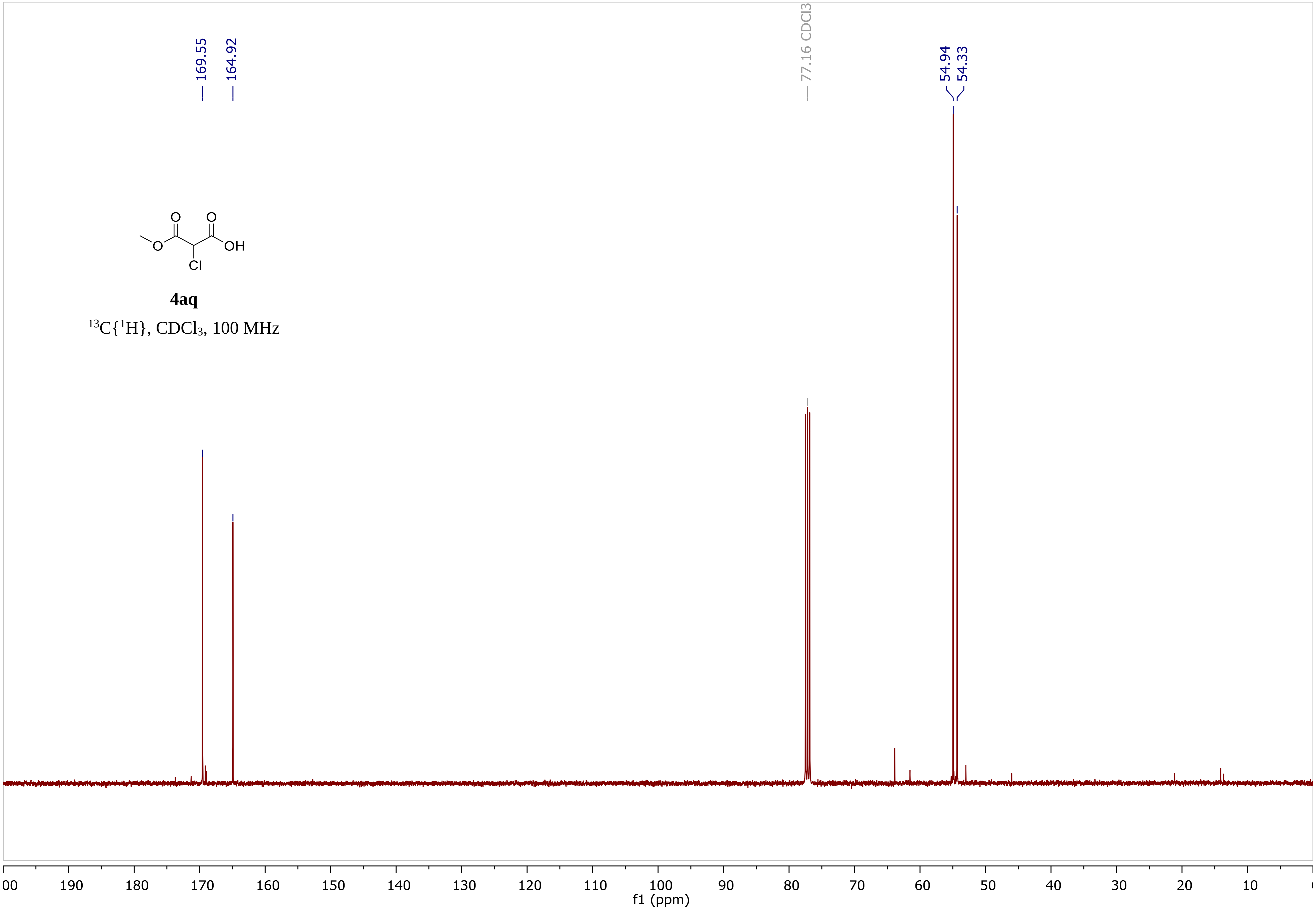
169.55
164.92
77.16 CDCl3
54.94
54.33
4aq
$^{13}C\{^1H\}$, $CDCl_3$, 100 MHz
00
190
180
170
160
150
140
130
120
110
100
90
80
70
60
50
40
30
20
10
f1 (ppm)

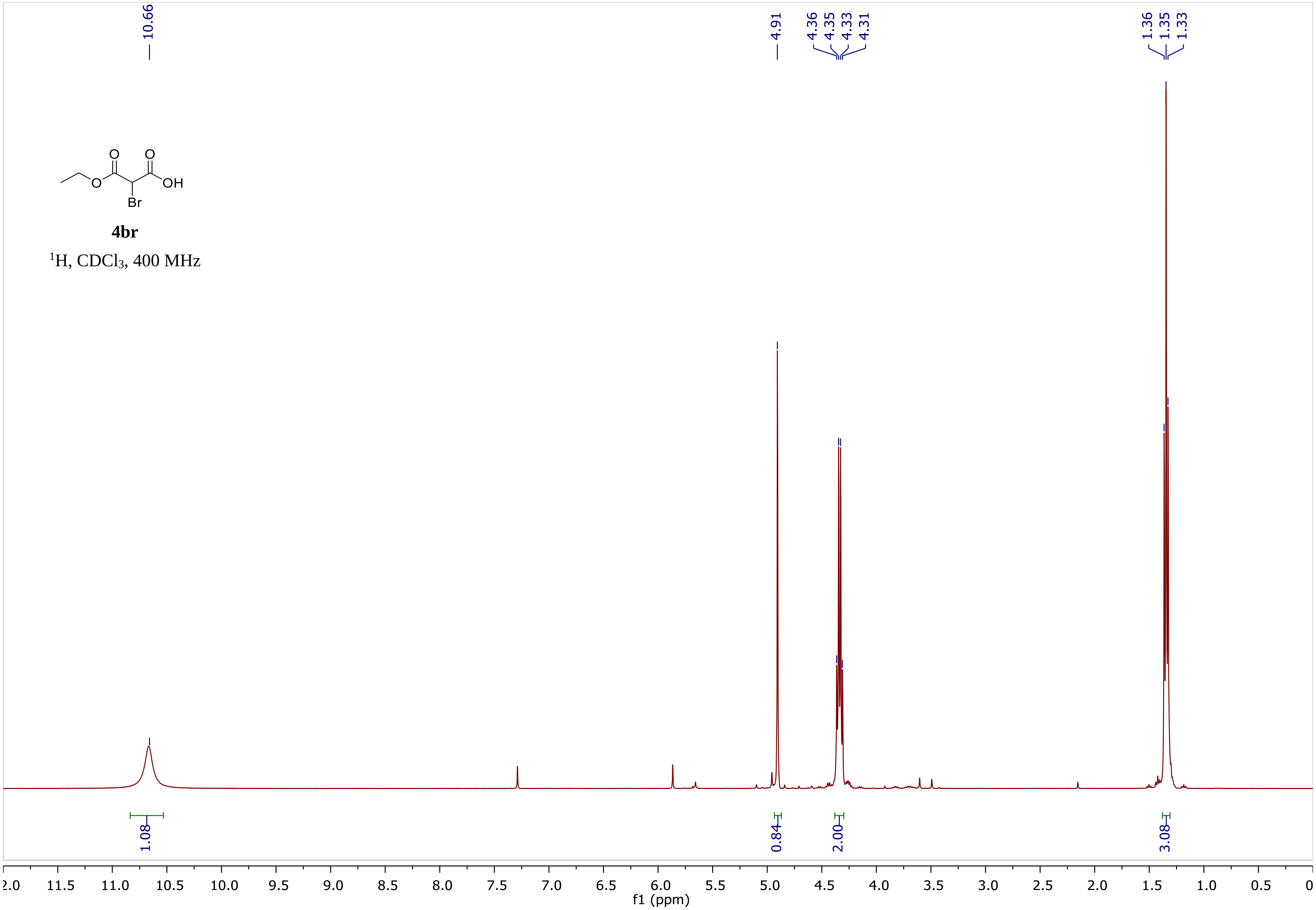
OH
O
O
O
Br
4br
1H, CDCl3, 400 MHz
10.66
4.91
4.36
4.35
4.33
4.31
1.36
1.35
1.33
1.08
0.84
2.00
3.08
2.0
11.5
11.0
10.5
10.0
9.5
9.0
8.5
8.0
7.5
7.0
6.5
6.0
5.5
5.0
4.5
4.0
3.5
3.0
2.5
2.0
1.5
1.0
0.5
0
f1 (ppm)

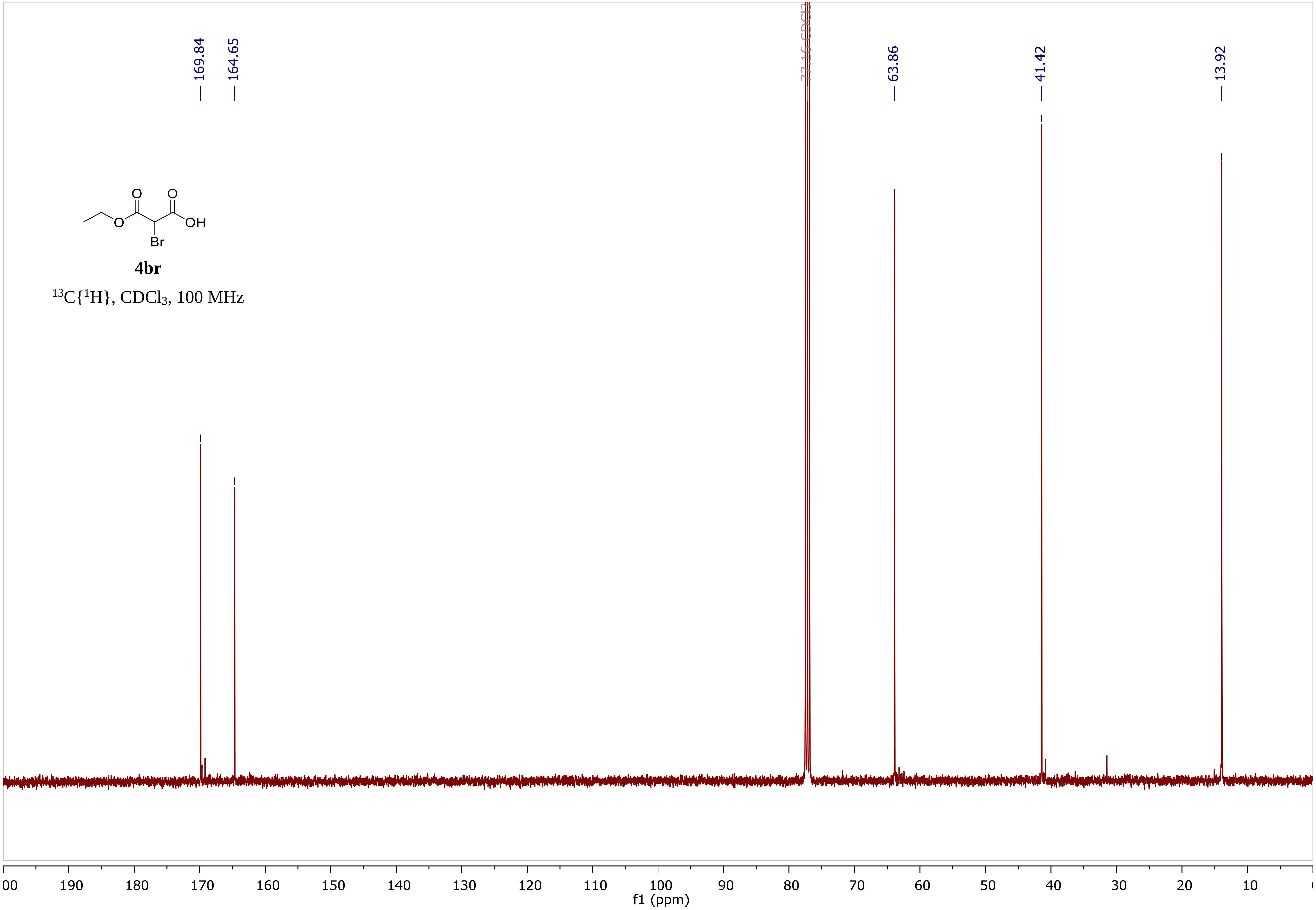

4br

$^{13}C\{^{1}H\}$, $CDCl_3$, 100 MHz

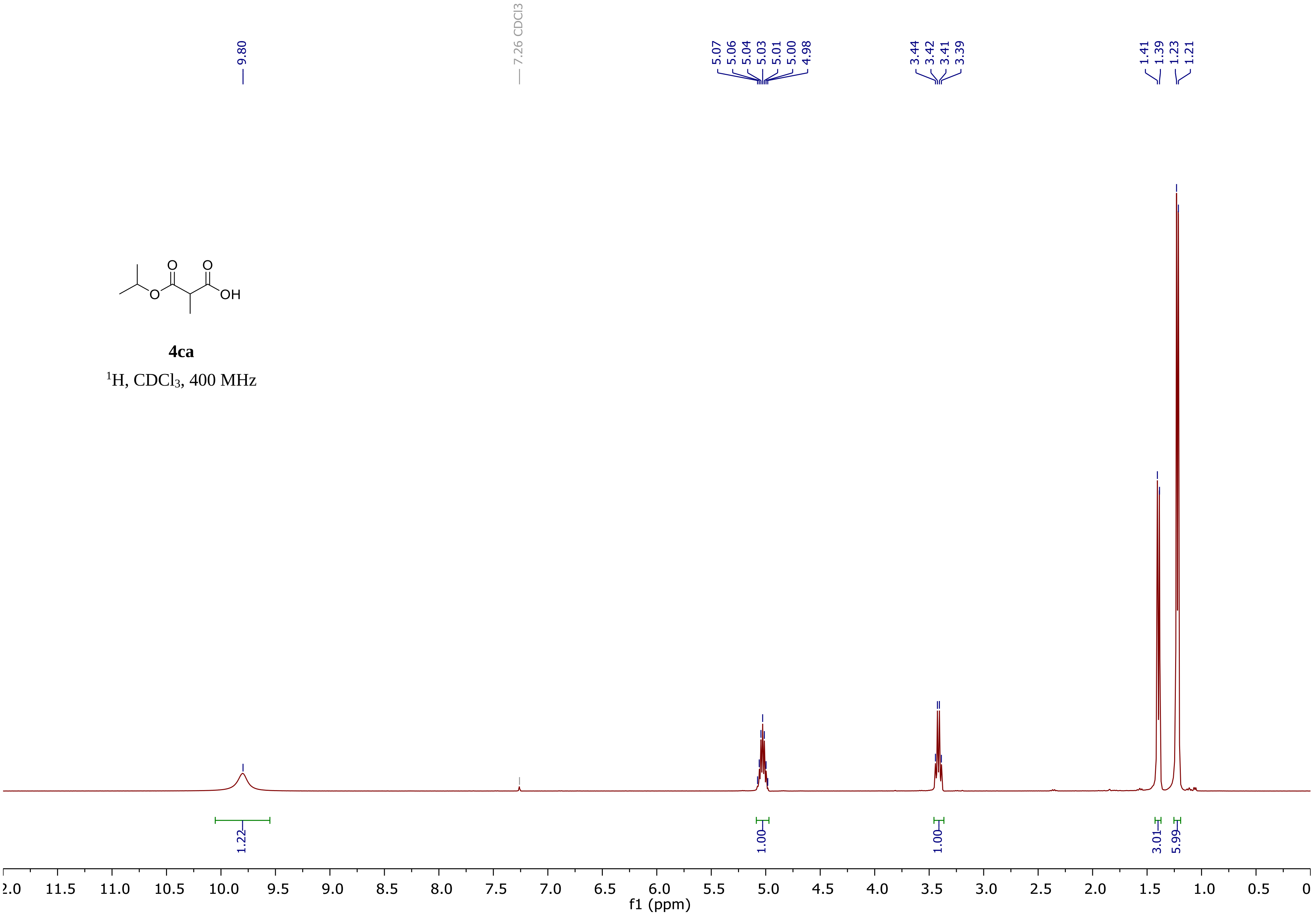

9.80
7.26 CDCl3
5.07
5.06
5.04
5.03
5.01
5.00
4.98
3.44
3.42
3.41
3.39
1.41
1.39
1.23
1.21
O
O
O
OH
4ca
^{1}H, $CDCl_3$, 400 MHz
1.22
1.00
1.00
3.01
5.99
2.0
11.5
11.0
10.5
10.0
9.5
9.0
8.5
8.0
7.5
7.0
6.5
6.0
5.5
5.0
4.5
4.0
3.5
3.0
2.5
2.0
1.5
1.0
0.5
0
f1 (ppm)

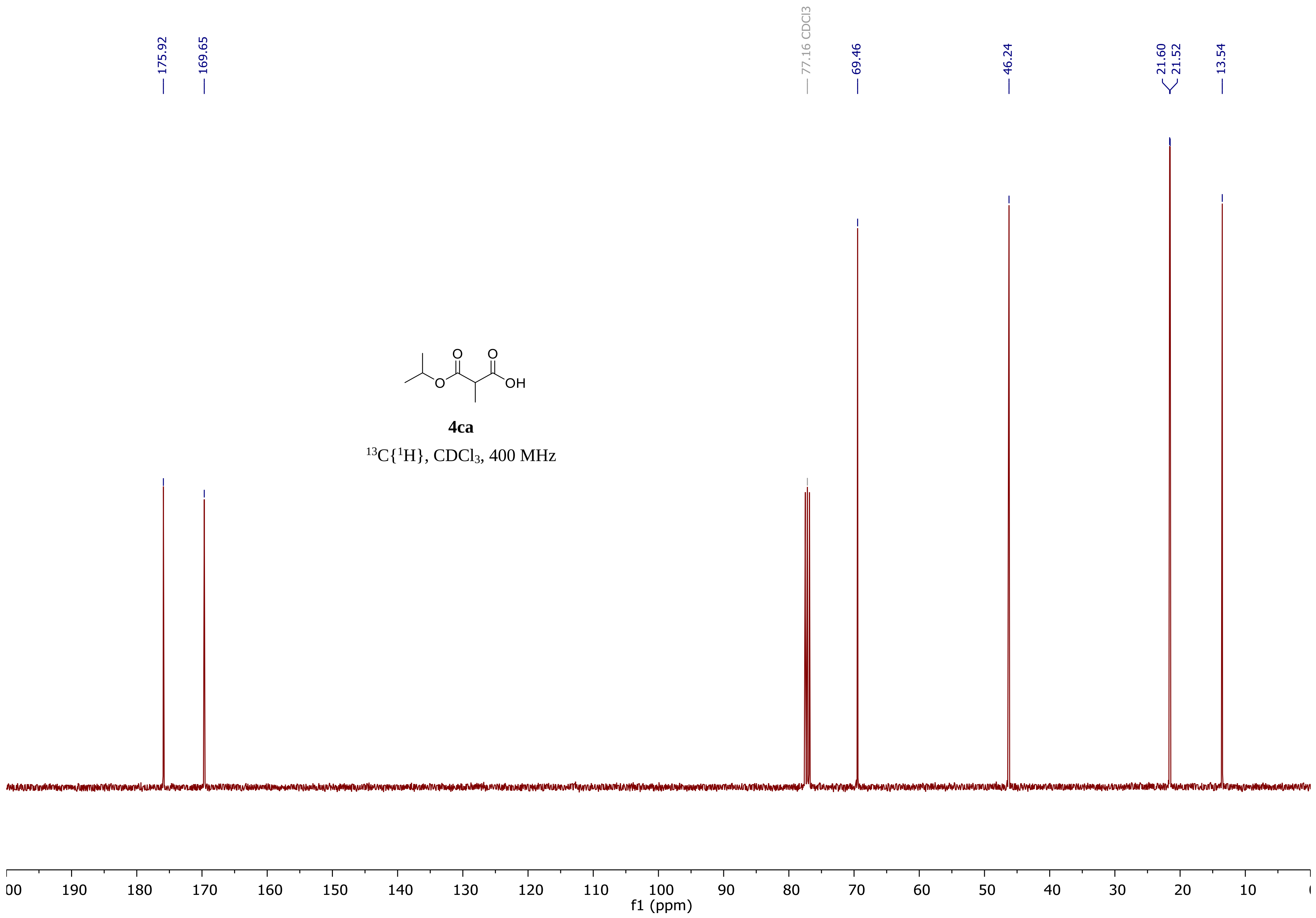

175.92
169.65
77.16 CDCl3
69.46
46.24
21.60
21.52
13.54
OH
O
O
O
4ca
$^{13}C\{^1H\}$, $CDCl_3$, 400 MHz
00
190
180
170
160
150
140
130
120
110
100
90
80
70
60
50
40
30
20
10
f1 (ppm)

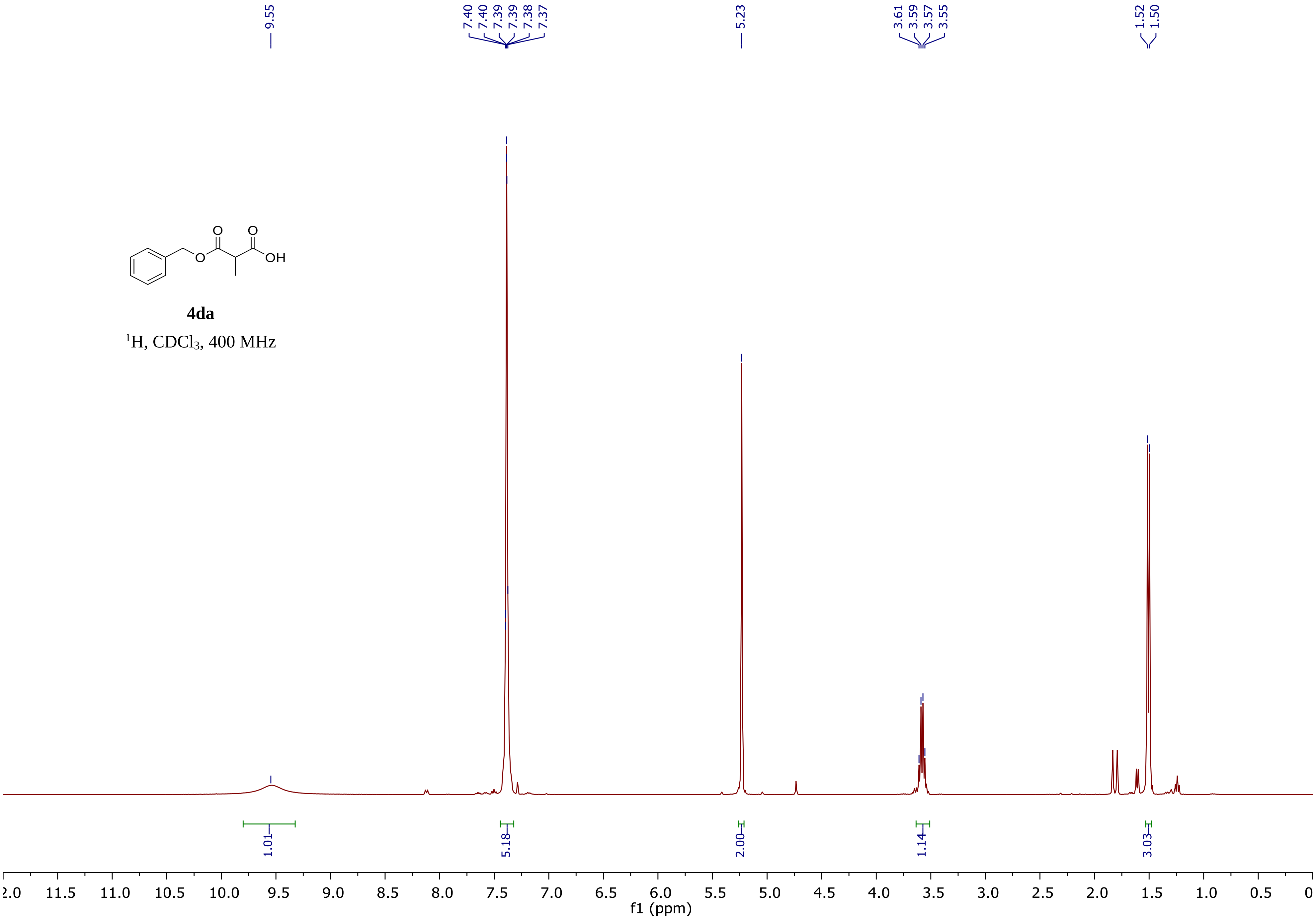

4da

^1H, $CDCl_3$, 400 MHz

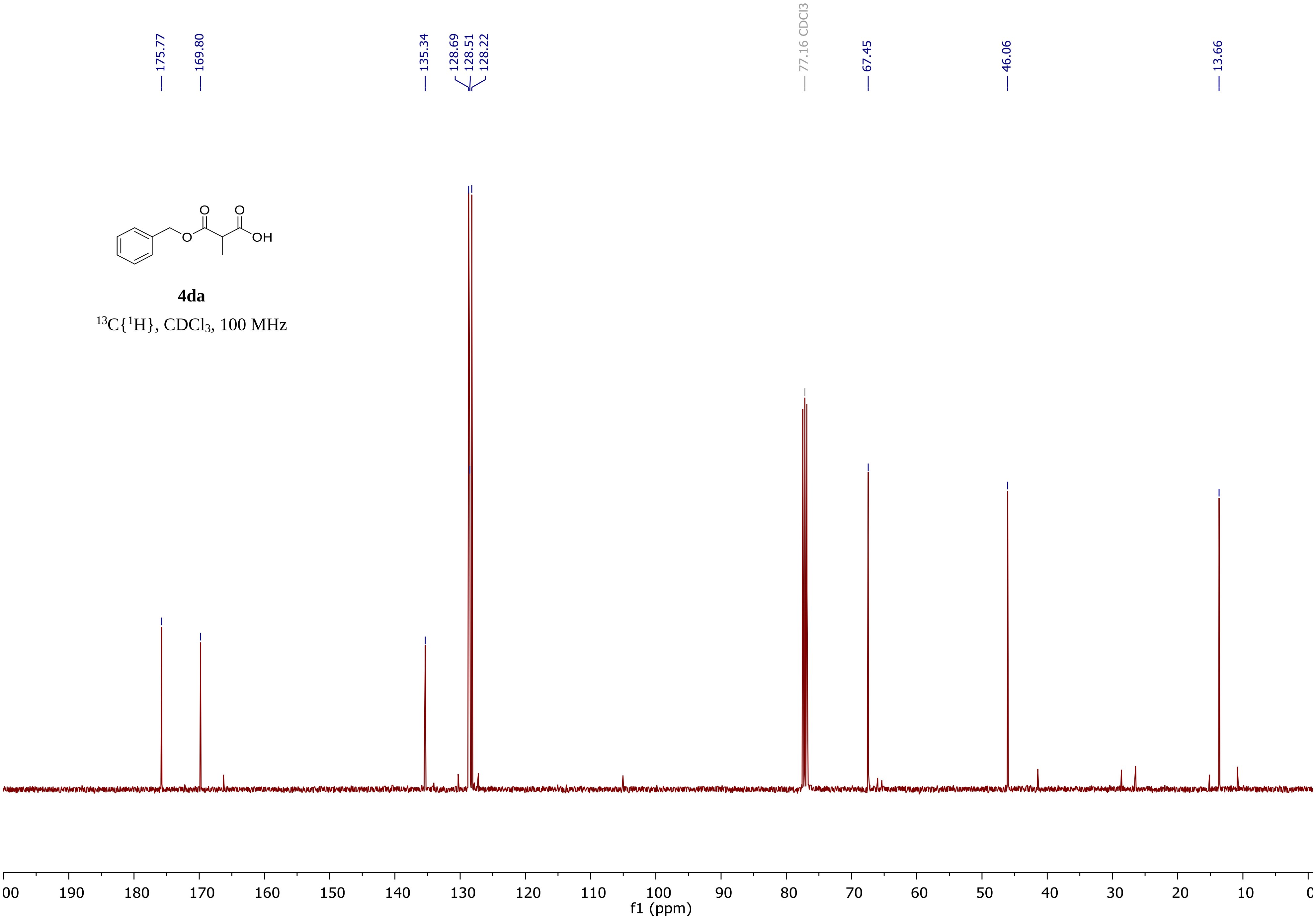
175.77
169.80
135.34
128.69
128.51
128.22
77.16 CDCl3
67.45
46.06
13.66
O
O
O
OH
4da
$^{13}C\{^{1}H\}$, $CDCl_3$, 100 MHz
00
190
180
170
160
150
140
130
120
110
100
90
80
70
60
50
40
30
20
10
0
f1 (ppm)

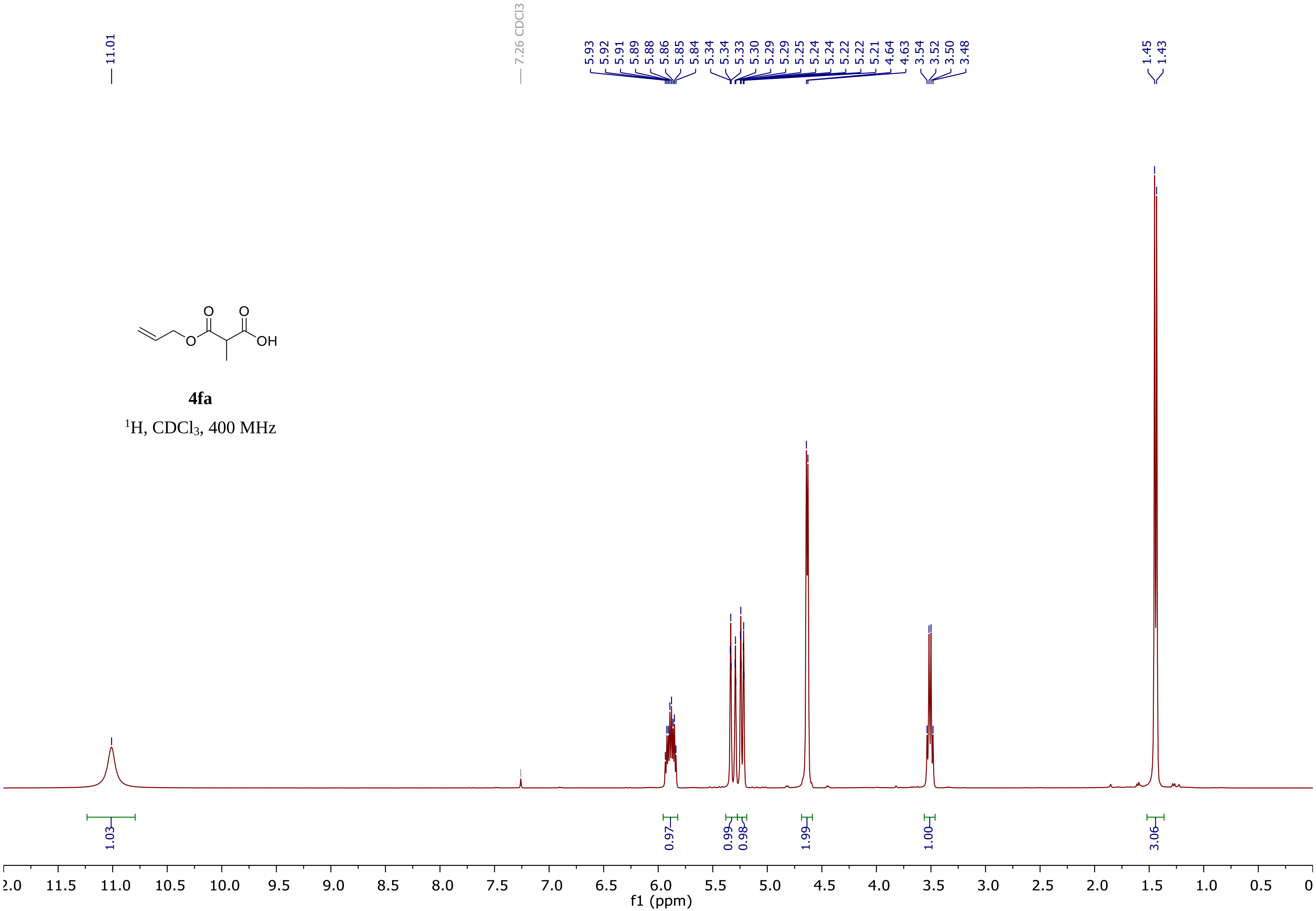

4fa

^{1}H, $CDCl_3$, 400 MHz

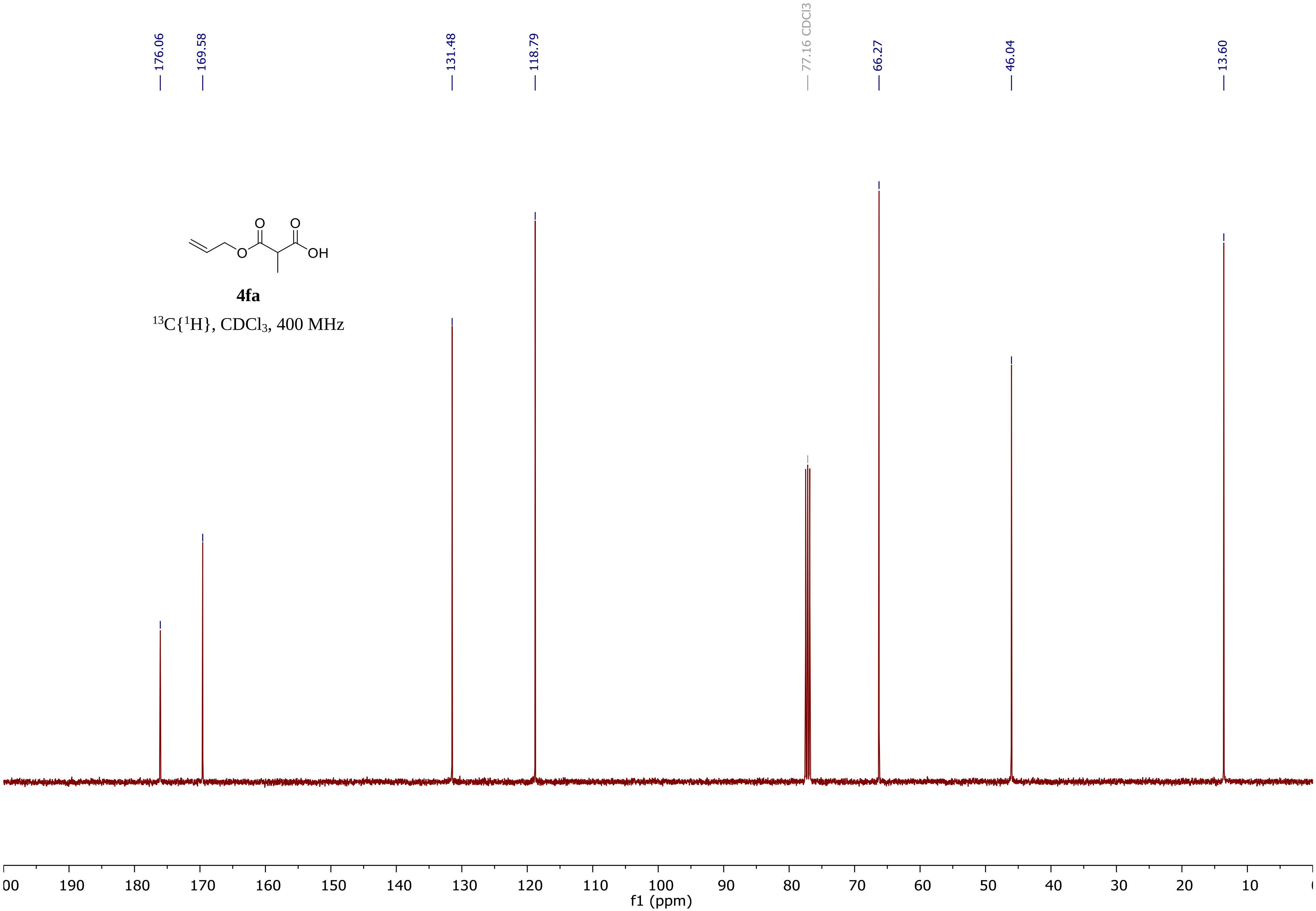
176.06
169.58
131.48
118.79
77.16 CDCl3
66.27
46.04
13.60
O
O
O
OH
4fa
$^{13}C\{^1H\}$, $CDCl_3$, 400 MHz
00
190
180
170
160
150
140
130
120
110
100
90
80
70
60
50
40
30
20
10
f1 (ppm)

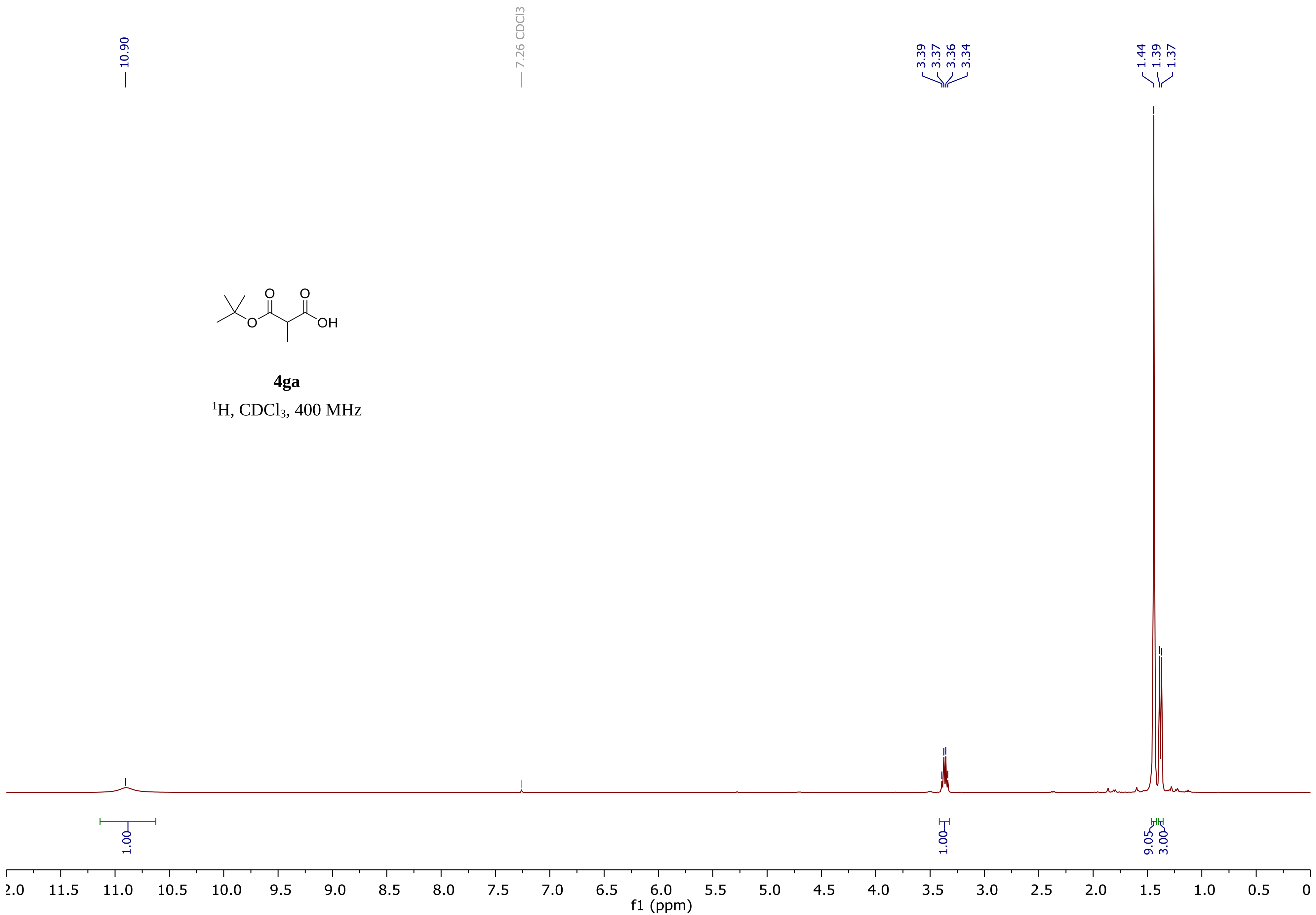

4ga

^{1}H, $CDCl_3$, 400 MHz

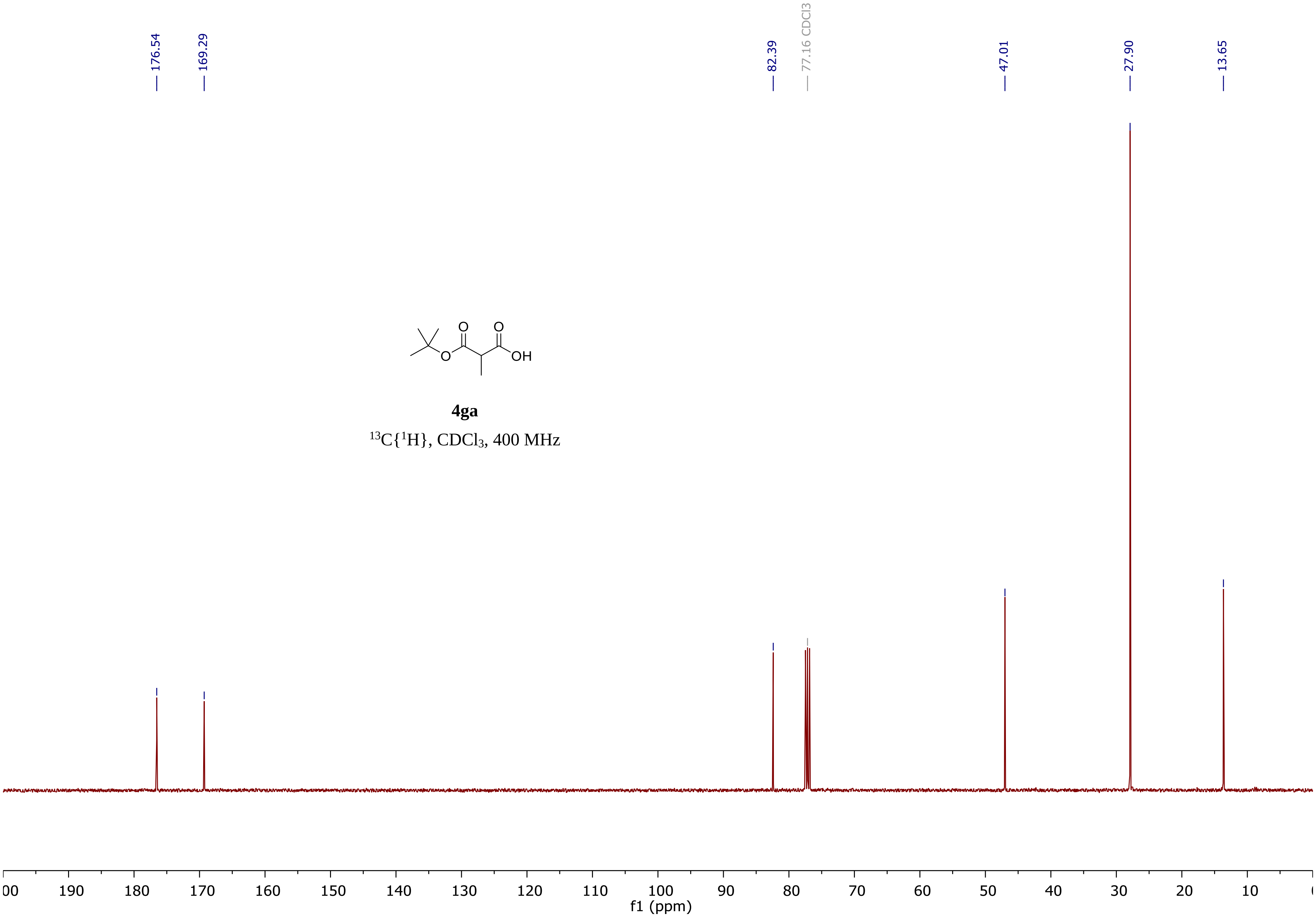

4ga

$^{13}C\{^{1}H\}$, $CDCl_3$, 400 MHz

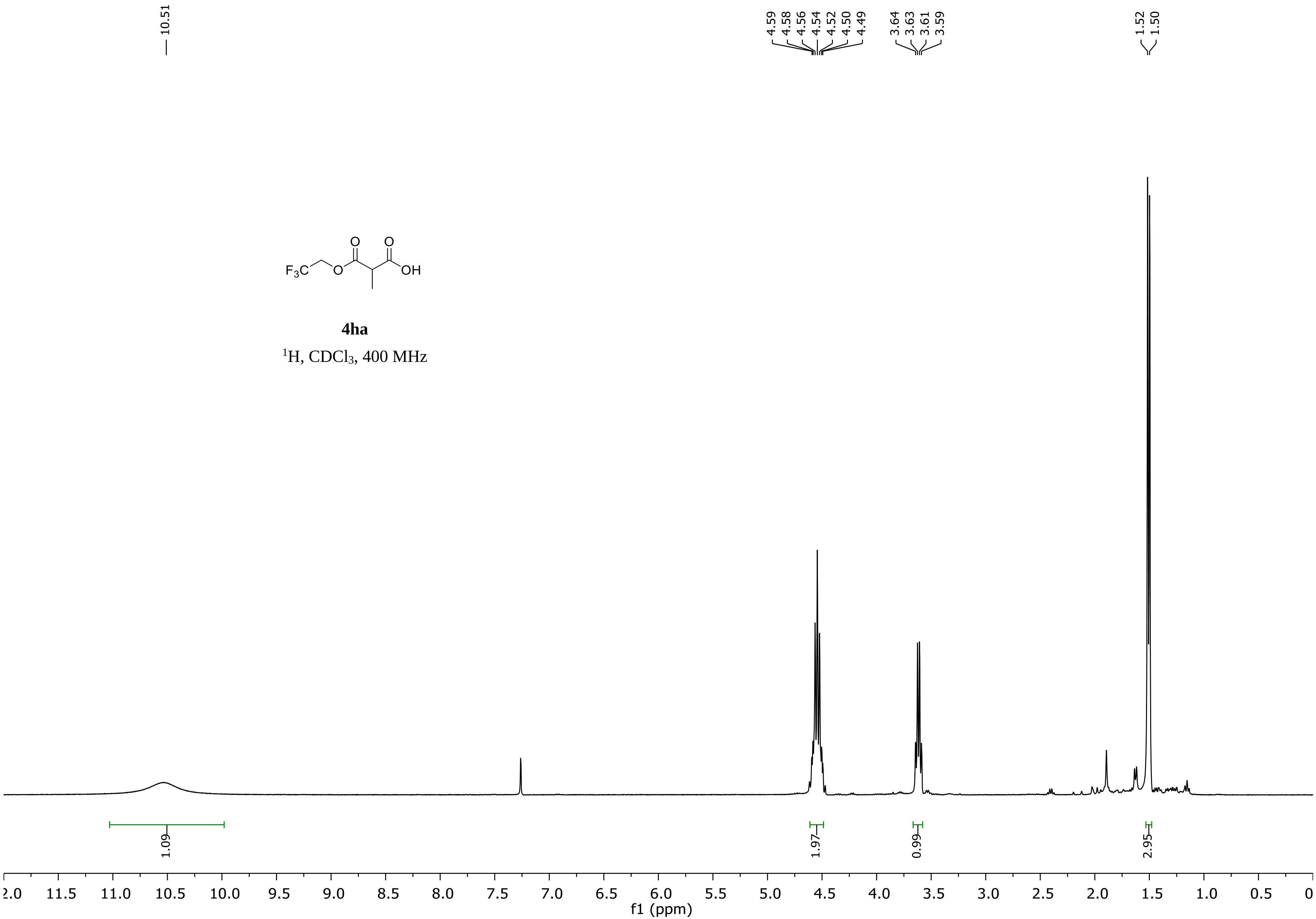

10.51
4.59 4.58 4.56 4.54 4.52 4.50 4.49
3.64 3.63 3.61 3.59
1.52 1.50
F3C O O O OH
4ha
1H, CDCl3, 400 MHz
1.09
1.97
0.99
2.95
2.0 11.5 11.0 10.5 10.0 9.5 9.0 8.5 8.0 7.5 7.0 6.5 6.0 5.5 5.0 4.5 4.0 3.5 3.0 2.5 2.0 1.5 1.0 0.5 0
f1 (ppm)

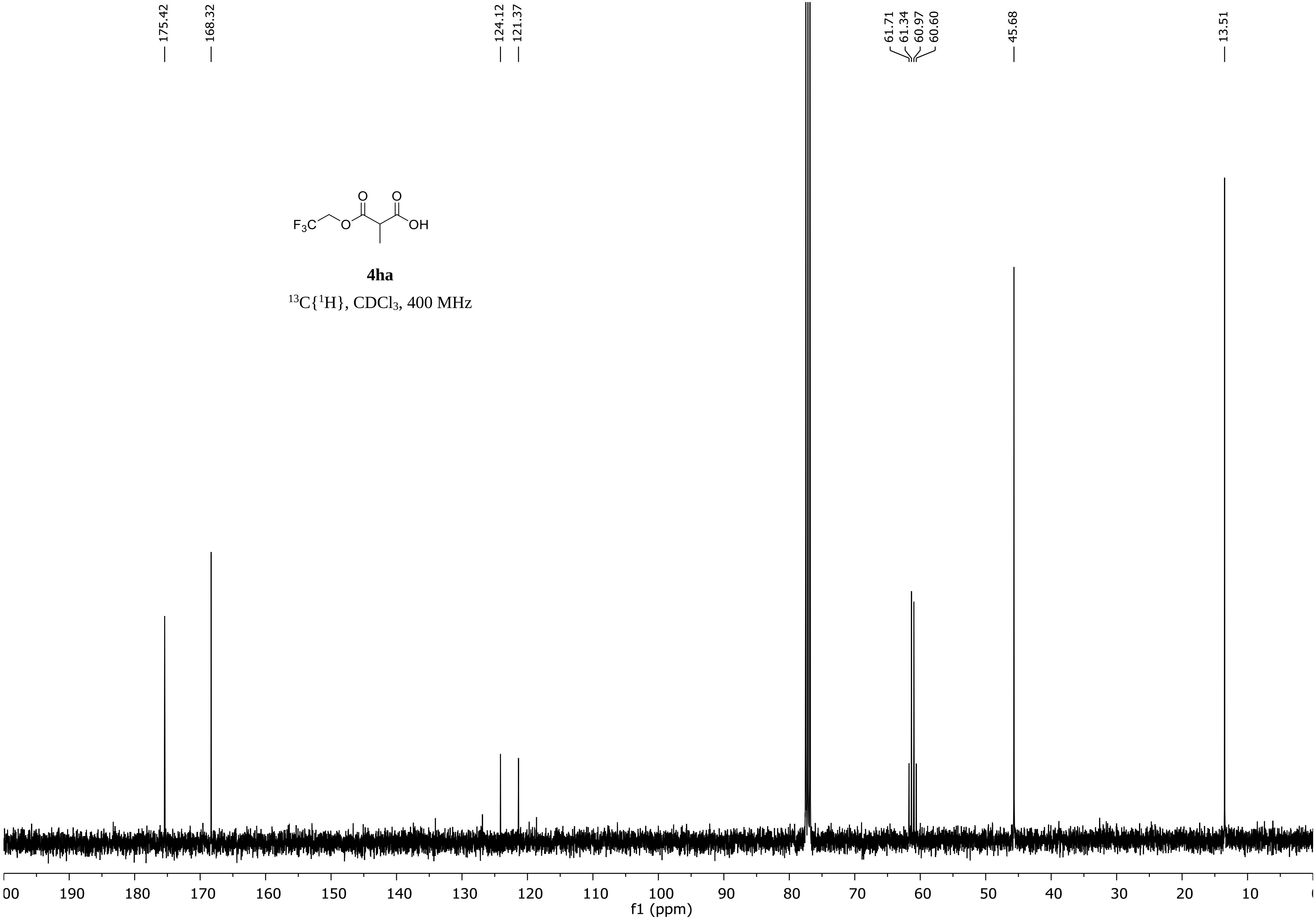

4ha

$^{13}C\{^{1}H\}$, $CDCl_3$, 400 MHz

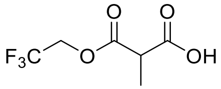

4ha

$^{19}F\{^1H\}$, $CDCl_3$, 377 MHz

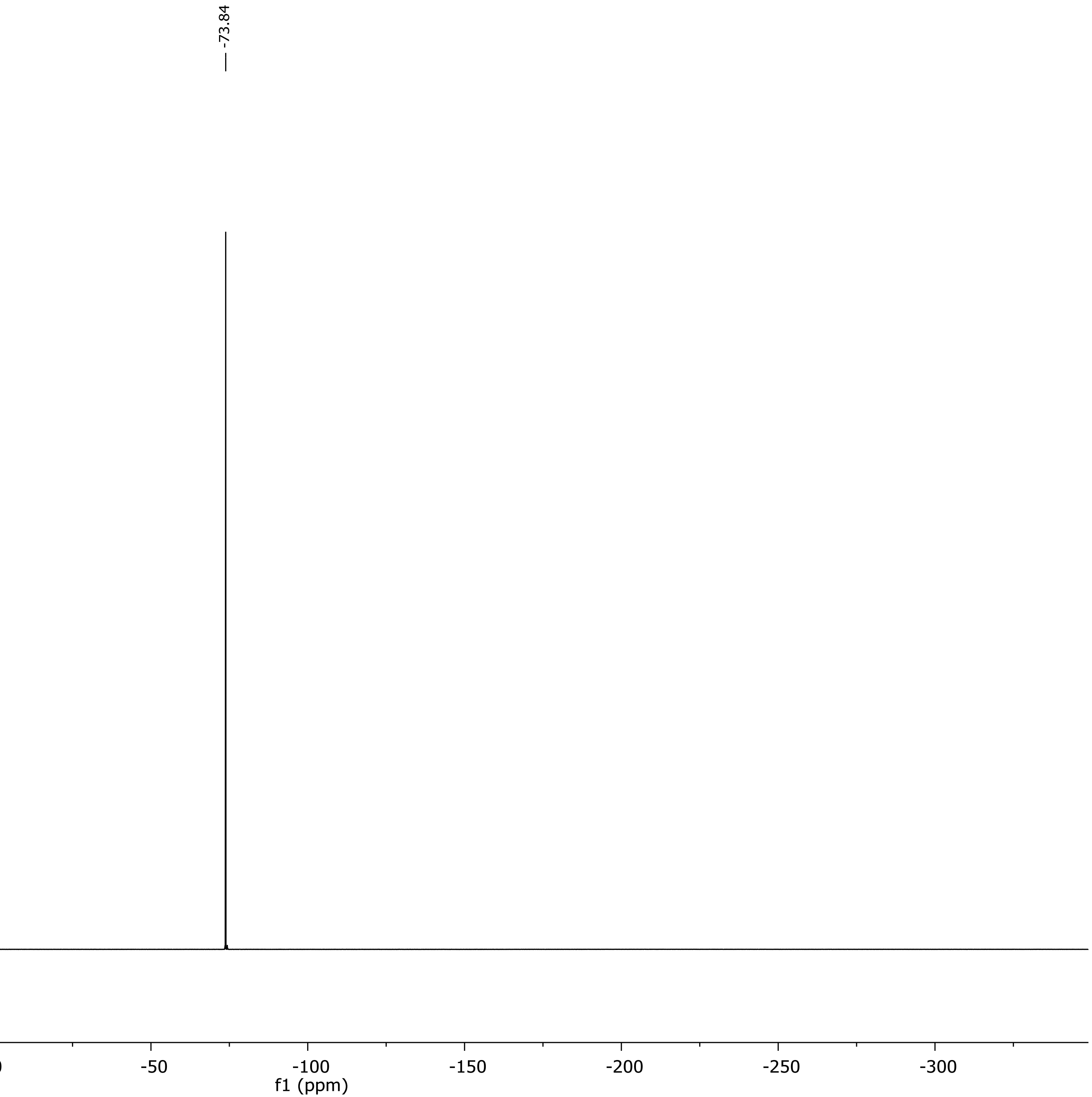

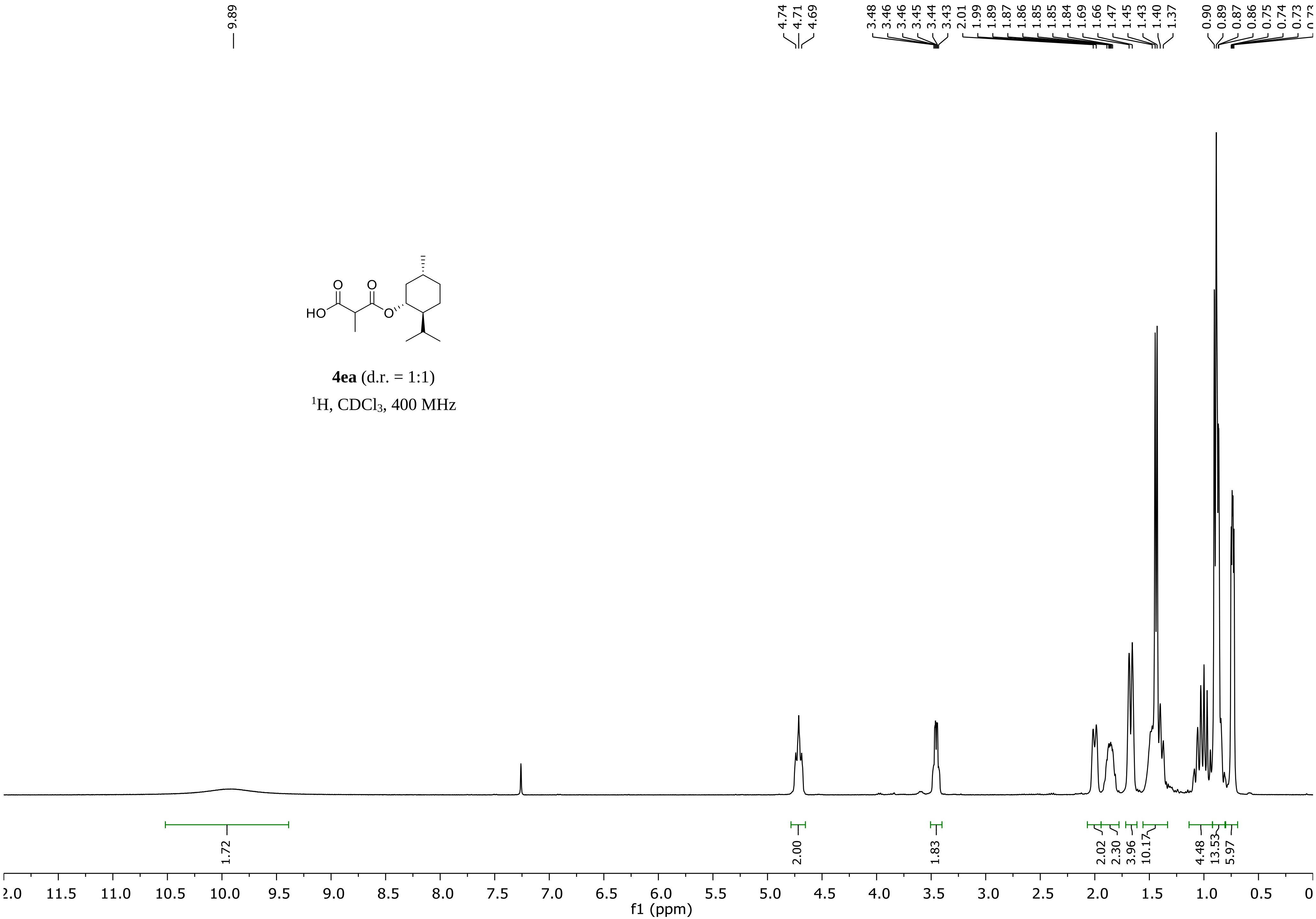

4ea (d.r. = 1:1)

^{1}H, $CDCl_3$, 400 MHz

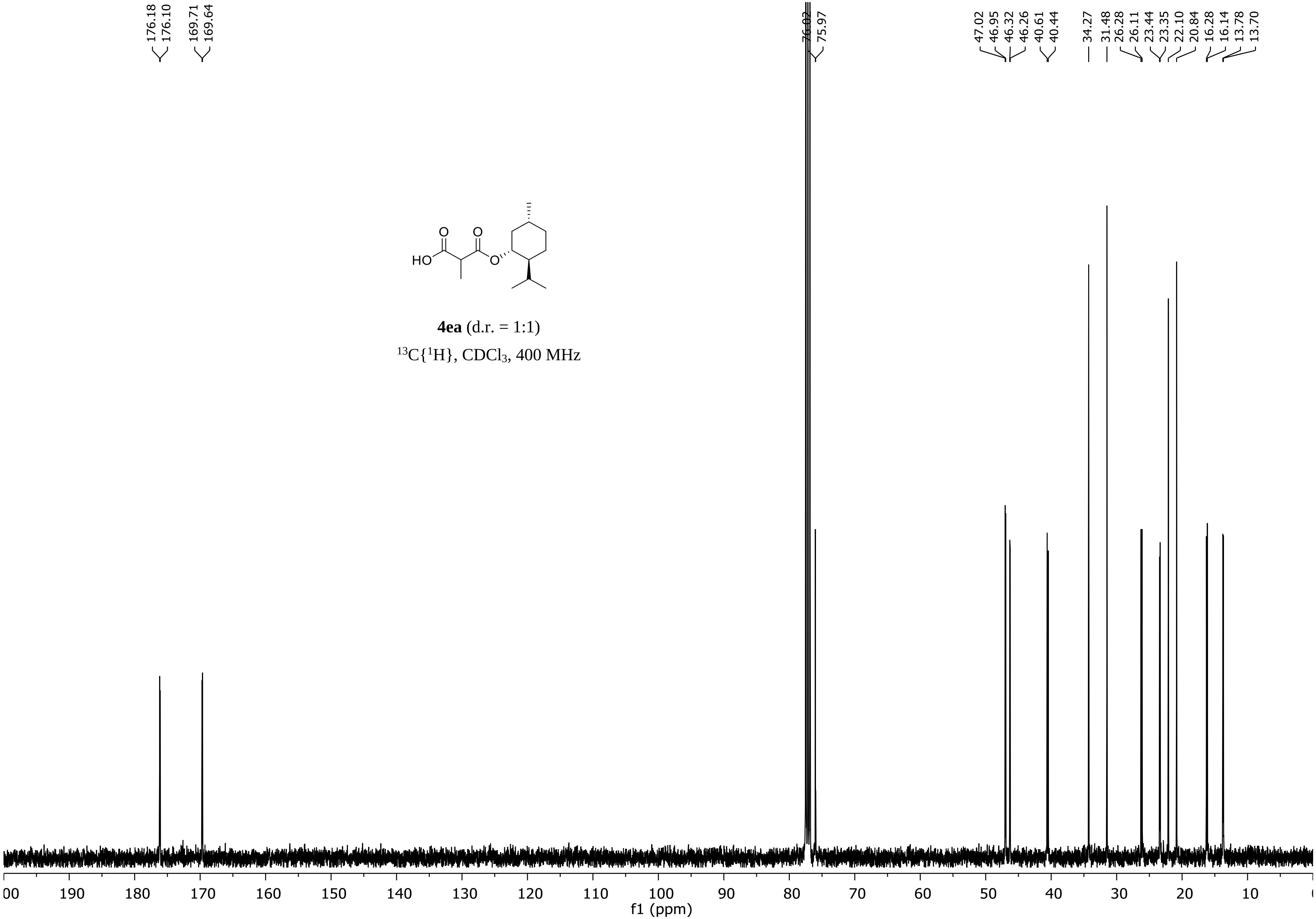

4ea (d.r. = 1:1)

$^{13}C\{^{1}H\}$, $CDCl_3$, 400 MHz

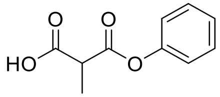

4ia

^{1}H, $CDCl_3$, 400 MHz

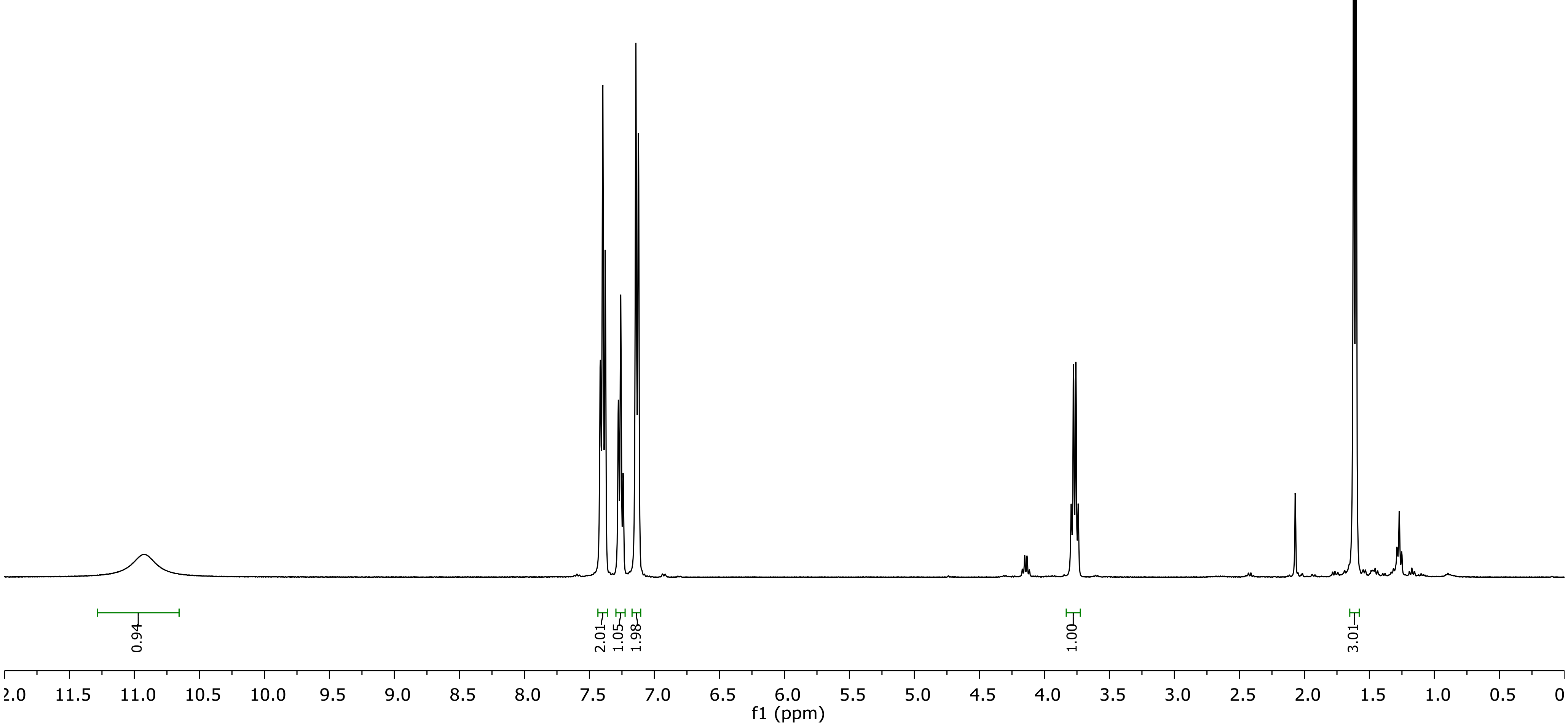

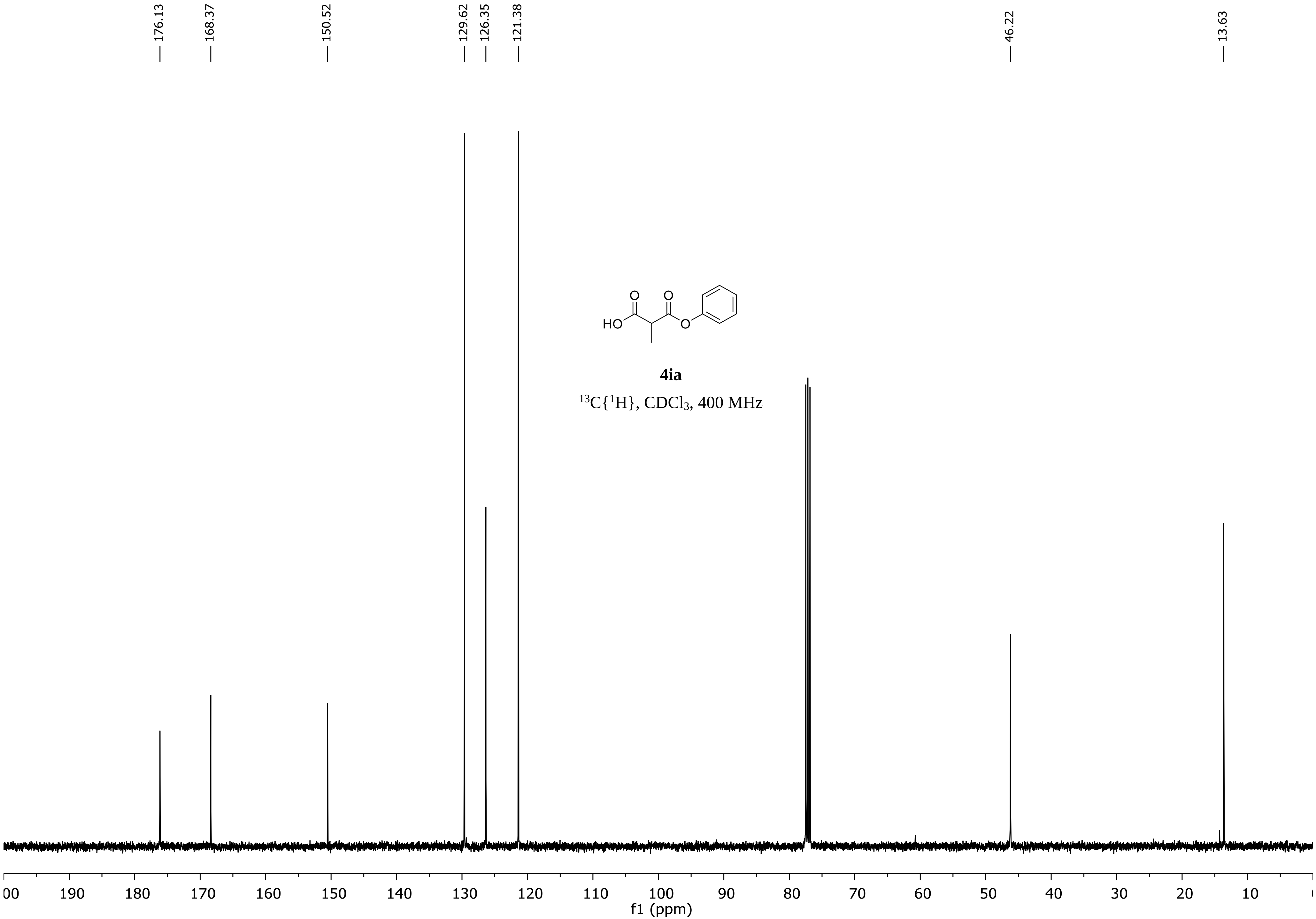
176.13
168.37
150.52
129.62
126.35
121.38
46.22
13.63
HO
O
O
O
4ia
13C{1H}, CDCl3, 400 MHz
00
190
180
170
160
150
140
130
120
110
100
90
80
70
60
50
40
30
20
10
f1 (ppm)

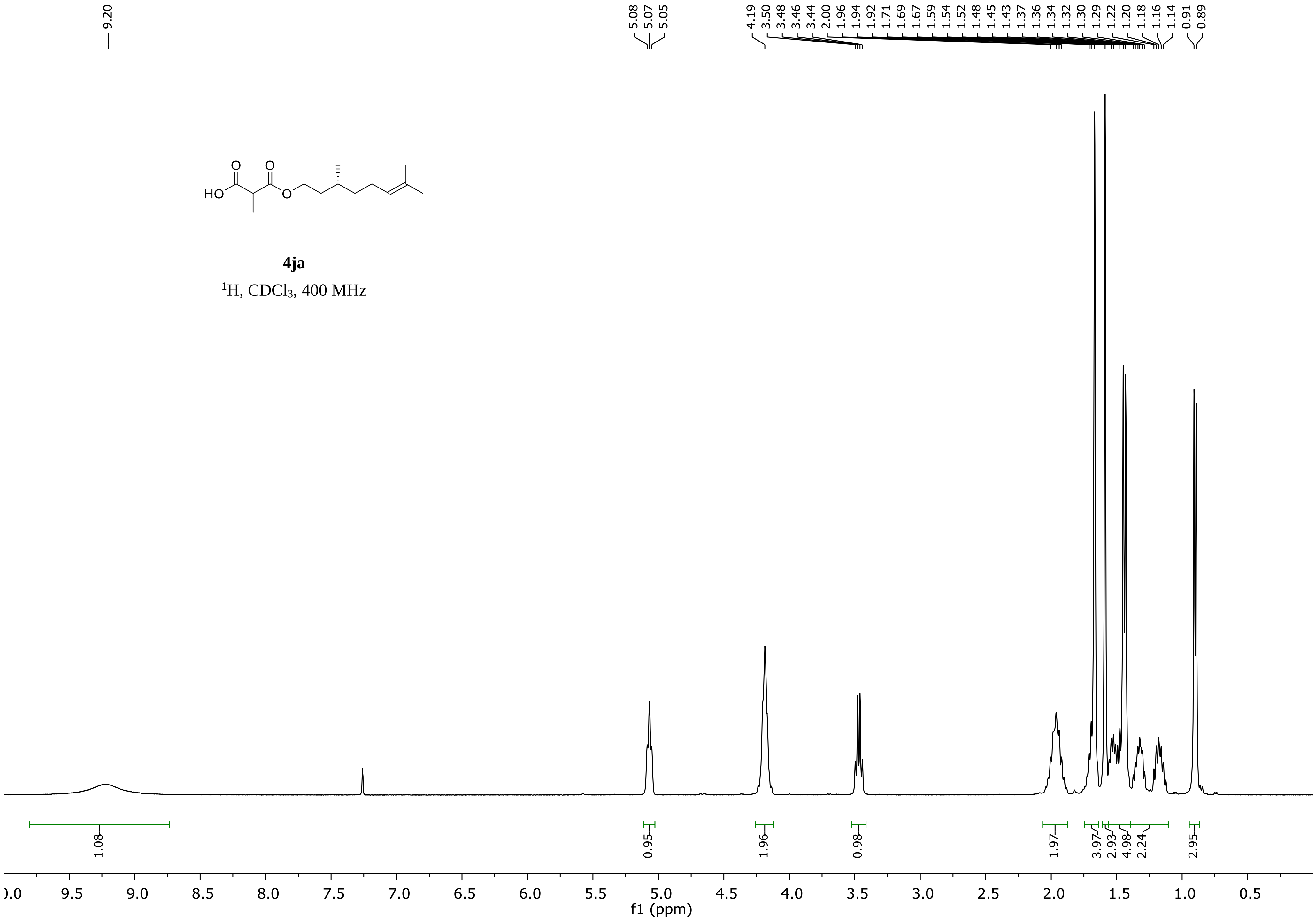
HO
O
O
O
4ja
1H, CDCl3, 400 MHz
9.20
5.08
5.07
5.05
4.19
3.50
3.48
3.46
3.44
2.00
1.96
1.94
1.92
1.71
1.69
1.67
1.59
1.54
1.52
1.48
1.45
1.43
1.37
1.36
1.34
1.32
1.30
1.29
1.22
1.20
1.18
1.16
1.14
0.91
0.89
1.08
0.95
1.96
0.98
1.97
3.97
2.93
4.98
2.24
2.95
f1 (ppm)

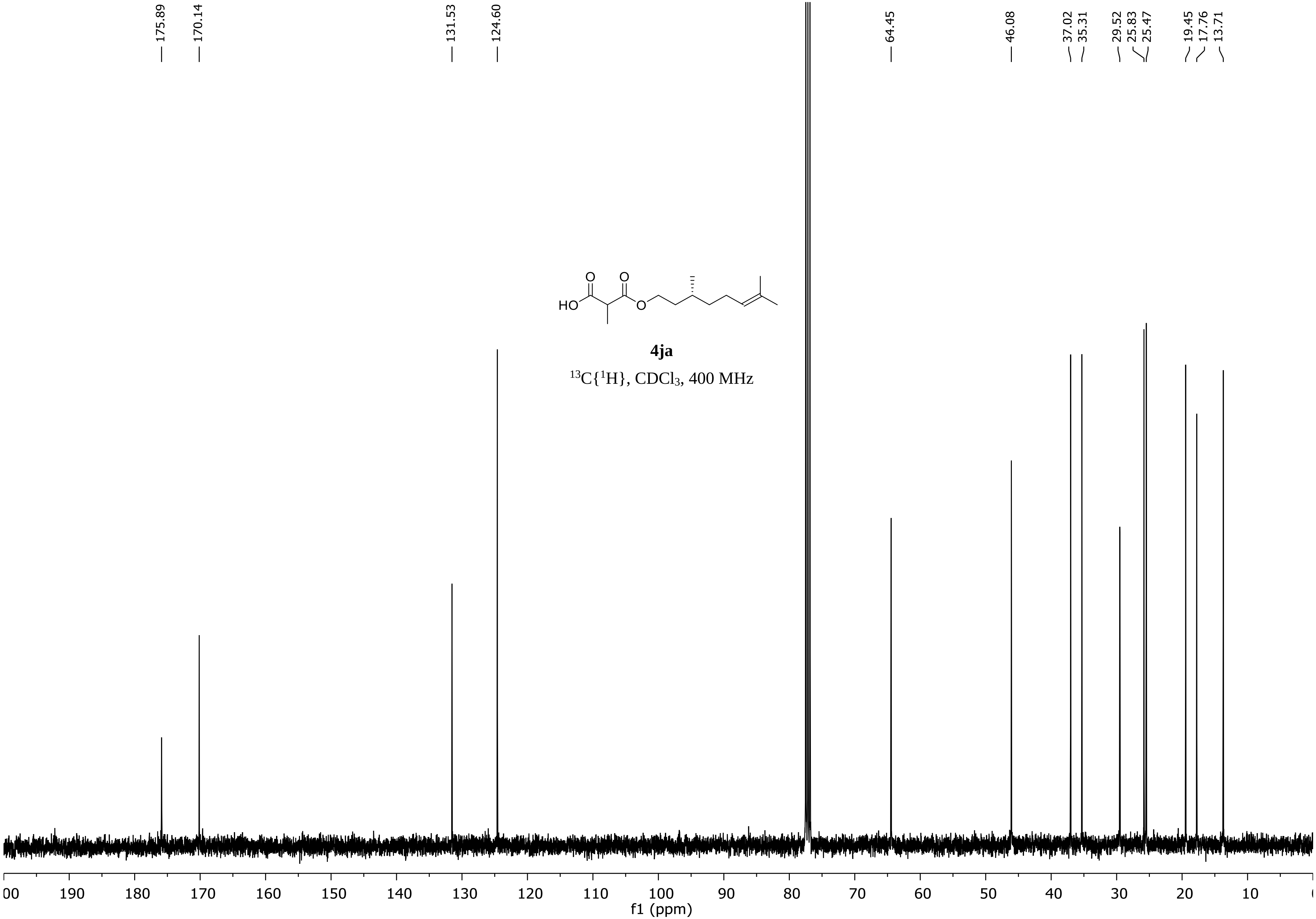
175.89
170.14
131.53
124.60
64.45
46.08
37.02
35.31
29.52
25.83
25.47
19.45
17.76
13.71
HO
O
O
O
4ja
$^{13}C\{^{1}H\}$, $CDCl_3$, 400 MHz
190
180
170
160
150
140
130
120
110
100
90
80
70
60
50
40
30
20
10
f1 (ppm)

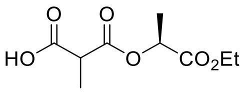

4ka (d.r. = 1:1)

^{1}H, $CDCl_3$, 400 MHz

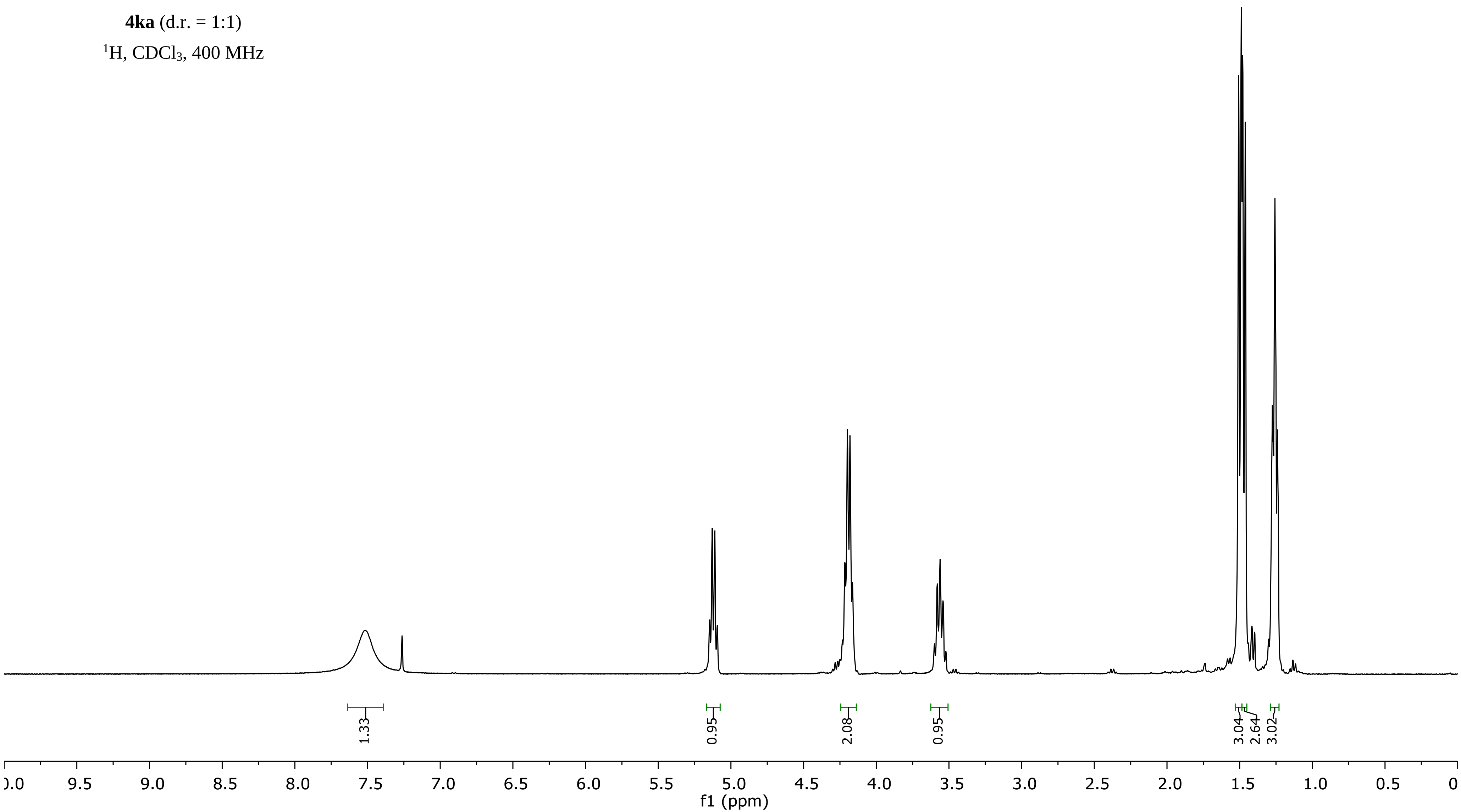

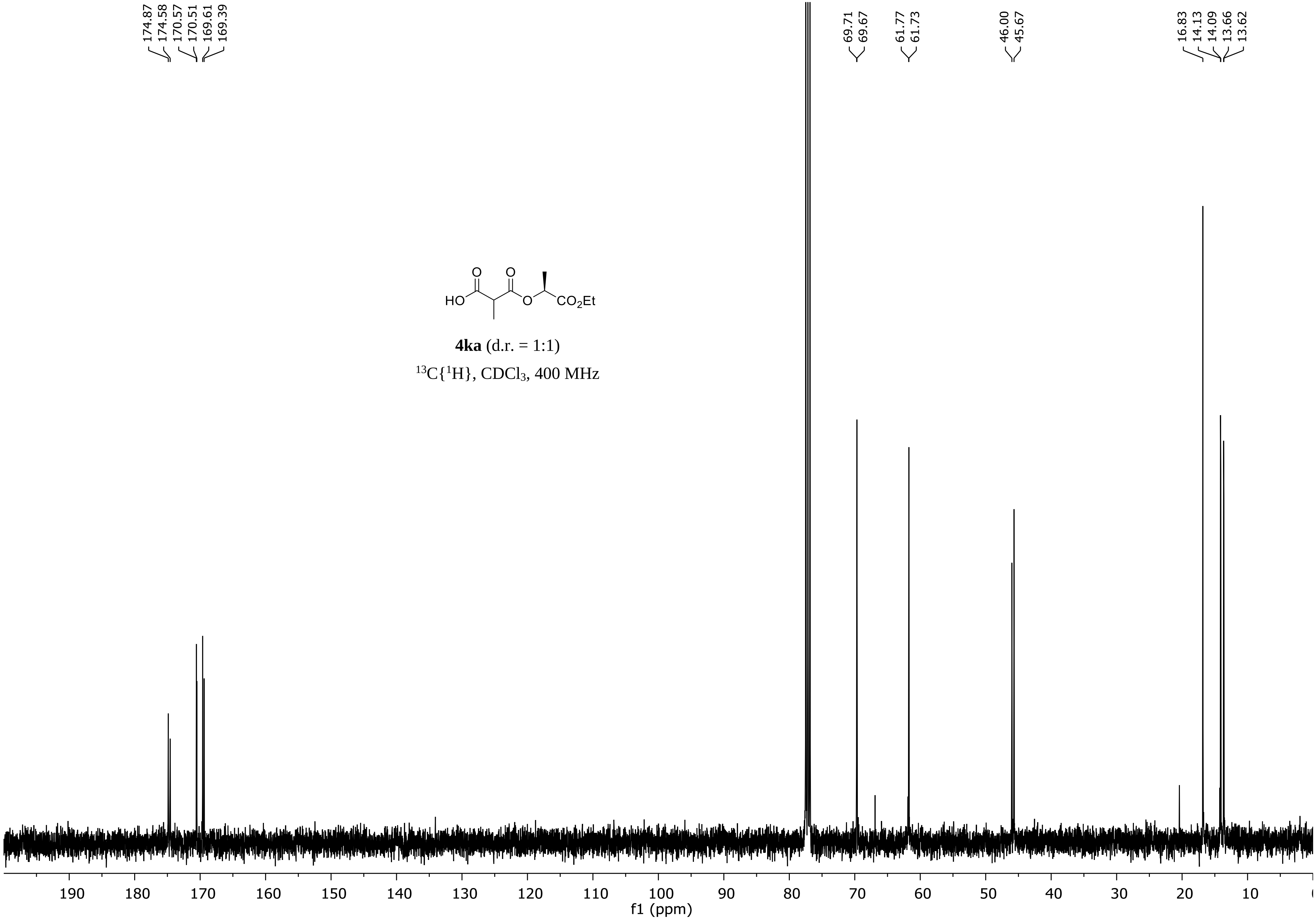
174.87
174.58
170.57
170.51
169.61
169.39
69.71
69.67
61.77
61.73
46.00
45.67
16.83
14.13
14.09
13.66
13.62
HO
O
O
O
CO2Et
4ka (d.r. = 1:1)
13C{1H}, CDCl3, 400 MHz
190
180
170
160
150
140
130
120
110
100
90
80
70
60
50
40
30
20
10
f1 (ppm)

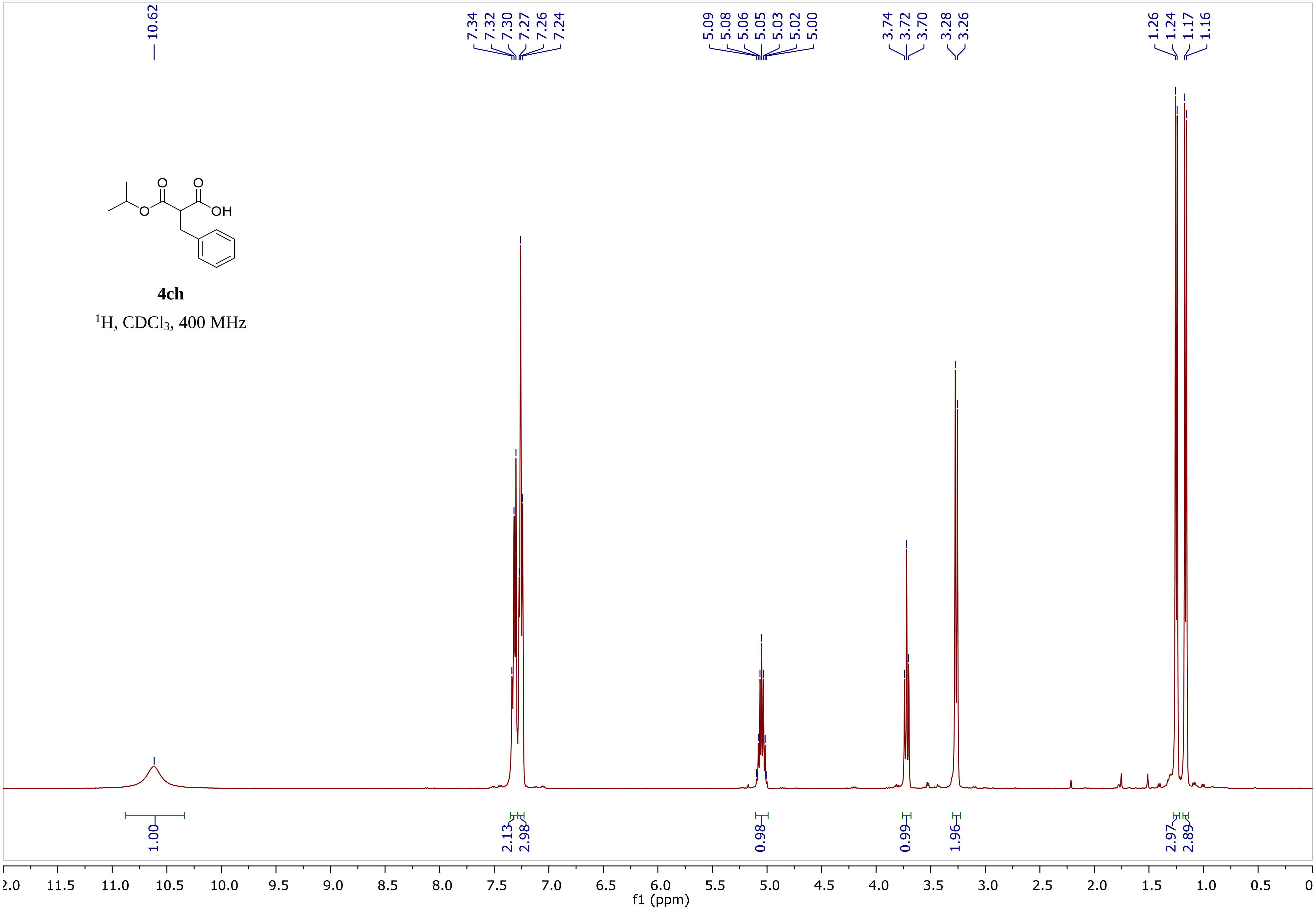
10.62
7.34
7.32
7.30
7.27
7.26
7.24
5.09
5.08
5.06
5.05
5.03
5.02
5.00
3.74
3.72
3.70
3.28
3.26
1.26
1.24
1.17
1.16
O
O
O
OH
4ch
^1H, $CDCl_3$, 400 MHz
1.00
2.13
2.98
0.98
0.99
1.96
2.97
2.89
f1 (ppm)

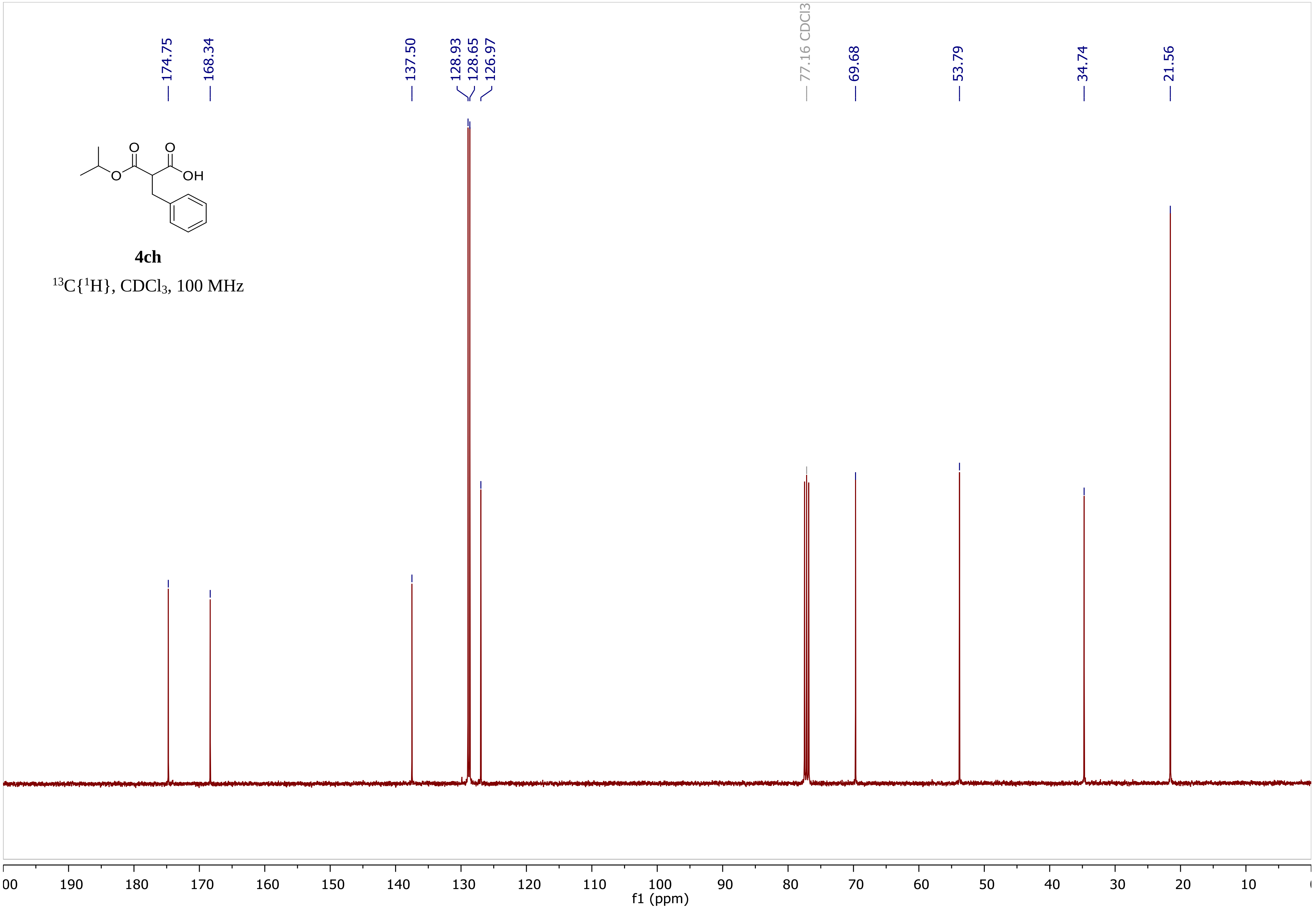

174.75
168.34
137.50
128.93
128.65
126.97
77.16 CDCl3
69.68
53.79
34.74
21.56
O
O
O
OH
4ch
$^{13}C\{^{1}H\}$, $CDCl_3$, 100 MHz
00
190
180
170
160
150
140
130
120
110
100
90
80
70
60
50
40
30
20
10
f1 (ppm)

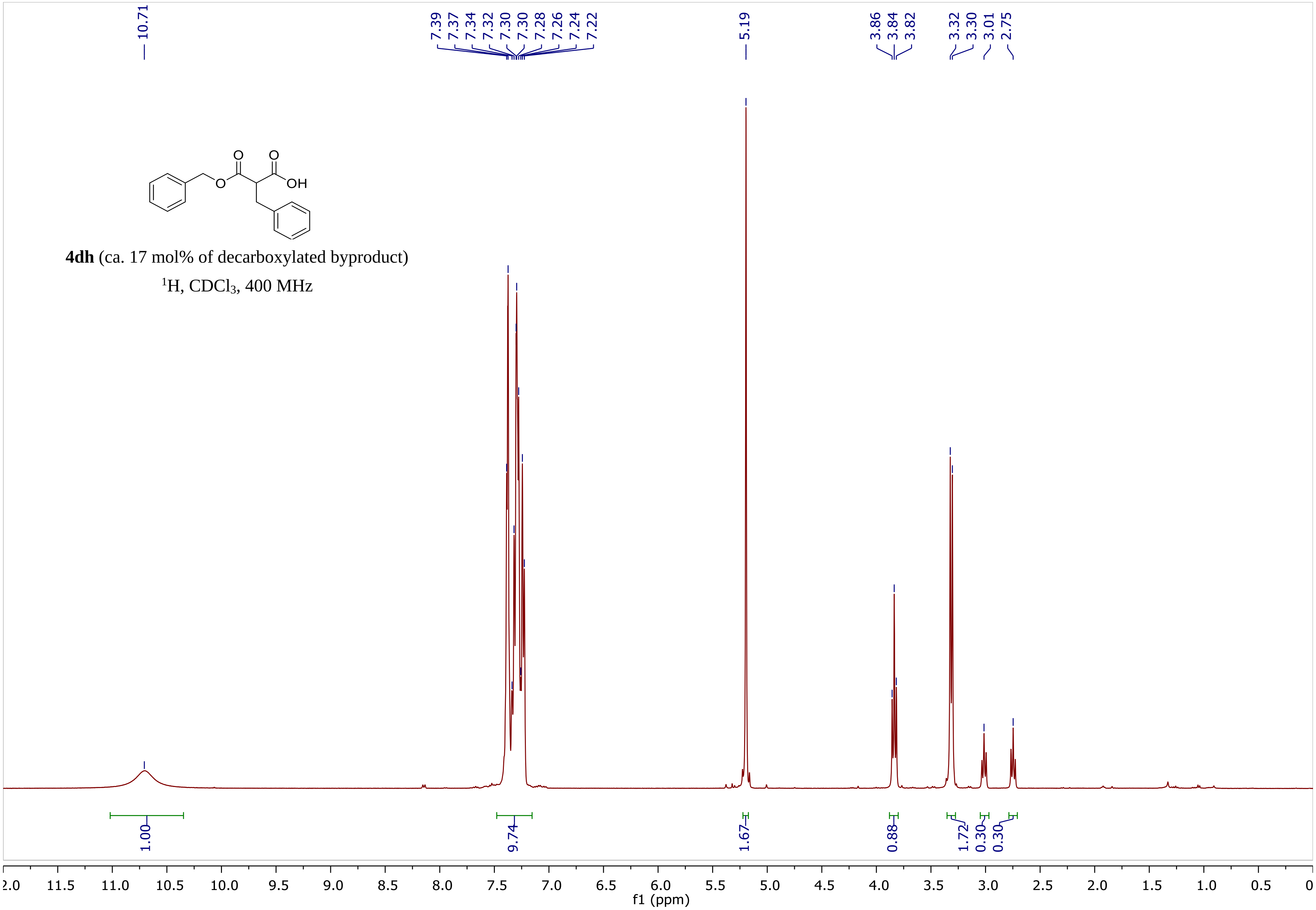

4dh (ca. 17 mol% of decarboxylated byproduct)

^{1}H, $CDCl_3$, 400 MHz

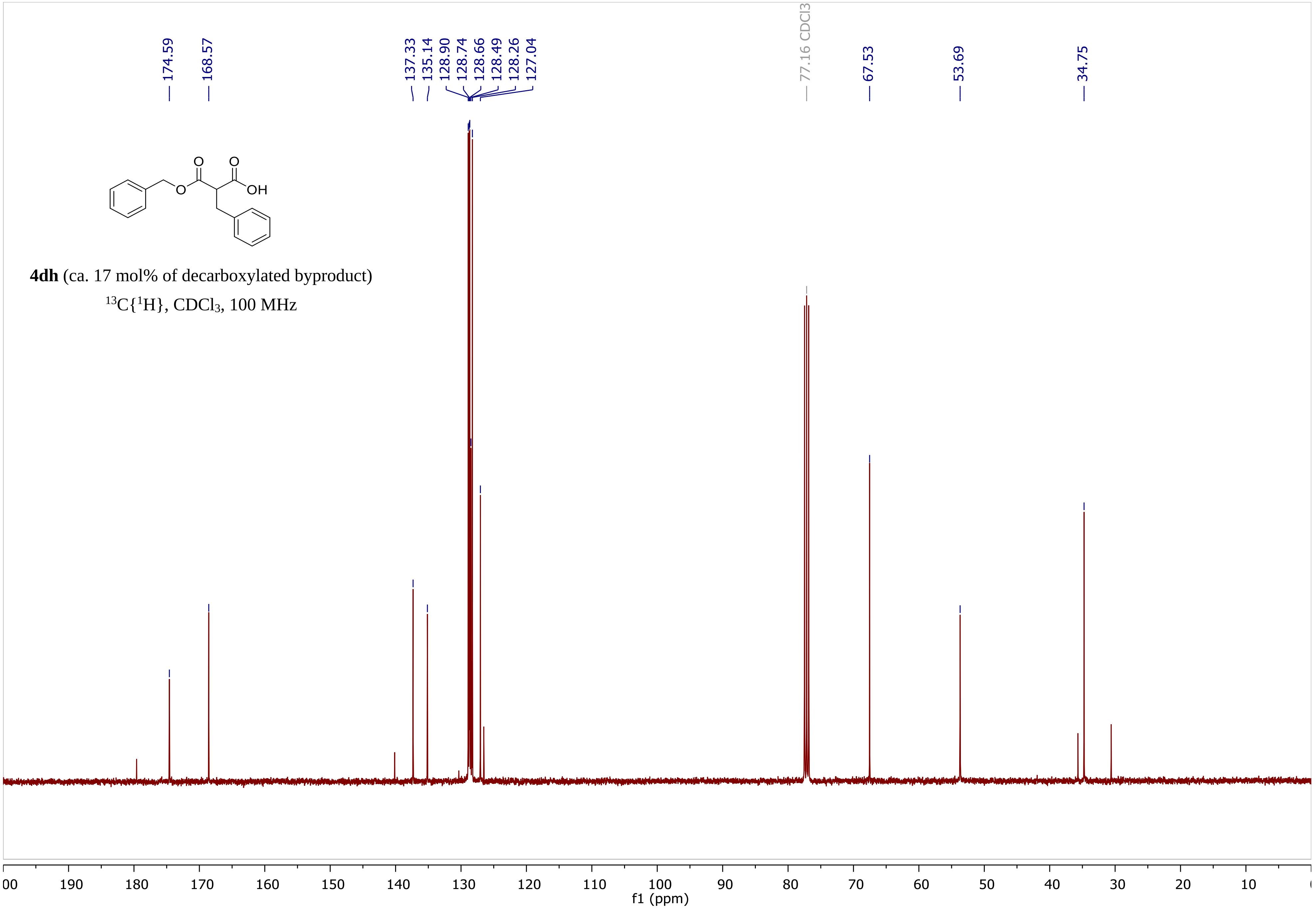

4dh (ca. 17 mol% of decarboxylated byproduct)

$^{13}C\{^{1}H\}$, $CDCl_3$, 100 MHz

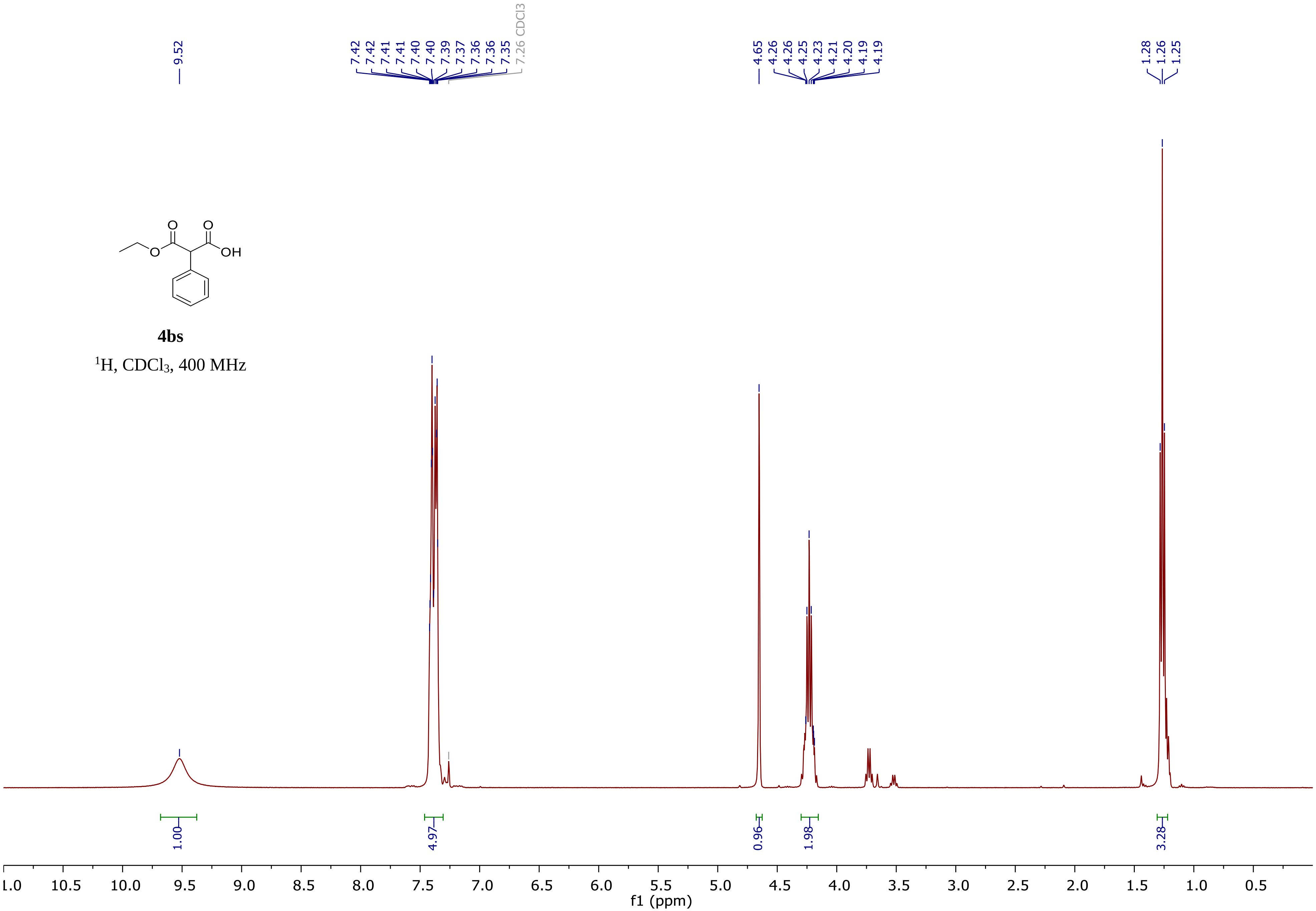
9.52
7.42
7.42
7.41
7.41
7.40
7.40
7.39
7.37
7.36
7.36
7.35
7.26 CDCl3
4.65
4.26
4.26
4.25
4.23
4.21
4.20
4.19
4.19
1.28
1.26
1.25
O
O
O
OH
4bs
¹H, CDCl₃, 400 MHz
1.00
4.97
0.96
1.98
3.28
f1 (ppm)

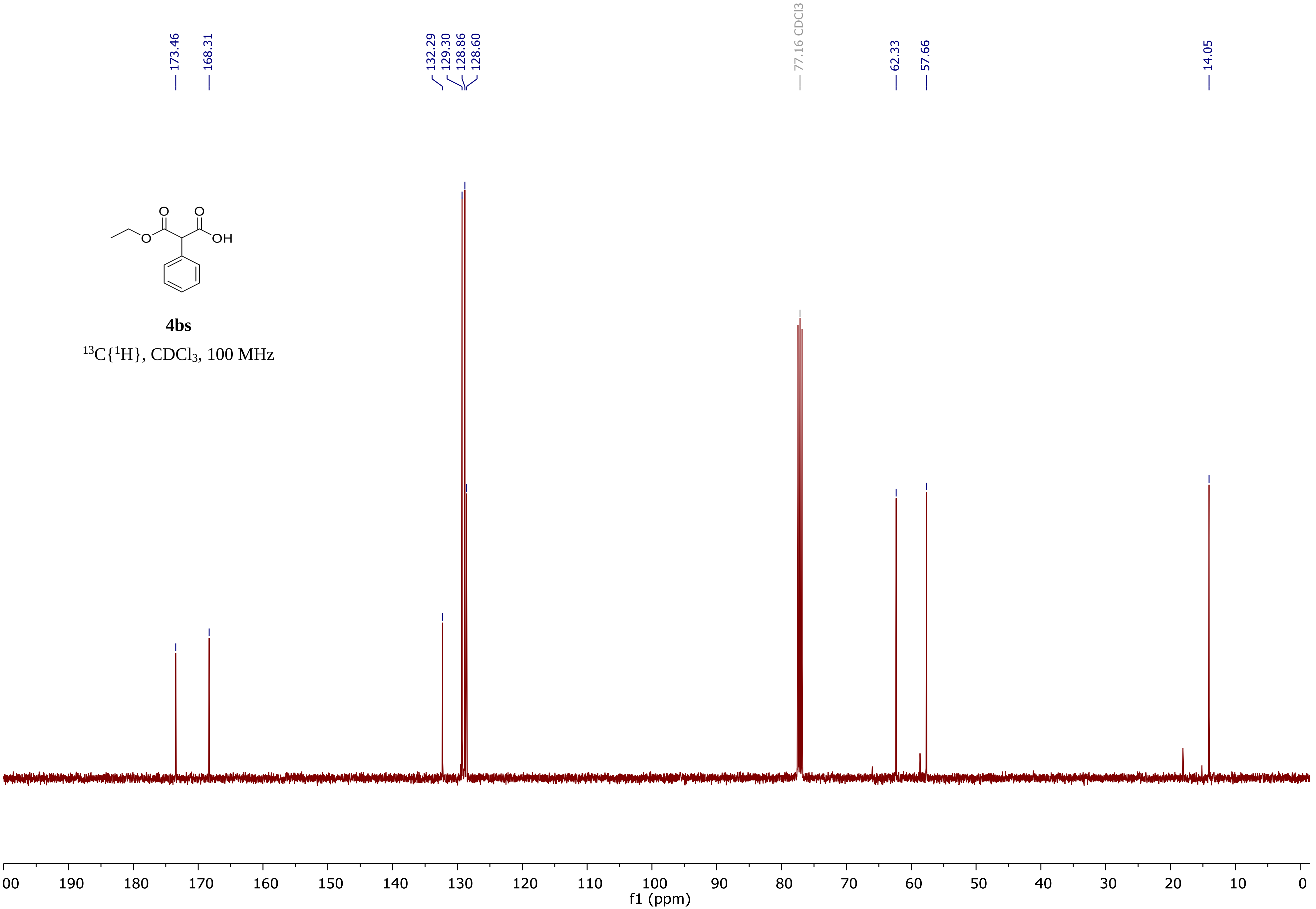

173.46
168.31
132.29
129.30
128.86
128.60
77.16 CDCl3
62.33
57.66
14.05
OH
O
4bs
$^{13}C\{^1H\}$, $CDCl_3$, 100 MHz
f1 (ppm)
